# Dynamic Network Biomarker of Pre-Exhausted CD8^+^ T Cells Contributed to T Cell Exhaustion in Colorectal Cancer

**DOI:** 10.3389/fimmu.2021.691142

**Published:** 2021-08-09

**Authors:** Jiaqi Hu, Chongyin Han, Jiayuan Zhong, Huisheng Liu, Rui Liu, Wei Luo, Pei Chen, Fei Ling

**Affiliations:** ^1^School of Biology and Biological Engineering, South China University of Technology, Guangzhou, China; ^2^School of Mathematics, South China University of Technology, Guangzhou, China; ^3^The First People’s Hospital of Foshan, Sun Yat-sen University, Foshan, China; ^4^Pazhou Lab, Guangzhou, China

**Keywords:** colorectal cancer, dynamic network biomarker, T cell exhaustion, pre-exhausted T cell, CCT6A, *TUBA1B*

## Abstract

Immunotherapy has achieved positive clinical responses in various cancers. However, in advanced colorectal cancer (CRC), immunotherapy is challenging because of the deterioration of T-cell exhaustion, the mechanism of which is still unclear. In this study, we depicted CD8^+^ T-cell developmental trajectories and characterized the pre-exhausted T cells isolated from CRC patients in the scRNA-seq data set using a dynamic network biomarker (DNB). Moreover, *CCT6A* identified by DNB was a biomarker for pre-exhausted T-cell subpopulation in CRC. Besides, *TUBA1B* expression was triggered by *CCT6A* as DNB core genes contributing to CD8^+^ T cell exhaustion, indicating that core genes serve as biomarkers in pre-exhausted T cells. Remarkably, both *TUBA1B* and *CCT6A* expressions were significantly associated with the overall survival of COAD patients in the TCGA database (*p* = 0.0082 and *p* = 0.026, respectively). We also observed that cellular communication between terminally differentiated exhausted T cells and pre-exhausted T cells contributes to exhaustion. These findings provide new insights into the mechanism of T-cell exhaustion and provide clue for targeted immunotherapy in CRC.

## Introduction

Colorectal cancer (CRC) is the third most commonly diagnosed cancer and the fourth most fatal cancer globally (1). With the entry of tumor therapy into the *PD1*/*PD-L1* immunization era, many cancers have achieved clinical success except for some CRC patients (2). Most T cells in advanced CRC patients are exhausted (3), and T cell exhaustion is considered to be an important factor affecting the efficacy of immunotherapy in patients with CRC (4). T-cell exhaustion is a process of dysfunction that commonly occurs in cancer; it is caused by antigens and persistence of inflammation (4, 5). However, the process of CD8+ T cell exhaustion in CRC is partially unclear. Exhausted T cells have been characterized by progressive loss of effector functions, high and sustained inhibitory receptor expression, homeostatic self-renewal, and distinct transcriptional (6). Additionally, it has been demonstrated that in the immune microenvironment of non-small cell lung cancer (NSCLC), there is a certain kind of pre-exhausted CD8^+^ T cell population (7), which leads to poor prognosis, during T-cell exhaustion. Thus, it is vital to find pre-exhausted T cells in CRC based on the T-cell exhaustion process. Targeting pre-exhausted T cells may have a larger clinical window than those targeting only terminal Tex cells. Single-cell RNA sequencing is a powerful tool to resolve the diversity of T cells in the CRC microenvironment. Combined scRNA-seq and DNB together, we might be able to find pre-exhausted CD8+ T cells and core functional molecular network in CRC microenvironment. However, it is unknown whether these genes play a role in driving pre-exhausted CD8^+^ T cell toward exhaustion.

Many factors contribute to T-cell exhaustion, including cytokines, transcription factors, and cell–cell interactions (4). A previous study found that the large-scale T-cell activation, release of cytokines and chemokines, and massive T cell proliferation lead to profound T-cell exhaustion in humans by the stimulation of CD3 (8). However, it is unknown whether these genes play a role in driving pre-exhausted CD8^+^ T toward exhaustion. These genes may be breakthroughs in suppressing T cell exhaustion or may be able to serve as important clinical prognostic indicators. Besides, multi-region single-cell sequencing reveals that cell-cell communication in tumor microenvironment somehow also facilitates immune escape (9). And cell-cell communication can provide additional insight to improve predicting response to therapies (10). Thus, pre-exhausted CD8^+^ T cells may contribute significantly to T cell exhaustion at both the molecular and cellular levels.

In this study, we aimed to elucidate the process of T-cell exhaustion in CRC and identify relevant targets and biomarkers in pre-exhausted T cell. To identify the biomarkers for pre-exhausted T cell in CRC, we constructed the CD8^+^ T-cell differentiation trajectory in CRC tumor tissues on the scRNA-seq data set provided by Zemin Zhang’s lab (GSE108989) (11, 12). We also used the DNB method in scRNA-seq data sets to analyze the gene network changes of CD8+ T cells in CRC tumor tissues during exhaustion and explain the functions of those core genes at network level. Lastly, we explored the cellular communication between pre-exhausted T cells and terminal Tex cells, finding how Tex cells promote pre-exhausted cells to exhaust. In conclusion, this study not only successfully identified a population of pre-exhausted T cells in the CRC immune microenvironment but also elucidated their bidirectional role in T cell exhaustion at both network and cellular levels. We hope that these results will provide novel prognosis markers or helpful targets for better immunotherapy in CRC.

## Materials and Methods

### Theoretical Basis

**Figure 1:**
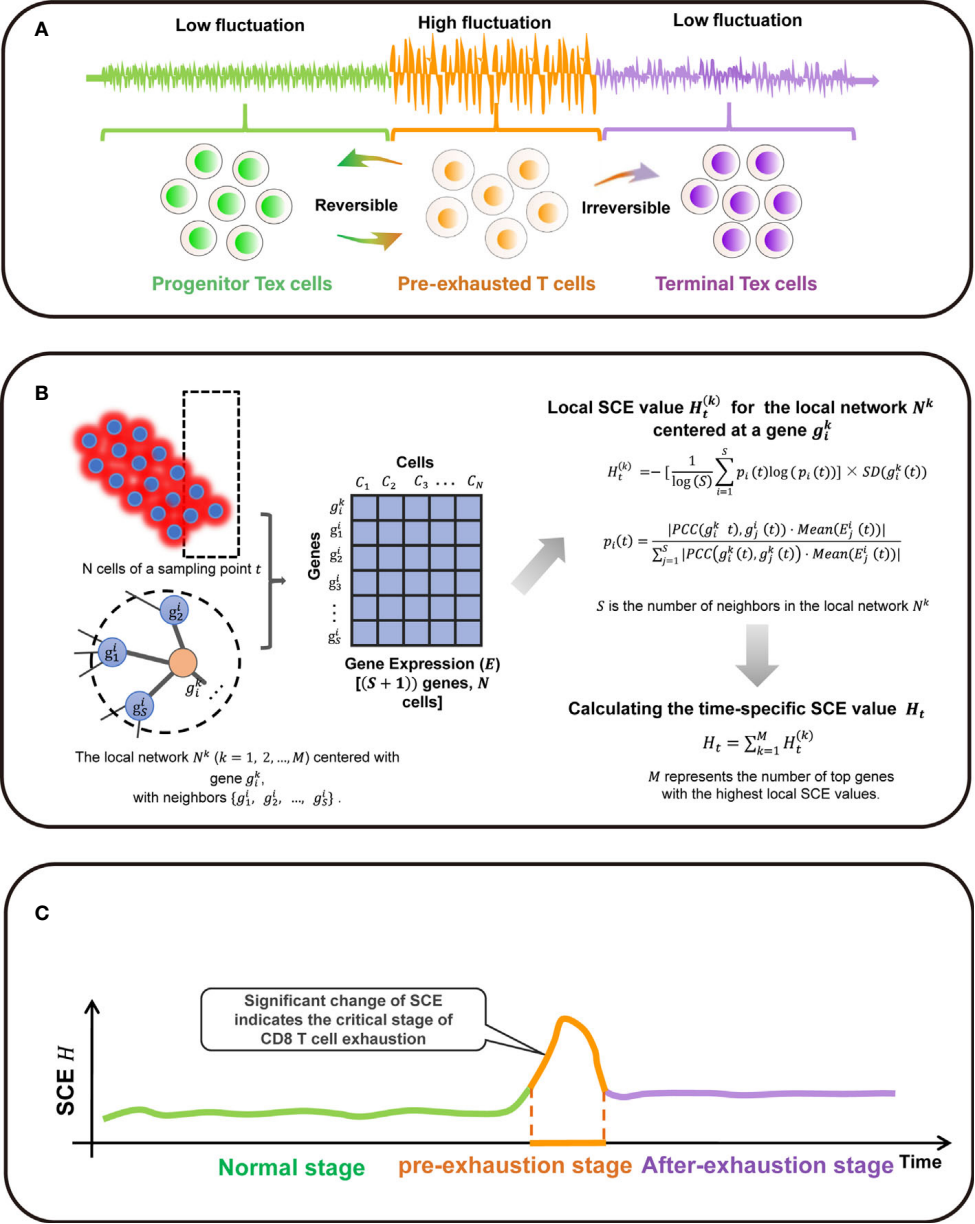
Overall project design together with algorithm details. **(A) **The three stages transition of PPI network during T cell exhaustion progression in classic dynamic network biomarker theory. **(B)** Single-cell sample-based node entropy algorithm. **(C)** A spike of the SNE curve exists in T cell exhaustion progression.

The SCE method is designed to detect a critical state before a critical transition from the relatively normal state into the exhausted state (**Figure 1A**). There exists a group of molecules defined as DNB biomolecules, which satisfy the following three statistic conditions:

1. *SD_in_* for genes inside the DNB group drastically increases, where *SD_in_* represents the standard deviation or coefficient of variation;
2. *PCC_in_* for genes inside the DNB group drastically increases, where *PCC_in_* represents the Pearson’s correlation coefficient;
3. *PCC_out_* rapidly decreases, where *PCC_out_* represents the Pearson’s correlation coefficient between any one member in the DNB group and any other non-DNB member;

The abovementioned three statistic conditions are necessary conditions for phase transition in biological system. According to that, it is obvious that the critical transition of a system is actually quantified by some variables that are strongly fluctuating. The perturbation of local networks of these variables provides the early-warning signals of the critical transition.

### Algorithm to Detect the Signal of Critical Transition Based on Single-Cell Entropy (SCE)

Given the time series of single-cell RNA sequencing (scRNA-seq) data, the following algorithm is carried out to predict the critical transition (**Figure 1B**).

**[Step 1]** Normalizing the scRNA-seq data. At each time point, the logarithm + is applied to normalize the initial gene expression matrix with *M* rows/genes and *N* columns/cells.

**[Step 2]**. Map the genes to protein-protein interaction (PPI) network defined as a global template network *N^G^*. In this work, the PPI network is downloaded from the STRING database, in which all the isolated nodes are discarded. Clearly, each individual’s PPI network is identical as the global template network *N^G^*.

**[Step 3]** Extracting each local network/subnetwork from global template network *N^G^*. Specifically, there are *M* local networks *N^k^* (*k* = 1, 2, 3, …, *M*) corresponding to its *M* genes. The local network *N^k^* is centered at a gene $g_{i}^{k}$, whose 1st-order neighbors $\left\{g_{1}^{i},g_{2}^{i},\ldots g_{S}^{i}\right\}$ are the edges

**[Step 4]** Calculating the gene-specific local SCE value $H_{t}^{\left(k\right)}$ for each local network at a sampling point *t*. Given a local network *N^k^* centered at a gene $g_{i}^{k}$, the corresponding local SCE value is obtained from

(2) $$H_{t}^{\left(k\right)}=\left[-\frac{1}{\log(S)}\Sigma_{i=1}^{S}p_{i}(t)\log(p_{i}(t))\right]\times SD(g_{i}^{k}(t)),$$

with

(3) $$p_{i}(t)=\frac{\left|PCC(g_{i}^{k}(t),g_{j}^{i}(t))\cdot Mean(E_{j}^{i}(t))\right|}{\Sigma_{j=1}^{S}\left|PCC\left(g_{i}^{k}(t),g_{j}^{k}(t)\right)\cdot Mean(E_{j}^{i}(t))\right|},$$

where$PCC(g_{i}^{k}(t),g_{j}^{i}(t))$ represents the Pearson’s correlation coefficient (PCC) between the center gene $g_{i}^{k}$ and a neighbor $g_{j}^{i}$ at a sampling point *t* and $SD(g_{i}^{k}(t))$ represents the standard deviations of the central gene $g_{i}^{k}$ at a sampling point *t*. The value $E_{j}^{i}(t)$ represents the gene expression of a neighbor $g_{j}^{i}$ and constant *S* is the number of neighbors in the local network *N^k^*. Thus, the local SCE value (Eq. (2)) is dependent not only on the expression of the center gene of a local network but also on the contribution from the neighboring genes.

**[Step 5]** Calculating the time-specific SCE value *H_t_* based on a group of genes with highest local SCE values, i.e.,

(4) $$H_{t}=\Sigma_{k=1}^{T}H_{t}^{\left(k\right)},$$

where constant *T* is an adjustable parameter representing the number of top 5% genes with the highest local SCE values. In Eq. (4), *H_t_* can be considered as the SCE score of sampling point *t* and detect the early-warning signals of the critical transition. At each time point, the specific SCE values of a certain cell population is used as the time-specific SCE score in the tipping-point detection.

When the system approaches the vicinity of the critical point, the signaling genes or dynamical network biomarker (DNB) molecules exhibit obviously collective behaviors with large fluctuations, leading to the property that the Pearson’s correlation coefficient (PCC) and gene expression of DNB members in a critical state are different from those in a before-transition state. Moreover, the term $SD(g_{i}^{k}(t))$ in Eq. (1) also brings a boost to the SCE score of the local network *N^k^*. Therefore, the local SCE value $H_{t}^{\left(k\right)}$ in Eq. (2) or the time-specific SCE value *H_t_* increases when the system is near the tipping point (**Figure 1C**).

### Data Processing and Trajectory Analysis

The scRNA-seq data of CD8^+^ T cells were from 12 CRC patients from Zemin Zhang’s lab with the accession number GSE108989 (11). The validation of bulk RNA-seq data of COAD was from the TCGA database (13). Then, the exhausted CD8^+^ T cell differentiation trajectory was inferred after dimension reduction and cell ordering in R package *Monocle* (version 2.14.0).

### Differential Expression and Functional Enrichment Analysis

We performed clustering analysis using *Seurat* (version 3.2.1) pipelines. The *FindNeighbors* parameters of 1:20 and *resolution* parameters of 1.6 were set for CD8^+^ T cells subpopulation. The function of *FindMarkers* in R package Seurat (version 3.2.1) was used to find DEG for each cluster. The DEG analysis was performed in with *DESeq2* package (version 1.26.0). Both *Gene Ontology* (GO) and *Kyoto Encyclopedia of Genes and Genomes* (KEGG) pathway enrichment analyses were performed by *clusterProfiler* package (version 3.14.3). Also, *Cluego* (version 2.5.7) plug-in of Cytoscape (version 3.7.2) was adopted for go enrichment analysis. The GSVA analysis was performed using R package GSVA (version 1.34.0). Reactome pathway analysis was performed by Reactome (https://reactome.org) (14).

### PPI Network Analysis and Web Tool

The interaction of DNB genes was conducted by *Cytoscape* (version 3.7.2) software. We exported the adjacency matrix by visualizing in *Cytoscape* (3.7.2), calculating the degree of each gene using the *CytoHubba* plugin, and selecting the top 50 genes for visualization. Functional protein interaction analysis was performed using *STRING* (https://string-db.org/). The possible interaction between cell populations was evaluated using *iTALK* (version 0.1.0) based on the curated available ligand-receptor pairs implemented in the package. Overall survival curves based on gene expression levels of COAD patients were drawn using the *GEPIA2* database (http://gepia2.cancer-pku.cn/).

### Statistics and Visualization

We used R (version 3.6.2) for statistical analysis and visualization. The detailed code is available from the link of GitHub (https://github.com/james778800/CRC_Tex_DNB). *p* ≤ 0.05 was considered to be statistically significant.

## Results

### Result 1: Trajectory of CD8^+^ T Cell Exhaustion in CRC and Identification of Cell Subtypes

**Figure 2:**
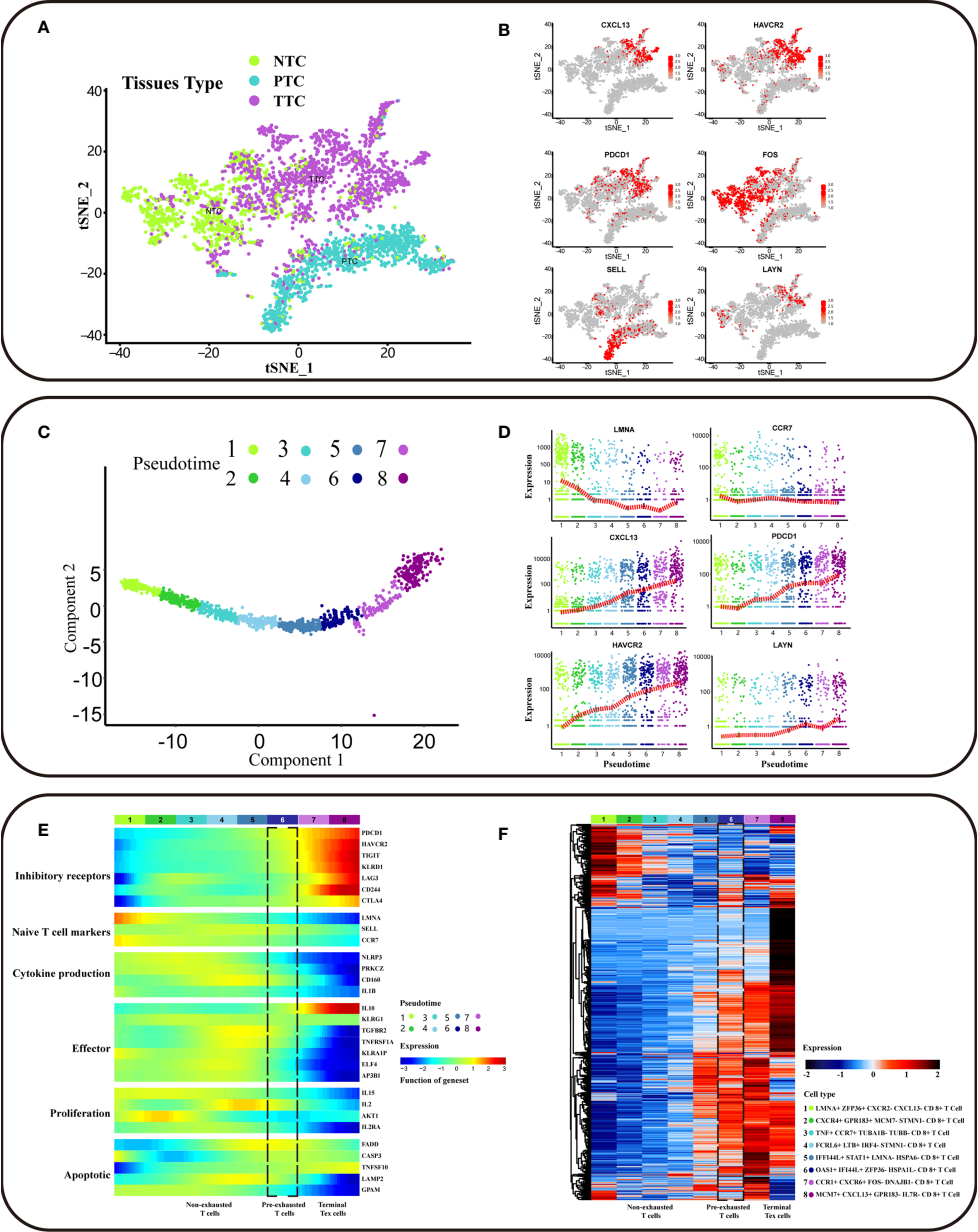
Trajectory of differentiation profiles of CD8^+^ T cells. **(A)** t-SNE clustering of T cells (n = 3628) from scRNA-seq of CRC patients showing three clusters (n = 3).** **Bright green dots for NTC (CD8^+^ T cells from adjacent normal colorectal tissues), blue-green dots for PTC (CD8^+^ T cells from peripheral blood), purple dots for TTC (CD8^+^ T cells from tumor). **(B)** The gene expression of *HAVCR2, CXCL13, PDCD1, FOS, SELL*, and *LAYN *in the scRNA-seq landscape.** **Low to high gene expression is indicated by a gradation from grey to red. The cell subpopulations with high gene expression correspond to sources referenced to the location of the NTC, PTC, and TTC clusters in **(A)**. **(C) **Potential developmental trajectory of CD8^+^ T cells in tumor tissues (n = 1646) inferred by Monocle2 based on gene expression. Pseudotime is shown numbered from 1 to 8, and the start of pseudotime is indicated. **(D)** The expression levels of *LAYN*, *CXCL13, CCR7, LMNA, PDCD1*and *HAVCR2* in different pseudotime of CD8^+^ T cell subpopulation. The x-axis represents pseudotime, and the y-axis represents gene expression. The red dash indicates the average gene expression. **(E) **The heatmap showing the dynamic expression changes of genes, including the function of T cell cytokine production, T cell apoptotic process, inhibitory receptors, cell proliferation, regulators associated with T cell exhaustion and T cell effector function. **(F) **The heatmap showing cell type-specific gene marker expression in the different T cell clusters. The marker genes for each cluster as determined by Seurat analysis with four selected genes per cluster highlighted on the top.

Single-cell sequencing captured CD8^+^ cells from different sample origins, including PTC (CD8^+^ T cells from peripheral blood), NTC (CD8^+^ T cells from adjacent normal colorectal tissues), and TTC (CD8^+^ T cells from the tumor). Based on the cluster analysis results, TTC was specific to PTC and NTC (**Figure 2A**). Exhausted T cells were present in all three tissues, and a series of genes associated with T cell exhaustion in TTC was differentially expressed genes (DEG) (**additional file: Table 1**). High expression of exhausted T-cell marker genes supported by established studies (4–6, 15), including *CXCL13*, *HAVCR2*, and *PDCD1*, was concentrated in tumor tissue in single-cell transcriptional profiles. T-cell infiltration and exhaustion was specific to tumor tissues, more severely than that in other tissues. High expression of *FOS* was specific for CD8^+^ T cells in normal CRC tissue, and *SELL* was specific for PTC. In addition, the expression of exhaustion marker gene *LAYN* was high in NTC and TTC (**Figure 2B** and **Figure S1B**). Thereafter, we extracted a sample of 1646 labeled CD8^+^ T cells from tumor tissues. Among them, CD8_C07-*LAYN* accounted for the greatest number of CD8^+^ T cells (**Figure S1A**).

CD8+ T cell exhaustion contained eight clusters based on different intracellular gene expression patterns (**Figures 2C**). The CD8+ T cell subpopulation at the initiation of differentiation trajectory showed significantly high expression of LMNA and CCR7 genes (**Figures 2D**). Associations between PD-L1/PD-1 expression were analyzed, and PDCD1 expression was found to be increased with T-cell exhaustion. As T-cell exhaustion had not yet started, the expression of naïve T cell markers was high, besides the expression of inhibitory receptor (IRS) genes was low (**Figures 2D, E**). In the exhaustion process, non-exhausted and pre-exhausted and terminal exhausted T-cell subpopulations have unique characteristics. In addition to the high expression of IRS genes with the exhaustion of T cells, the key functions genes in T cell were also changed. The low expression of T cell effector function and cytokine production genes with the exhaustion of T cells, except for IL10. In addition, IL-10 was the STAT3-inducible cytokine associated with attenuated T cell activation (6). IL10 expression was high in terminal Tex cells, suggesting that immune response was decreased. In contrast, the expression of IL10 in pre-exhausted T cells was lower than terminal Tex cells, suggesting that pre-exhausted T cells may retain some immune response function. The expression of CD160 in pre-exhausted T cells was higher than terminal Tex cells, suggesting that the cytolytic effector activity of pre-exhausted T cells may begin to weaken. Besides, non-exhausted T cells can maintain cellular function and homeostatic self-renewal in an antigen-independent manner via the cytokines IL-15 (16, 17). However, the expression of IL15 was low in terminal subsets. The terminal subsets were unable to mediate homeostatic self-renewal through IL-15 and the low expression of IL15 in pre-exhausted T cells. The low expression of cell proliferation genes includes IL-2, with the exhaustion of T cells. IL-2 cytokines were involved in T-cell activation and proliferation (18, 19). The expression of IL2 was low in terminal Tex cells, and terminal subsets were inactive in responding to additional proliferative signals. Besides, the expression of IL2 was lower in pre-exhausted T cells than non-exhausted subsets, implying that the proliferative effect of pre-exhausted T cells begins to be gradually lost due to persistent antigenic signaling. The expression of genes related to apoptosis, including FADD, CASP3, LAMP3, and TNFSF10, was low in both terminal Tex cells and pre-exhausted T cells. This suggested that T- cell apoptosis was disrupted with the exhaustion of T cells (**Figure 2E**).

To identify the specific cell populations involved in exhaustion, we performed DEG analysis for different subpopulations of CD8+ T cells. We labeled the CD8+ T cell subpopulation at initiation of differentiation trajectory as LMNA+ ZFP36+ CXCL13− CXCR− CD8+ T cells. This subpopulation may have exhaustion potential. Also, it still has the normal immune functions, with high expression of progenitor-like CD8+ T cell marker genes (**Figure 2D**). In contrast, the CD8+ T cell subpopulation at end of differentiation trajectory showed high expression of inhibitory receptor (IRS) genes, including CXCL13 and HAVCR2. CXCL13 and HAVCR2 were DEGs in this subpopulation (**additional file: Table 2**). In addition, we observed that terminal exhausted T cell subpopulation are different. We labeled the terminal exhausted T cell as MCM7+ CXCL13+ GPR183- IL7R- CD8+ T cell and CCR1+ CXCR6+ FOS− DNAJB1− CD8+ T cell (**Figure 2F**). In conclusion, we explored the trajectory of CD8+ T cell exhaustion in CRC clinical tumor tissues.

### Result 2: *CCT6A* Identified by DNB Was a Biomarker for Pre-Exhausted T Cell Subpopulation Associated With CRC Survival

**Figure 3:**
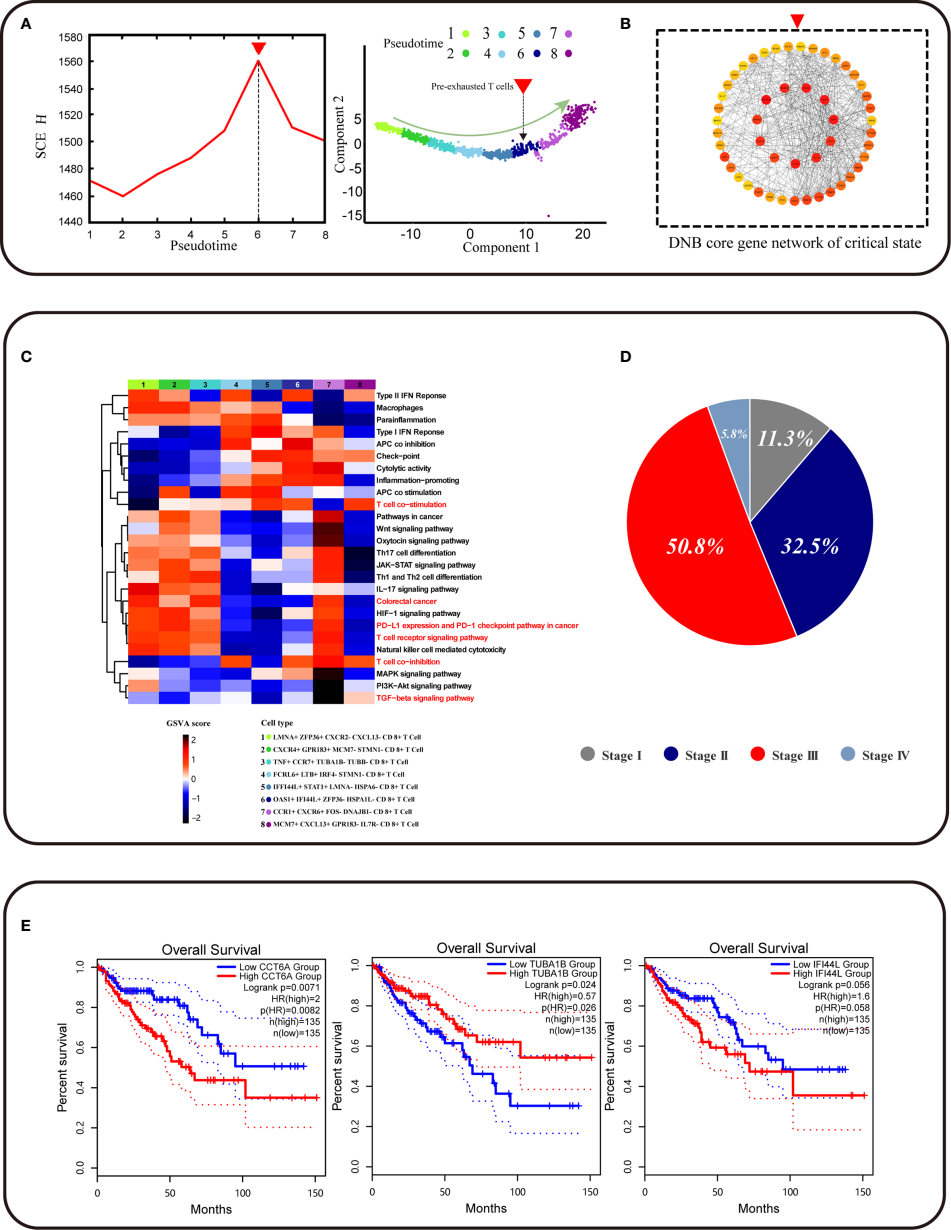
Identification of CD8^+^ T cell subtype and core genes survival analyses. **(A) **The graph on the left shows that the curve of SNE score H defined in *Methods* suddenly increases when the system is near the critical point (p = 0), which is viewed as a critical state transition at a bifurcation point. The graph on the right shows that pre-exhausted T cells were at critical state and CD8+ T cell developmental trajectory is same as **Figure 2C**. **(B)** The graph represents the gene regulatory network for the DNB core genes. **(C)** Function analysis (GSVA) shows that different gene sets may play a different role in CD8^+^ T cell exhaustion progress. **(D) **Pie chart representing the proportion of patients associated with different periods of colorectal cancer. **(E)** *CCT6A* *TUBA1B* *IFI44L* survival analyses using the TCGA COAD dataset.

We found a pre-exhausted CD8^+^ T cell subpopulation using the DNB method, with a strong signal of the critical state before CD8^+^ T cell exhaustion by a significant change of single-cell entropy (SCE) at the sixth period (**Figure 3A**). We labeled the sixth-period subpopulation as *OAS1* ^+^ *IFI44L* ^+^ *ZFP36* ^−^ *HSPA1L* ^−^ CD8^+^ T cells. Notably, *OAS1*, the pre-exhausted marker, was the DEG for these pre-exhausted CD8^+^ T cells and a DNB gene, indicating that the cell subpopulation started to show exhaustion characteristics and could be defined as the pre-exhausted CD8^+^ T cells. We obtained a total of 230 DNB genes. At the critical point, the DNB module genes fluctuated enormously with a high deviation in their gene expression and were highly correlated with the module inside. Thus, we constructed a network for DNB core genes, ranked high by the molecular degree in the network, such as *CCT2*, *CCT5*, *EIF4A3*, and *CCT6A*, which may play a key role in CD8^+^ T cell exhaustion (**Figure 3B**, **Figure S1C** and **additional file: Table 3**). GO enrichment analysis of DNB genes indicated that three GO terms were significantly enriched, including the regulation of hematopoietic stem cell differentiation, regulation of hematopoiesis, and type I interferon signaling pathway (**Figures S2A–C**).

To further identify functional changes of core DNB genes in the pre-exhausted CD8+ T cells defined by DNB, the gene set variation analysis (GSVA) analysis of these CD8+ T cells was performed. The results indicated that the correlation of T cells gradually showed functional degradation with the development of exhaustion and functional correlation in T cell co-stimulation. Functional changes were also associated with the progressive loss of normal immune function of exhausted CD8+ T cells (**Figure 3C**). The progressive functional increase in type1 IFN response associated with IFNG, as a DNB gene member that functions in the pathway, affects immune function. With the accumulation of cytotoxicity in CD8+ T cell differentiation, pre-exhausted CD8+ T cells gradually enter an exhausted expression pattern. We found apparent changes in the CRC pathway that showed a relationship with CRC progression. Therefore, we found a pre-exhausted T cell subpopulation with gene expression characteristics and functional transition states during T cell exhaustion.

Moreover, we found an increase in the percentage of pre-exhausted CD8+ T cells in different stages of CRC patients in the progression from stage II to stage III of cancer, indicating that the direction of potential pre-exhausted T cells differentiation was correlated with cancer progression (**Figure 3D**). The expression of genes related to the pre-exhausted T cell subpopulation was validated using TCGA data. IFI44L was the DEG for pre-exhausted CD8+ T cells, and the dataset of COAD in TCGA showed a correlation between this gene and patient survival (p = 0.058). CCT6A as the core gene of DNB gene network expression was significantly associated with the overall survival of COAD patients (p = 0.026) (**Figure 3E**). The DNB core gene CCT6A, identified using the DNB method, plays a vital role in the DNB core gene network. In addition, it is a biomarker for pre-exhausted T cell subpopulations associated with CRC survival.

### Result 3: *CCT6A* Drives TUBA1B Expression Overturn Changes Contributed to CD8^+^ T Cell Exhaustion

**Figure 4:**
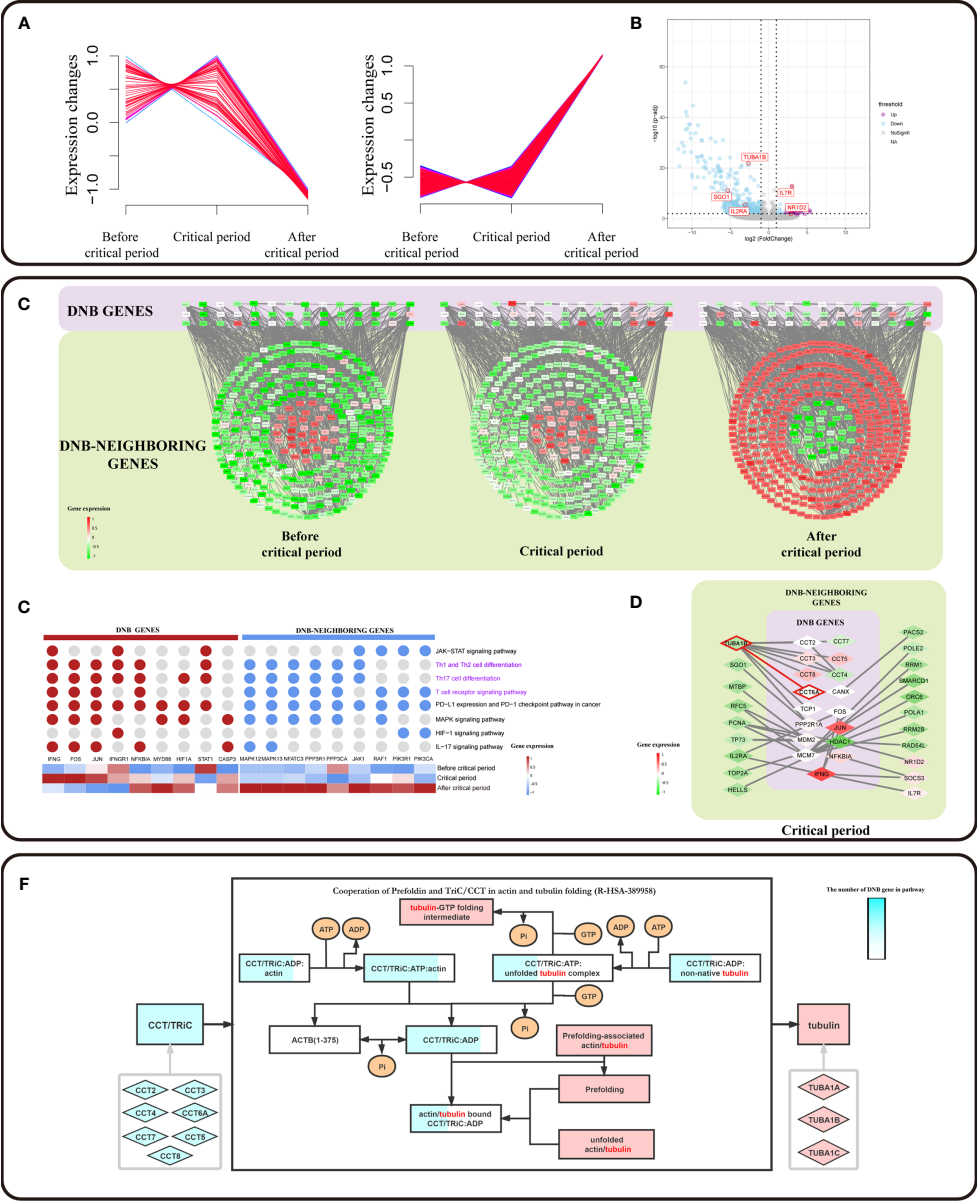
DNB core genes drive the flipped expression of DNB-neighboring genes. **(A)** The series of diagrams illustrate the patterns of dynamic changes in DNB-neighboring genes between exhaustion critical period and terminally exhausted period using Mfuzz. The x-axis represents three T cell exhaustion periods, and the y-axis represents gene expression. The dash indicates the dynamic genes expression. **(B)** Volcano plot of DEGs between pre-exhausted T cell subpopulation and terminal Tex cell subpopulation. Purple dots represent differentially upregulated genes, blue dots represent differentially down regulated genes, and grey dots represent non-significant genes (p value <0.05 and |logFC|>1). **(C)** Cytoscape visualization of DNB genes and DNB-neighboring genes interaction network, including three T cell exhaustion periods. The network nodes arranged in squares from the purple region are DNB genes. The network nodes arranged in circles from the dark green region are DNB-neighboring genes. The genes and their location in the network are the same for all three different exhaustion states, and low to high gene expression is indicated by a gradation from green to red. **(D)** Genes associated with pathways located in immune-related pathways (pathways associated with T cell pathways are indicated in purple). Expression of DNB genes in the pathway indicated by a gradation from blue to red. **(E)** Cytoscape visualization of DNB genes and DNB neighboring-DEG interaction network in the critical state. The network nodes from the purple region are DNB genes. The network nodes from the dark green region are DNB-neighboring-DEGs. Expression of genes indicated by a gradation from green to red. **(F)** Reactome pathway analysis results contain Cooperation of Prefoldin and TriC/CCT in actin and tubulin folding (R-HSA-389958) in human biology. The light blue rhombus represents the CCT gene family and pink represents tubulin genes. Squares represent the biological molecule. The blue area in the square represents the number of genes in the overexpression pathway.

Based on a previous study, DNB members have been considered leading factors, situated at important positions of pathways that regulate vital immune-associated processes during cell differentiation initiation and development (20, 21). DNB members could affect DNB-neighboring genes. We used a soft clustering algorithm to classify DNB-neighboring genes according to their expression trends and found that the gene expression levels of the CD8+ T cell subpopulation were altered between the critical period and after the critical period (**Figure 4A**). In addition, the critical period identified by the DNB method was considered as the period of pre-exhausted T cells (**Figure 3A**). These cells became terminal Tex cells after the critical period. Besides, a series of DNB-neighboring DEG was not only the DEG between pre-exhausted T cells and terminal Tex cells, but also as DNB-neighboring gene, including *TUBA1B, IL7R, SGO1* and *IL2RA* etc. (**Figure 4B** and **additional file: Table 4**).

To systematically investigate the roles of DNB core genes in pre-exhausted T cell subpopulation, we constructed a protein-protein interaction (PPI) network for DNB and DNB-neighboring genes. We found a network with a DNB core module that showed different expression patterns. DNB-neighboring genes showed expression turnover after the critical state (**Figure 4C**). As a result, those DNB-neighboring genes were considered as reversed genes, which had variable expression over time at the network level and played an important role in the T cell exhaustion. In addition, DNB core genes interacted with reversed genes in the network, possibly changing gene expression patterns or regulating downstream relationships. Remarkably, 69.66% of the reverse genes were enriched in immune effect-related KEGG pathways, including the *JAK-STAT* signaling pathway, *PD-L1* expression, and *PD-1* checkpoint pathway in cancer, Th1 and Th2 (**Figure 4D**). These pathways have upstream and downstream regulatory relationships between DNB genes and DNB-neighboring genes; transcription factors, such as *FOS*, *JUN*, and other transcription factors, are associated with DNB genes. *IFNG* as a member of the DNB gene plays a central role in this pathway. We localized the DNB genes and DNB-neighboring genes in the enriched pathway based on the gene expression characteristics that signify the pseudotime trajectory of CD8^+^ T cell exhaustion. Accordingly, we observed that the DNB-neighboring genes have turnover gene expression changes before and after the critical state and play a key role in immune-related pathways (**Figure S3A**), particularly *PD-L1* expression and *PD-1* checkpoint pathway in cancer.

Furthermore, we extracted the sub-networks of DNB-neighboring DEG and DEGs, which were from the network of DNB genes and DNB-neighboring genes. These DNB-neighboring DEGs were driven by the DNB core genes. We observed CCT family genes act as DNB core genes driving overturn changes in *TUBA1B* (**Figure 4E** and **Figure S3C**). We found that the expression of *TUBA1B*, one of the current therapeutic targets for cancer drugs, increased simultaneously with the expression of DNB gene *CCT6A*. *TUBA1B*, a DNB-neighboring DEG in pre-exhausted CD8^+^ T cells, was significantly associated with the overall survival of CRC patients (*p* = 0.0082) (**Figure 3E**). In addition, in TCGA colon and rectal cohorts combined cancer data sets, the CCT gene family has a synergistic expression pattern at different stages, whereas the CCT gene family has a similar expression pattern to *TUBA1B* in normal tissues and tumor tissues. Besides, the expression of *CCT6A* and *TUBA1B* in normal tissues had a correlation of 0.76, indicating a significant correlation. In contrast, the correlation in tumor tissues was low and insignificant (**Figures S3C, D**). The protein encoded by *CCT6A* is a molecular chaperone that is a member of the chaperonin containing the TCP1 complex (CCT), also known as the TCP1 ring complex (TriC). Reactome pathway analysis showed that unfolded tubulins bound to prefoldin were transferred to CCT *via* a docking mechanism. TriC/CCT forms a binary complex with unfolded β-tubulin. In actin and tubulin folding, the emerging polypeptide chain is transferred from the ribosome to TriC *via* prefoldin (**Figure 4F**). With the interactions between TriC/CCT and tubulins, *CCT6A* expression influenced the assembly of the TriC/CCT complex. Therefore, *CCT6A* drives *TUBA1B*, as the synergistic increase in DEG expression may contribute to CD8^+^ T-cell exhaustion.

### Result 4: Cellular Communication Based on DNB Genes Contributes to T Cell Exhaustion

**Figure 5:**
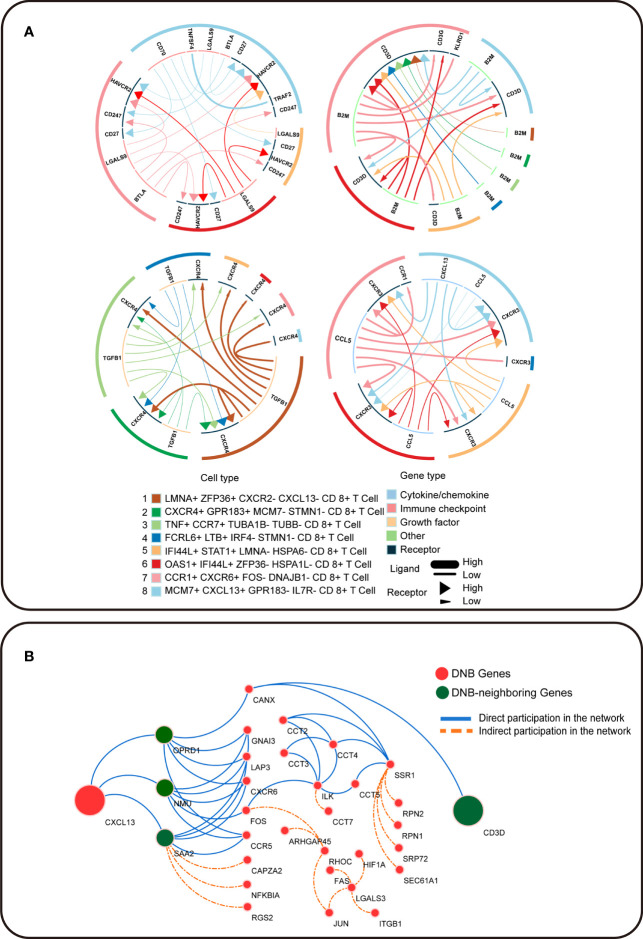
Intercellular communication in T cell exhaustion. **(A)** The receptor–ligand pairs identified by iTALK. **(B)** Cytoscape visualization of the protein–protein interaction network in CRC. Red circles represent DNB genes, and green circles represent DNB-neighboring genes. The solid blue line represents genes that directly participated in the network, and the orange dotted line represents genes that indirectly participated in the network.

To further understand the pre-exhausted subpopulation of CD8^+^ T cells, we analyzed the receptor–ligand interactions between different cell subpopulations, which may change the cancer-immune microenvironment associated with exhausted differentiation of CD8^+^ T cells (**Figure 5A**). Among the interactions in the *OAS1* ^+^ *IFI44L* ^+^ *ZFP36* ^-^ *HSPA1L* ^-^ CD8^+^ T cell subpopulation, the *LGALS9* gene in the immune checkpoint interacted with the exhaustion of the terminal exhausted CD8^+^ T cell subpopulations: *CCR1* ^+^ *CXCR6* ^+^ *FOS* ^−^ *DNAJB1* ^−^ CD8^+^ T cells and *MCM7* ^+^ *CXCL13* ^+^ *GPR183* ^-^ *IL7R* ^−^ CD8^+^ T cells, which has the receptor encoded by *HAVCR2*. *HAVCR2* is an important biomarker during T cell exhaustion, and the protein encoded by this gene belongs to the *TIM* immunoglobulin superfamily. The activation of *TIM*-related signals pathway significantly inhibits T cell killing efficiency against colon cancer cells. Tim-3 signaling decreased the expression of perforin and granzyme B in T cells, which reduced the cytotoxicity of T cells against colon cancer cells. We observed that *CXCL13* is a dynamic network marker in the *MCM*7^+^ *CXCL13* ^+^ *GPR183* ^−^ *IL7R* ^−^ CD8^+^ T-cell population and a marker gene for terminal exhausted T cell. Receptor *CXCR3* interacted with *CXCL13* in the pre-exhausted T cell subpopulation. In this study, *CXCL13* was the marker gene for terminally exhausted CD8^+^ T cell subpopulations, and *CD3D* was a DNB-neighboring gene. DNB genes interact with their DNB-neighbor genes and may provide feedback into the DNB gene population to affect cellular communication (**Figure 5B**). In summary, the expression of CD8^+^ T cell exhausted differentiation-associated genes is mediated by complex gene network changes.

## Discussion

Deciphering the process of T cell exhaustion is a significant challenge for immunotherapy; however, scRNA-seq is an efficient tool for characterizing the cell subpopulation in CRC. This study provided the T cell exhaustion trajectory. We used DNB to identify biomarkers for pre-exhausted CD8^+^ T cells in the critical exhaustion period. We found receptor–ligand interactions of terminally differentiated exhausted T cells with pre-exhausted T cells at the cellular communication level.

However, this study has several limitations. The pre-exhausted T cell selected for the critical time had only 160, but the cell number could meet our analysis requirement (n > 6). More evidence is warranted through further specific knockout experiments and animal models to confirm the exact mechanism of how *CCT6A* drives pre-exhausted T cells to exhaustion.

The differentiation trajectory toward exhaustion provided a different cell population for cellular targeting in immune research, rather than terminal Tex cells currently used in immunotherapy (4). A key feature of Tex cells is the increased and sustained expression of multiple inhibitory receptors (IRS) (5, 6, 22). According to the expression of multiple functional genes, the inhibitory receptors of pre-exhausted cells have not been highly expressed, the function of cytokines and effectors has begun to decline, and cell proliferation and apoptosis have just begun to appear disorder. Moreover, the expression of *IL2, IL10, IL12, LAYN* in pre-exhausted T cells were consistent with the expression of genes in other cancer (23), suggesting that pre-exhausted T cell have common features in immune microenvironment (11).

We identified the pre-exhausted T cell subpopulation during critical exhaustion by DNB method. Compared with the traditional method based on the differential expression of molecular biomarkers in a static manner to detect molecular changes in cell subpopulations (24), DNB is superior in identifying exhausted pathological differentiation of CD8^+^ T cells in CRC. According to the DNB ranking, *CCT6A* in the CD8^+^ T cell subpopulation were pre-exhausted biomarkers. *CCT6A* was strongly correlated with survival of CRC patients, which is consisted with that *CCT6A* may account for the survival of non-small cell lung cancer (NSCLC) patients (25). Pre-exhausted T cells emerged as a critical transitional period, wherein the two groups of DNB-neighboring genes flipped their expression patterns. DNB-neighboring genes showed expression turnover after the exhausted critical period. Those genes were enriched in *JAK-STAT* signaling pathway, *PD-L1* expression and *PD-1* checkpoint pathway in cancer. In addition, *JAK/STAT* signaling pathway is critical for the response of T cells to cytokines (26). Besides, co-blockade of the *LAG-3* and *PD-1* pathways in chronic LCMV exhibited robust and synergistic reversal of T cell exhaustion, with similar results in tumor systems and other infection models (6). We assumed that combining *PD-1* blockade with multi-targeted immunotherapy may enhance the reversal of T cell exhaustion effects. DNB-neighboring reverse genes in pre-exhausted CD8^+^ T cell that may provide clues to reversing exhaustion and improving the efficacy of immune response. The reversal of T cell exhaustion requires further investigation.

In the DNB core genes interaction network, CCT6A interacted with *TUBA1B*. CCT plays an important role in the correct synthesis and assembly of tubulin proteins (27). Tubulin was coded by *TUBA1B* (tubulin alpha 1b), the principal constituent of microtubules. Furthermore, *CCT6A* is associated with T cell reduction in cytotoxicity, immune function loss, and reduced T cell cytotoxicity during exhaustion with prolonged exposure to tumor antigens (28). In our Reaction pathway analysis, *CCT6A* played a role in forming tubulin folding intermediates *via* CCT/TriC formation of tubulin folding intermediates by CCT/TriC. During the process of CD8^+^ T cell normal differentiation, CD8^+^ T cells can activate and proliferate into Cytotoxic T lymphocyte (CTL). Besides, once the T cell receptor (TCR) of CTL specifically binds to MHC on the surface of target cells, the TCR and co-receptors accumulate towards the site of effector-target cell contact and lead to CTL polarization (29). The cytoskeletal system, including actin and tubulin, is rearranged and redistributed towards the contact site, thus ensuring that the non-specific effector molecules stored in the CTL are secreted and directed to the contacted target cells (30). However, the transcriptional profiles during T cell exhaustion were abnormal. *CCT6A*, as the DNB core genes, drives *TUBA1B* at the exhaustion critical state, may have an impact on tubulin folding, suggesting that tubulin of the cytoskeletal system of T cells prior to exhaustion may not ensure normal polarization of CD8^+^ CTL cells. The mechanism of *CCT6A* from these studies combined with DNB core gene expression patterns in ours suggested that *CCT6A* as the core gene have the potential to be an immunotherapy target for pre-exhausted cell.

We found that our dynamic network marker *CXCL13* is involved in signaling during T cell exhaustion. Unlike studies that focus on the communication of other cell types to CD8^+^ T cells, we focused on the interactions between CD8^+^ T cell subtypes during exhaustion. In addition, we observed that *CXCL13*, as the ligand of terminal Tex cells, interacted with *CXCR3* as the receptor of pre-exhausted T cells. *CXCR3*, as a co-receptor for the chemokine *CXCL13*, is chemotactic and involved in regulating the differentiation and development of memory cells and effector T cells (31). This indicates that cellular communication of terminal Tex cells with pre-exhausted T cells might lead to differentiation toward exhaustion. Considering that various factors mediate T cell exhaustion, the cellular communication of various CD8^+^ T cell subtypes during CD8^+^ T cell exhaustion may provide new insights into T cell exhaustion mechanisms.

In conclusion, we showed the CD8^+^ T cell exhaustion trajectories and characterized the pre-exhausted T cells from tumor tissues of CRC patients at the single-cell level using DNB. Both cellular communication and DNB-neighboring genes expression were actuated by DNB core genes that contribute to T cell exhaustion. The DNB core gene *CCT6A* provides a new clue for potential immunotherapy targets.

## Data Availability Statement

Publicly available data sets were analyzed in this study. This data can be found here: GSE108989: https://www.ncbi.nlm.nih.gov/bioproject/?term=GSE108989; TCGA: https://portal.gdc.cancer.gov/repository.

## Ethics Statement

The studies involving human participants were reviewed and approved by the Research and Ethical Committee of Peking University People’s Hospital. The patients/participants provided their written informed consent to participate in this study.

## Author Contributions

JH and CH carried out the primary literature search. JH and JZ performed the data analysis. JH, WL and FL drafted the manuscript. JH, CH, JZ, HL, RL, and PC performed the literature search and revised the manuscript. All authors contributed to the article and approved the submitted version.

## Funding

This work was supported by the National Natural Science Foundation of China (Grant Nos. 11771152, 11901203, 11971176, and 12026608), Guangdong Basic and Applied Basic Research Foundation (Grant Nos. 2019B151502062, 2021A1515012317).

## Conflict of Interest

The authors declare that the research was conducted in the absence of any commercial or financial relationships that could be construed as a potential conflict of interest.

## Publisher’s Note

All claims expressed in this article are solely those of the authors and do not necessarily represent those of their affiliated organizations, or those of the publisher, the editors and the reviewers. Any product that may be evaluated in this article, or claim that may be made by its manufacturer, is not guaranteed or endorsed by the publisher.

## Supplementary Material

Supplementary Figure 1 Genes expression of differentiation profiles in CD8^+^ T cells. **(A)** t-SNE clustering of CD8+ T cells in tumor tissue (n = 1646) from scRNA-seq of CRC patients showing 8 clusters (n =8). **(B)** Violin plot indicating the expression distribution of *HAVCR2, CXCL13, PDCD1, FOS, SELL and LAYN in* NTC, PTC and TTC. **(C)** The expression levels of *CCT6A, SRSF1, EIF4A3* and *IFI44L* in different pseudotime of CD8^+^ T cell subpopulation. The x-axis represents pseudotime, and the y-axis represents gene expression. **(D)** Five heatmaps showing the dynamic expression changes of genes, including the function of T cell cytokine production, T cell apoptotic process, inhibitory receptors, cell proliferation and T cell effector function.

Supplementary Figure 2 GO enrichment results of DNB genes. **(A)** Pie chart representing the proportion of the top GO terms are ranked by the GO enrichment. **(B)** Shown is a Cytoscape visualization of the top GO terms interaction network. **(C)** Histogram showing the top GO terms ranked by GO enrichment.

Supplementary Figure 3 DNB genes drive the flipped expression of DEGs. **(A)** Pie chart representing the proportion of the top pathways of KEGG ranked by the KEGG enrichment. **(B)** The network plot shows the DNB genes interaction with DNB-neighboring DEG, including three T cell exhaustion periods. The network nodes from the purple region are DNB genes. The network nodes from the dark green region are DNB-neighboring-DEGs. The genes and their location in the network are the same for all three different exhaustion periods, and low to high gene expression is indicated by a gradation from green to red. **(C)** Gene expression heatmap showing gene expression of the CCT family and *TUBA1B* at different cancer stages and sources in TCGA COREAD dataset, and low to high gene expression is indicated by a gradation from blue to red. **(D)** Correlation of *CCT6A* and *TUBA1B* in TCGA COREAD dataset. The correlation coefficient (R) and significance of the correlation coefficient (P) were calculated.

## Abbreviations

CRC, colorectal cancer; DNB, dynamic network biomarker; COAD, colon adenocarcinoma; TCGA, The Cancer Genome Atlas; Tex cell, exhausted T cell; GO, gene ontology; KEGG, Kyoto Encyclopedia of Genes and Genomes; READ, rectum adenocarcinoma; SCE, single-cell entropy; DEG, differentially expressed gene; IRS, inhibitory receptors; GSVA, gene set variation analysis; NSCLC, non-small cell lung cancer; PTC, CD8^+^ T cells from peripheral blood; NTC, CD8^+^ T cells from adjacent normal colorectal tissues; TTC, CD8^+^ T cells from tumor; TCR, T-cell receptor; CTL, cytotoxic T lymphocyte.
