# Supplementary figures and images for: Dynamic Network Biomarker of Pre-Exhausted CD8+ T Cells Contributed to T Cell Exhaustion in Colorectal Cancer
Source: Front Immunol. 2021 Aug 9;12:691142. doi: 10.3389/fimmu.2021.691142 (PMC8381053; doi:10.3389/fimmu.2021.691142)

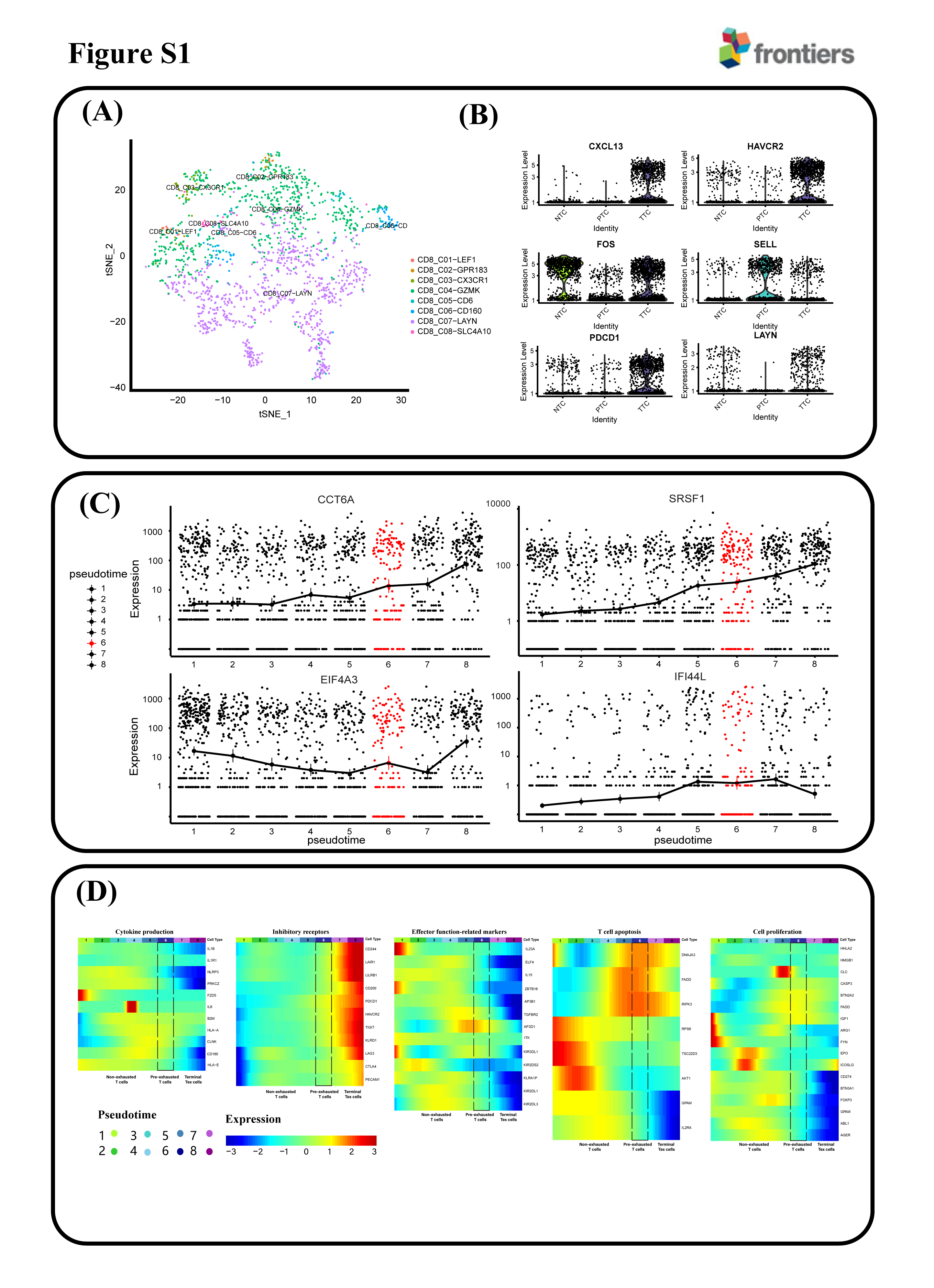

Supplement: Supplementary Figure 1 — Genes expression of differentiation profiles in CD8+ T cells. (A) t-SNE clustering of CD8+ T cells in tumor tissue (n = 1646) from scRNA-seq of CRC patients showing 8 clusters (n =8). (B) Violin plot indicating the expression distribution of HAVCR2, CXCL13, PDCD1, FOS, SELL and LAYN in NTC, PTC and TTC. (C) The expression levels of CCT6A, SRSF1, EIF4A3 and IFI44L in different pseudotime of CD8+ T cell subpopulation. The x-axis represents pseudotime, and the y-axis represents gene expression. (D) Five heatmaps showing the dynamic expression changes of genes, including the function of T cell cytokine production, T cell apoptotic process, inhibitory receptors, cell proliferation and T cell effector function. [file Image_1.tif]

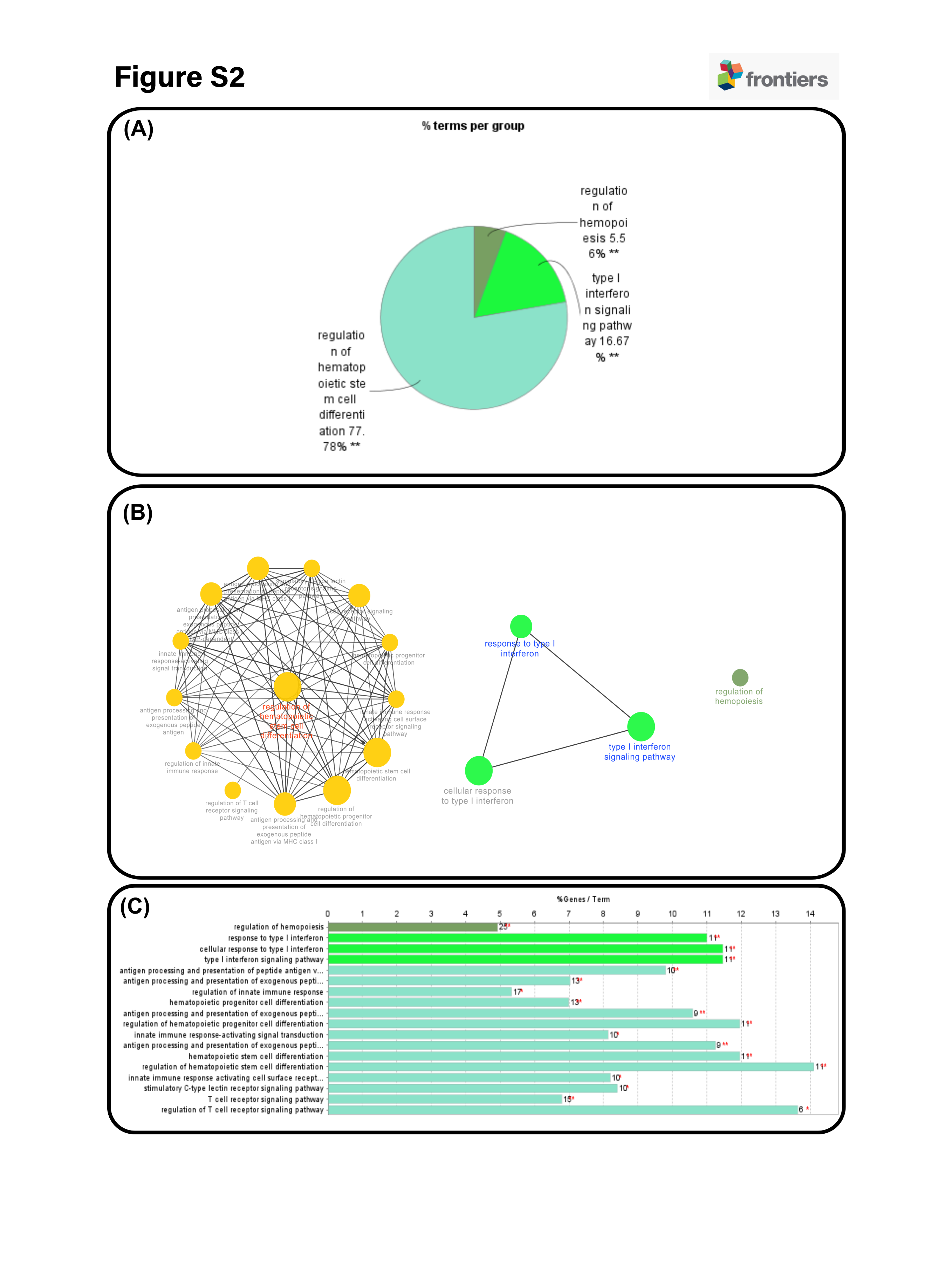

Supplement: Supplementary Figure 2 — GO enrichment results of DNB genes. (A) Pie chart representing the proportion of the top GO terms are ranked by the GO enrichment. (B) Shown is a Cytoscape visualization of the top GO terms interaction network. (C) Histogram showing the top GO terms ranked by GO enrichment. [file Image_2.tif]

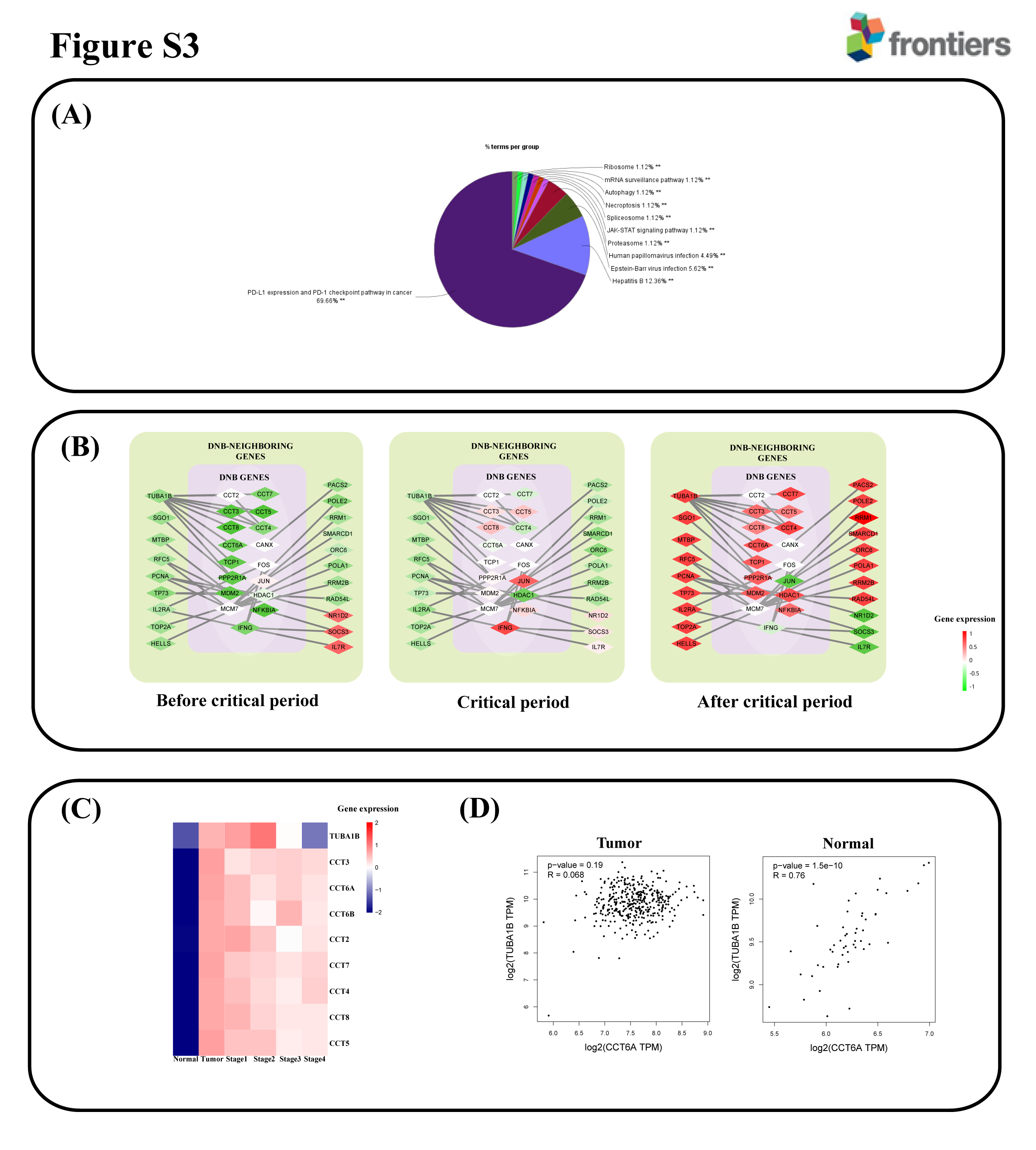

Supplement: Supplementary Figure 3 — DNB genes drive the flipped expression of DEGs. (A) Pie chart representing the proportion of the top pathways of KEGG ranked by the KEGG enrichment. (B) The network plot shows the DNB genes interaction with DNB-neighboring DEG, including three T cell exhaustion periods. The network nodes from the purple region are DNB genes. The network nodes from the dark green region are DNB-neighboring-DEGs. The genes and their location in the network are the same for all three different exhaustion periods, and low to high gene expression is indicated by a gradation from green to red. (C) Gene expression heatmap showing gene expression of the CCT family and TUBA1B at different cancer stages and sources in TCGA COREAD dataset, and low to high gene expression is indicated by a gradation from blue to red. (D) Correlation of CCT6A and TUBA1B in TCGA COREAD dataset. The correlation coefficient (R) and significance of the correlation coefficient (P) were calculated. [file Image_3.tif]
