# Supplementary material for: Dynamic Network Biomarker of Pre-Exhausted CD8+ T Cells Contributed to T Cell Exhaustion in Colorectal Cancer
Source: Front Immunol. 2021 Aug 9;12:691142. doi: 10.3389/fimmu.2021.691142 (PMC8381053; doi:10.3389/fimmu.2021.691142)
Supplement: Supplementary file 4 [file Table_1.docx]

| **CRC Tumor Tissue DEG** | | | | **CRC Peripheral blood DEG** | | | | **CRC NormalTissue DEG** | | | |
| --- | --- | --- | --- | --- | --- | --- | --- | --- | --- | --- | --- |
| GeneSymbol | p_val | logFC | p_val_adj | GeneSymbol | p_val | logFC | p_val_adj | GeneSymbol | p_val | logFC | p_val_adj |
| CXCL13 | 2.88E-247 | 2.363018382 | 5.86E-243 | CX3CR1 | 0 | 1.669265185 | 0 | GPR15 | 1.06E-274 | 1.018379901 | 2.16E-270 |
| HAVCR2 | 2.34E-207 | 1.502368915 | 4.76E-203 | S1PR1 | 0 | 0.989386268 | 0 | FOS | 7.58E-197 | 1.526767452 | 1.54E-192 |
| CD82 | 9.19E-196 | 1.152245572 | 1.87E-191 | ITGA1 | 0 | -1.691191448 | 0 | NR4A3 | 6.57E-189 | 1.099462367 | 1.34E-184 |
| RPL3 | 1.04E-185 | -0.640287696 | 2.11E-181 | RGS1 | 0 | -3.00959202 | 0 | FOSB | 7.78E-188 | 1.134727984 | 1.58E-183 |
| VCAM1 | 4.11E-183 | 1.082638693 | 8.35E-179 | TNFAIP3 | 5.36E-288 | -2.362310588 | 1.09E-283 | NR4A2 | 1.55E-182 | 1.346559352 | 3.16E-178 |
| HLA-DRA | 2.39E-170 | 1.189237686 | 4.86E-166 | SAMSN1 | 2.83E-268 | -1.583432127 | 5.76E-264 | ZFP36 | 1.08E-172 | 1.026689468 | 2.19E-168 |
| DUSP4 | 6.44E-160 | 1.016227425 | 1.31E-155 | RIPOR2 | 6.38E-259 | 1.231763047 | 1.30E-254 | CD160 | 9.67E-167 | 1.113338495 | 1.97E-162 |
| RBPJ | 9.74E-158 | 1.135682877 | 1.98E-153 | ITGAE | 8.60E-259 | -1.785106441 | 1.75E-254 | CD69 | 1.08E-161 | 1.299674469 | 2.19E-157 |
| MYO7A | 3.24E-147 | 0.533408565 | 6.58E-143 | PLAC8 | 4.30E-250 | 0.989615087 | 8.73E-246 | TMIGD2 | 2.30E-147 | 0.769747024 | 4.67E-143 |
| TNFSF4 | 3.48E-138 | 0.8625808 | 7.06E-134 | TXNIP | 1.12E-245 | 1.056733923 | 2.27E-241 | ZNF331 | 2.05E-133 | 1.137860508 | 4.17E-129 |
| HLA-DRB1 | 1.89E-135 | 0.885253177 | 3.84E-131 | FGFBP2 | 6.58E-235 | 1.483235233 | 1.34E-230 | BTG2 | 1.01E-127 | 1.04320055 | 2.05E-123 |
| HLA-DRB6 | 2.57E-131 | 0.770827181 | 5.23E-127 | SRGN | 3.55E-231 | -1.029150501 | 7.22E-227 | PTGER4 | 1.53E-127 | 1.083967966 | 3.11E-123 |
| PDCD1 | 1.93E-130 | 0.920911486 | 3.91E-126 | LINC00861 | 4.06E-223 | 0.945132179 | 8.25E-219 | ITGB2 | 5.68E-125 | -0.816644465 | 1.15E-120 |
| HLA-DRB5 | 2.90E-128 | 0.778311216 | 5.89E-124 | ITM2C | 9.77E-219 | -1.357733889 | 1.98E-214 | SPRY1 | 4.76E-122 | 0.622692665 | 9.68E-118 |
| OASL | 3.51E-122 | 0.771393355 | 7.13E-118 | CREM | 3.78E-217 | -1.979932574 | 7.68E-213 | ITGA1 | 1.51E-107 | 0.854882275 | 3.06E-103 |
| SAMSN1 | 2.67E-117 | 0.725058284 | 5.42E-113 | NR4A2 | 4.33E-216 | -2.446265571 | 8.80E-212 | NR4A1 | 1.67E-105 | 0.843431216 | 3.39E-101 |
| APOBEC3G | 5.31E-115 | 0.829577147 | 1.08E-110 | FOSB | 1.12E-212 | -1.492798852 | 2.28E-208 | CSRNP1 | 4.15E-104 | 0.623568234 | 8.44E-100 |
| NDFIP2 | 9.90E-111 | 0.800900897 | 2.01E-106 | S1PR5 | 7.58E-211 | 0.970818456 | 1.54E-206 | TNFAIP3 | 1.39E-100 | 0.834041389 | 2.82E-96 |
| PLAC8 | 2.73E-110 | -0.698676537 | 5.55E-106 | RGS2 | 8.47E-209 | -1.998028116 | 1.72E-204 | GZMH | 4.47E-100 | -1.054861462 | 9.09E-96 |
| RPS6 | 1.44E-109 | -0.500804413 | 2.93E-105 | DUSP4 | 4.21E-207 | -1.540227182 | 8.55E-203 | SIK1 | 2.57E-98 | 0.387354959 | 5.22E-94 |
| RPS3 | 4.68E-109 | -0.459981075 | 9.52E-105 | CXCR4 | 1.13E-203 | -1.720906999 | 2.31E-199 | SMIM3 | 8.76E-98 | 0.749747477 | 1.78E-93 |
| RPL13 | 5.40E-108 | -0.477820345 | 1.10E-103 | PXN | 9.37E-193 | 0.910606524 | 1.90E-188 | PER1 | 1.37E-97 | 0.485017107 | 2.79E-93 |
| MIR155HG | 1.20E-106 | 0.609708769 | 2.45E-102 | PELO | 4.50E-186 | -0.762340583 | 9.15E-182 | YPEL5 | 9.85E-97 | 0.82436558 | 2.00E-92 |
| GZMB | 2.00E-106 | 0.899090457 | 4.07E-102 | PLEK | 2.65E-180 | 1.374913856 | 5.39E-176 | ITGB1 | 1.45E-95 | -0.914655801 | 2.95E-91 |
| CTLA4 | 1.78E-103 | 0.749946907 | 3.62E-99 | ZFP36 | 2.94E-177 | -1.726815764 | 5.97E-173 | JUNB | 4.94E-94 | 1.014075316 | 1.00E-89 |
| CD74 | 4.25E-102 | 0.679650857 | 8.64E-98 | CD69 | 6.24E-173 | -1.763247828 | 1.27E-168 | IL12RB2 | 1.57E-93 | 0.549447372 | 3.19E-89 |
| PHLDA1 | 3.87E-100 | 0.756989781 | 7.87E-96 | FCGR3A | 7.07E-172 | 1.413275073 | 1.44E-167 | FOSL2 | 6.57E-93 | 0.710047418 | 1.33E-88 |
| CCL3 | 4.66E-100 | 0.844999929 | 9.47E-96 | SLC7A5 | 1.18E-168 | -0.780413266 | 2.39E-164 | FAM46C | 3.63E-91 | 0.702379325 | 7.37E-87 |
| CX3CR1 | 1.32E-98 | -1.141522088 | 2.69E-94 | RPL3 | 1.53E-167 | 0.639188013 | 3.12E-163 | ANKRD28 | 5.06E-91 | 0.738010654 | 1.03E-86 |
| TXNIP | 1.52E-98 | -0.756962433 | 3.09E-94 | PHLDA1 | 2.42E-162 | -1.185338658 | 4.92E-158 | TIPARP | 1.89E-89 | 0.7681625 | 3.84E-85 |
| HLA-DQB1 | 9.62E-98 | 0.653770809 | 1.96E-93 | GIMAP7 | 6.85E-162 | 0.802853697 | 1.39E-157 | HLA-DPB1 | 2.64E-89 | -0.646895294 | 5.37E-85 |
| BHLHE40 | 1.76E-97 | 0.73540426 | 3.57E-93 | FGR | 1.10E-159 | 1.087486551 | 2.24E-155 | SAMD3 | 3.03E-89 | -0.743799391 | 6.15E-85 |
| CD160 | 1.82E-94 | -0.923602195 | 3.70E-90 | SKIL | 4.99E-159 | -1.118825737 | 1.01E-154 | PFKFB3 | 8.82E-86 | 0.787629093 | 1.79E-81 |
| LYST | 6.42E-94 | 0.650051714 | 1.31E-89 | CCL4 | 1.20E-158 | -1.933882744 | 2.44E-154 | SYTL3 | 1.33E-85 | 0.703310948 | 2.69E-81 |
| BHLHE40-AS1 | 2.37E-93 | 0.329580297 | 4.82E-89 | PFKFB3 | 2.59E-154 | -1.200876509 | 5.26E-150 | RANBP2 | 1.59E-85 | 0.784698303 | 3.24E-81 |
| HLA-DQA1 | 1.67E-92 | 0.7208488 | 3.39E-88 | FOS | 1.26E-149 | -2.698895383 | 2.56E-145 | RASGEF1B | 1.10E-84 | 0.583001216 | 2.23E-80 |
| EEF1G | 3.67E-92 | -0.454299 | 7.46E-88 | ADD3 | 2.57E-149 | 1.001038475 | 5.23E-145 | APOBEC3G | 4.64E-83 | -0.871016032 | 9.42E-79 |
| LGALS3 | 1.19E-91 | 0.585082097 | 2.41E-87 | IFNG | 4.49E-149 | -1.782500656 | 9.13E-145 | MAP3K8 | 5.19E-83 | 0.851521133 | 1.05E-78 |
| APOBEC3C | 2.04E-91 | 0.69419742 | 4.15E-87 | KLF2 | 1.35E-147 | 0.508746744 | 2.74E-143 | RGS1 | 1.14E-82 | 0.632709717 | 2.32E-78 |
| IFNG | 6.95E-91 | 0.522875663 | 1.41E-86 | PDE4B | 3.40E-145 | -1.055643128 | 6.92E-141 | MYADM | 2.48E-82 | 0.694235334 | 5.04E-78 |
| SEMA4A | 6.98E-91 | 0.550351946 | 1.42E-86 | CAPG | 6.09E-145 | -1.179655866 | 1.24E-140 | PRMT9 | 1.17E-81 | 0.431376923 | 2.37E-77 |
| RPS14 | 2.89E-89 | -0.576899014 | 5.88E-85 | A2M | 3.99E-144 | 0.757983714 | 8.11E-140 | CAPG | 8.74E-81 | 0.631502309 | 1.78E-76 |
| ITM2C | 3.00E-89 | 0.620868731 | 6.10E-85 | NR4A1 | 3.72E-142 | -1.423246113 | 7.56E-138 | PPP1R15A | 3.22E-80 | 0.676066531 | 6.54E-76 |
| RGS1 | 4.15E-89 | 0.495658112 | 8.43E-85 | DNAJA1 | 6.20E-142 | -1.322406619 | 1.26E-137 | S1PR1 | 2.87E-79 | -0.63883711 | 5.84E-75 |
| CPNE7 | 4.81E-87 | 0.395025885 | 9.77E-83 | NR4A3 | 1.25E-141 | -1.08529449 | 2.54E-137 | PABPC1 | 5.81E-79 | 0.534583427 | 1.18E-74 |
| RPLP2 | 7.81E-87 | -0.478298894 | 1.59E-82 | PPP1R15A | 1.68E-141 | -1.267096932 | 3.42E-137 | B3GNT7 | 6.40E-79 | 0.450017657 | 1.30E-74 |
| ITGAE | 8.06E-87 | 0.590819792 | 1.64E-82 | ZNF331 | 2.21E-140 | -1.811076469 | 4.50E-136 | DUSP1 | 2.40E-78 | 0.7934 | 4.88E-74 |
| CXCR6 | 1.08E-86 | 0.89176737 | 2.19E-82 | KLRG1 | 3.29E-139 | 1.059385568 | 6.68E-135 | KLRC1 | 3.76E-78 | 0.9031254 | 7.64E-74 |
| AFAP1L2 | 1.65E-86 | 0.56062569 | 3.36E-82 | RPS14 | 8.36E-138 | 0.717608703 | 1.70E-133 | ASB2 | 5.96E-78 | 0.619558883 | 1.21E-73 |
| RPS18 | 1.67E-86 | -0.467286382 | 3.39E-82 | RPS3 | 5.02E-137 | 0.553583909 | 1.02E-132 | HLA-DPA1 | 1.43E-77 | -0.655828279 | 2.91E-73 |
| TBCD | 2.54E-85 | 0.632994511 | 5.15E-81 | PDE4D | 6.15E-136 | -0.891270003 | 1.25E-131 | HLA-DRA | 1.58E-76 | -1.091438188 | 3.22E-72 |
| NAB1 | 3.40E-85 | 0.52948108 | 6.92E-81 | JUN | 1.52E-134 | -1.257250067 | 3.10E-130 | CREM | 2.02E-76 | 0.588055402 | 4.11E-72 |
| HSPA1B | 1.12E-83 | 1.295114865 | 2.28E-79 | EGR1 | 5.26E-134 | -0.773690838 | 1.07E-129 | RBKS | 4.35E-76 | 0.281092914 | 8.84E-72 |
| GAPDH | 3.35E-83 | 0.543590092 | 6.81E-79 | IVNS1ABP | 3.74E-133 | -1.007991345 | 7.60E-129 | JUND | 3.85E-75 | 0.278431358 | 7.82E-71 |
| PDE4DIP | 1.81E-82 | 0.634301619 | 3.68E-78 | BIN2 | 4.68E-133 | 0.838864692 | 9.51E-129 | HLA-DRB5 | 1.14E-74 | -0.717487342 | 2.33E-70 |
| LDHA | 1.08E-81 | 0.612662349 | 2.19E-77 | STK17B | 1.04E-132 | -0.784022574 | 2.11E-128 | LDLRAD4 | 1.85E-74 | 0.803430845 | 3.77E-70 |
| SLC27A2 | 1.86E-80 | 0.493455167 | 3.78E-76 | RGCC | 2.59E-131 | -1.095678878 | 5.26E-127 | MCL1 | 2.98E-74 | 0.67611964 | 6.05E-70 |
| S1PR1 | 3.26E-80 | -0.535157572 | 6.63E-76 | LDLRAD4 | 3.33E-131 | -1.001196584 | 6.76E-127 | HLA-DRB6 | 4.63E-73 | -0.672290832 | 9.42E-69 |
| RPL5 | 2.12E-79 | -0.485043585 | 4.32E-75 | CXCR6 | 2.26E-129 | -1.465451685 | 4.60E-125 | CXCL13 | 6.63E-73 | -1.884970292 | 1.35E-68 |
| FTL | 1.69E-78 | -0.355484568 | 3.44E-74 | CCL4L1 | 3.74E-129 | -1.181469618 | 7.60E-125 | EIF1 | 7.01E-73 | 0.426280444 | 1.42E-68 |
| HNRNPLL | 4.88E-78 | 0.465592737 | 9.92E-74 | TRAF3IP3 | 5.03E-129 | 0.742207741 | 1.02E-124 | GFPT2 | 7.91E-71 | 0.618942726 | 1.61E-66 |
| PTGER2 | 1.23E-77 | -0.606615376 | 2.51E-73 | EMP3 | 2.75E-128 | 0.508908554 | 5.59E-124 | PCED1B | 2.36E-70 | -0.395556289 | 4.80E-66 |
| TNFRSF9 | 3.84E-77 | 0.730207816 | 7.80E-73 | RPS6 | 9.17E-128 | 0.592898433 | 1.86E-123 | THBS1 | 1.16E-69 | 0.515227919 | 2.36E-65 |
| DUSP10 | 7.59E-76 | 0.645462956 | 1.54E-71 | MCL1 | 9.30E-127 | -0.9537825 | 1.89E-122 | HLA-DRB1 | 4.44E-68 | -0.735716893 | 9.03E-64 |
| ADAM19 | 1.43E-75 | 0.594297821 | 2.90E-71 | RASGRP2 | 1.60E-125 | 0.498540594 | 3.25E-121 | PDE4A | 8.40E-65 | 0.418583425 | 1.71E-60 |
| HLA-DPA1 | 1.53E-75 | 0.543417881 | 3.11E-71 | ITGB2 | 2.85E-125 | 0.74350235 | 5.80E-121 | KLRC2 | 3.58E-64 | 0.926445665 | 7.27E-60 |
| ZNF683 | 2.33E-75 | 0.758267575 | 4.74E-71 | DUSP10 | 2.75E-124 | -0.977940197 | 5.59E-120 | RGS2 | 3.74E-64 | 0.52394006 | 7.59E-60 |
| SARDH | 2.87E-74 | 0.337397529 | 5.84E-70 | PTPN22 | 3.79E-124 | -0.831729275 | 7.70E-120 | REL | 6.33E-64 | 0.627017407 | 1.29E-59 |
| SLC7A5 | 4.28E-74 | 0.357886947 | 8.71E-70 | RAP1GAP2 | 4.60E-124 | 0.539544091 | 9.34E-120 | LOC284454 | 1.02E-62 | 0.423697976 | 2.08E-58 |
| HLA-DMA | 7.17E-74 | 0.56782182 | 1.46E-69 | CSRNP1 | 7.41E-124 | -0.962362493 | 1.51E-119 | CD74 | 1.22E-62 | -0.62808859 | 2.47E-58 |
| FGFBP2 | 4.09E-73 | -1.007428049 | 8.32E-69 | NFKBIA | 5.55E-123 | -1.271333934 | 1.13E-118 | AREG | 5.33E-62 | 0.255765318 | 1.08E-57 |
| RPL8 | 1.54E-71 | -0.389506523 | 3.13E-67 | YPEL5 | 9.64E-123 | -1.242828308 | 1.96E-118 | SRGN | 6.98E-61 | 0.444896615 | 1.42E-56 |
| RIPOR2 | 5.75E-71 | -0.775854591 | 1.17E-66 | FAM46C | 4.65E-122 | -1.115351064 | 9.44E-118 | C12orf75 | 8.36E-61 | -0.564025039 | 1.70E-56 |
| CXCR4 | 3.88E-70 | 0.602379377 | 7.88E-66 | GAPDH | 6.84E-122 | -0.754926876 | 1.39E-117 | UBASH3B | 1.35E-60 | 0.551455655 | 2.74E-56 |
| S1PR5 | 6.00E-69 | -0.592280193 | 1.22E-64 | GIMAP4 | 3.63E-121 | 0.768983581 | 7.39E-117 | EGR1 | 2.35E-60 | 0.361589077 | 4.77E-56 |
| CD38 | 6.11E-69 | 0.466479003 | 1.24E-64 | RASGEF1B | 1.69E-119 | -0.783698535 | 3.43E-115 | PCED1B-AS1 | 1.88E-59 | -0.4354583 | 3.81E-55 |
| COTL1 | 4.42E-68 | 0.538812303 | 8.97E-64 | DDIT4 | 3.39E-119 | -1.097806129 | 6.90E-115 | SRSF7 | 1.26E-58 | 0.492101118 | 2.56E-54 |
| RPL34 | 1.28E-67 | -0.465430916 | 2.61E-63 | PRSS23 | 5.67E-119 | 0.852297393 | 1.15E-114 | LOC100130476 | 4.28E-58 | 0.481590816 | 8.69E-54 |
| RPS12 | 2.83E-67 | -0.422189478 | 5.75E-63 | TGFBR3 | 9.92E-118 | 0.722539248 | 2.02E-113 | GPR65 | 1.15E-57 | 0.702513961 | 2.33E-53 |
| HLA-DMB | 3.74E-67 | 0.499691507 | 7.60E-63 | AES | 4.29E-117 | 0.775875959 | 8.72E-113 | PELO | 1.89E-57 | 0.41507883 | 3.85E-53 |
| GOLIM4 | 4.74E-66 | 0.305263561 | 9.63E-62 | TMSB10 | 6.61E-117 | 0.610482821 | 1.34E-112 | ADAM28 | 2.67E-57 | 0.55095521 | 5.43E-53 |
| ACP5 | 5.23E-66 | 0.592764511 | 1.06E-61 | SERTAD1 | 3.16E-116 | -0.873609129 | 6.42E-112 | CD55 | 8.99E-57 | 0.701496446 | 1.83E-52 |
| RPL13A | 1.19E-65 | -0.415909481 | 2.42E-61 | RPL13 | 1.41E-115 | 0.548401414 | 2.87E-111 | KDM6B | 1.14E-55 | 0.321749557 | 2.31E-51 |
| RASGRP2 | 2.92E-65 | -0.356335081 | 5.94E-61 | PER1 | 1.97E-113 | -0.565382481 | 4.00E-109 | MSH3 | 3.81E-55 | 0.525001137 | 7.74E-51 |
| GGA2 | 6.89E-64 | 0.488981392 | 1.40E-59 | SELL | 7.69E-113 | 1.46275334 | 1.56E-108 | JAML | 8.23E-55 | 0.564444518 | 1.67E-50 |
| RPL32 | 1.12E-63 | -0.426690426 | 2.28E-59 | RASA3 | 6.17E-112 | 0.71593547 | 1.25E-107 | EMP3 | 8.65E-55 | -0.321872825 | 1.76E-50 |
| SKIL | 1.82E-63 | 0.502239514 | 3.69E-59 | BTG2 | 7.81E-111 | -1.372125723 | 1.59E-106 | ZFP36L2 | 1.07E-54 | 0.500484565 | 2.18E-50 |
| RPL19 | 3.91E-63 | -0.392456586 | 7.94E-59 | ITGAM | 1.26E-110 | 0.584507118 | 2.55E-106 | NFKBIZ | 3.16E-54 | 0.776154705 | 6.43E-50 |
| SNORA33 | 5.18E-63 | -0.395716878 | 1.05E-58 | PTGER4 | 2.06E-110 | -1.165864136 | 4.18E-106 | TSPYL2 | 6.81E-54 | 0.825676052 | 1.38E-49 |
| NUSAP1 | 5.80E-63 | 0.436016086 | 1.18E-58 | IRF4 | 2.05E-108 | -0.942891052 | 4.16E-104 | IL18RAP | 1.13E-53 | 0.495420206 | 2.30E-49 |
| APOBEC3H | 8.30E-63 | 0.312739302 | 1.69E-58 | JUND | 3.03E-107 | -0.266335517 | 6.15E-103 | KLRG1 | 1.56E-52 | -0.769540537 | 3.17E-48 |
| SRGAP3 | 3.02E-62 | 0.359600226 | 6.14E-58 | DUSP2 | 6.94E-106 | -1.00880063 | 1.41E-101 | JMY | 1.62E-52 | 0.410485672 | 3.29E-48 |
| CCL4 | 3.38E-62 | 0.395498945 | 6.87E-58 | S100A4 | 7.82E-106 | 0.684011974 | 1.59E-101 | CCDC173 | 2.10E-52 | 0.387085134 | 4.28E-48 |
| IL7R | 3.61E-62 | -0.542030868 | 7.34E-58 | STK38 | 1.48E-105 | 0.741891007 | 3.00E-101 | PDCD4 | 2.20E-52 | 0.47821759 | 4.47E-48 |
| BTG3 | 6.30E-62 | 0.458161976 | 1.28E-57 | MYADM | 6.83E-105 | -0.950498027 | 1.39E-100 | TMSB10 | 2.50E-52 | -0.401976573 | 5.09E-48 |
| HMGB2 | 9.85E-61 | 0.542693662 | 2.00E-56 | RPL19 | 8.13E-105 | 0.535892846 | 1.65E-100 | LINC00861 | 1.36E-50 | -0.571840658 | 2.76E-46 |
| PXN | 3.20E-60 | -0.533652137 | 6.51E-56 | RPL13A | 1.50E-104 | 0.559723286 | 3.04E-100 | RIPOR2 | 1.99E-50 | -0.710849624 | 4.04E-46 |
| LAG3 | 1.26E-59 | 0.448873678 | 2.56E-55 | CYTIP | 2.95E-104 | -0.774650846 | 6.00E-100 | PTPN22 | 3.15E-50 | 0.508161995 | 6.41E-46 |
| MYO5B | 2.71E-59 | 0.332350942 | 5.50E-55 | SRSF7 | 3.38E-104 | -0.770994575 | 6.87E-100 | OASL | 4.12E-50 | -0.631190945 | 8.38E-46 |
| ITM2A | 7.83E-59 | 0.592612939 | 1.59E-54 | LOC284454 | 1.92E-103 | -0.67712838 | 3.89E-99 | EOMES | 6.98E-50 | -0.561827167 | 1.42E-45 |
| RPL4 | 4.58E-58 | -0.384164167 | 9.31E-54 | SORL1 | 1.26E-102 | 0.662185336 | 2.55E-98 | KLRC3 | 7.87E-50 | 0.605184474 | 1.60E-45 |
| GPR15 | 1.09E-57 | -0.469781303 | 2.22E-53 | NAMPT | 5.13E-102 | -0.747840162 | 1.04E-97 | CD7 | 2.23E-49 | 0.352310169 | 4.53E-45 |
| RNF19A | 2.54E-57 | 0.481747339 | 5.17E-53 | TNFRSF9 | 1.09E-101 | -1.115730999 | 2.21E-97 | CX3CR1 | 2.24E-49 | -1.018154504 | 4.56E-45 |
| ABCG1 | 3.47E-57 | 0.390484753 | 7.05E-53 | HSPA1B | 1.65E-101 | -1.438175762 | 3.35E-97 | KLF6 | 2.71E-49 | 0.607327894 | 5.51E-45 |
| CCR1 | 6.95E-57 | 0.46536143 | 1.41E-52 | JAML | 7.82E-101 | -0.784639904 | 1.59E-96 | SYNE1 | 5.40E-49 | -0.559085429 | 1.10E-44 |
| PTTG1 | 1.28E-56 | 0.305920278 | 2.60E-52 | SOCS1 | 1.02E-100 | -0.374173775 | 2.06E-96 | LINC-PINT | 6.95E-49 | 0.509397942 | 1.41E-44 |
| FUT8 | 1.51E-56 | 0.393183402 | 3.06E-52 | RPL14 | 1.32E-99 | 0.468733946 | 2.68E-95 | CD82 | 9.50E-49 | -0.747823665 | 1.93E-44 |
| FCRL6 | 3.95E-56 | -0.740502419 | 8.03E-52 | FCRL6 | 2.28E-99 | 0.97899105 | 4.64E-95 | BRE-AS1 | 1.15E-48 | 0.657736959 | 2.33E-44 |
| BIN2 | 9.06E-56 | -0.576880594 | 1.84E-51 | COTL1 | 7.29E-99 | -0.837061445 | 1.48E-94 | GNLY | 3.23E-48 | -0.818629476 | 6.56E-44 |
| RGCC | 2.17E-55 | 0.457207373 | 4.42E-51 | RPL34 | 1.09E-98 | 0.632511286 | 2.21E-94 | CD27 | 5.86E-48 | -0.618124187 | 1.19E-43 |
| RPS16 | 6.59E-55 | -0.318374193 | 1.34E-50 | SIK1 | 1.28E-98 | -0.425679732 | 2.60E-94 | HLA-DQA1 | 3.57E-47 | -0.556024594 | 7.25E-43 |
| CD44 | 1.60E-54 | 0.45461197 | 3.26E-50 | LDHA | 2.31E-98 | -0.851149136 | 4.70E-94 | ATP8B4 | 6.13E-47 | 0.440019114 | 1.25E-42 |
| LINC00861 | 1.76E-54 | -0.526728602 | 3.59E-50 | BIRC3 | 3.18E-98 | -0.941005013 | 6.46E-94 | CLDND1 | 1.97E-46 | 0.37657479 | 4.00E-42 |
| CENPF | 2.04E-54 | 0.348056142 | 4.14E-50 | PATL2 | 3.47E-98 | 1.042892992 | 7.05E-94 | HLA-DQA2 | 2.24E-46 | -0.252966989 | 4.56E-42 |
| ANXA5 | 2.41E-54 | 0.563823094 | 4.90E-50 | PZP | 2.49E-97 | 0.548643923 | 5.06E-93 | ARL4A | 3.81E-46 | 0.417788085 | 7.73E-42 |
| HSPA1L | 4.46E-54 | 1.137113619 | 9.07E-50 | HAVCR2 | 1.20E-96 | -1.386299311 | 2.44E-92 | PIK3R1 | 1.04E-45 | 0.670115254 | 2.11E-41 |
| RPS4X | 3.09E-53 | -0.345986098 | 6.28E-49 | TNIP3 | 1.34E-96 | -0.569734509 | 2.73E-92 | ADGRG5 | 1.51E-45 | -0.323916123 | 3.08E-41 |
| CSF1 | 3.95E-53 | 0.447299753 | 8.04E-49 | DUSP1 | 1.57E-96 | -1.333485921 | 3.19E-92 | GLRX | 1.25E-44 | -0.448095781 | 2.53E-40 |
| RPL14 | 6.60E-53 | -0.323098914 | 1.34E-48 | LINC-PINT | 2.48E-96 | -0.863709511 | 5.04E-92 | FAM53C | 2.27E-44 | 0.444385644 | 4.61E-40 |
| TANK | 1.37E-52 | 0.461134479 | 2.79E-48 | PPP1R16B | 2.47E-95 | -0.577235313 | 5.03E-91 | NKG7 | 4.78E-44 | -0.517287616 | 9.71E-40 |
| GIMAP1-GIMAP5 | 2.83E-52 | -0.490197078 | 5.74E-48 | EZR | 1.53E-93 | -0.770512851 | 3.12E-89 | XCL1 | 3.75E-43 | 0.416010412 | 7.61E-39 |
| PLSCR1 | 2.23E-51 | 0.444883821 | 4.52E-47 | LEF1 | 3.84E-93 | 0.961614313 | 7.81E-89 | GIMAP4 | 1.68E-42 | -0.590101065 | 3.42E-38 |
| RPL11 | 4.20E-51 | -0.370794234 | 8.54E-47 | CDKN1A | 8.71E-93 | -0.779665868 | 1.77E-88 | TSPO | 2.05E-42 | -0.360230035 | 4.17E-38 |
| HERPUD1 | 5.20E-51 | 0.450417671 | 1.06E-46 | BTG1 | 1.16E-91 | -0.76643964 | 2.37E-87 | APOBEC3C | 2.05E-42 | -0.54986946 | 4.18E-38 |
| IVNS1ABP | 5.21E-51 | 0.530913142 | 1.06E-46 | CD96 | 1.23E-91 | -0.675336916 | 2.51E-87 | VPS37B | 3.48E-42 | 0.392370222 | 7.07E-38 |
| SMC4 | 1.21E-50 | 0.450221205 | 2.47E-46 | ATP8B4 | 1.79E-91 | -0.593490093 | 3.65E-87 | CD27-AS1 | 6.34E-42 | -0.508887883 | 1.29E-37 |
| PAG1 | 1.36E-50 | 0.442416854 | 2.76E-46 | VCAM1 | 2.41E-90 | -0.996721092 | 4.90E-86 | ATF3 | 1.03E-41 | 0.432957584 | 2.09E-37 |
| RPL10 | 1.80E-50 | -0.257435783 | 3.65E-46 | CLK1 | 6.80E-90 | -0.808810946 | 1.38E-85 | SERTAD1 | 1.87E-41 | 0.334015906 | 3.79E-37 |
| GIMAP7 | 2.09E-50 | -0.481824879 | 4.25E-46 | REL | 4.48E-89 | -0.736707299 | 9.10E-85 | HLA-DQB1 | 3.14E-41 | -0.38450488 | 6.38E-37 |
| PLEK | 3.98E-50 | -0.870435992 | 8.08E-46 | RPS18 | 9.39E-89 | 0.522520554 | 1.91E-84 | HAVCR2 | 5.33E-41 | -0.950977397 | 1.08E-36 |
| JUN | 4.23E-50 | 0.317048169 | 8.60E-46 | IFITM1 | 1.74E-88 | 0.508921853 | 3.53E-84 | NELL2 | 7.92E-41 | -0.518172423 | 1.61E-36 |
| RAB37 | 1.70E-49 | -0.477642963 | 3.46E-45 | DNAJB1 | 2.97E-88 | -1.80893032 | 6.04E-84 | ABCB1 | 3.29E-40 | 0.388598548 | 6.69E-36 |
| CD27 | 2.93E-49 | 0.506679396 | 5.95E-45 | C1orf162 | 3.21E-88 | 0.633688638 | 6.52E-84 | RASA3 | 1.25E-39 | -0.450031406 | 2.53E-35 |
| TCF7 | 3.40E-49 | -0.485863848 | 6.90E-45 | SYNE1 | 5.12E-88 | 0.721672334 | 1.04E-83 | SCML4 | 1.85E-39 | 0.451026878 | 3.76E-35 |
| SRGN | 3.99E-49 | 0.255191412 | 8.11E-45 | LGALS3 | 1.20E-87 | -0.680641588 | 2.44E-83 | CLK1 | 2.03E-39 | 0.409965539 | 4.12E-35 |
| GIMAP5 | 6.48E-49 | -0.500107997 | 1.32E-44 | NDFIP2 | 5.61E-87 | -0.900331059 | 1.14E-82 | ID2 | 3.67E-39 | 0.478871807 | 7.45E-35 |
| SLA | 1.76E-48 | 0.500699748 | 3.57E-44 | JUNB | 5.63E-87 | -1.244932116 | 1.14E-82 | SH2D1A | 7.79E-39 | -0.315109067 | 1.58E-34 |
| PLP2 | 4.07E-48 | 0.363590782 | 8.27E-44 | HSP90AA1 | 7.96E-87 | -1.177579768 | 1.62E-82 | TNFSF4 | 1.13E-38 | -0.61107977 | 2.29E-34 |
| SLC5A3 | 4.10E-48 | 0.42723255 | 8.34E-44 | SAMD3 | 1.17E-86 | 0.710295189 | 2.38E-82 | CDC25B | 1.25E-38 | -0.343699093 | 2.55E-34 |
| ENTPD1 | 7.21E-48 | 0.626765855 | 1.47E-43 | CCL3 | 1.49E-86 | -1.243409939 | 3.04E-82 | RUNX2 | 1.65E-38 | -0.360975147 | 3.35E-34 |
| RPS23 | 7.29E-48 | -0.385299817 | 1.48E-43 | SLC7A5P2 | 1.60E-86 | -0.412477206 | 3.24E-82 | STK17B | 2.13E-38 | 0.364131794 | 4.33E-34 |
| RPS19 | 1.11E-47 | -0.345989448 | 2.26E-43 | MYL12A | 2.70E-86 | 0.412578719 | 5.49E-82 | FGFBP2 | 2.48E-38 | -0.844054546 | 5.05E-34 |
| RPL31 | 1.31E-47 | -0.454260975 | 2.67E-43 | JMJD6 | 4.59E-86 | -0.733027461 | 9.32E-82 | A2M | 2.51E-38 | -0.46518124 | 5.10E-34 |
| HSPH1 | 2.12E-47 | 1.069714024 | 4.31E-43 | KLRF1 | 5.11E-86 | 0.753146091 | 1.04E-81 | CDKN1A | 4.92E-38 | 0.326236503 | 1.00E-33 |
| RPL18 | 4.62E-47 | -0.361015877 | 9.40E-43 | RPL27A | 8.13E-86 | 0.422885961 | 1.65E-81 | FYN | 6.61E-38 | 0.384092826 | 1.34E-33 |
| SLF1 | 4.83E-47 | 0.311688683 | 9.82E-43 | RBPJ | 1.25E-85 | -0.96362182 | 2.55E-81 | SDCBP | 8.23E-38 | 0.615307382 | 1.67E-33 |
| AES | 6.35E-47 | -0.532758385 | 1.29E-42 | RPL5 | 1.57E-85 | 0.537653106 | 3.20E-81 | ADGRG1 | 2.03E-37 | -0.60676841 | 4.13E-33 |
| FCGR3A | 1.10E-46 | -0.875902765 | 2.24E-42 | EEF1G | 3.96E-85 | 0.514105158 | 8.04E-81 | ITGAE | 3.63E-37 | 0.350479493 | 7.38E-33 |
| CD27-AS1 | 1.99E-46 | 0.444052515 | 4.05E-42 | FOSL2 | 7.73E-85 | -0.790967955 | 1.57E-80 | PDE4DIP | 5.48E-37 | -0.462073982 | 1.11E-32 |
| RPS5 | 3.53E-46 | -0.414060651 | 7.17E-42 | GIMAP1-GIMAP5 | 1.88E-84 | 0.611886691 | 3.83E-80 | CD96 | 1.01E-36 | 0.390907926 | 2.05E-32 |
| TNFSF10 | 5.96E-46 | 0.371237845 | 1.21E-41 | CXCL13 | 2.44E-84 | -2.008307023 | 4.96E-80 | LIME1 | 6.34E-36 | -0.376296519 | 1.29E-31 |
| DDIT4 | 7.43E-46 | 0.46336455 | 1.51E-41 | RPL32 | 4.89E-84 | 0.566185481 | 9.93E-80 | CASP1 | 2.07E-35 | -0.387505026 | 4.21E-31 |
| TMEM123 | 1.31E-45 | -0.523649439 | 2.66E-41 | PDCD1 | 1.08E-83 | -0.916748767 | 2.19E-79 | PLEK | 3.25E-35 | -0.842724403 | 6.60E-31 |
| PTGDR | 2.58E-45 | -0.421101948 | 5.24E-41 | CKS2 | 2.98E-83 | -0.476547581 | 6.05E-79 | SLC2A3 | 4.16E-35 | 0.544568019 | 8.46E-31 |
| FGR | 3.13E-45 | -0.654484135 | 6.36E-41 | CCND3 | 4.00E-83 | 0.640649535 | 8.14E-79 | HOPX | 9.70E-35 | 0.459894301 | 1.97E-30 |
| RUNX2 | 3.24E-45 | 0.369272198 | 6.59E-41 | LOC100130476 | 6.35E-83 | -0.591087215 | 1.29E-78 | JAKMIP1 | 9.70E-35 | -0.353710123 | 1.97E-30 |
| CCL4L1 | 1.63E-44 | 0.274648508 | 3.31E-40 | CDC25B | 1.23E-82 | 0.481681755 | 2.49E-78 | FCGR3A | 9.83E-35 | -0.913897978 | 2.00E-30 |
| EZH2 | 1.72E-44 | 0.300485016 | 3.51E-40 | ID2 | 1.27E-82 | -0.759940846 | 2.59E-78 | ELL2 | 1.41E-34 | 0.345312815 | 2.87E-30 |
| MYBL1 | 1.93E-44 | -0.252976137 | 3.93E-40 | TBCD | 1.00E-81 | -0.76424855 | 2.04E-77 | TSC22D3 | 5.04E-34 | 0.49962472 | 1.02E-29 |
| KLF2 | 2.59E-44 | -0.300907846 | 5.27E-40 | SPRY1 | 1.06E-81 | -0.486380061 | 2.16E-77 | DOCK10 | 1.61E-33 | 0.41883356 | 3.26E-29 |
| DNAJA1 | 5.25E-44 | 0.621976597 | 1.07E-39 | RPL11 | 6.77E-81 | 0.504517675 | 1.38E-76 | PPP1CB | 2.69E-33 | 0.476923621 | 5.46E-29 |
| PABPC1 | 6.97E-44 | -0.345123806 | 1.42E-39 | TSC22D3 | 2.15E-80 | -1.124216731 | 4.37E-76 | VCAM1 | 2.89E-33 | -0.612418474 | 5.86E-29 |
| BIRC3 | 7.92E-44 | 0.41494535 | 1.61E-39 | HNRNPLL | 2.41E-80 | -0.628999783 | 4.90E-76 | PRF1 | 5.92E-33 | -0.466106175 | 1.20E-28 |
| JAKMIP1 | 8.01E-44 | 0.282727298 | 1.63E-39 | RPLP2 | 1.25E-79 | 0.560763832 | 2.54E-75 | LYST | 7.15E-33 | -0.41766542 | 1.45E-28 |
| ADD3 | 1.08E-43 | -0.600040227 | 2.20E-39 | H3F3B | 3.05E-79 | -0.484978811 | 6.21E-75 | PSMB9 | 1.11E-32 | -0.329299353 | 2.26E-28 |
| RPS8 | 1.42E-43 | -0.362464639 | 2.88E-39 | TCF7 | 1.03E-78 | 0.674525864 | 2.09E-74 | DDX3X | 1.19E-32 | 0.46442613 | 2.43E-28 |
| CD2 | 1.88E-43 | 0.358364624 | 3.81E-39 | LIME1 | 3.38E-78 | 0.530239129 | 6.86E-74 | GADD45B | 1.24E-32 | 0.314116303 | 2.52E-28 |
| SORL1 | 3.04E-43 | -0.344612075 | 6.18E-39 | SLA2 | 4.31E-78 | -0.704803816 | 8.77E-74 | FAM177A1 | 2.89E-32 | 0.456022582 | 5.87E-28 |
| ICAM2 | 3.48E-43 | -0.375501389 | 7.08E-39 | CD82 | 1.36E-77 | -0.964783353 | 2.76E-73 | UBE2F | 3.99E-32 | -0.421603468 | 8.11E-28 |
| PDE4B | 1.05E-42 | 0.409651365 | 2.13E-38 | ENTPD1 | 2.06E-77 | -0.992765501 | 4.18E-73 | S100A4 | 4.30E-32 | -0.384769274 | 8.74E-28 |
| SLC3A2 | 4.32E-42 | 0.41795635 | 8.79E-38 | RPS23 | 2.52E-77 | 0.482065298 | 5.11E-73 | XCL2 | 4.67E-32 | 0.336389902 | 9.50E-28 |
| TOX | 5.11E-42 | 0.438318632 | 1.04E-37 | HSPH1 | 3.43E-76 | -1.253216577 | 6.96E-72 | S1PR5 | 4.90E-32 | -0.516704561 | 9.97E-28 |
| RPL36 | 5.12E-42 | -0.257169038 | 1.04E-37 | PRMT2 | 3.91E-76 | 0.700055548 | 7.95E-72 | PLXDC1 | 5.81E-32 | 0.341142888 | 1.18E-27 |
| FCMR | 6.61E-42 | -0.395660462 | 1.34E-37 | SDCBP | 5.65E-76 | -0.963430328 | 1.15E-71 | PXN | 1.09E-31 | -0.501180884 | 2.21E-27 |
| AHI1 | 1.11E-41 | 0.311255237 | 2.26E-37 | RPL31 | 1.04E-75 | 0.596588635 | 2.11E-71 | AUTS2 | 1.55E-31 | 0.302718285 | 3.16E-27 |
| GZMK | 1.21E-41 | 0.442037695 | 2.46E-37 | UCP2 | 2.36E-75 | 0.658416906 | 4.79E-71 | PDE4D | 1.59E-31 | 0.340008534 | 3.23E-27 |
| SESN1 | 1.21E-41 | -0.392369254 | 2.47E-37 | RPL18 | 3.30E-75 | 0.492038383 | 6.70E-71 | CTSW | 1.76E-31 | -0.428030241 | 3.58E-27 |
| GALM | 1.61E-41 | 0.426677974 | 3.28E-37 | TSPAN32 | 4.24E-75 | 0.509030693 | 8.61E-71 | CAPN2 | 2.11E-31 | -0.414648235 | 4.30E-27 |
| RPL30 | 1.82E-41 | -0.373993476 | 3.71E-37 | ATM | 8.27E-75 | 0.604142635 | 1.68E-70 | CTLA4 | 2.26E-31 | -0.471454617 | 4.59E-27 |
| RPS13 | 3.29E-41 | -0.368156437 | 6.69E-37 | ST6GAL1 | 1.18E-74 | 0.569635944 | 2.39E-70 | TUBA1A | 4.12E-31 | 0.387580104 | 8.38E-27 |
| TXK | 6.99E-41 | -0.499339538 | 1.42E-36 | TANK | 1.80E-74 | -0.715900453 | 3.65E-70 | CENPBD1P1 | 7.09E-31 | 0.331352008 | 1.44E-26 |
| LAIR1 | 4.04E-40 | -0.493840619 | 8.22E-36 | ANKRD28 | 2.41E-74 | -0.833472431 | 4.89E-70 | FGR | 1.09E-30 | -0.629558336 | 2.22E-26 |
| HIF1A | 4.71E-40 | 0.479500206 | 9.58E-36 | MALAT1 | 3.39E-74 | 0.271176992 | 6.89E-70 | DUSP2 | 2.20E-30 | 0.483384599 | 4.48E-26 |
| CKS2 | 4.75E-40 | 0.272489539 | 9.64E-36 | RANBP2 | 4.22E-74 | -0.73101018 | 8.58E-70 | FURIN | 3.22E-30 | 0.294228036 | 6.53E-26 |
| RPL37A | 9.96E-40 | -0.264514709 | 2.02E-35 | VCL | 4.32E-74 | 0.537273507 | 8.78E-70 | MASTL | 3.47E-30 | 0.256657226 | 7.05E-26 |
| BCL2L11 | 1.04E-39 | 0.352404587 | 2.11E-35 | C1orf56 | 3.76E-73 | 0.451407872 | 7.64E-69 | GPR18 | 3.56E-30 | 0.477233912 | 7.24E-26 |
| MYL12A | 1.05E-39 | -0.264556308 | 2.13E-35 | SLC2A3 | 5.23E-73 | -0.897498313 | 1.06E-68 | SARAF | 5.45E-30 | 0.301016661 | 1.11E-25 |
| SLC38A2 | 2.06E-39 | 0.478253692 | 4.19E-35 | RPL37 | 7.14E-73 | 0.389195965 | 1.45E-68 | LMNA | 5.77E-30 | 0.262859887 | 1.17E-25 |
| RPL15 | 2.13E-39 | -0.346718752 | 4.33E-35 | ZC3H12A | 1.66E-72 | -0.71814846 | 3.37E-68 | UCP2 | 9.31E-30 | -0.448545059 | 1.89E-25 |
| RAB3GAP1 | 5.10E-39 | 0.274868222 | 1.04E-34 | RAB37 | 3.81E-72 | 0.581026429 | 7.74E-68 | SLC5A3 | 1.27E-29 | -0.365053609 | 2.59E-25 |
| IFI16 | 7.05E-39 | 0.401259684 | 1.43E-34 | GIMAP5 | 7.32E-72 | 0.603906695 | 1.49E-67 | PATL2 | 1.33E-29 | -0.63686142 | 2.69E-25 |
| LY9 | 1.37E-38 | -0.353344444 | 2.79E-34 | PTGDR | 7.96E-72 | 0.588892126 | 1.62E-67 | PLCG2 | 2.58E-29 | 0.28652428 | 5.25E-25 |
| TFRC | 1.56E-38 | 0.387650051 | 3.18E-34 | TFRC | 8.99E-72 | -0.651381848 | 1.83E-67 | CHD7 | 2.78E-29 | 0.27928648 | 5.64E-25 |
| PRR29 | 1.90E-38 | -0.307791135 | 3.86E-34 | MYO1F | 1.21E-71 | 0.687631705 | 2.46E-67 | CEP78 | 3.10E-29 | -0.389280281 | 6.30E-25 |
| ICOS | 2.36E-38 | 0.493823918 | 4.80E-34 | ATF3 | 1.62E-71 | -0.794327854 | 3.29E-67 | MGAT5 | 7.07E-29 | 0.319679393 | 1.44E-24 |
| SCML4 | 4.41E-38 | -0.451517593 | 8.95E-34 | HERPUD1 | 2.54E-71 | -0.866625978 | 5.17E-67 | GBP4 | 9.35E-29 | -0.350113445 | 1.90E-24 |
| AKAP5 | 4.73E-38 | 0.288025251 | 9.61E-34 | DUSP5 | 2.62E-71 | -0.294867116 | 5.32E-67 | GLA | 1.54E-28 | 0.38253815 | 3.13E-24 |
| CCDC141 | 5.06E-38 | 0.444728342 | 1.03E-33 | LAG3 | 2.62E-71 | -0.531738389 | 5.33E-67 | SNHG16 | 1.58E-28 | 0.303576495 | 3.20E-24 |
| CXCR3 | 7.06E-38 | 0.360092314 | 1.43E-33 | NFKBIZ | 2.67E-71 | -1.158253449 | 5.42E-67 | CXCR4 | 1.99E-28 | 0.320895595 | 4.05E-24 |
| RGS2 | 7.12E-38 | 0.472821309 | 1.45E-33 | LDLRAP1 | 2.87E-71 | 0.839710851 | 5.84E-67 | G3BP2 | 2.13E-28 | 0.421879666 | 4.32E-24 |
| IFI6 | 9.14E-38 | 0.416073236 | 1.86E-33 | BTG3 | 2.91E-71 | -0.593782207 | 5.92E-67 | TNFSF10 | 2.37E-28 | -0.338097633 | 4.81E-24 |
| NFKBIA | 1.00E-37 | 0.38035707 | 2.03E-33 | DDX3X | 3.26E-71 | -0.710061202 | 6.63E-67 | ITGB2-AS1 | 2.68E-28 | -0.347215516 | 5.44E-24 |
| B3GNT2 | 2.15E-37 | 0.36050203 | 4.37E-33 | PTCH1 | 6.25E-71 | 0.33436115 | 1.27E-66 | BBIP1 | 5.42E-28 | 0.324245401 | 1.10E-23 |
| SGMS1 | 2.69E-37 | 0.32395868 | 5.47E-33 | GABARAPL1 | 1.36E-70 | -0.614900133 | 2.76E-66 | PPP1R16B | 6.41E-28 | 0.309914439 | 1.30E-23 |
| TIAM1 | 3.71E-37 | 0.302908669 | 7.53E-33 | FAM177A1 | 4.94E-70 | -0.599497941 | 1.00E-65 | ABLIM1 | 7.28E-28 | 0.336154559 | 1.48E-23 |
| GALNT2 | 1.19E-36 | 0.299827775 | 2.42E-32 | KPNA2 | 8.17E-70 | -0.834306924 | 1.66E-65 | LIMS1 | 9.30E-28 | 0.346385757 | 1.89E-23 |
| RPL27A | 2.02E-36 | -0.277306245 | 4.11E-32 | MAFF | 1.12E-69 | -0.578919416 | 2.29E-65 | DENND4A | 9.37E-28 | 0.382751763 | 1.90E-23 |
| CCND3 | 3.22E-36 | -0.456009208 | 6.55E-32 | LIMD2 | 2.64E-69 | 0.49122257 | 5.37E-65 | SLC7A5P2 | 1.07E-27 | 0.271445506 | 2.17E-23 |
| MTHFD2 | 8.34E-36 | 0.384396241 | 1.69E-31 | CBLB | 3.30E-69 | -0.647875269 | 6.72E-65 | USP36 | 1.17E-27 | 0.393615071 | 2.38E-23 |
| ZAP70 | 1.10E-35 | -0.374642891 | 2.24E-31 | RPS4X | 1.07E-68 | 0.431532983 | 2.17E-64 | ZGPAT | 1.28E-27 | -0.325376307 | 2.60E-23 |
| RPS27A | 1.30E-35 | -0.260610785 | 2.64E-31 | FLNA | 1.41E-68 | 0.575888428 | 2.87E-64 | ADD3 | 1.39E-27 | -0.557619523 | 2.83E-23 |
| SH2D1A | 1.45E-35 | 0.290231146 | 2.95E-31 | RNF19A | 1.70E-68 | -0.789651868 | 3.45E-64 | TRAF3IP3 | 1.43E-27 | -0.400529414 | 2.90E-23 |
| TNFRSF18 | 1.81E-35 | 0.345974303 | 3.68E-31 | RPL36 | 2.65E-68 | 0.368113197 | 5.38E-64 | TUBB4B | 1.77E-27 | 0.319950891 | 3.60E-23 |
| RPS20 | 2.20E-35 | -0.27634312 | 4.48E-31 | RPL30 | 2.94E-68 | 0.518412728 | 5.97E-64 | SELL | 1.84E-27 | -1.007067214 | 3.74E-23 |
| CCND2 | 3.10E-35 | 0.390881755 | 6.30E-31 | ADGRG1 | 6.50E-68 | 0.882357278 | 1.32E-63 | ARL5B | 2.93E-27 | 0.330854686 | 5.96E-23 |
| HLA-DPB1 | 3.17E-35 | 0.286519877 | 6.44E-31 | G3BP2 | 1.13E-67 | -0.69159765 | 2.30E-63 | SOCS3 | 3.10E-27 | 0.378159131 | 6.30E-23 |
| HSP90AB1 | 4.75E-35 | 0.648031387 | 9.65E-31 | ZGPAT | 1.86E-67 | 0.492693465 | 3.78E-63 | GIMAP7 | 4.00E-27 | -0.399963457 | 8.13E-23 |
| TARP | 6.03E-35 | -0.51164661 | 1.23E-30 | EIF4A3 | 1.90E-67 | -0.695795776 | 3.86E-63 | ETF1 | 9.58E-27 | 0.356433733 | 1.95E-22 |
| MX1 | 6.90E-35 | 0.480316606 | 1.40E-30 | BCL2L11 | 4.62E-67 | -0.592165929 | 9.40E-63 | PDE4B | 1.08E-26 | 0.286773433 | 2.20E-22 |
| CYTIP | 6.96E-35 | 0.30758527 | 1.41E-30 | LITAF | 6.46E-67 | 0.500240709 | 1.31E-62 | PZP | 1.18E-26 | -0.313117105 | 2.39E-22 |
| RPLP0 | 7.53E-35 | -0.279841753 | 1.53E-30 | C1orf21 | 7.29E-67 | 0.467214964 | 1.48E-62 | DCTN6 | 1.24E-26 | 0.382004922 | 2.53E-22 |
| CDK6 | 8.12E-35 | 0.333840034 | 1.65E-30 | HSP90AB1 | 9.35E-67 | -0.813655081 | 1.90E-62 | PIK3R5 | 1.33E-26 | -0.302362446 | 2.70E-22 |
| HSP90AA1 | 9.22E-35 | 0.897068589 | 1.87E-30 | LINC00612 | 9.89E-67 | 0.385757412 | 2.01E-62 | IRF4 | 1.39E-26 | 0.361556181 | 2.83E-22 |
| OAS3 | 1.18E-34 | 0.336923314 | 2.40E-30 | RPL37A | 1.22E-66 | 0.388707036 | 2.49E-62 | UBE2F-SCLY | 1.56E-26 | -0.295808089 | 3.17E-22 |
| SLC1A5 | 2.04E-34 | 0.303983786 | 4.14E-30 | LMNA | 1.24E-66 | -1.070559567 | 2.51E-62 | GLUL | 1.79E-26 | -0.531540726 | 3.64E-22 |
| FASLG | 2.32E-34 | 0.385937068 | 4.71E-30 | RPS8 | 1.93E-66 | 0.4856384 | 3.92E-62 | AHNAK | 1.80E-26 | -0.319927731 | 3.66E-22 |
| CKAP2 | 3.35E-34 | 0.258609624 | 6.80E-30 | CD7 | 2.02E-66 | -0.377824219 | 4.11E-62 | CD83 | 2.50E-26 | 0.332288002 | 5.07E-22 |
| TRAF3IP3 | 3.76E-34 | -0.414936887 | 7.63E-30 | RPL35 | 2.14E-66 | 0.281485875 | 4.36E-62 | SELPLG | 3.62E-26 | -0.368631577 | 7.36E-22 |
| DYNLL1 | 4.22E-34 | 0.271164316 | 8.58E-30 | RPL4 | 3.12E-66 | 0.446313783 | 6.34E-62 | BATF | 4.15E-26 | -0.273018461 | 8.43E-22 |
| TSPAN32 | 4.55E-34 | -0.347857581 | 9.24E-30 | ICOS | 5.67E-66 | -1.031070925 | 1.15E-61 | TBC1D4 | 4.39E-26 | -0.332363192 | 8.93E-22 |
| ARHGAP25 | 5.07E-34 | -0.46351046 | 1.03E-29 | RPS12 | 8.96E-66 | 0.49496132 | 1.82E-61 | PERP | 4.76E-26 | 0.296451887 | 9.68E-22 |
| KDM5B | 5.37E-34 | 0.288082425 | 1.09E-29 | SYTL3 | 8.98E-66 | -0.637040235 | 1.83E-61 | TMEM2 | 5.19E-26 | 0.480039967 | 1.05E-21 |
| NAMPT | 1.27E-33 | 0.32042676 | 2.59E-29 | NEDD9 | 1.16E-65 | -0.587490544 | 2.36E-61 | IFI44 | 6.62E-26 | -0.365851874 | 1.35E-21 |
| PLEKHA1 | 1.55E-33 | -0.39262778 | 3.15E-29 | RPS27A | 1.23E-65 | 0.381734731 | 2.50E-61 | SNAP47 | 1.72E-25 | -0.324235985 | 3.49E-21 |
| PDE4D | 1.70E-33 | 0.287871577 | 3.45E-29 | PCED1B-AS1 | 1.33E-65 | 0.466326327 | 2.71E-61 | STAT4 | 2.06E-25 | 0.266323885 | 4.19E-21 |
| IFI44L | 2.25E-33 | 0.464395256 | 4.57E-29 | CD38 | 1.82E-65 | -0.523392092 | 3.70E-61 | SYTL2 | 2.19E-25 | 0.34193285 | 4.44E-21 |
| CREM | 3.07E-33 | 0.407505979 | 6.23E-29 | GPR15 | 5.27E-65 | -0.729418075 | 1.07E-60 | SNHG15 | 3.11E-25 | 0.338046003 | 6.31E-21 |
| RPL10A | 4.57E-33 | -0.303800065 | 9.28E-29 | PMAIP1 | 1.04E-64 | -0.878699369 | 2.12E-60 | C1orf162 | 3.29E-25 | -0.357263227 | 6.69E-21 |
| TGFBR3 | 5.89E-33 | -0.414121701 | 1.20E-28 | IDI1 | 1.62E-64 | -0.676524122 | 3.29E-60 | SLA2 | 3.31E-25 | 0.416247954 | 6.72E-21 |
| FKBP1A | 5.96E-33 | 0.39061236 | 1.21E-28 | SLA | 1.81E-64 | -0.823178344 | 3.68E-60 | MORF4L2 | 3.40E-25 | 0.345888829 | 6.92E-21 |
| HSPD1 | 7.16E-33 | 0.783438914 | 1.45E-28 | RPL26 | 1.48E-63 | 0.331755055 | 3.00E-59 | TXNIP | 4.51E-25 | -0.430216414 | 9.16E-21 |
| CCR5 | 7.22E-33 | 0.316850396 | 1.47E-28 | ATF4 | 3.73E-63 | -0.573399674 | 7.57E-59 | BTN3A2 | 5.27E-25 | -0.356244783 | 1.07E-20 |
| ARID5B | 1.72E-32 | 0.432334051 | 3.50E-28 | FAM53C | 3.87E-63 | -0.63036225 | 7.86E-59 | AVPI1 | 7.22E-25 | 0.260072195 | 1.47E-20 |
| CLIC1 | 2.88E-32 | 0.28386615 | 5.86E-28 | SLC38A2 | 4.81E-63 | -0.642144034 | 9.78E-59 | PRMT2 | 8.02E-25 | -0.406326486 | 1.63E-20 |
| LILRB1 | 3.36E-32 | -0.305669685 | 6.83E-28 | ELF1 | 1.41E-62 | -0.6022087 | 2.87E-58 | P2RX4 | 8.66E-25 | 0.3496621 | 1.76E-20 |
| TPT1 | 6.04E-32 | -0.252763813 | 1.23E-27 | FCMR | 1.73E-62 | 0.529658776 | 3.52E-58 | IL7R | 1.02E-24 | 0.327456791 | 2.07E-20 |
| RALA | 7.37E-32 | 0.254131888 | 1.50E-27 | NEU1 | 2.64E-62 | -0.781316784 | 5.37E-58 | SAT1 | 1.02E-24 | 0.435829461 | 2.08E-20 |
| TRABD2A | 8.07E-32 | -0.344362591 | 1.64E-27 | HIF1A | 2.68E-62 | -0.688325683 | 5.44E-58 | SYTL1 | 1.65E-24 | -0.338734977 | 3.35E-20 |
| CACYBP | 2.70E-31 | 0.451450251 | 5.48E-27 | ACP5 | 2.98E-62 | -0.676452377 | 6.06E-58 | CD101 | 1.76E-24 | 0.308937546 | 3.57E-20 |
| A2M | 3.42E-31 | -0.388724635 | 6.95E-27 | TMEM63A | 5.03E-62 | 0.555498685 | 1.02E-57 | RBPJ | 1.89E-24 | -0.722447621 | 3.83E-20 |
| RSAD2 | 3.89E-31 | 0.323392422 | 7.90E-27 | GPR171 | 7.02E-62 | -0.639918781 | 1.43E-57 | MIR155HG | 2.50E-24 | -0.345749526 | 5.08E-20 |
| RPS9 | 3.89E-31 | -0.272437506 | 7.91E-27 | RPS29 | 1.00E-61 | 0.352748722 | 2.04E-57 | RNF139 | 3.07E-24 | 0.406492361 | 6.25E-20 |
| SPON2 | 5.00E-31 | -0.401078015 | 1.02E-26 | C11orf21 | 1.33E-61 | 0.420072595 | 2.71E-57 | ZC3H12A | 4.07E-24 | 0.323922135 | 8.27E-20 |
| C1orf56 | 6.72E-31 | -0.319344898 | 1.37E-26 | EGR2 | 3.27E-61 | -0.874302002 | 6.64E-57 | LIMD2 | 5.04E-24 | -0.304561597 | 1.02E-19 |
| SERPINB6 | 1.02E-30 | -0.346327496 | 2.07E-26 | RPS25 | 3.33E-61 | 0.37590595 | 6.77E-57 | PYHIN1 | 5.23E-24 | -0.332704769 | 1.06E-19 |
| HSPB1 | 1.74E-30 | 0.545007464 | 3.54E-26 | FASLG | 3.88E-61 | -0.73133589 | 7.88E-57 | OAS2 | 6.57E-24 | -0.383789612 | 1.34E-19 |
| MYC | 4.77E-30 | -0.453516198 | 9.69E-26 | FTL | 5.97E-61 | 0.317523505 | 1.21E-56 | SNHG12 | 9.55E-24 | 0.273215877 | 1.94E-19 |
| IFI44 | 4.88E-30 | 0.378270945 | 9.91E-26 | SNORA33 | 8.54E-61 | 0.440938633 | 1.74E-56 | DNAJB9 | 1.17E-23 | 0.315411142 | 2.38E-19 |
| DENND2D | 1.01E-29 | -0.310852165 | 2.04E-25 | TRABD2A | 9.28E-61 | 0.52650838 | 1.89E-56 | TGFBR3 | 1.30E-23 | -0.370926021 | 2.64E-19 |
| PGK1 | 1.05E-29 | 0.29108418 | 2.13E-25 | SLC1A5 | 3.11E-60 | -0.535030736 | 6.32E-56 | ALG13 | 1.35E-23 | 0.399854325 | 2.74E-19 |
| SLBP | 1.21E-29 | 0.267562484 | 2.46E-25 | VPS37B | 3.61E-60 | -0.444927123 | 7.34E-56 | MAFF | 1.80E-23 | 0.285729951 | 3.66E-19 |
| LEF1 | 2.64E-29 | -0.619762758 | 5.37E-25 | ICAM2 | 3.90E-60 | 0.481807234 | 7.92E-56 | MRPS6 | 1.87E-23 | -0.303608487 | 3.79E-19 |
| TRAF5 | 2.97E-29 | 0.348098566 | 6.04E-25 | RPL29 | 4.04E-60 | 0.339372478 | 8.22E-56 | ZFAND5 | 1.89E-23 | 0.345778668 | 3.85E-19 |
| IDH2 | 3.49E-29 | 0.344617882 | 7.09E-25 | ASB2 | 5.74E-60 | -0.560586436 | 1.17E-55 | GPR171 | 2.57E-23 | 0.354674112 | 5.23E-19 |
| AKIRIN2 | 4.14E-29 | 0.308473754 | 8.41E-25 | SRGAP3 | 5.95E-60 | -0.437547099 | 1.21E-55 | EZR | 3.57E-23 | 0.359185099 | 7.25E-19 |
| OTULIN | 4.61E-29 | 0.254527123 | 9.36E-25 | RPS13 | 7.01E-60 | 0.481122047 | 1.42E-55 | MTFP1 | 5.51E-23 | 0.27143063 | 1.12E-18 |
| MRPS6 | 6.07E-29 | 0.260232174 | 1.23E-24 | ALOX5AP | 1.78E-59 | -0.589296425 | 3.62E-55 | ZNF683 | 7.50E-23 | -0.576729388 | 1.52E-18 |
| RACK1 | 7.53E-29 | -0.276689819 | 1.53E-24 | OSBPL5 | 2.14E-59 | 0.403127572 | 4.36E-55 | STK38 | 7.78E-23 | -0.364888013 | 1.58E-18 |
| CTSD | 1.99E-28 | 0.362597137 | 4.04E-24 | CD300A | 2.35E-59 | 0.615398216 | 4.78E-55 | ODF2L | 8.14E-23 | 0.314046205 | 1.65E-18 |
| OAS1 | 2.19E-28 | 0.342430746 | 4.46E-24 | UBA52 | 4.34E-59 | 0.38265082 | 8.81E-55 | IFI44L | 1.69E-22 | -0.439930381 | 3.44E-18 |
| UBA52 | 2.29E-28 | -0.25580775 | 4.65E-24 | RPL8 | 4.36E-59 | 0.373534367 | 8.87E-55 | EVI2A | 2.17E-22 | 0.396013057 | 4.42E-18 |
| STK38 | 4.24E-28 | -0.43935636 | 8.61E-24 | ABCB1 | 4.99E-59 | -0.411440167 | 1.01E-54 | PPP1R15B | 3.66E-22 | 0.255062265 | 7.44E-18 |
| JMJD6 | 4.62E-28 | 0.31906113 | 9.39E-24 | CDC42SE1 | 6.55E-59 | 0.487997408 | 1.33E-54 | FUS | 4.15E-22 | 0.281849078 | 8.43E-18 |
| TMEM63A | 5.76E-28 | -0.353422921 | 1.17E-23 | PRMT9 | 9.91E-59 | -0.466619999 | 2.01E-54 | INPP4B | 4.24E-22 | -0.355971251 | 8.61E-18 |
| TMIGD2 | 6.36E-28 | -0.383322066 | 1.29E-23 | IDI2-AS1 | 1.19E-58 | -0.326922372 | 2.41E-54 | NFKBID | 7.73E-22 | 0.40271249 | 1.57E-17 |
| GSAP | 1.65E-27 | -0.314140633 | 3.35E-23 | HSPD1 | 1.43E-58 | -0.91802814 | 2.90E-54 | NFKBIA | 1.29E-21 | 0.412824292 | 2.63E-17 |
| RBL2 | 1.73E-27 | -0.334333925 | 3.51E-23 | SIRPG | 2.67E-58 | -0.688648031 | 5.43E-54 | ARHGAP9 | 1.33E-21 | 0.28330442 | 2.70E-17 |
| LDLRAP1 | 1.89E-27 | -0.585689693 | 3.84E-23 | LILRB1 | 3.22E-58 | 0.423804014 | 6.55E-54 | SENP3-EIF4A1 | 1.35E-21 | 0.267730005 | 2.75E-17 |
| BTG1 | 2.04E-27 | 0.25976729 | 4.14E-23 | HLA-E | 8.95E-58 | 0.291294666 | 1.82E-53 | BRD2 | 1.65E-21 | 0.332202897 | 3.35E-17 |
| TBC1D4 | 2.31E-27 | 0.331214804 | 4.69E-23 | PTTG1 | 2.91E-57 | -0.361820768 | 5.91E-53 | ISYNA1 | 1.67E-21 | 0.254665836 | 3.40E-17 |
| CD58 | 3.13E-27 | 0.261125732 | 6.37E-23 | RPS10-NUDT3 | 3.84E-57 | 0.268180807 | 7.81E-53 | ST6GAL1 | 2.18E-21 | -0.302382666 | 4.43E-17 |
| YARS | 3.49E-27 | 0.27804595 | 7.09E-23 | UBC | 6.03E-57 | -0.428381163 | 1.22E-52 | ADRB2 | 2.64E-21 | -0.44882062 | 5.36E-17 |
| ZFAND2A | 3.99E-27 | 0.344440026 | 8.11E-23 | KLRC1 | 7.86E-57 | -0.938871211 | 1.60E-52 | TMEM173 | 3.12E-21 | -0.395400562 | 6.34E-17 |
| SELL | 4.90E-27 | -0.884832256 | 9.97E-23 | SH3BP5 | 1.48E-56 | 0.544635002 | 3.01E-52 | IFRD1 | 4.72E-21 | 0.296139107 | 9.59E-17 |
| RCBTB2 | 6.10E-27 | -0.348251616 | 1.24E-22 | RAP2B | 3.00E-56 | 0.526713359 | 6.10E-52 | C1orf21 | 4.84E-21 | -0.300849167 | 9.83E-17 |
| CHORDC1 | 8.11E-27 | 0.453154173 | 1.65E-22 | PGK1 | 3.08E-56 | -0.548944928 | 6.25E-52 | FUT8 | 6.45E-21 | -0.268843574 | 1.31E-16 |
| LAYN | 1.33E-26 | 0.288251421 | 2.70E-22 | SRSF2 | 3.73E-56 | -0.577298617 | 7.58E-52 | PRKCB | 6.86E-21 | -0.253880444 | 1.39E-16 |
| IFITM1 | 1.82E-26 | -0.278035701 | 3.70E-22 | SH2D2A | 5.72E-56 | -0.408362065 | 1.16E-51 | RPS15A | 8.53E-21 | -0.271462724 | 1.73E-16 |
| GZMM | 3.94E-26 | -0.268468196 | 8.01E-22 | SENP3-EIF4A1 | 5.75E-56 | -0.467798757 | 1.17E-51 | RHOC | 1.34E-20 | 0.387992586 | 2.73E-16 |
| AHSA1 | 5.56E-26 | 0.354417623 | 1.13E-21 | AHR | 9.57E-56 | -0.547271912 | 1.94E-51 | ELF1 | 1.44E-20 | 0.30426551 | 2.93E-16 |
| LAP3 | 6.22E-26 | 0.286980089 | 1.26E-21 | SYTL1 | 1.03E-55 | 0.520998185 | 2.09E-51 | AKAP13 | 1.53E-20 | 0.339849655 | 3.10E-16 |
| CHN1 | 6.31E-26 | 0.308645651 | 1.28E-21 | RPS9 | 1.16E-55 | 0.41665537 | 2.35E-51 | SELENOK | 1.53E-20 | 0.275799361 | 3.12E-16 |
| KLF12 | 6.33E-26 | -0.284452534 | 1.29E-21 | TMEM2 | 1.75E-55 | -0.723834458 | 3.56E-51 | SRSF3 | 1.76E-20 | 0.320719218 | 3.58E-16 |
| HSPA8 | 6.34E-26 | 0.541174965 | 1.29E-21 | ADAM19 | 1.77E-55 | -0.671605979 | 3.60E-51 | HLA-DMA | 2.18E-20 | -0.355001168 | 4.43E-16 |
| IRF4 | 6.86E-26 | 0.292750271 | 1.40E-21 | S100A6 | 1.96E-55 | 0.359617244 | 3.98E-51 | PLAC8 | 2.31E-20 | -0.399058241 | 4.69E-16 |
| HSPE1 | 1.06E-25 | 0.426250459 | 2.16E-21 | CYTH1 | 4.95E-55 | 0.572270572 | 1.01E-50 | YWHAQ | 2.52E-20 | -0.276624622 | 5.12E-16 |
| HAUS3 | 2.02E-25 | 0.312251408 | 4.11E-21 | ZFAND5 | 7.20E-55 | -0.612879756 | 1.46E-50 | HMGN3 | 3.38E-20 | -0.311780946 | 6.86E-16 |
| ATM | 2.24E-25 | -0.351888289 | 4.55E-21 | DCTN6 | 7.25E-55 | -0.593711054 | 1.47E-50 | CCL3L3 | 6.32E-20 | 0.504823421 | 1.28E-15 |
| ITGAL | 2.81E-25 | -0.34670135 | 5.71E-21 | IFRD1 | 7.60E-55 | -0.680070703 | 1.54E-50 | LUC7L3 | 1.06E-19 | -0.324824804 | 2.16E-15 |
| TIGIT | 2.97E-25 | 0.373482912 | 6.03E-21 | RPS19 | 8.12E-55 | 0.424949362 | 1.65E-50 | GZMB | 1.23E-19 | -0.386282622 | 2.49E-15 |
| CD9 | 3.46E-25 | 0.305976356 | 7.03E-21 | P2RY10 | 1.14E-54 | -0.559209767 | 2.31E-50 | TESPA1 | 2.38E-19 | -0.308406583 | 4.84E-15 |
| PRDM1 | 5.25E-25 | 0.346810195 | 1.07E-20 | RPS15A | 1.70E-54 | 0.42282922 | 3.46E-50 | TNF | 3.01E-19 | 0.596483889 | 6.12E-15 |
| CD300A | 1.68E-24 | -0.405821676 | 3.42E-20 | GLA | 2.62E-54 | -0.71815876 | 5.33E-50 | PPP1R10 | 4.06E-19 | 0.297996488 | 8.26E-15 |
| PRDX6 | 1.82E-24 | 0.287690359 | 3.71E-20 | NAB1 | 7.51E-54 | -0.505246662 | 1.53E-49 | SNHG1 | 4.28E-19 | 0.254379289 | 8.69E-15 |
| BATF | 2.36E-24 | 0.25157725 | 4.80E-20 | PTGER2 | 7.58E-54 | 0.496646771 | 1.54E-49 | HLA-DMB | 4.62E-19 | -0.323822325 | 9.39E-15 |
| GBP2 | 6.63E-24 | 0.346467853 | 1.35E-19 | PELI1 | 7.69E-54 | -0.517182236 | 1.56E-49 | IDS | 4.89E-19 | 0.312137971 | 9.93E-15 |
| ALOX5AP | 8.38E-24 | 0.388395875 | 1.70E-19 | S1PR4 | 7.73E-54 | 0.418792514 | 1.57E-49 | CRYBG1 | 4.92E-19 | 0.617490872 | 1.00E-14 |
| PRDX3 | 9.54E-24 | 0.264878739 | 1.94E-19 | MYO7A | 8.12E-54 | -0.389732355 | 1.65E-49 | CCL3L1 | 5.14E-19 | 0.507802307 | 1.05E-14 |
| PTPN7 | 1.02E-23 | 0.320231994 | 2.07E-19 | LUC7L3 | 1.04E-53 | 0.481846022 | 2.12E-49 | OSTF1 | 6.22E-19 | 0.315963158 | 1.27E-14 |
| STMN1 | 3.23E-23 | 0.625247875 | 6.56E-19 | TSPYL2 | 1.16E-53 | -0.922304413 | 2.35E-49 | PNPLA8 | 6.36E-19 | 0.287776434 | 1.29E-14 |
| H2AFZ | 4.01E-23 | 0.317632875 | 8.16E-19 | DAPK2 | 1.45E-53 | -0.458643884 | 2.95E-49 | CCL4L1 | 8.87E-19 | 0.490737677 | 1.80E-14 |
| CD200R1 | 5.37E-23 | 0.30110081 | 1.09E-18 | GZMB | 2.97E-53 | -0.944592873 | 6.04E-49 | TPST2 | 9.38E-19 | -0.283772578 | 1.91E-14 |
| MYO1F | 6.98E-23 | -0.41701756 | 1.42E-18 | ITM2A | 3.07E-53 | -0.6524228 | 6.24E-49 | DDX21 | 9.86E-19 | 0.316437507 | 2.00E-14 |
| RPLP1 | 7.43E-23 | -0.272855524 | 1.51E-18 | BRD2 | 3.46E-53 | -0.633309281 | 7.04E-49 | LAT2 | 2.70E-18 | 0.369947937 | 5.49E-14 |
| CDC42SE1 | 9.06E-23 | -0.328640055 | 1.84E-18 | ODC1 | 5.24E-53 | -0.592175182 | 1.07E-48 | GPR183 | 3.05E-18 | 0.309904263 | 6.19E-14 |
| MCM6 | 1.06E-22 | 0.33167337 | 2.16E-18 | CCL3L3 | 8.20E-53 | -0.45320541 | 1.67E-48 | ABI3 | 3.37E-18 | 0.340725497 | 6.86E-14 |
| TC2N | 1.62E-22 | -0.26935436 | 3.30E-18 | PRR29 | 1.63E-52 | 0.401574391 | 3.31E-48 | CYCS | 4.60E-18 | 0.301142021 | 9.36E-14 |
| TBL1XR1 | 2.82E-22 | 0.30321819 | 5.72E-18 | PTP4A1 | 3.74E-52 | -0.643728182 | 7.60E-48 | ATP5A1 | 4.72E-18 | -0.279927892 | 9.59E-14 |
| CTSA | 3.07E-22 | 0.33737398 | 6.23E-18 | ADRB2 | 6.05E-52 | 0.609648575 | 1.23E-47 | TIAM1 | 4.75E-18 | -0.250469368 | 9.65E-14 |
| PMAIP1 | 3.37E-22 | 0.476117276 | 6.85E-18 | RPL27 | 6.53E-52 | 0.361757893 | 1.33E-47 | LY75 | 4.94E-18 | -0.274440241 | 1.00E-13 |
| BPGM | 3.60E-22 | 0.321433179 | 7.31E-18 | GALNT2 | 1.01E-51 | -0.362313346 | 2.06E-47 | CCNH | 7.63E-18 | 0.35703064 | 1.55E-13 |
| KPNA2 | 4.56E-22 | 0.306265117 | 9.27E-18 | GMFG | 1.97E-51 | 0.347956468 | 4.00E-47 | CCL4 | 1.02E-17 | 0.59089942 | 2.07E-13 |
| NOL4L | 4.74E-22 | -0.308688213 | 9.64E-18 | BHLHE40 | 2.20E-51 | -0.68362965 | 4.48E-47 | PPM1M | 1.06E-17 | -0.276601801 | 2.16E-13 |
| EIF4B | 5.63E-22 | -0.295310496 | 1.14E-17 | CCL3L1 | 2.82E-51 | -0.438512264 | 5.73E-47 | RELL1 | 1.12E-17 | 0.281640951 | 2.28E-13 |
| SLC44A2 | 6.21E-22 | -0.333282386 | 1.26E-17 | MTHFD2 | 3.14E-51 | -0.603642213 | 6.38E-47 | YARS | 1.50E-17 | -0.274839986 | 3.05E-13 |
| ADHFE1 | 1.03E-21 | -0.265681709 | 2.10E-17 | GPSM3 | 3.76E-51 | 0.321274917 | 7.63E-47 | TARP | 1.60E-17 | 0.343527828 | 3.26E-13 |
| MBP | 1.18E-21 | -0.37360955 | 2.40E-17 | ADGRE5 | 5.53E-51 | -0.583266883 | 1.12E-46 | RUNX3 | 1.96E-17 | 0.27367835 | 3.98E-13 |
| TRAT1 | 1.44E-21 | 0.253168192 | 2.92E-17 | ARHGAP25 | 5.91E-51 | 0.561306933 | 1.20E-46 | MED29 | 2.16E-17 | 0.267998684 | 4.40E-13 |
| STIP1 | 1.71E-21 | 0.336419347 | 3.47E-17 | NUAK2 | 6.23E-51 | 0.388923408 | 1.27E-46 | CD84 | 2.49E-17 | -0.270293341 | 5.07E-13 |
| PON2 | 2.17E-21 | 0.266414032 | 4.42E-17 | RAB29 | 7.88E-51 | 0.552086648 | 1.60E-46 | GZMK | 2.65E-17 | -0.312706416 | 5.38E-13 |
| FRMD4B | 2.72E-21 | 0.257676293 | 5.53E-17 | TNFRSF1B | 1.07E-50 | -0.519608203 | 2.17E-46 | BTG1 | 2.83E-17 | 0.301421022 | 5.76E-13 |
| SP140 | 3.27E-21 | 0.257888088 | 6.64E-17 | RPS20 | 1.32E-50 | 0.377533286 | 2.68E-46 | SERPINB9 | 3.06E-17 | 0.521950369 | 6.21E-13 |
| TPI1 | 3.31E-21 | 0.287849738 | 6.72E-17 | CD9 | 1.63E-50 | -0.484702041 | 3.31E-46 | SCAMP2 | 3.30E-17 | -0.256938525 | 6.70E-13 |
| PKM | 3.50E-21 | 0.386305843 | 7.11E-17 | TNFSF4 | 1.82E-50 | -0.624379184 | 3.71E-46 | EGR2 | 3.31E-17 | 0.43723582 | 6.72E-13 |
| MFNG | 3.59E-21 | -0.399279955 | 7.29E-17 | ELL2 | 2.55E-50 | -0.368932004 | 5.17E-46 | HSPB1 | 4.52E-17 | -0.392299792 | 9.18E-13 |
| FLNA | 3.60E-21 | -0.343655244 | 7.31E-17 | RALGAPA1 | 2.73E-50 | -0.327699935 | 5.54E-46 | SLC5A6 | 5.76E-17 | 0.307772774 | 1.17E-12 |
| PZP | 3.76E-21 | -0.272224021 | 7.63E-17 | ABCG1 | 3.02E-50 | -0.399211935 | 6.13E-46 | JUN | 6.01E-17 | 0.475601786 | 1.22E-12 |
| LDLR | 5.20E-21 | 0.331175542 | 1.06E-16 | AKAP5 | 3.28E-50 | -0.440489999 | 6.66E-46 | SQOR | 6.85E-17 | -0.287962678 | 1.39E-12 |
| DNAJB1 | 5.41E-21 | 1.126524792 | 1.10E-16 | SLC27A2 | 3.36E-50 | -0.467249821 | 6.83E-46 | BHLHE40 | 1.31E-16 | -0.360030493 | 2.67E-12 |
| PIP4K2A | 6.95E-21 | -0.297752759 | 1.41E-16 | PFDN5 | 7.31E-50 | 0.371904928 | 1.49E-45 | IDI1 | 1.47E-16 | 0.273038341 | 2.99E-12 |
| SMIM3 | 7.82E-21 | -0.394837965 | 1.59E-16 | CNOT6L | 8.85E-50 | -0.522844479 | 1.80E-45 | RAP2B | 1.64E-16 | -0.284022064 | 3.33E-12 |
| TTN | 8.06E-21 | 0.431514825 | 1.64E-16 | SH3BP5-AS1 | 1.11E-49 | 0.482155201 | 2.25E-45 | GBP2 | 1.72E-16 | -0.304290657 | 3.49E-12 |
| SRSF2 | 8.63E-21 | 0.264808918 | 1.75E-16 | KDM6B | 1.65E-49 | -0.364236898 | 3.34E-45 | EIF4A3 | 1.83E-16 | 0.301598858 | 3.71E-12 |
| CYTH1 | 8.64E-21 | -0.371044678 | 1.76E-16 | THEM4 | 4.61E-49 | 0.40214856 | 9.37E-45 | PAM | 2.17E-16 | -0.251144813 | 4.40E-12 |
| CTSB | 1.17E-20 | 0.305519281 | 2.38E-16 | THEMIS2 | 6.27E-49 | 0.575864333 | 1.27E-44 | CYTIP | 2.49E-16 | 0.251687941 | 5.05E-12 |
| GSTK1 | 1.54E-20 | -0.263430555 | 3.14E-16 | TIPARP | 7.11E-49 | -0.716204305 | 1.45E-44 | LEF1 | 2.77E-16 | -0.461398461 | 5.62E-12 |
| STX11 | 1.58E-20 | 0.281882612 | 3.20E-16 | GTF2B | 7.59E-49 | -0.549619021 | 1.54E-44 | GABARAPL1 | 2.79E-16 | 0.299568806 | 5.66E-12 |
| S100A4 | 1.63E-20 | -0.363353644 | 3.30E-16 | ARHGAP9 | 8.96E-49 | -0.432875898 | 1.82E-44 | RBBP4 | 3.13E-16 | -0.317221291 | 6.35E-12 |
| SIRPG | 3.84E-20 | 0.333210141 | 7.80E-16 | RPS24 | 1.21E-48 | 0.379623547 | 2.46E-44 | MRFAP1L1 | 3.60E-16 | -0.257326414 | 7.32E-12 |
| KLRG1 | 4.87E-20 | -0.538268699 | 9.90E-16 | ENTPD1-AS1 | 1.43E-48 | -0.268460751 | 2.90E-44 | UBE2L6 | 3.98E-16 | -0.294378918 | 8.09E-12 |
| UBB | 5.48E-20 | 0.256473076 | 1.11E-15 | ZAP70 | 1.88E-48 | 0.446295358 | 3.82E-44 | NR3C1 | 4.24E-16 | 0.329979624 | 8.61E-12 |
| PELI1 | 7.33E-20 | 0.267365665 | 1.49E-15 | CRTAM | 2.50E-48 | -0.867803586 | 5.08E-44 | SAMD9L | 4.63E-16 | -0.343589015 | 9.41E-12 |
| IL16 | 1.20E-19 | -0.364337944 | 2.43E-15 | CCM2 | 2.68E-48 | 0.374363102 | 5.45E-44 | GSPT1 | 6.08E-16 | 0.267579685 | 1.24E-11 |
| GIMAP4 | 1.68E-19 | -0.322778108 | 3.42E-15 | RPL15 | 2.13E-47 | 0.405071929 | 4.33E-43 | TRIM39 | 8.52E-16 | 0.289701587 | 1.73E-11 |
| VSIR | 1.70E-19 | -0.30036283 | 3.45E-15 | HSPA1L | 2.53E-47 | -1.033853316 | 5.15E-43 | TES | 8.61E-16 | -0.323657072 | 1.75E-11 |
| SUN2 | 1.87E-19 | -0.309122767 | 3.81E-15 | SNHG12 | 2.69E-47 | -0.428580663 | 5.47E-43 | SLAMF6 | 9.10E-16 | -0.267474046 | 1.85E-11 |
| PATL2 | 2.68E-19 | -0.597720174 | 5.46E-15 | DUSP16 | 3.98E-47 | -0.429498375 | 8.09E-43 | DAPK2 | 1.43E-15 | 0.350605176 | 2.90E-11 |
| EPB41 | 5.19E-19 | -0.273862824 | 1.05E-14 | WHRN | 5.04E-47 | -0.255899089 | 1.02E-42 | OAS1 | 1.57E-15 | -0.332768721 | 3.19E-11 |
| SH3BP5 | 8.50E-19 | -0.329169393 | 1.73E-14 | TUBB4B | 6.09E-47 | -0.631255736 | 1.24E-42 | IFI16 | 1.85E-15 | -0.295973404 | 3.76E-11 |
| SH3BP5-AS1 | 8.95E-19 | -0.292461556 | 1.82E-14 | AHNAK | 6.82E-47 | 0.439847093 | 1.39E-42 | NOSIP | 2.01E-15 | -0.340230833 | 4.08E-11 |
| SLC25A5 | 9.05E-19 | 0.267130928 | 1.84E-14 | TMEM173 | 1.33E-46 | 0.537684129 | 2.70E-42 | CASP2 | 2.25E-15 | -0.277366885 | 4.57E-11 |
| LTB | 9.76E-19 | -0.350305173 | 1.98E-14 | MIR155HG | 2.28E-46 | -0.497179372 | 4.63E-42 | GLIPR1 | 2.30E-15 | 0.291777092 | 4.68E-11 |
| C1orf162 | 1.47E-18 | -0.329237275 | 2.99E-14 | KLF6 | 2.73E-46 | -0.685262829 | 5.55E-42 | TXK | 2.43E-15 | 0.29972728 | 4.94E-11 |
| WNK1 | 1.83E-18 | 0.259364628 | 3.71E-14 | NR3C1 | 3.24E-46 | -0.577725232 | 6.58E-42 | ATF7IP | 3.25E-15 | 0.259624236 | 6.61E-11 |
| SERTAD1 | 2.21E-18 | 0.284729518 | 4.50E-14 | IL16 | 3.29E-46 | 0.472075562 | 6.69E-42 | STOM | 3.30E-15 | 0.292886332 | 6.71E-11 |
| SAMHD1 | 3.20E-18 | -0.325607041 | 6.51E-14 | AHI1 | 3.90E-46 | -0.342021366 | 7.92E-42 | LAIR1 | 3.88E-15 | 0.305724953 | 7.90E-11 |
| SKAP2 | 3.28E-18 | -0.303217501 | 6.67E-14 | RACK1 | 5.79E-46 | 0.374524069 | 1.18E-41 | LINC01004 | 3.90E-15 | 0.268189845 | 7.92E-11 |
| LITAF | 3.75E-18 | -0.317938608 | 7.62E-14 | CCND2 | 1.92E-45 | -0.510847557 | 3.91E-41 | IL10RA | 5.20E-15 | -0.274575769 | 1.06E-10 |
| ELOVL5 | 5.24E-18 | 0.258828192 | 1.07E-13 | CCNH | 2.20E-45 | -0.561305564 | 4.47E-41 | NEDD9 | 7.63E-15 | 0.250256663 | 1.55E-10 |
| RASA3 | 6.07E-18 | -0.353676111 | 1.23E-13 | SAT1 | 2.47E-45 | -0.705092435 | 5.01E-41 | FCRL3 | 9.22E-15 | -0.274346388 | 1.87E-10 |
| IQGAP1 | 6.32E-18 | 0.2580348 | 1.28E-13 | SELENOK | 2.74E-45 | -0.387816707 | 5.57E-41 | APEH | 1.26E-14 | -0.264655052 | 2.56E-10 |
| CASP3 | 8.79E-18 | 0.263511832 | 1.79E-13 | PIK3R5 | 2.80E-45 | 0.422275925 | 5.69E-41 | CBLB | 1.41E-14 | 0.25205461 | 2.86E-10 |
| SIGIRR | 2.11E-17 | -0.278163304 | 4.30E-13 | TTC16 | 4.17E-45 | 0.376125749 | 8.47E-41 | P2RY10 | 1.48E-14 | 0.270174882 | 3.02E-10 |
| DSTN | 2.43E-17 | -0.37553768 | 4.93E-13 | ZFP36L2 | 4.17E-45 | -0.624191479 | 8.48E-41 | SLFN11 | 1.51E-14 | 0.256353697 | 3.06E-10 |
| MCM5 | 2.63E-17 | 0.359379904 | 5.35E-13 | CEP78 | 4.93E-45 | 0.525008079 | 1.00E-40 | PDCD1 | 1.81E-14 | -0.437871948 | 3.67E-10 |
| PTPN4 | 2.91E-17 | -0.276355579 | 5.92E-13 | RPL10A | 5.11E-45 | 0.394440119 | 1.04E-40 | STMN1 | 1.98E-14 | -0.511857492 | 4.02E-10 |
| APBA2 | 5.22E-17 | -0.354275588 | 1.06E-12 | ITGB2-AS1 | 5.55E-45 | 0.486011954 | 1.13E-40 | PARP9 | 2.27E-14 | -0.266620088 | 4.62E-10 |
| PDIA6 | 8.89E-17 | 0.256670323 | 1.81E-12 | HSPE1 | 6.23E-45 | -0.465689145 | 1.27E-40 | IFI6 | 2.91E-14 | -0.303794299 | 5.91E-10 |
| NOP53 | 1.27E-16 | -0.276484356 | 2.58E-12 | KLF3 | 7.22E-45 | 0.321156501 | 1.47E-40 | JMJD1C | 3.22E-14 | 0.255295425 | 6.55E-10 |
| PPT1 | 2.11E-16 | 0.259030108 | 4.28E-12 | PLEKHA1 | 1.04E-44 | 0.446537916 | 2.12E-40 | NFE2L2 | 3.43E-14 | 0.311435416 | 6.96E-10 |
| TUBA4A | 3.00E-16 | 0.336369377 | 6.10E-12 | GALM | 1.09E-44 | -0.483224462 | 2.22E-40 | MX1 | 7.44E-14 | -0.30285899 | 1.51E-09 |
| MSH3 | 3.35E-16 | -0.274442154 | 6.80E-12 | CRBN | 2.61E-44 | 0.545239365 | 5.29E-40 | HSPA5 | 8.09E-14 | 0.280022216 | 1.64E-09 |
| ST6GAL1 | 5.69E-16 | -0.301147286 | 1.16E-11 | ARID5A | 2.65E-44 | -0.495680911 | 5.39E-40 | DOK2 | 9.70E-14 | -0.301748456 | 1.97E-09 |
| EEF2 | 6.04E-16 | -0.266780015 | 1.23E-11 | RPL22 | 3.24E-44 | 0.274146831 | 6.59E-40 | RPS6KA1 | 1.19E-13 | -0.274441807 | 2.42E-09 |
| SAMD9L | 6.25E-16 | 0.302624111 | 1.27E-11 | ZBTB1 | 4.04E-44 | -0.504004515 | 8.21E-40 | LY6E | 1.38E-13 | -0.266584803 | 2.80E-09 |
| NOP58 | 1.09E-15 | 0.260417472 | 2.21E-11 | SURF4 | 5.09E-44 | -0.481074572 | 1.03E-39 | PLK3 | 1.55E-13 | 0.271955545 | 3.16E-09 |
| PTPN6 | 1.60E-15 | -0.306313904 | 3.26E-11 | ACTN1 | 6.37E-44 | 0.563143086 | 1.30E-39 | TOB1 | 2.85E-13 | -0.317915276 | 5.80E-09 |
| SGK1 | 1.62E-15 | 0.354055973 | 3.30E-11 | RHOC | 6.85E-44 | -0.440024157 | 1.39E-39 | PNRC1 | 2.89E-13 | 0.258689946 | 5.88E-09 |
| HSPA4 | 1.64E-15 | 0.253473523 | 3.33E-11 | CLDND1 | 2.52E-43 | -0.548837975 | 5.12E-39 | XAF1 | 3.25E-13 | -0.282166393 | 6.60E-09 |
| EHD1 | 2.10E-15 | -0.278756762 | 4.28E-11 | CLIC5 | 2.63E-43 | -0.30901854 | 5.35E-39 | BIN2 | 3.71E-13 | -0.325145222 | 7.55E-09 |
| EIF3L | 8.56E-15 | -0.336871569 | 1.74E-10 | TNFAIP8L2 | 3.21E-43 | 0.357541343 | 6.52E-39 | GIMAP2 | 4.74E-13 | -0.254029413 | 9.62E-09 |
| ABLIM1 | 1.19E-14 | -0.324343712 | 2.42E-10 | RAB3GAP1 | 3.91E-43 | -0.434339198 | 7.94E-39 | AES | 5.50E-13 | -0.288719515 | 1.12E-08 |
| STK10 | 1.30E-14 | -0.268159148 | 2.63E-10 | LAYN | 3.97E-43 | -0.503318067 | 8.06E-39 | TMF1 | 6.85E-13 | 0.268720559 | 1.39E-08 |
| PRMT2 | 4.00E-14 | -0.36578451 | 8.13E-10 | LYAR | 4.45E-43 | 0.418974795 | 9.04E-39 | FLNA | 6.96E-13 | -0.252190304 | 1.41E-08 |
| TOB1 | 1.10E-13 | 0.420601589 | 2.24E-09 | RAP1B | 1.29E-42 | 0.363754891 | 2.62E-38 | RGCC | 7.99E-13 | 0.252124937 | 1.62E-08 |
| GIMAP8 | 1.34E-13 | -0.266005502 | 2.73E-09 | MFNG | 1.73E-42 | 0.543429573 | 3.52E-38 | THEMIS2 | 8.92E-13 | -0.29950566 | 1.81E-08 |
| CRBN | 1.64E-13 | -0.313606152 | 3.34E-09 | VSIR | 2.33E-42 | 0.472841646 | 4.73E-38 | MYO1F | 1.01E-12 | -0.313771287 | 2.05E-08 |
| RASSF1 | 1.65E-13 | -0.320716421 | 3.36E-09 | GIMAP1 | 3.00E-42 | 0.45806513 | 6.10E-38 | ANXA5 | 1.04E-12 | -0.379990459 | 2.11E-08 |
| IL6ST | 1.74E-13 | 0.270246986 | 3.53E-09 | TYMP | 3.39E-42 | -0.378246939 | 6.90E-38 | KPNA2 | 1.22E-12 | 0.287940235 | 2.47E-08 |
| FOXP1 | 1.84E-13 | -0.25690001 | 3.75E-09 | GSPT1 | 3.49E-42 | -0.430730917 | 7.10E-38 | LDHB | 1.49E-12 | -0.290833795 | 3.03E-08 |
| DNAJA4 | 2.03E-13 | 0.310196606 | 4.12E-09 | HSPA8 | 6.44E-42 | -0.654235333 | 1.31E-37 | CHD1 | 1.63E-12 | 0.295984564 | 3.30E-08 |
| PCNA | 3.56E-13 | 0.271680222 | 7.24E-09 | PRKCB | 1.14E-41 | 0.452378535 | 2.31E-37 | TMEM50A | 2.28E-12 | -0.270340817 | 4.63E-08 |
| TMSB10 | 3.63E-13 | -0.275123937 | 7.37E-09 | PLK3 | 1.57E-41 | -0.544967943 | 3.19E-37 | ODC1 | 3.95E-12 | 0.318565891 | 8.02E-08 |
| ZBED2 | 4.56E-13 | 0.277366971 | 9.27E-09 | GSAP | 1.63E-41 | 0.37147057 | 3.31E-37 | CNOT6L | 4.57E-12 | 0.266167052 | 9.28E-08 |
| GIMAP1 | 6.15E-13 | -0.274719437 | 1.25E-08 | HSPA5 | 2.12E-41 | -0.576846195 | 4.30E-37 | ATM | 5.03E-12 | -0.281311337 | 1.02E-07 |
| PCMTD2 | 6.82E-13 | -0.260045338 | 1.39E-08 | RPL7A | 2.25E-41 | 0.321895603 | 4.58E-37 | NUSAP1 | 5.37E-12 | -0.259517479 | 1.09E-07 |
| PATJ | 7.02E-13 | -0.259159696 | 1.43E-08 | IDS | 3.56E-41 | -0.458663702 | 7.23E-37 | METTL3 | 5.86E-12 | -0.266892186 | 1.19E-07 |
| NEU1 | 8.43E-13 | 0.353165917 | 1.71E-08 | FLT3LG | 3.98E-41 | 0.261599768 | 8.10E-37 | CCR6 | 8.35E-12 | 0.34252327 | 1.70E-07 |
| MCUB | 8.73E-13 | -0.289979649 | 1.77E-08 | ETF1 | 5.56E-41 | -0.442451146 | 1.13E-36 | CACYBP | 1.55E-11 | -0.33464133 | 3.14E-07 |
| ZNF652 | 1.53E-12 | -0.272639274 | 3.11E-08 | TOX | 5.61E-41 | -0.504885939 | 1.14E-36 | MCM7 | 1.91E-11 | -0.384481724 | 3.89E-07 |
| GIMAP6 | 2.19E-12 | -0.325223539 | 4.45E-08 | TUBA1A | 6.12E-41 | -0.663227595 | 1.24E-36 | IKZF2 | 2.17E-11 | 0.288587786 | 4.42E-07 |
| FKBP4 | 2.73E-12 | 0.258383156 | 5.54E-08 | JMJD1C | 6.47E-41 | -0.554800553 | 1.31E-36 | SURF4 | 2.19E-11 | 0.250963201 | 4.45E-07 |
| CD52 | 3.01E-12 | -0.252058561 | 6.12E-08 | CARD10 | 6.91E-41 | 0.341082115 | 1.40E-36 | CTSC | 3.29E-11 | -0.260390505 | 6.68E-07 |
| RAP2B | 3.29E-12 | -0.270426615 | 6.68E-08 | RPS5 | 7.75E-41 | 0.443645399 | 1.58E-36 | RAB11FIP1 | 3.36E-11 | 0.295986663 | 6.84E-07 |
| THEMIS2 | 3.77E-12 | -0.309574286 | 7.66E-08 | DENND1B | 1.35E-40 | -0.4599385 | 2.75E-36 | AHR | 4.77E-11 | 0.253816843 | 9.69E-07 |
| APEX1 | 6.05E-12 | -0.254359317 | 1.23E-07 | TMEM204 | 1.72E-40 | 0.426092158 | 3.49E-36 | ITGA5 | 5.25E-11 | -0.250449641 | 1.07E-06 |
| RAB29 | 7.16E-12 | -0.327036756 | 1.45E-07 | CSF1 | 1.81E-40 | -0.475194031 | 3.68E-36 | CTSB | 5.53E-11 | -0.287299076 | 1.12E-06 |
| PLIN2 | 8.66E-12 | 0.338556839 | 1.76E-07 | CD52 | 2.59E-40 | 0.441955241 | 5.27E-36 | IER2 | 6.64E-11 | 0.313166379 | 1.35E-06 |
| PRKCA | 9.73E-12 | -0.252928017 | 1.98E-07 | GGA2 | 3.42E-40 | -0.437389805 | 6.96E-36 | CCR7 | 8.50E-11 | -0.703720521 | 1.73E-06 |
| ARL4C | 1.16E-11 | -0.261859686 | 2.36E-07 | KLRC2 | 3.43E-40 | -0.590478848 | 6.96E-36 | VAMP1 | 1.26E-10 | -0.263017613 | 2.56E-06 |
| DNAJB4 | 1.31E-11 | 0.336002187 | 2.67E-07 | TES | 4.19E-40 | 0.509853697 | 8.52E-36 | SC5D | 1.48E-10 | 0.317040055 | 3.00E-06 |
| ARHGAP45 | 3.01E-11 | -0.292182691 | 6.11E-07 | NOSIP | 4.58E-40 | 0.627473304 | 9.31E-36 | STN1 | 1.59E-10 | 0.270680508 | 3.22E-06 |
| UCP2 | 5.79E-11 | -0.294254206 | 1.18E-06 | GTF3A | 4.87E-40 | 0.43444196 | 9.89E-36 | SORL1 | 1.95E-10 | -0.378443639 | 3.96E-06 |
| MAP3K8 | 1.23E-10 | -0.300290836 | 2.50E-06 | TMIGD2 | 5.08E-40 | -0.442821244 | 1.03E-35 | GNAS | 1.96E-10 | 0.279291264 | 3.99E-06 |
| SERINC5 | 1.57E-10 | -0.346335438 | 3.19E-06 | PDE4A | 7.04E-40 | -0.349782819 | 1.43E-35 | CCDC69 | 2.90E-10 | 0.292276556 | 5.90E-06 |
| STN1 | 2.07E-10 | -0.258735984 | 4.20E-06 | NOP58 | 7.85E-40 | -0.508062928 | 1.59E-35 | MGAT4A | 4.72E-10 | 0.285364658 | 9.60E-06 |
| TTC38 | 4.03E-10 | -0.346077349 | 8.18E-06 | CD47 | 1.01E-39 | 0.412667848 | 2.06E-35 | HMGB2 | 6.54E-10 | -0.253695364 | 1.33E-05 |
| SLC9A3R1 | 9.45E-10 | -0.280214687 | 1.92E-05 | IL21R | 1.19E-39 | -0.369940898 | 2.42E-35 | ETNK1 | 1.22E-09 | -0.271106037 | 2.47E-05 |
| RGS19 | 1.58E-09 | -0.263323734 | 3.21E-05 | PIM3 | 1.36E-39 | -0.395571407 | 2.76E-35 | DPP4 | 1.49E-09 | -0.269290481 | 3.02E-05 |
| HCG18 | 9.05E-09 | -0.262342536 | 0.000183965 | CYB561 | 2.51E-39 | 0.405364778 | 5.11E-35 | CNOT8 | 1.71E-09 | -0.25487148 | 3.48E-05 |
| CHMP3 | 1.37E-08 | -0.253081116 | 0.000278749 | CD83 | 3.65E-39 | -0.790511147 | 7.43E-35 | LDLRAP1 | 2.78E-09 | -0.314823936 | 5.65E-05 |
| GLUL | 2.45E-08 | 0.277093774 | 0.000497709 | CYCS | 3.73E-39 | -0.410006127 | 7.58E-35 | CRBN | 3.44E-09 | -0.250084819 | 6.99E-05 |
| CCR7 | 2.70E-08 | -0.367171611 | 0.000547922 | RPL35A | 4.96E-39 | 0.347549932 | 1.01E-34 | RNF125 | 3.73E-09 | 0.344471286 | 7.59E-05 |
| LMNA | 3.87E-08 | 0.437171641 | 0.000786855 | TTC38 | 1.31E-38 | 0.574437884 | 2.66E-34 | AHSA1 | 3.98E-09 | -0.284490605 | 8.10E-05 |
| GPR65 | 1.34E-07 | -0.280463012 | 0.002729672 | HMGCS1 | 2.09E-38 | -0.321354137 | 4.24E-34 | PTP4A1 | 5.14E-09 | 0.262465329 | 0.000104454 |
| TUBA1B | 1.45E-07 | 0.350086175 | 0.0029512 | ID3 | 2.19E-38 | -0.391966979 | 4.45E-34 | FAM107B | 8.83E-09 | -0.276244815 | 0.000179505 |
| C4orf3 | 3.57E-07 | -0.281680203 | 0.007246895 | HCST | 2.54E-38 | 0.300823273 | 5.17E-34 | ADGRE5 | 2.49E-08 | 0.259256938 | 0.000506967 |
| XBP1 | 4.79E-07 | -0.252495711 | 0.009730835 | ARL4A | 2.90E-38 | -0.427145427 | 5.89E-34 | ARID5B | 3.22E-08 | -0.268434757 | 0.000653842 |
| NOSIP | 8.27E-07 | -0.334515056 | 0.016810368 | IL18RAP | 4.13E-38 | -0.454940876 | 8.39E-34 | SESN1 | 1.13E-07 | 0.255075937 | 0.002301555 |
| SYNE1 | 8.46E-07 | -0.291468525 | 0.017186353 | DZIP3 | 6.18E-38 | -0.425973723 | 1.26E-33 | NDFIP2 | 3.77E-07 | -0.282808531 | 0.00765475 |
| ADGRG1 | 1.09E-05 | -0.435088123 | 0.221322622 | PHTF1 | 6.34E-38 | -0.42984749 | 1.29E-33 | PHTF1 | 5.35E-06 | 0.253780874 | 0.10868156 |
| MCM7 | 1.40E-05 | 0.307103994 | 0.285220298 | S100A10 | 8.62E-38 | 0.351870356 | 1.75E-33 | LINC00674 | 7.13E-06 | 0.252877695 | 0.144993529 |
| ZNF83 | 0.000103469 | -0.265564195 | 1 | FCRL3 | 9.53E-38 | 0.486220845 | 1.94E-33 | IFNGR1 | 8.86E-05 | 0.352392485 | 1 |
| KLRC3 | 0.000770015 | -0.263808236 | 1 | CD44 | 1.28E-37 | -0.480239412 | 2.60E-33 | CRTAM | 9.40E-05 | 0.371785809 | 1 |
| ANXA1 | 0.001768208 | 0.33209233 | 1 | RLF | 1.41E-37 | -0.407235491 | 2.87E-33 | FCRL6 | 0.00017409 | -0.328935722 | 1 |
| NR4A3 | 0.00194975 | -0.37449217 | 1 | EEF1D | 2.18E-37 | 0.311291556 | 4.43E-33 | IFNG | 0.000223472 | 0.422299471 | 1 |
| KLRC2 | 0.002661168 | -0.454212006 | 1 | UBASH3B | 3.18E-37 | -0.412364551 | 6.46E-33 | INSIG1 | 0.000244559 | 0.250968151 | 1 |
| FOS | 0.002720257 | -0.431297972 | 1 | GPRIN3 | 3.40E-37 | -0.409564721 | 6.91E-33 | TRAF1 | 0.00145204 | 0.284790541 | 1 |
| NR4A2 | 0.005049365 | -0.276510718 | 1 | TRNT1 | 8.52E-37 | 0.360952683 | 1.73E-32 | HSPA1L | 0.006067948 | -0.664830459 | 1 |
| FOSB | 0.027483018 | -0.25888451 | 1 | ATP10D | 9.45E-37 | -0.318388212 | 1.92E-32 | HSPH1 | 0.012090834 | -0.417853325 | 1 |
| CD69 | 0.19187201 | -0.355995353 | 1 | MBP | 1.03E-36 | 0.465277125 | 2.10E-32 | HSPA1B | 0.946268037 | -0.615793184 | 1 |
| PTGER4 | 0.259350116 | -0.323280854 | 1 | PDCL3 | 1.26E-36 | -0.30958557 | 2.55E-32 |  |  |  |  |
|  |  |  |  | HLA-DRA | 1.39E-36 | -0.718293062 | 2.82E-32 |  |  |  |  |
|  |  |  |  | ITGA6 | 2.07E-36 | 0.30953119 | 4.22E-32 |  |  |  |  |
|  |  |  |  | MFSD11 | 2.12E-36 | -0.376541917 | 4.32E-32 |  |  |  |  |
|  |  |  |  | GADD45B | 2.50E-36 | -0.453639462 | 5.08E-32 |  |  |  |  |
|  |  |  |  | A2M-AS1 | 3.73E-36 | 0.37281534 | 7.58E-32 |  |  |  |  |
|  |  |  |  | EIF1 | 4.79E-36 | -0.3925185 | 9.74E-32 |  |  |  |  |
|  |  |  |  | CCR7 | 5.42E-36 | 0.871461511 | 1.10E-31 |  |  |  |  |
|  |  |  |  | DNAJB9 | 6.27E-36 | -0.527035482 | 1.27E-31 |  |  |  |  |
|  |  |  |  | FXYD5 | 6.94E-36 | 0.351819906 | 1.41E-31 |  |  |  |  |
|  |  |  |  | TNF | 7.24E-36 | -0.81007301 | 1.47E-31 |  |  |  |  |
|  |  |  |  | ATP1B3 | 7.87E-36 | -0.47427395 | 1.60E-31 |  |  |  |  |
|  |  |  |  | CD8A | 7.90E-36 | -0.2690796 | 1.61E-31 |  |  |  |  |
|  |  |  |  | PIK3R1 | 8.43E-36 | -0.678003783 | 1.71E-31 |  |  |  |  |
|  |  |  |  | AGTPBP1 | 8.59E-36 | 0.38762341 | 1.75E-31 |  |  |  |  |
|  |  |  |  | CTLA4 | 9.55E-36 | -0.585817889 | 1.94E-31 |  |  |  |  |
|  |  |  |  | SYAP1 | 1.24E-35 | -0.342031749 | 2.52E-31 |  |  |  |  |
|  |  |  |  | AUTS2 | 1.96E-35 | -0.296707771 | 3.99E-31 |  |  |  |  |
|  |  |  |  | SAMHD1 | 2.39E-35 | 0.445102586 | 4.87E-31 |  |  |  |  |
|  |  |  |  | SNX9 | 2.53E-35 | -0.343010169 | 5.14E-31 |  |  |  |  |
|  |  |  |  | ARHGAP18 | 2.82E-35 | -0.333106054 | 5.73E-31 |  |  |  |  |
|  |  |  |  | PAFAH2 | 4.21E-35 | 0.302634265 | 8.55E-31 |  |  |  |  |
|  |  |  |  | LY9 | 4.94E-35 | 0.379335465 | 1.00E-30 |  |  |  |  |
|  |  |  |  | TIGIT | 1.38E-34 | -0.483252833 | 2.81E-30 |  |  |  |  |
|  |  |  |  | EVI2A | 1.83E-34 | -0.425341615 | 3.72E-30 |  |  |  |  |
|  |  |  |  | SERPINB6 | 2.09E-34 | 0.407689027 | 4.26E-30 |  |  |  |  |
|  |  |  |  | SEL1L3 | 2.47E-34 | -0.399098469 | 5.03E-30 |  |  |  |  |
|  |  |  |  | PPP1CB | 2.73E-34 | -0.472896646 | 5.54E-30 |  |  |  |  |
|  |  |  |  | NASP | 2.84E-34 | -0.41258404 | 5.77E-30 |  |  |  |  |
|  |  |  |  | IL2RB | 3.16E-34 | -0.422571944 | 6.42E-30 |  |  |  |  |
|  |  |  |  | EIF5 | 3.46E-34 | -0.409458072 | 7.03E-30 |  |  |  |  |
|  |  |  |  | CTSD | 4.25E-34 | -0.43853713 | 8.64E-30 |  |  |  |  |
|  |  |  |  | HMGN3 | 5.21E-34 | 0.432958116 | 1.06E-29 |  |  |  |  |
|  |  |  |  | CCR5 | 6.29E-34 | -0.398335609 | 1.28E-29 |  |  |  |  |
|  |  |  |  | HMGB2 | 6.50E-34 | -0.491920305 | 1.32E-29 |  |  |  |  |
|  |  |  |  | GZMA | 8.36E-34 | -0.562429988 | 1.70E-29 |  |  |  |  |
|  |  |  |  | RAB8B | 9.38E-34 | -0.380567436 | 1.91E-29 |  |  |  |  |
|  |  |  |  | SLC3A2 | 1.10E-33 | -0.419132749 | 2.24E-29 |  |  |  |  |
|  |  |  |  | LEPROTL1 | 1.31E-33 | -0.335047933 | 2.65E-29 |  |  |  |  |
|  |  |  |  | PERP | 1.60E-33 | -0.4049485 | 3.25E-29 |  |  |  |  |
|  |  |  |  | RPS6KA1 | 1.61E-33 | 0.476006648 | 3.26E-29 |  |  |  |  |
|  |  |  |  | TRAT1 | 1.63E-33 | -0.452008447 | 3.32E-29 |  |  |  |  |
|  |  |  |  | PLSCR1 | 1.65E-33 | -0.412348778 | 3.35E-29 |  |  |  |  |
|  |  |  |  | CALM1 | 2.11E-33 | 0.265169139 | 4.29E-29 |  |  |  |  |
|  |  |  |  | LPIN1 | 2.69E-33 | -0.34134133 | 5.46E-29 |  |  |  |  |
|  |  |  |  | CKLF | 3.07E-33 | -0.256869759 | 6.23E-29 |  |  |  |  |
|  |  |  |  | PCED1B | 3.08E-33 | 0.253309398 | 6.26E-29 |  |  |  |  |
|  |  |  |  | MAL | 3.42E-33 | 0.33264953 | 6.95E-29 |  |  |  |  |
|  |  |  |  | CD63 | 4.04E-33 | -0.432774536 | 8.22E-29 |  |  |  |  |
|  |  |  |  | PCMTD2 | 4.28E-33 | 0.441601823 | 8.70E-29 |  |  |  |  |
|  |  |  |  | TC2N | 5.14E-33 | 0.340797753 | 1.04E-28 |  |  |  |  |
|  |  |  |  | SGMS1 | 6.32E-33 | -0.385413707 | 1.28E-28 |  |  |  |  |
|  |  |  |  | CCL5 | 6.74E-33 | -0.324920313 | 1.37E-28 |  |  |  |  |
|  |  |  |  | IER5 | 7.20E-33 | -0.255663373 | 1.46E-28 |  |  |  |  |
|  |  |  |  | PLEKHA2 | 1.07E-32 | -0.258600339 | 2.18E-28 |  |  |  |  |
|  |  |  |  | SPON2 | 1.32E-32 | 0.522568023 | 2.69E-28 |  |  |  |  |
|  |  |  |  | RPL23 | 1.49E-32 | 0.314918637 | 3.04E-28 |  |  |  |  |
|  |  |  |  | UBXN11 | 1.58E-32 | 0.321165697 | 3.22E-28 |  |  |  |  |
|  |  |  |  | SIGIRR | 1.94E-32 | 0.367797899 | 3.95E-28 |  |  |  |  |
|  |  |  |  | SLC9A3R1 | 2.44E-32 | 0.454816985 | 4.95E-28 |  |  |  |  |
|  |  |  |  | ITGB1 | 2.89E-32 | 0.549019189 | 5.87E-28 |  |  |  |  |
|  |  |  |  | MAP3K8 | 3.25E-32 | -0.71059215 | 6.60E-28 |  |  |  |  |
|  |  |  |  | LINC00963 | 3.36E-32 | -0.311557784 | 6.82E-28 |  |  |  |  |
|  |  |  |  | LIMS1 | 3.75E-32 | -0.373282717 | 7.63E-28 |  |  |  |  |
|  |  |  |  | CTSW | 3.85E-32 | 0.333454273 | 7.83E-28 |  |  |  |  |
|  |  |  |  | SOCS3 | 4.74E-32 | -0.484085867 | 9.64E-28 |  |  |  |  |
|  |  |  |  | B3GNT2 | 5.03E-32 | -0.412218078 | 1.02E-27 |  |  |  |  |
|  |  |  |  | NUSAP1 | 5.20E-32 | -0.326776023 | 1.06E-27 |  |  |  |  |
|  |  |  |  | HSPE1-MOB4 | 7.55E-32 | -0.348946963 | 1.53E-27 |  |  |  |  |
|  |  |  |  | MCM6 | 9.50E-32 | -0.396096832 | 1.93E-27 |  |  |  |  |
|  |  |  |  | CHD1 | 1.00E-31 | -0.39433868 | 2.04E-27 |  |  |  |  |
|  |  |  |  | SFMBT2 | 1.04E-31 | -0.291359181 | 2.12E-27 |  |  |  |  |
|  |  |  |  | MAP4 | 1.23E-31 | -0.342412702 | 2.49E-27 |  |  |  |  |
|  |  |  |  | ITPRIP | 1.31E-31 | -0.453822502 | 2.66E-27 |  |  |  |  |
|  |  |  |  | RPS16 | 2.14E-31 | 0.282013269 | 4.36E-27 |  |  |  |  |
|  |  |  |  | RPS11 | 2.42E-31 | 0.296373528 | 4.92E-27 |  |  |  |  |
|  |  |  |  | CKAP2 | 2.61E-31 | -0.307139256 | 5.30E-27 |  |  |  |  |
|  |  |  |  | CMC1 | 2.62E-31 | 0.34081521 | 5.32E-27 |  |  |  |  |
|  |  |  |  | JOSD1 | 2.99E-31 | -0.256163261 | 6.09E-27 |  |  |  |  |
|  |  |  |  | IL10RA | 3.27E-31 | 0.426039377 | 6.64E-27 |  |  |  |  |
|  |  |  |  | CYTH4 | 4.05E-31 | 0.406703848 | 8.24E-27 |  |  |  |  |
|  |  |  |  | C16orf54 | 4.95E-31 | 0.319480423 | 1.01E-26 |  |  |  |  |
|  |  |  |  | TOB2 | 5.57E-31 | -0.350289228 | 1.13E-26 |  |  |  |  |
|  |  |  |  | IL12RB2 | 5.64E-31 | -0.336650766 | 1.15E-26 |  |  |  |  |
|  |  |  |  | NLRP1 | 7.52E-31 | 0.419088894 | 1.53E-26 |  |  |  |  |
|  |  |  |  | SERF2 | 8.44E-31 | 0.256400811 | 1.72E-26 |  |  |  |  |
|  |  |  |  | NAA50 | 9.23E-31 | -0.30870265 | 1.88E-26 |  |  |  |  |
|  |  |  |  | METTL3 | 1.51E-30 | 0.419368919 | 3.08E-26 |  |  |  |  |
|  |  |  |  | TPI1 | 1.53E-30 | -0.400465935 | 3.11E-26 |  |  |  |  |
|  |  |  |  | NKG7 | 1.85E-30 | 0.476022602 | 3.77E-26 |  |  |  |  |
|  |  |  |  | RAB11FIP1 | 1.89E-30 | -0.39048656 | 3.84E-26 |  |  |  |  |
|  |  |  |  | PPP1R15B | 2.08E-30 | -0.377868902 | 4.23E-26 |  |  |  |  |
|  |  |  |  | RCBTB2 | 3.12E-30 | 0.457342472 | 6.33E-26 |  |  |  |  |
|  |  |  |  | GIMAP6 | 3.23E-30 | 0.517778527 | 6.55E-26 |  |  |  |  |
|  |  |  |  | OASL | 3.42E-30 | -0.46907281 | 6.95E-26 |  |  |  |  |
|  |  |  |  | LTB | 3.52E-30 | 0.449222743 | 7.16E-26 |  |  |  |  |
|  |  |  |  | DENND2D | 3.94E-30 | 0.359626721 | 8.00E-26 |  |  |  |  |
|  |  |  |  | FUT11 | 4.91E-30 | 0.335465572 | 9.97E-26 |  |  |  |  |
|  |  |  |  | CD101 | 6.72E-30 | -0.364826783 | 1.37E-25 |  |  |  |  |
|  |  |  |  | WNK1 | 9.12E-30 | -0.388061797 | 1.85E-25 |  |  |  |  |
|  |  |  |  | DSTN | 1.51E-29 | 0.481159729 | 3.07E-25 |  |  |  |  |
|  |  |  |  | ARF4 | 1.65E-29 | -0.405812668 | 3.36E-25 |  |  |  |  |
|  |  |  |  | ANKRD37 | 2.20E-29 | -0.348201548 | 4.47E-25 |  |  |  |  |
|  |  |  |  | MFSD14A | 2.68E-29 | -0.320239899 | 5.44E-25 |  |  |  |  |
|  |  |  |  | ZBTB21 | 3.11E-29 | -0.284082474 | 6.32E-25 |  |  |  |  |
|  |  |  |  | SLF1 | 3.64E-29 | -0.301617935 | 7.40E-25 |  |  |  |  |
|  |  |  |  | ASAP1 | 5.53E-29 | -0.26855708 | 1.12E-24 |  |  |  |  |
|  |  |  |  | SFPQ | 6.92E-29 | -0.274581235 | 1.41E-24 |  |  |  |  |
|  |  |  |  | ZNF10 | 7.91E-29 | -0.272601626 | 1.61E-24 |  |  |  |  |
|  |  |  |  | AKIRIN2 | 1.29E-28 | -0.369501761 | 2.62E-24 |  |  |  |  |
|  |  |  |  | HIF1A-AS2 | 1.29E-28 | -0.305632614 | 2.62E-24 |  |  |  |  |
|  |  |  |  | RBL2 | 1.48E-28 | 0.346340655 | 3.01E-24 |  |  |  |  |
|  |  |  |  | MCUB | 1.82E-28 | 0.397845668 | 3.69E-24 |  |  |  |  |
|  |  |  |  | HLA-DMA | 2.21E-28 | -0.424464015 | 4.50E-24 |  |  |  |  |
|  |  |  |  | NFE2L2 | 2.35E-28 | -0.41240919 | 4.77E-24 |  |  |  |  |
|  |  |  |  | LYST | 2.39E-28 | -0.485691404 | 4.87E-24 |  |  |  |  |
|  |  |  |  | FLOT2 | 2.51E-28 | 0.349450167 | 5.10E-24 |  |  |  |  |
|  |  |  |  | HEATR5B | 2.51E-28 | 0.262412911 | 5.11E-24 |  |  |  |  |
|  |  |  |  | AMD1 | 2.63E-28 | -0.444441426 | 5.34E-24 |  |  |  |  |
|  |  |  |  | FAM160B1 | 3.66E-28 | -0.332583622 | 7.43E-24 |  |  |  |  |
|  |  |  |  | SMIM14 | 3.74E-28 | 0.318992749 | 7.59E-24 |  |  |  |  |
|  |  |  |  | DNAJB4 | 4.61E-28 | -0.422467296 | 9.38E-24 |  |  |  |  |
|  |  |  |  | ATXN1 | 4.78E-28 | -0.27364198 | 9.72E-24 |  |  |  |  |
|  |  |  |  | MORF4L2 | 6.23E-28 | -0.346124161 | 1.27E-23 |  |  |  |  |
|  |  |  |  | ELOVL5 | 8.12E-28 | -0.353410894 | 1.65E-23 |  |  |  |  |
|  |  |  |  | LAT | 1.33E-27 | 0.322056166 | 2.70E-23 |  |  |  |  |
|  |  |  |  | LAT2 | 1.37E-27 | -0.378330996 | 2.78E-23 |  |  |  |  |
|  |  |  |  | DENND4A | 1.45E-27 | -0.361249562 | 2.96E-23 |  |  |  |  |
|  |  |  |  | CCDC141 | 2.08E-27 | -0.451410357 | 4.22E-23 |  |  |  |  |
|  |  |  |  | KLRC3 | 2.22E-27 | -0.369791455 | 4.51E-23 |  |  |  |  |
|  |  |  |  | NLRC5 | 2.49E-27 | 0.343996676 | 5.06E-23 |  |  |  |  |
|  |  |  |  | RAD51-AS1 | 2.57E-27 | 0.259224759 | 5.22E-23 |  |  |  |  |
|  |  |  |  | GNAS | 2.63E-27 | -0.386414827 | 5.34E-23 |  |  |  |  |
|  |  |  |  | SMC4 | 3.64E-27 | -0.376190571 | 7.40E-23 |  |  |  |  |
|  |  |  |  | MYC | 4.15E-27 | 0.461678703 | 8.44E-23 |  |  |  |  |
|  |  |  |  | CCNL1 | 4.23E-27 | -0.338051016 | 8.60E-23 |  |  |  |  |
|  |  |  |  | NUP98 | 4.40E-27 | -0.346941962 | 8.93E-23 |  |  |  |  |
|  |  |  |  | ARHGAP1 | 4.40E-27 | 0.369524396 | 8.94E-23 |  |  |  |  |
|  |  |  |  | PNISR | 6.49E-27 | 0.320420579 | 1.32E-22 |  |  |  |  |
|  |  |  |  | SLFN11 | 6.70E-27 | -0.350436821 | 1.36E-22 |  |  |  |  |
|  |  |  |  | USP28 | 6.79E-27 | 0.321669735 | 1.38E-22 |  |  |  |  |
|  |  |  |  | SYTL2 | 1.15E-26 | -0.325305329 | 2.35E-22 |  |  |  |  |
|  |  |  |  | ZNF683 | 1.27E-26 | -0.497576438 | 2.58E-22 |  |  |  |  |
|  |  |  |  | SUSD6 | 1.37E-26 | -0.327741498 | 2.79E-22 |  |  |  |  |
|  |  |  |  | SARAF | 1.38E-26 | -0.353195959 | 2.81E-22 |  |  |  |  |
|  |  |  |  | OTULIN | 1.75E-26 | -0.271649333 | 3.55E-22 |  |  |  |  |
|  |  |  |  | ARL4C | 2.07E-26 | 0.327058289 | 4.21E-22 |  |  |  |  |
|  |  |  |  | WDR47 | 2.24E-26 | -0.288235052 | 4.56E-22 |  |  |  |  |
|  |  |  |  | TBL1XR1 | 2.29E-26 | -0.353876767 | 4.65E-22 |  |  |  |  |
|  |  |  |  | UBB | 2.44E-26 | -0.291852071 | 4.95E-22 |  |  |  |  |
|  |  |  |  | CD58 | 2.59E-26 | -0.31328931 | 5.27E-22 |  |  |  |  |
|  |  |  |  | SRSF3 | 4.45E-26 | -0.352341486 | 9.04E-22 |  |  |  |  |
|  |  |  |  | NOP53 | 4.88E-26 | 0.375180084 | 9.91E-22 |  |  |  |  |
|  |  |  |  | EZH2 | 6.31E-26 | -0.264064906 | 1.28E-21 |  |  |  |  |
|  |  |  |  | GOLGB1 | 9.28E-26 | -0.307367577 | 1.89E-21 |  |  |  |  |
|  |  |  |  | PLEKHB1 | 1.04E-25 | 0.331211631 | 2.10E-21 |  |  |  |  |
|  |  |  |  | EML4 | 1.43E-25 | -0.332386516 | 2.90E-21 |  |  |  |  |
|  |  |  |  | TUBA4A | 1.43E-25 | -0.589592508 | 2.90E-21 |  |  |  |  |
|  |  |  |  | MYO1G | 1.55E-25 | 0.427382506 | 3.15E-21 |  |  |  |  |
|  |  |  |  | HLA-DMB | 2.16E-25 | -0.355298732 | 4.38E-21 |  |  |  |  |
|  |  |  |  | RCSD1 | 2.42E-25 | 0.349321776 | 4.92E-21 |  |  |  |  |
|  |  |  |  | CAPN2 | 2.73E-25 | 0.392322619 | 5.55E-21 |  |  |  |  |
|  |  |  |  | PAG1 | 3.17E-25 | -0.349099442 | 6.44E-21 |  |  |  |  |
|  |  |  |  | CDK6 | 3.26E-25 | -0.33931542 | 6.62E-21 |  |  |  |  |
|  |  |  |  | CHORDC1 | 4.81E-25 | -0.423316111 | 9.78E-21 |  |  |  |  |
|  |  |  |  | HLA-DRB1 | 6.11E-25 | -0.547478848 | 1.24E-20 |  |  |  |  |
|  |  |  |  | LDLR | 6.51E-25 | -0.359496831 | 1.32E-20 |  |  |  |  |
|  |  |  |  | CNN2 | 7.28E-25 | 0.348362262 | 1.48E-20 |  |  |  |  |
|  |  |  |  | ING4 | 7.85E-25 | 0.32289339 | 1.60E-20 |  |  |  |  |
|  |  |  |  | FAM102A | 8.19E-25 | -0.256031534 | 1.66E-20 |  |  |  |  |
|  |  |  |  | TPST2 | 8.74E-25 | 0.425382869 | 1.78E-20 |  |  |  |  |
|  |  |  |  | PDLIM7 | 9.62E-25 | -0.260293276 | 1.95E-20 |  |  |  |  |
|  |  |  |  | PNPLA8 | 9.88E-25 | -0.287595312 | 2.01E-20 |  |  |  |  |
|  |  |  |  | STX11 | 1.12E-24 | -0.338813117 | 2.27E-20 |  |  |  |  |
|  |  |  |  | USP36 | 1.23E-24 | -0.383375912 | 2.51E-20 |  |  |  |  |
|  |  |  |  | RAB27A | 1.38E-24 | -0.299872185 | 2.81E-20 |  |  |  |  |
|  |  |  |  | RGS19 | 1.40E-24 | 0.379319198 | 2.84E-20 |  |  |  |  |
|  |  |  |  | SMIM3 | 1.48E-24 | -0.396331144 | 3.00E-20 |  |  |  |  |
|  |  |  |  | KIF2A | 1.67E-24 | 0.266692152 | 3.39E-20 |  |  |  |  |
|  |  |  |  | NACA | 1.70E-24 | 0.250554115 | 3.45E-20 |  |  |  |  |
|  |  |  |  | ANXA5 | 2.11E-24 | -0.394195055 | 4.28E-20 |  |  |  |  |
|  |  |  |  | ICAM1 | 2.38E-24 | -0.39220634 | 4.83E-20 |  |  |  |  |
|  |  |  |  | DUSP6 | 2.48E-24 | -0.371460215 | 5.05E-20 |  |  |  |  |
|  |  |  |  | DDIT3 | 2.88E-24 | -0.334562008 | 5.86E-20 |  |  |  |  |
|  |  |  |  | DIP2A | 3.30E-24 | 0.327719428 | 6.71E-20 |  |  |  |  |
|  |  |  |  | BBS2 | 4.10E-24 | 0.340286355 | 8.33E-20 |  |  |  |  |
|  |  |  |  | RNF139 | 4.60E-24 | -0.395573503 | 9.35E-20 |  |  |  |  |
|  |  |  |  | STK10 | 4.79E-24 | 0.329375646 | 9.74E-20 |  |  |  |  |
|  |  |  |  | TNFRSF18 | 4.96E-24 | -0.367512053 | 1.01E-19 |  |  |  |  |
|  |  |  |  | HIPK1 | 6.02E-24 | -0.327339635 | 1.22E-19 |  |  |  |  |
|  |  |  |  | CYP51A1 | 6.32E-24 | -0.267095891 | 1.28E-19 |  |  |  |  |
|  |  |  |  | ITGAL | 7.20E-24 | 0.351080916 | 1.46E-19 |  |  |  |  |
|  |  |  |  | HLA-DQB1 | 1.10E-23 | -0.524980269 | 2.24E-19 |  |  |  |  |
|  |  |  |  | BRE-AS1 | 1.18E-23 | -0.509198858 | 2.40E-19 |  |  |  |  |
|  |  |  |  | RASSF1 | 1.22E-23 | 0.384634397 | 2.47E-19 |  |  |  |  |
|  |  |  |  | TUBA1C | 2.28E-23 | -0.299353543 | 4.63E-19 |  |  |  |  |
|  |  |  |  | ZBED2 | 2.28E-23 | -0.311225343 | 4.63E-19 |  |  |  |  |
|  |  |  |  | BCL6 | 2.62E-23 | -0.295982282 | 5.33E-19 |  |  |  |  |
|  |  |  |  | SNHG1 | 2.80E-23 | -0.336183719 | 5.69E-19 |  |  |  |  |
|  |  |  |  | JMY | 3.26E-23 | -0.308938406 | 6.62E-19 |  |  |  |  |
|  |  |  |  | EFHD2 | 3.31E-23 | 0.303513681 | 6.73E-19 |  |  |  |  |
|  |  |  |  | CXCR3 | 3.41E-23 | -0.332647811 | 6.93E-19 |  |  |  |  |
|  |  |  |  | BTN3A3 | 3.43E-23 | 0.307021912 | 6.97E-19 |  |  |  |  |
|  |  |  |  | SSH1 | 3.85E-23 | -0.253518183 | 7.82E-19 |  |  |  |  |
|  |  |  |  | DENND2C | 4.20E-23 | -0.290088824 | 8.53E-19 |  |  |  |  |
|  |  |  |  | SH2D3C | 5.19E-23 | 0.331200438 | 1.05E-18 |  |  |  |  |
|  |  |  |  | GPR65 | 5.65E-23 | -0.490779948 | 1.15E-18 |  |  |  |  |
|  |  |  |  | NPRL2 | 5.83E-23 | 0.362649813 | 1.18E-18 |  |  |  |  |
|  |  |  |  | MORC2-AS1 | 6.22E-23 | 0.3928738 | 1.26E-18 |  |  |  |  |
|  |  |  |  | KDM5B | 6.70E-23 | -0.306885853 | 1.36E-18 |  |  |  |  |
|  |  |  |  | LY75 | 8.17E-23 | 0.36295265 | 1.66E-18 |  |  |  |  |
|  |  |  |  | CCR1 | 8.38E-23 | -0.402414714 | 1.70E-18 |  |  |  |  |
|  |  |  |  | ARHGEF7 | 1.12E-22 | -0.26194572 | 2.27E-18 |  |  |  |  |
|  |  |  |  | EPB41 | 1.22E-22 | 0.281126722 | 2.48E-18 |  |  |  |  |
|  |  |  |  | UBE2A | 1.32E-22 | -0.252178467 | 2.69E-18 |  |  |  |  |
|  |  |  |  | PLP2 | 2.14E-22 | -0.26098043 | 4.34E-18 |  |  |  |  |
|  |  |  |  | GYG1 | 2.17E-22 | -0.331091886 | 4.40E-18 |  |  |  |  |
|  |  |  |  | IFITM2 | 2.35E-22 | 0.285570389 | 4.78E-18 |  |  |  |  |
|  |  |  |  | DTHD1 | 2.42E-22 | -0.322252501 | 4.92E-18 |  |  |  |  |
|  |  |  |  | ASXL2 | 2.51E-22 | -0.297793651 | 5.11E-18 |  |  |  |  |
|  |  |  |  | TMEM123 | 2.69E-22 | 0.449167868 | 5.46E-18 |  |  |  |  |
|  |  |  |  | INSIG1 | 2.69E-22 | -0.411339372 | 5.46E-18 |  |  |  |  |
|  |  |  |  | EIF2S3 | 3.05E-22 | 0.312632203 | 6.20E-18 |  |  |  |  |
|  |  |  |  | BTN3A2 | 3.19E-22 | 0.271866576 | 6.48E-18 |  |  |  |  |
|  |  |  |  | DAXX | 3.24E-22 | 0.344530349 | 6.59E-18 |  |  |  |  |
|  |  |  |  | GLG1 | 3.40E-22 | 0.308627528 | 6.91E-18 |  |  |  |  |
|  |  |  |  | BCAS2 | 3.50E-22 | -0.342746472 | 7.11E-18 |  |  |  |  |
|  |  |  |  | SLC44A2 | 4.22E-22 | 0.367495703 | 8.59E-18 |  |  |  |  |
|  |  |  |  | ZNF302 | 4.49E-22 | 0.271537621 | 9.13E-18 |  |  |  |  |
|  |  |  |  | BTN3A1 | 4.84E-22 | 0.260611058 | 9.84E-18 |  |  |  |  |
|  |  |  |  | HPGD | 7.23E-22 | -0.308273704 | 1.47E-17 |  |  |  |  |
|  |  |  |  | CD2 | 7.49E-22 | -0.316860614 | 1.52E-17 |  |  |  |  |
|  |  |  |  | GIMAP8 | 9.29E-22 | 0.339024649 | 1.89E-17 |  |  |  |  |
|  |  |  |  | CALCOCO1 | 9.56E-22 | 0.318764892 | 1.94E-17 |  |  |  |  |
|  |  |  |  | SLC25A5 | 1.19E-21 | -0.332072308 | 2.42E-17 |  |  |  |  |
|  |  |  |  | PTPN7 | 1.20E-21 | -0.356347973 | 2.44E-17 |  |  |  |  |
|  |  |  |  | FAM107B | 1.20E-21 | 0.392741521 | 2.44E-17 |  |  |  |  |
|  |  |  |  | TRAF3 | 1.21E-21 | -0.258414227 | 2.45E-17 |  |  |  |  |
|  |  |  |  | LPAR6 | 1.58E-21 | 0.356477035 | 3.22E-17 |  |  |  |  |
|  |  |  |  | SPTY2D1 | 2.11E-21 | -0.272189714 | 4.29E-17 |  |  |  |  |
|  |  |  |  | PLXDC1 | 2.11E-21 | -0.317530954 | 4.29E-17 |  |  |  |  |
|  |  |  |  | ORMDL1 | 2.18E-21 | 0.374434101 | 4.42E-17 |  |  |  |  |
|  |  |  |  | DDX24 | 2.44E-21 | -0.262097465 | 4.97E-17 |  |  |  |  |
|  |  |  |  | MTFP1 | 3.69E-21 | -0.257390019 | 7.50E-17 |  |  |  |  |
|  |  |  |  | ZNF276 | 4.40E-21 | 0.386897365 | 8.94E-17 |  |  |  |  |
|  |  |  |  | IQGAP1 | 6.13E-21 | -0.328319587 | 1.25E-16 |  |  |  |  |
|  |  |  |  | COQ10B | 7.66E-21 | -0.285770987 | 1.56E-16 |  |  |  |  |
|  |  |  |  | RPA2 | 7.69E-21 | 0.393235276 | 1.56E-16 |  |  |  |  |
|  |  |  |  | ACOT7 | 7.88E-21 | -0.270387323 | 1.60E-16 |  |  |  |  |
|  |  |  |  | NFATC3 | 1.23E-20 | 0.330106052 | 2.50E-16 |  |  |  |  |
|  |  |  |  | SASH3 | 1.57E-20 | 0.310467363 | 3.20E-16 |  |  |  |  |
|  |  |  |  | CHMP3 | 1.67E-20 | 0.370327293 | 3.40E-16 |  |  |  |  |
|  |  |  |  | EIF4B | 1.78E-20 | 0.323537396 | 3.61E-16 |  |  |  |  |
|  |  |  |  | YWHAB | 1.90E-20 | 0.250839885 | 3.87E-16 |  |  |  |  |
|  |  |  |  | MOB3A | 1.92E-20 | 0.334992849 | 3.90E-16 |  |  |  |  |
|  |  |  |  | HLA-DRB6 | 2.17E-20 | -0.434013221 | 4.41E-16 |  |  |  |  |
|  |  |  |  | FKBP1A | 2.18E-20 | -0.340883199 | 4.43E-16 |  |  |  |  |
|  |  |  |  | POLR2A | 2.22E-20 | -0.303497916 | 4.51E-16 |  |  |  |  |
|  |  |  |  | GLRX | 3.03E-20 | 0.30513018 | 6.16E-16 |  |  |  |  |
|  |  |  |  | RASA1 | 3.70E-20 | -0.257747019 | 7.52E-16 |  |  |  |  |
|  |  |  |  | STK17A | 3.88E-20 | -0.277681501 | 7.88E-16 |  |  |  |  |
|  |  |  |  | PTPN4 | 4.71E-20 | 0.30029408 | 9.58E-16 |  |  |  |  |
|  |  |  |  | GEMIN7 | 5.22E-20 | 0.282426346 | 1.06E-15 |  |  |  |  |
|  |  |  |  | LINC01550 | 6.25E-20 | 0.315905359 | 1.27E-15 |  |  |  |  |
|  |  |  |  | IDH2 | 7.73E-20 | -0.319814013 | 1.57E-15 |  |  |  |  |
|  |  |  |  | COQ8A | 8.25E-20 | 0.29118281 | 1.68E-15 |  |  |  |  |
|  |  |  |  | OAT | 8.47E-20 | -0.285639137 | 1.72E-15 |  |  |  |  |
|  |  |  |  | PDK1 | 8.77E-20 | 0.291354005 | 1.78E-15 |  |  |  |  |
|  |  |  |  | ZNF267 | 1.03E-19 | -0.32723913 | 2.10E-15 |  |  |  |  |
|  |  |  |  | ATF7IP | 1.07E-19 | -0.337527116 | 2.17E-15 |  |  |  |  |
|  |  |  |  | APOBEC3C | 1.36E-19 | -0.427579833 | 2.76E-15 |  |  |  |  |
|  |  |  |  | RHBDD2 | 1.39E-19 | -0.302737068 | 2.83E-15 |  |  |  |  |
|  |  |  |  | TP53INP1 | 1.94E-19 | -0.256465404 | 3.94E-15 |  |  |  |  |
|  |  |  |  | EIF3F | 1.95E-19 | 0.266575235 | 3.96E-15 |  |  |  |  |
|  |  |  |  | LDHB | 2.12E-19 | 0.445179415 | 4.32E-15 |  |  |  |  |
|  |  |  |  | MRFAP1L1 | 2.35E-19 | 0.3567752 | 4.77E-15 |  |  |  |  |
|  |  |  |  | SELPLG | 2.61E-19 | 0.252707013 | 5.30E-15 |  |  |  |  |
|  |  |  |  | SLC25A45 | 3.02E-19 | 0.293297799 | 6.14E-15 |  |  |  |  |
|  |  |  |  | PRKCQ-AS1 | 3.13E-19 | 0.333563496 | 6.37E-15 |  |  |  |  |
|  |  |  |  | LY75-CD302 | 3.57E-19 | 0.338032019 | 7.25E-15 |  |  |  |  |
|  |  |  |  | APOL2 | 4.22E-19 | 0.274290744 | 8.58E-15 |  |  |  |  |
|  |  |  |  | GIMAP2 | 4.57E-19 | 0.337854241 | 9.28E-15 |  |  |  |  |
|  |  |  |  | MAPK1IP1L | 4.76E-19 | -0.292553239 | 9.67E-15 |  |  |  |  |
|  |  |  |  | H2AFZ | 5.06E-19 | -0.347219593 | 1.03E-14 |  |  |  |  |
|  |  |  |  | VIM | 5.24E-19 | -0.357282561 | 1.06E-14 |  |  |  |  |
|  |  |  |  | TMEM14C | 7.25E-19 | -0.261548446 | 1.47E-14 |  |  |  |  |
|  |  |  |  | LPCAT1 | 7.35E-19 | 0.250018988 | 1.49E-14 |  |  |  |  |
|  |  |  |  | SDR39U1 | 7.90E-19 | 0.340328108 | 1.61E-14 |  |  |  |  |
|  |  |  |  | FTH1 | 9.53E-19 | -0.409667523 | 1.94E-14 |  |  |  |  |
|  |  |  |  | PDE4DIP | 1.05E-18 | -0.419733757 | 2.14E-14 |  |  |  |  |
|  |  |  |  | CASP3 | 1.08E-18 | -0.299926594 | 2.19E-14 |  |  |  |  |
|  |  |  |  | EIF3L | 1.22E-18 | 0.424274365 | 2.48E-14 |  |  |  |  |
|  |  |  |  | SLC6A6 | 1.32E-18 | -0.251792671 | 2.68E-14 |  |  |  |  |
|  |  |  |  | GPR18 | 1.35E-18 | -0.403123934 | 2.73E-14 |  |  |  |  |
|  |  |  |  | WSB1 | 1.53E-18 | -0.323225694 | 3.11E-14 |  |  |  |  |
|  |  |  |  | ZFP36L1 | 1.75E-18 | -0.386529583 | 3.55E-14 |  |  |  |  |
|  |  |  |  | HLA-DRB5 | 2.45E-18 | -0.408671679 | 4.99E-14 |  |  |  |  |
|  |  |  |  | PPP1CA | 3.18E-18 | 0.285137265 | 6.45E-14 |  |  |  |  |
|  |  |  |  | PDCD4 | 3.72E-18 | -0.306636585 | 7.56E-14 |  |  |  |  |
|  |  |  |  | FYN | 4.04E-18 | -0.250406695 | 8.20E-14 |  |  |  |  |
|  |  |  |  | FRMD4B | 4.50E-18 | -0.325783795 | 9.14E-14 |  |  |  |  |
|  |  |  |  | PLPP1 | 4.52E-18 | -0.332449644 | 9.18E-14 |  |  |  |  |
|  |  |  |  | TRA2B | 4.59E-18 | -0.263418275 | 9.33E-14 |  |  |  |  |
|  |  |  |  | PPP2CA | 5.12E-18 | -0.276566537 | 1.04E-13 |  |  |  |  |
|  |  |  |  | PYHIN1 | 5.69E-18 | 0.340953065 | 1.16E-13 |  |  |  |  |
|  |  |  |  | STAT3 | 5.79E-18 | -0.261282853 | 1.18E-13 |  |  |  |  |
|  |  |  |  | TBX21 | 7.49E-18 | 0.262211754 | 1.52E-13 |  |  |  |  |
|  |  |  |  | SLAMF6 | 7.73E-18 | 0.290956052 | 1.57E-13 |  |  |  |  |
|  |  |  |  | MAPRE2 | 8.15E-18 | -0.264060453 | 1.66E-13 |  |  |  |  |
|  |  |  |  | ZRANB2 | 9.54E-18 | 0.263751239 | 1.94E-13 |  |  |  |  |
|  |  |  |  | CD200R1 | 1.23E-17 | -0.29599485 | 2.51E-13 |  |  |  |  |
|  |  |  |  | GZMM | 1.26E-17 | 0.252362058 | 2.55E-13 |  |  |  |  |
|  |  |  |  | CD55 | 1.79E-17 | -0.552549576 | 3.65E-13 |  |  |  |  |
|  |  |  |  | PTPN6 | 1.81E-17 | 0.265355864 | 3.67E-13 |  |  |  |  |
|  |  |  |  | CENPT | 2.53E-17 | 0.31741123 | 5.15E-13 |  |  |  |  |
|  |  |  |  | STAT6 | 2.62E-17 | 0.276985136 | 5.33E-13 |  |  |  |  |
|  |  |  |  | GK5 | 2.76E-17 | 0.287394729 | 5.61E-13 |  |  |  |  |
|  |  |  |  | HLA-DQA1 | 3.48E-17 | -0.460948431 | 7.07E-13 |  |  |  |  |
|  |  |  |  | FOXO1 | 4.23E-17 | -0.25037259 | 8.60E-13 |  |  |  |  |
|  |  |  |  | SYNJ2BP | 4.70E-17 | 0.301551728 | 9.55E-13 |  |  |  |  |
|  |  |  |  | KCNAB2 | 4.89E-17 | 0.30041661 | 9.94E-13 |  |  |  |  |
|  |  |  |  | ST8SIA4 | 5.16E-17 | -0.264101302 | 1.05E-12 |  |  |  |  |
|  |  |  |  | TRMT13 | 5.90E-17 | 0.255750097 | 1.20E-12 |  |  |  |  |
|  |  |  |  | MED15 | 6.38E-17 | 0.282282155 | 1.30E-12 |  |  |  |  |
|  |  |  |  | IL7R | 6.87E-17 | 0.278646625 | 1.40E-12 |  |  |  |  |
|  |  |  |  | FUT8 | 7.70E-17 | -0.257364612 | 1.56E-12 |  |  |  |  |
|  |  |  |  | PRDM1 | 7.88E-17 | -0.423760508 | 1.60E-12 |  |  |  |  |
|  |  |  |  | SERPINB9 | 8.34E-17 | -0.477897703 | 1.70E-12 |  |  |  |  |
|  |  |  |  | RNF103-CHMP3 | 9.25E-17 | 0.36562845 | 1.88E-12 |  |  |  |  |
|  |  |  |  | ATP5A1 | 9.45E-17 | 0.254173019 | 1.92E-12 |  |  |  |  |
|  |  |  |  | GABPB1 | 1.22E-16 | -0.252565494 | 2.49E-12 |  |  |  |  |
|  |  |  |  | PRF1 | 1.26E-16 | 0.35375482 | 2.56E-12 |  |  |  |  |
|  |  |  |  | EIF3D | 1.40E-16 | 0.337014213 | 2.85E-12 |  |  |  |  |
|  |  |  |  | APMAP | 1.45E-16 | 0.404307719 | 2.95E-12 |  |  |  |  |
|  |  |  |  | TRAF1 | 1.48E-16 | -0.323649944 | 3.00E-12 |  |  |  |  |
|  |  |  |  | ALG13 | 1.73E-16 | -0.368338168 | 3.52E-12 |  |  |  |  |
|  |  |  |  | FOXP1 | 1.81E-16 | 0.312166379 | 3.69E-12 |  |  |  |  |
|  |  |  |  | NCR3 | 1.86E-16 | 0.256481248 | 3.78E-12 |  |  |  |  |
|  |  |  |  | STAM | 2.13E-16 | -0.258292771 | 4.34E-12 |  |  |  |  |
|  |  |  |  | ARHGAP45 | 2.25E-16 | 0.355583941 | 4.58E-12 |  |  |  |  |
|  |  |  |  | FBXO32 | 2.33E-16 | 0.260318389 | 4.74E-12 |  |  |  |  |
|  |  |  |  | XBP1 | 2.38E-16 | 0.338979026 | 4.83E-12 |  |  |  |  |
|  |  |  |  | TRAPPC2 | 2.90E-16 | 0.328680978 | 5.89E-12 |  |  |  |  |
|  |  |  |  | MAT2A | 3.12E-16 | -0.318189555 | 6.35E-12 |  |  |  |  |
|  |  |  |  | FBXW5 | 3.29E-16 | 0.33275892 | 6.70E-12 |  |  |  |  |
|  |  |  |  | TPM4 | 4.58E-16 | -0.252406642 | 9.32E-12 |  |  |  |  |
|  |  |  |  | SC5D | 5.00E-16 | -0.427154907 | 1.02E-11 |  |  |  |  |
|  |  |  |  | NAAA | 6.10E-16 | 0.293260077 | 1.24E-11 |  |  |  |  |
|  |  |  |  | CASP1 | 6.28E-16 | 0.330084709 | 1.28E-11 |  |  |  |  |
|  |  |  |  | FOXK1 | 6.70E-16 | 0.252885976 | 1.36E-11 |  |  |  |  |
|  |  |  |  | ARHGAP30 | 7.44E-16 | 0.320835726 | 1.51E-11 |  |  |  |  |
|  |  |  |  | ARAP2 | 7.83E-16 | -0.264264174 | 1.59E-11 |  |  |  |  |
|  |  |  |  | CD37 | 7.83E-16 | 0.25535017 | 1.59E-11 |  |  |  |  |
|  |  |  |  | CD5 | 7.92E-16 | 0.277104691 | 1.61E-11 |  |  |  |  |
|  |  |  |  | DPP4 | 9.42E-16 | 0.288475647 | 1.92E-11 |  |  |  |  |
|  |  |  |  | FGD3 | 9.61E-16 | 0.291774732 | 1.95E-11 |  |  |  |  |
|  |  |  |  | CBR4 | 1.21E-15 | 0.258513357 | 2.46E-11 |  |  |  |  |
|  |  |  |  | APEX1 | 1.53E-15 | 0.343818782 | 3.11E-11 |  |  |  |  |
|  |  |  |  | ADHFE1 | 1.71E-15 | 0.267388324 | 3.47E-11 |  |  |  |  |
|  |  |  |  | KRI1 | 2.32E-15 | 0.294804256 | 4.71E-11 |  |  |  |  |
|  |  |  |  | MRPS18B | 2.94E-15 | 0.335512534 | 5.98E-11 |  |  |  |  |
|  |  |  |  | GVINP1 | 3.04E-15 | 0.289759719 | 6.17E-11 |  |  |  |  |
|  |  |  |  | SPSB3 | 3.98E-15 | 0.280885919 | 8.08E-11 |  |  |  |  |
|  |  |  |  | SLC25A20 | 4.24E-15 | 0.301138812 | 8.61E-11 |  |  |  |  |
|  |  |  |  | TUBA1B | 4.80E-15 | -0.388342485 | 9.76E-11 |  |  |  |  |
|  |  |  |  | MAD1L1 | 4.85E-15 | 0.252180269 | 9.86E-11 |  |  |  |  |
|  |  |  |  | OAS3 | 5.48E-15 | -0.251183174 | 1.11E-10 |  |  |  |  |
|  |  |  |  | NOL4L | 5.98E-15 | 0.291502325 | 1.22E-10 |  |  |  |  |
|  |  |  |  | NFKBID | 6.39E-15 | -0.295855001 | 1.30E-10 |  |  |  |  |
|  |  |  |  | CTNNA1 | 7.64E-15 | -0.250158012 | 1.55E-10 |  |  |  |  |
|  |  |  |  | DOCK10 | 8.63E-15 | -0.301150999 | 1.75E-10 |  |  |  |  |
|  |  |  |  | HPS1 | 9.14E-15 | 0.260355067 | 1.86E-10 |  |  |  |  |
|  |  |  |  | TTN | 1.14E-14 | -0.383303317 | 2.32E-10 |  |  |  |  |
|  |  |  |  | ARID5B | 1.22E-14 | -0.312738271 | 2.48E-10 |  |  |  |  |
|  |  |  |  | ZNF91 | 1.28E-14 | 0.389147007 | 2.61E-10 |  |  |  |  |
|  |  |  |  | TRAF5 | 1.29E-14 | -0.27188465 | 2.62E-10 |  |  |  |  |
|  |  |  |  | GZMH | 1.54E-14 | 0.489760913 | 3.13E-10 |  |  |  |  |
|  |  |  |  | RELL1 | 1.94E-14 | -0.270866682 | 3.95E-10 |  |  |  |  |
|  |  |  |  | SNHG15 | 2.03E-14 | -0.30097308 | 4.12E-10 |  |  |  |  |
|  |  |  |  | BCLAF1 | 2.29E-14 | -0.256194243 | 4.65E-10 |  |  |  |  |
|  |  |  |  | TNFRSF1A | 2.41E-14 | 0.309811625 | 4.89E-10 |  |  |  |  |
|  |  |  |  | SRRT | 2.42E-14 | -0.290979762 | 4.93E-10 |  |  |  |  |
|  |  |  |  | CA5B | 2.44E-14 | 0.263300221 | 4.96E-10 |  |  |  |  |
|  |  |  |  | SGSM3 | 2.61E-14 | 0.332271456 | 5.30E-10 |  |  |  |  |
|  |  |  |  | FLI1 | 3.02E-14 | 0.272877108 | 6.13E-10 |  |  |  |  |
|  |  |  |  | TESPA1 | 3.47E-14 | 0.258350134 | 7.06E-10 |  |  |  |  |
|  |  |  |  | ZNF37BP | 4.13E-14 | 0.314086488 | 8.39E-10 |  |  |  |  |
|  |  |  |  | ERP29 | 4.76E-14 | 0.297146197 | 9.67E-10 |  |  |  |  |
|  |  |  |  | AP1G2 | 7.26E-14 | 0.27099819 | 1.48E-09 |  |  |  |  |
|  |  |  |  | TNFRSF14 | 8.76E-14 | 0.297976345 | 1.78E-09 |  |  |  |  |
|  |  |  |  | CDC42EP3 | 1.00E-13 | 0.265256742 | 2.03E-09 |  |  |  |  |
|  |  |  |  | PDIA6 | 1.01E-13 | -0.270770702 | 2.04E-09 |  |  |  |  |
|  |  |  |  | C2orf68 | 1.38E-13 | 0.277840515 | 2.81E-09 |  |  |  |  |
|  |  |  |  | AP1M1 | 1.70E-13 | 0.284036814 | 3.45E-09 |  |  |  |  |
|  |  |  |  | C12orf75 | 1.85E-13 | 0.317929666 | 3.76E-09 |  |  |  |  |
|  |  |  |  | CD74 | 1.85E-13 | -0.341649739 | 3.76E-09 |  |  |  |  |
|  |  |  |  | IMP3 | 1.86E-13 | 0.257550864 | 3.79E-09 |  |  |  |  |
|  |  |  |  | CRYBG1 | 2.19E-13 | -0.61339075 | 4.45E-09 |  |  |  |  |
|  |  |  |  | SLC43A3 | 2.26E-13 | -0.261895424 | 4.59E-09 |  |  |  |  |
|  |  |  |  | KCNQ1OT1 | 2.39E-13 | -0.279961497 | 4.86E-09 |  |  |  |  |
|  |  |  |  | ARHGAP15 | 2.73E-13 | 0.289960142 | 5.54E-09 |  |  |  |  |
|  |  |  |  | DGKA | 3.17E-13 | 0.370307847 | 6.45E-09 |  |  |  |  |
|  |  |  |  | CALHM2 | 3.78E-13 | 0.317455431 | 7.67E-09 |  |  |  |  |
|  |  |  |  | C4orf3 | 3.85E-13 | 0.317148849 | 7.82E-09 |  |  |  |  |
|  |  |  |  | PTP4A2 | 4.50E-13 | 0.31559897 | 9.15E-09 |  |  |  |  |
|  |  |  |  | ANXA6 | 4.57E-13 | 0.271589694 | 9.30E-09 |  |  |  |  |
|  |  |  |  | AKAP13 | 4.67E-13 | -0.257633202 | 9.48E-09 |  |  |  |  |
|  |  |  |  | RHOH | 8.59E-13 | -0.260179111 | 1.75E-08 |  |  |  |  |
|  |  |  |  | IL6ST | 1.08E-12 | -0.30755784 | 2.20E-08 |  |  |  |  |
|  |  |  |  | EOMES | 1.30E-12 | 0.309048135 | 2.64E-08 |  |  |  |  |
|  |  |  |  | ACAA2 | 1.36E-12 | 0.329506928 | 2.77E-08 |  |  |  |  |
|  |  |  |  | SRPK2 | 1.48E-12 | 0.254787262 | 3.00E-08 |  |  |  |  |
|  |  |  |  | TXK | 1.74E-12 | 0.263876788 | 3.53E-08 |  |  |  |  |
|  |  |  |  | PCSK7 | 1.93E-12 | 0.283210714 | 3.92E-08 |  |  |  |  |
|  |  |  |  | CCDC18-AS1 | 2.14E-12 | 0.302885081 | 4.36E-08 |  |  |  |  |
|  |  |  |  | IER2 | 2.44E-12 | -0.410377273 | 4.97E-08 |  |  |  |  |
|  |  |  |  | EGLN2 | 3.16E-12 | 0.269885613 | 6.42E-08 |  |  |  |  |
|  |  |  |  | LAIR1 | 3.25E-12 | 0.252463318 | 6.60E-08 |  |  |  |  |
|  |  |  |  | SRSF1 | 3.60E-12 | 0.261529851 | 7.32E-08 |  |  |  |  |
|  |  |  |  | DENND4B | 5.33E-12 | 0.274530531 | 1.08E-07 |  |  |  |  |
|  |  |  |  | UBQLN2 | 5.55E-12 | 0.347407916 | 1.13E-07 |  |  |  |  |
|  |  |  |  | CFAP20 | 6.18E-12 | -0.306430817 | 1.26E-07 |  |  |  |  |
|  |  |  |  | SPN | 6.38E-12 | 0.275246428 | 1.30E-07 |  |  |  |  |
|  |  |  |  | GAB3 | 7.28E-12 | 0.250079218 | 1.48E-07 |  |  |  |  |
|  |  |  |  | HAUS3 | 7.72E-12 | -0.253756021 | 1.57E-07 |  |  |  |  |
|  |  |  |  | TM2D3 | 8.82E-12 | -0.257447678 | 1.79E-07 |  |  |  |  |
|  |  |  |  | DIS3L | 8.83E-12 | 0.254607022 | 1.79E-07 |  |  |  |  |
|  |  |  |  | PKM | 9.30E-12 | -0.324476756 | 1.89E-07 |  |  |  |  |
|  |  |  |  | STIP1 | 1.16E-11 | -0.271521243 | 2.36E-07 |  |  |  |  |
|  |  |  |  | XCL1 | 1.24E-11 | -0.293403182 | 2.52E-07 |  |  |  |  |
|  |  |  |  | IFI6 | 1.37E-11 | -0.256221327 | 2.79E-07 |  |  |  |  |
|  |  |  |  | CNPY2 | 1.49E-11 | 0.25569962 | 3.03E-07 |  |  |  |  |
|  |  |  |  | SCOC | 1.88E-11 | 0.278802156 | 3.81E-07 |  |  |  |  |
|  |  |  |  | NDE1 | 1.93E-11 | 0.265800744 | 3.93E-07 |  |  |  |  |
|  |  |  |  | RNFT1 | 2.00E-11 | 0.274158247 | 4.06E-07 |  |  |  |  |
|  |  |  |  | PGS1 | 2.33E-11 | 0.264958002 | 4.73E-07 |  |  |  |  |
|  |  |  |  | GZMK | 2.67E-11 | -0.283995445 | 5.42E-07 |  |  |  |  |
|  |  |  |  | ANAPC5 | 3.15E-11 | 0.271159344 | 6.39E-07 |  |  |  |  |
|  |  |  |  | ANXA4 | 4.92E-11 | 0.253683117 | 1.00E-06 |  |  |  |  |
|  |  |  |  | CHURC1 | 6.27E-11 | 0.291970672 | 1.27E-06 |  |  |  |  |
|  |  |  |  | SYNGR2 | 8.62E-11 | -0.256289343 | 1.75E-06 |  |  |  |  |
|  |  |  |  | ESYT1 | 9.47E-11 | 0.265350539 | 1.93E-06 |  |  |  |  |
|  |  |  |  | PTEN | 1.03E-10 | 0.269147581 | 2.09E-06 |  |  |  |  |
|  |  |  |  | RNF166 | 1.08E-10 | 0.252498045 | 2.19E-06 |  |  |  |  |
|  |  |  |  | GAS5 | 1.59E-10 | -0.318828698 | 3.23E-06 |  |  |  |  |
|  |  |  |  | TAOK3 | 1.90E-10 | 0.276721745 | 3.85E-06 |  |  |  |  |
|  |  |  |  | CHMP1B | 2.22E-10 | -0.292930901 | 4.51E-06 |  |  |  |  |
|  |  |  |  | GLOD4 | 2.29E-10 | 0.266410242 | 4.66E-06 |  |  |  |  |
|  |  |  |  | TRADD | 2.68E-10 | 0.250646934 | 5.44E-06 |  |  |  |  |
|  |  |  |  | UBE2G2 | 2.79E-10 | 0.286894612 | 5.66E-06 |  |  |  |  |
|  |  |  |  | MX1 | 2.94E-10 | -0.347776651 | 5.98E-06 |  |  |  |  |
|  |  |  |  | APOBEC3G | 3.20E-10 | -0.367498143 | 6.51E-06 |  |  |  |  |
|  |  |  |  | TRG-AS1 | 3.41E-10 | 0.266590807 | 6.93E-06 |  |  |  |  |
|  |  |  |  | ZBED5 | 3.45E-10 | 0.276500678 | 7.01E-06 |  |  |  |  |
|  |  |  |  | CACYBP | 3.73E-10 | -0.27670596 | 7.57E-06 |  |  |  |  |
|  |  |  |  | PEPD | 4.87E-10 | 0.257557004 | 9.89E-06 |  |  |  |  |
|  |  |  |  | VPS35 | 7.66E-10 | 0.276720796 | 1.56E-05 |  |  |  |  |
|  |  |  |  | RRP15 | 1.35E-09 | 0.271146743 | 2.75E-05 |  |  |  |  |
|  |  |  |  | TRIP11 | 1.48E-09 | 0.250039802 | 3.00E-05 |  |  |  |  |
|  |  |  |  | HDDC2 | 1.99E-09 | 0.255652955 | 4.05E-05 |  |  |  |  |
|  |  |  |  | TSN | 2.17E-09 | 0.286139264 | 4.41E-05 |  |  |  |  |
|  |  |  |  | ANKZF1 | 2.55E-09 | 0.288190193 | 5.19E-05 |  |  |  |  |
|  |  |  |  | SQSTM1 | 4.10E-09 | -0.25732402 | 8.32E-05 |  |  |  |  |
|  |  |  |  | TCF25 | 4.84E-09 | 0.276623476 | 9.83E-05 |  |  |  |  |
|  |  |  |  | ACP1 | 6.14E-09 | 0.290364694 | 0.000124711 |  |  |  |  |
|  |  |  |  | 6-Mar | 6.64E-09 | 0.294582871 | 0.000134967 |  |  |  |  |
|  |  |  |  | ZNF766 | 7.07E-09 | 0.250624754 | 0.000143636 |  |  |  |  |
|  |  |  |  | ZNF33B | 9.44E-09 | 0.262976955 | 0.00019192 |  |  |  |  |
|  |  |  |  | PILRB | 1.13E-08 | 0.264261746 | 0.000229331 |  |  |  |  |
|  |  |  |  | KLHDC3 | 1.21E-08 | 0.283099433 | 0.000244991 |  |  |  |  |
|  |  |  |  | RSU1 | 1.30E-08 | 0.252003096 | 0.000264205 |  |  |  |  |
|  |  |  |  | C6orf62 | 1.69E-08 | 0.272379314 | 0.000343406 |  |  |  |  |
|  |  |  |  | MFSD8 | 1.73E-08 | 0.253152766 | 0.000352284 |  |  |  |  |
|  |  |  |  | BRD3OS | 2.39E-08 | 0.2686878 | 0.000485158 |  |  |  |  |
|  |  |  |  | SEC24C | 2.81E-08 | 0.2817622 | 0.000571424 |  |  |  |  |
|  |  |  |  | SF3A3 | 3.18E-08 | 0.263543333 | 0.000647223 |  |  |  |  |
|  |  |  |  | GOLGA4 | 4.50E-08 | 0.256114008 | 0.000914965 |  |  |  |  |
|  |  |  |  | NDUFS2 | 6.80E-08 | 0.254902679 | 0.001382514 |  |  |  |  |
|  |  |  |  | ZNF559 | 7.94E-08 | 0.286796173 | 0.001612714 |  |  |  |  |
|  |  |  |  | NEAT1 | 1.75E-07 | -0.258200647 | 0.003552132 |  |  |  |  |
|  |  |  |  | MYD88 | 2.26E-07 | 0.277938949 | 0.004584612 |  |  |  |  |
|  |  |  |  | BIN1 | 6.41E-07 | 0.253396658 | 0.013022395 |  |  |  |  |
|  |  |  |  | HOPX | 7.01E-07 | -0.255301152 | 0.014241075 |  |  |  |  |
|  |  |  |  | EIF3E | 1.31E-06 | 0.262086155 | 0.026631211 |  |  |  |  |
|  |  |  |  | CCR6 | 1.84E-06 | -0.326615452 | 0.037473115 |  |  |  |  |
|  |  |  |  | TTC14 | 2.41E-06 | 0.266630109 | 0.049066034 |  |  |  |  |
|  |  |  |  | PLIN2 | 2.61E-06 | -0.278729609 | 0.0530244 |  |  |  |  |
|  |  |  |  | SLC35A4 | 2.88E-06 | 0.258132985 | 0.058464638 |  |  |  |  |
|  |  |  |  | HSPB1 | 8.06E-06 | -0.355345298 | 0.163751818 |  |  |  |  |
|  |  |  |  | GNLY | 2.37E-05 | 0.380618355 | 0.481987415 |  |  |  |  |
|  |  |  |  | CD160 | 2.94E-05 | -0.277253717 | 0.597204864 |  |  |  |  |
|  |  |  |  | SERINC5 | 4.24E-05 | 0.356218252 | 0.860897758 |  |  |  |  |
|  |  |  |  | WDR6 | 4.61E-05 | 0.254458523 | 0.937151133 |  |  |  |  |
|  |  |  |  | ZNF600 | 5.36E-05 | 0.268115003 | 1 |  |  |  |  |
|  |  |  |  | KLRD1 | 0.000252112 | 0.289153356 | 1 |  |  |  |  |
|  |  |  |  | ZNF83 | 0.000252199 | 0.282093449 | 1 |  |  |  |  |
|  |  |  |  | STMN1 | 0.00050235 | -0.362089789 | 1 |  |  |  |  |
|  |  |  |  | GPR183 | 0.00084845 | -0.428008488 | 1 |  |  |  |  |
|  |  |  |  | HSPB11 | 0.001030059 | 0.256136956 | 1 |  |  |  |  |
|  |  |  |  | NELL2 | 0.031009346 | 0.358507189 | 1 |  |  |  |  |
|  |  |  |  | ANXA1 | 0.10619474 | -0.385128025 | 1 |  |  |  |  |
