# Supplementary material for: Dynamic Network Biomarker of Pre-Exhausted CD8+ T Cells Contributed to T Cell Exhaustion in Colorectal Cancer
Source: Front Immunol. 2021 Aug 9;12:691142. doi: 10.3389/fimmu.2021.691142 (PMC8381053; doi:10.3389/fimmu.2021.691142)
Supplement: Supplementary file 5 [file Table_2.docx]

| **Cluster1** | | | | | |
| --- | --- | --- | --- | --- | --- |
| GeneSymbol | p_val | logFC | pct.1 | pct.2 | p_val_adj |
| ZFP36 | 2.34E-70 | 1.134233 | 0.983 | 0.97 | 4.23E-66 |
| EIF1 | 4.77E-60 | 0.695755 | 0.997 | 0.999 | 8.60E-56 |
| APOBEC3C | 3.07E-56 | -0.80817 | 0.796 | 0.974 | 5.53E-52 |
| DNAJB1 | 6.52E-56 | 1.095966 | 0.997 | 0.905 | 1.18E-51 |
| CXCR4 | 5.81E-52 | 0.850094 | 0.994 | 0.991 | 1.05E-47 |
| ACTB | 1.91E-51 | -0.53588 | 1 | 1 | 3.43E-47 |
| LMNA | 1.00E-49 | 1.314732 | 0.735 | 0.372 | 1.81E-45 |
| TNFAIP3 | 7.65E-45 | 0.806687 | 0.992 | 0.974 | 1.38E-40 |
| HAVCR2 | 1.11E-44 | -0.92014 | 0.461 | 0.801 | 2.00E-40 |
| APOBEC3G | 6.26E-42 | -0.69175 | 0.849 | 0.976 | 1.13E-37 |
| PSMB9 | 7.50E-42 | -0.58604 | 0.832 | 0.979 | 1.35E-37 |
| NR4A2 | 5.22E-41 | 0.792473 | 0.969 | 0.748 | 9.41E-37 |
| APOBEC3F | 9.20E-41 | -0.31052 | 0.469 | 0.83 | 1.66E-36 |
| PFN1 | 6.59E-40 | -0.44796 | 1 | 1 | 1.19E-35 |
| BTG2 | 6.69E-40 | 0.995762 | 0.93 | 0.745 | 1.21E-35 |
| ITGAE | 1.21E-39 | -0.70483 | 0.754 | 0.947 | 2.18E-35 |
| CORO1A | 4.45E-39 | -0.55342 | 0.953 | 0.998 | 8.02E-35 |
| CXCR6 | 2.36E-38 | -1.03132 | 0.682 | 0.9 | 4.25E-34 |
| CREM | 3.02E-38 | 0.998091 | 0.944 | 0.813 | 5.45E-34 |
| SIT1 | 6.19E-38 | -0.52149 | 0.436 | 0.796 | 1.12E-33 |
| SARAF | 8.41E-38 | 0.624415 | 0.992 | 0.999 | 1.52E-33 |
| SH3BGRL3 | 1.39E-37 | -0.50301 | 0.992 | 1 | 2.50E-33 |
| SAMD9L | 1.87E-37 | -0.58921 | 0.358 | 0.752 | 3.38E-33 |
| CD27 | 2.65E-37 | -0.70815 | 0.598 | 0.873 | 4.78E-33 |
| FKBP1A | 6.90E-37 | -0.6369 | 0.673 | 0.914 | 1.24E-32 |
| YPEL5 | 2.95E-36 | 0.842293 | 0.941 | 0.905 | 5.31E-32 |
| DUSP1 | 4.50E-36 | 0.854897 | 0.966 | 0.943 | 8.11E-32 |
| CD27-AS1 | 1.22E-35 | -0.59955 | 0.52 | 0.83 | 2.21E-31 |
| LCP2 | 3.35E-35 | -0.42934 | 0.74 | 0.958 | 6.03E-31 |
| PKM | 8.18E-35 | -0.63061 | 0.76 | 0.952 | 1.48E-30 |
| SRSF7 | 1.37E-34 | 0.603906 | 0.992 | 0.995 | 2.47E-30 |
| SASH3 | 2.04E-34 | -0.52601 | 0.587 | 0.879 | 3.67E-30 |
| ARPC5 | 2.17E-34 | -0.5227 | 0.712 | 0.939 | 3.90E-30 |
| COPZ1 | 3.11E-34 | -0.43163 | 0.383 | 0.748 | 5.60E-30 |
| CALM3 | 6.54E-34 | -0.3418 | 0.525 | 0.852 | 1.18E-29 |
| SIRPG | 1.12E-33 | -0.54175 | 0.503 | 0.807 | 2.02E-29 |
| TNFSF10 | 2.03E-33 | -0.39322 | 0.237 | 0.623 | 3.66E-29 |
| CD82 | 2.43E-33 | -0.62466 | 0.676 | 0.894 | 4.38E-29 |
| CSRNP1 | 5.99E-33 | 0.802541 | 0.732 | 0.451 | 1.08E-28 |
| APOBEC3D | 7.48E-33 | -0.27787 | 0.291 | 0.673 | 1.35E-28 |
| TUBB4B | 9.29E-33 | 0.878679 | 0.855 | 0.634 | 1.67E-28 |
| TRAF3IP3 | 1.10E-32 | -0.50554 | 0.612 | 0.879 | 1.98E-28 |
| CD52 | 1.29E-32 | -0.47595 | 0.947 | 0.993 | 2.32E-28 |
| SYNC | 1.34E-32 | -0.29297 | 0.299 | 0.653 | 2.41E-28 |
| LOC606724 | 2.38E-32 | -0.35135 | 0.86 | 0.966 | 4.28E-28 |
| SRGN | 3.14E-32 | 0.432921 | 0.997 | 0.998 | 5.66E-28 |
| DNAJA1 | 3.30E-32 | 0.817697 | 0.975 | 0.9 | 5.95E-28 |
| SERTAD1 | 3.46E-32 | 0.820021 | 0.723 | 0.465 | 6.24E-28 |
| FASLG | 5.13E-32 | -0.84505 | 0.411 | 0.718 | 9.25E-28 |
| HSPA8 | 6.50E-32 | 0.764164 | 1 | 0.998 | 1.17E-27 |
| GBP1 | 6.96E-32 | -0.4803 | 0.411 | 0.773 | 1.25E-27 |
| PPP1R15A | 8.49E-32 | 0.805382 | 0.944 | 0.825 | 1.53E-27 |
| CCL3 | 2.15E-31 | -0.80616 | 0.439 | 0.753 | 3.87E-27 |
| RBBP4 | 2.70E-31 | -0.50388 | 0.503 | 0.793 | 4.87E-27 |
| FOS | 3.79E-31 | 0.970187 | 0.961 | 0.811 | 6.83E-27 |
| COTL1 | 3.89E-31 | -0.61541 | 0.883 | 0.975 | 7.01E-27 |
| CXCL13 | 4.79E-31 | -1.29754 | 0.483 | 0.722 | 8.64E-27 |
| SNAP47 | 9.87E-31 | -0.39294 | 0.176 | 0.524 | 1.78E-26 |
| FTH1 | 2.24E-30 | 0.759201 | 1 | 0.999 | 4.04E-26 |
| OAS2 | 2.71E-30 | -0.57342 | 0.265 | 0.605 | 4.88E-26 |
| ANXA6 | 2.96E-30 | -0.51582 | 0.64 | 0.891 | 5.33E-26 |
| CAP1 | 4.33E-30 | -0.46229 | 0.863 | 0.99 | 7.80E-26 |
| ACP5 | 7.22E-30 | -0.60614 | 0.363 | 0.677 | 1.30E-25 |
| ARPC2 | 7.38E-30 | -0.38955 | 0.989 | 0.999 | 1.33E-25 |
| GPR183 | 1.46E-29 | 0.995222 | 0.855 | 0.731 | 2.63E-25 |
| TSC22D3 | 1.51E-29 | 0.807343 | 0.944 | 0.982 | 2.72E-25 |
| SRSF2 | 1.57E-29 | 0.579602 | 0.966 | 0.976 | 2.82E-25 |
| PARP9 | 1.63E-29 | -0.46552 | 0.439 | 0.773 | 2.94E-25 |
| PTPN6 | 2.99E-29 | -0.46972 | 0.427 | 0.776 | 5.40E-25 |
| RPL3 | 6.74E-29 | 0.392257 | 1 | 0.998 | 1.21E-24 |
| PSMA2 | 7.47E-29 | -0.44237 | 0.587 | 0.877 | 1.35E-24 |
| CD38 | 1.08E-28 | -0.47289 | 0.237 | 0.569 | 1.94E-24 |
| UBC | 1.26E-28 | 0.422171 | 1 | 1 | 2.28E-24 |
| ARPC5L | 1.42E-28 | -0.32875 | 0.31 | 0.668 | 2.56E-24 |
| XAF1 | 1.48E-28 | -0.41198 | 0.38 | 0.705 | 2.67E-24 |
| C7orf25 | 1.51E-28 | -0.42979 | 0.556 | 0.863 | 2.72E-24 |
| OAS1 | 1.76E-28 | -0.59823 | 0.128 | 0.45 | 3.17E-24 |
| JUNB | 3.02E-28 | 0.843331 | 0.964 | 0.939 | 5.44E-24 |
| DDX60 | 3.18E-28 | -0.45425 | 0.254 | 0.595 | 5.74E-24 |
| MYADM | 3.19E-28 | 0.828367 | 0.841 | 0.644 | 5.76E-24 |
| PSME2 | 3.89E-28 | -0.42706 | 0.754 | 0.946 | 7.01E-24 |
| ANXA5 | 6.08E-28 | -0.52444 | 0.698 | 0.909 | 1.10E-23 |
| STAT1 | 1.03E-27 | -0.58113 | 0.656 | 0.877 | 1.85E-23 |
| CCR1 | 1.28E-27 | -0.59356 | 0.101 | 0.411 | 2.30E-23 |
| NKG7 | 1.65E-27 | -0.448 | 0.966 | 0.997 | 2.97E-23 |
| ATP5F1 | 1.71E-27 | -0.37698 | 0.525 | 0.832 | 3.07E-23 |
| FAM166A | 2.15E-27 | 0.390049 | 0.698 | 0.467 | 3.88E-23 |
| PTPN7 | 2.73E-27 | -0.4632 | 0.707 | 0.922 | 4.93E-23 |
| LASP1 | 2.95E-27 | -0.45069 | 0.453 | 0.765 | 5.32E-23 |
| H3F3B | 4.11E-27 | 0.373187 | 0.997 | 1 | 7.41E-23 |
| CD2BP2 | 5.95E-27 | -0.4363 | 0.285 | 0.62 | 1.07E-22 |
| RBPJ | 6.72E-27 | -0.57738 | 0.709 | 0.901 | 1.21E-22 |
| TNFSF4 | 9.46E-27 | -0.81929 | 0.17 | 0.478 | 1.71E-22 |
| CHST12 | 1.17E-26 | -0.38854 | 0.419 | 0.734 | 2.11E-22 |
| HSPH1 | 1.17E-26 | 1.074303 | 0.93 | 0.75 | 2.12E-22 |
| UCP2 | 1.27E-26 | -0.473 | 0.76 | 0.94 | 2.29E-22 |
| ADAM19 | 1.66E-26 | -0.25118 | 0.472 | 0.79 | 3.00E-22 |
| S100PBP | 2.35E-26 | -0.36102 | 0.162 | 0.478 | 4.23E-22 |
| GZMA | 2.37E-26 | -0.52579 | 0.947 | 0.991 | 4.27E-22 |
| ATP5G3 | 2.52E-26 | -0.36693 | 0.559 | 0.849 | 4.53E-22 |
| PRELID1 | 3.05E-26 | -0.354 | 0.425 | 0.741 | 5.49E-22 |
| GZMB | 3.24E-26 | -0.51051 | 0.86 | 0.983 | 5.84E-22 |
| FERMT3 | 3.40E-26 | -0.3952 | 0.564 | 0.841 | 6.13E-22 |
| ELL2 | 3.64E-26 | 0.477073 | 0.5 | 0.229 | 6.56E-22 |
| RAB27A | 6.59E-26 | -0.44422 | 0.497 | 0.8 | 1.19E-21 |
| CD63 | 6.64E-26 | -0.4531 | 0.679 | 0.904 | 1.20E-21 |
| IKZF3 | 6.75E-26 | -0.52268 | 0.785 | 0.937 | 1.22E-21 |
| SEC11A | 7.42E-26 | -0.36691 | 0.374 | 0.691 | 1.34E-21 |
| UBE2L6 | 7.65E-26 | -0.42685 | 0.628 | 0.901 | 1.38E-21 |
| GIMAP6 | 8.16E-26 | -0.51167 | 0.246 | 0.582 | 1.47E-21 |
| PARK7 | 8.25E-26 | -0.36156 | 0.723 | 0.929 | 1.49E-21 |
| HLA-DQA1 | 8.54E-26 | -0.44024 | 0.439 | 0.732 | 1.54E-21 |
| PAG1 | 1.06E-25 | -0.39928 | 0.645 | 0.869 | 1.91E-21 |
| MXD3 | 1.17E-25 | -0.28758 | 0.385 | 0.711 | 2.10E-21 |
| PSMB8 | 1.24E-25 | -0.44655 | 0.763 | 0.952 | 2.23E-21 |
| TMEM165 | 1.27E-25 | -0.32108 | 0.299 | 0.64 | 2.29E-21 |
| CD2 | 1.28E-25 | -0.44513 | 0.964 | 0.996 | 2.31E-21 |
| FIBP | 1.48E-25 | -0.26272 | 0.388 | 0.698 | 2.66E-21 |
| RPS6KA1 | 1.51E-25 | -0.51411 | 0.221 | 0.523 | 2.72E-21 |
| FUT8 | 1.68E-25 | -0.3311 | 0.268 | 0.588 | 3.03E-21 |
| IL2RG | 1.85E-25 | -0.39114 | 0.972 | 0.998 | 3.34E-21 |
| CSK | 2.50E-25 | -0.28249 | 0.321 | 0.66 | 4.50E-21 |
| ARPC3 | 2.52E-25 | -0.379 | 0.838 | 0.981 | 4.55E-21 |
| TRG-AS1 | 2.66E-25 | -0.47277 | 0.277 | 0.588 | 4.79E-21 |
| RAC2 | 3.00E-25 | -0.40297 | 0.947 | 0.997 | 5.41E-21 |
| PPP1R18 | 3.41E-25 | -0.26634 | 0.469 | 0.76 | 6.14E-21 |
| ESYT1 | 3.41E-25 | -0.4198 | 0.469 | 0.786 | 6.14E-21 |
| ARHGAP30 | 4.98E-25 | -0.40732 | 0.55 | 0.797 | 8.97E-21 |
| DAD1 | 5.59E-25 | -0.30204 | 0.531 | 0.815 | 1.01E-20 |
| COMMD3 | 6.88E-25 | -0.39685 | 0.218 | 0.54 | 1.24E-20 |
| HSP90AB1 | 7.89E-25 | 0.766889 | 0.992 | 0.993 | 1.42E-20 |
| ARPC4 | 8.60E-25 | -0.32125 | 0.723 | 0.928 | 1.55E-20 |
| LY6E | 1.04E-24 | -0.47889 | 0.718 | 0.943 | 1.87E-20 |
| BRK1 | 1.11E-24 | -0.39105 | 0.497 | 0.79 | 2.00E-20 |
| MIR155HG | 1.34E-24 | -0.49569 | 0.271 | 0.575 | 2.41E-20 |
| TBC1D4 | 1.86E-24 | -0.43349 | 0.24 | 0.559 | 3.36E-20 |
| ADGRG5 | 2.47E-24 | -0.38743 | 0.179 | 0.476 | 4.45E-20 |
| IDH2 | 2.72E-24 | -0.4491 | 0.768 | 0.94 | 4.90E-20 |
| UBXN11 | 2.80E-24 | -0.29575 | 0.936 | 0.992 | 5.04E-20 |
| ARF6 | 3.04E-24 | -0.33663 | 0.743 | 0.915 | 5.49E-20 |
| HSP90AA1 | 3.35E-24 | 0.947657 | 0.994 | 0.997 | 6.04E-20 |
| CLIC1 | 3.71E-24 | -0.3548 | 0.902 | 0.995 | 6.68E-20 |
| STMP1 | 3.86E-24 | -0.34799 | 0.263 | 0.554 | 6.96E-20 |
| NDFIP2 | 4.07E-24 | -0.7369 | 0.413 | 0.668 | 7.34E-20 |
| DTX3L | 4.52E-24 | -0.41952 | 0.243 | 0.547 | 8.16E-20 |
| BATF | 4.85E-24 | -0.29378 | 0.372 | 0.717 | 8.75E-20 |
| ZNF331 | 5.13E-24 | 0.773382 | 0.869 | 0.67 | 9.24E-20 |
| CXCR3 | 5.65E-24 | -0.33493 | 0.55 | 0.836 | 1.02E-19 |
| PCED1B | 6.01E-24 | -0.32395 | 0.732 | 0.943 | 1.08E-19 |
| NME1-NME2 | 6.44E-24 | -0.29102 | 0.341 | 0.655 | 1.16E-19 |
| PTPRCAP | 6.57E-24 | -0.38939 | 0.95 | 0.992 | 1.18E-19 |
| BST2 | 6.88E-24 | -0.33782 | 0.447 | 0.757 | 1.24E-19 |
| PYM1 | 7.93E-24 | -0.25212 | 0.073 | 0.352 | 1.43E-19 |
| GAPDH | 8.01E-24 | -0.47131 | 0.997 | 1 | 1.44E-19 |
| CD83 | 8.11E-24 | 0.642364 | 0.581 | 0.313 | 1.46E-19 |
| PPM1M | 1.18E-23 | -0.34 | 0.215 | 0.524 | 2.12E-19 |
| PTPA | 1.32E-23 | -0.25587 | 0.154 | 0.452 | 2.38E-19 |
| IL16 | 1.34E-23 | -0.37664 | 0.547 | 0.821 | 2.42E-19 |
| FBXO6 | 1.36E-23 | -0.36602 | 0.109 | 0.387 | 2.45E-19 |
| CDK6 | 1.41E-23 | -0.41117 | 0.355 | 0.634 | 2.55E-19 |
| PPP1CA | 1.92E-23 | -0.36925 | 0.763 | 0.941 | 3.47E-19 |
| GSTO1 | 2.05E-23 | -0.32581 | 0.324 | 0.628 | 3.69E-19 |
| SRI | 2.32E-23 | -0.30496 | 0.466 | 0.782 | 4.19E-19 |
| INPP4B | 2.37E-23 | -0.33308 | 0.553 | 0.822 | 4.27E-19 |
| CTSD | 2.75E-23 | -0.48099 | 0.757 | 0.932 | 4.95E-19 |
| SFXN1 | 2.78E-23 | -0.26901 | 0.346 | 0.655 | 5.02E-19 |
| PUF60 | 3.86E-23 | -0.37193 | 0.433 | 0.74 | 6.96E-19 |
| CAPZB | 4.62E-23 | -0.34524 | 0.835 | 0.973 | 8.33E-19 |
| SRP9 | 5.16E-23 | -0.29532 | 0.455 | 0.759 | 9.31E-19 |
| MYL12A | 5.38E-23 | -0.37273 | 0.966 | 0.997 | 9.69E-19 |
| NR4A3 | 5.50E-23 | 0.5069 | 0.589 | 0.324 | 9.92E-19 |
| SP110 | 5.87E-23 | -0.31703 | 0.612 | 0.859 | 1.06E-18 |
| ATP5L | 6.86E-23 | -0.27504 | 0.802 | 0.948 | 1.24E-18 |
| IFI44 | 7.19E-23 | -0.52797 | 0.26 | 0.544 | 1.30E-18 |
| OAS3 | 7.89E-23 | -0.3014 | 0.198 | 0.488 | 1.42E-18 |
| PER1 | 8.47E-23 | 0.515609 | 0.709 | 0.47 | 1.53E-18 |
| FANCL | 9.36E-23 | -0.31027 | 0.123 | 0.414 | 1.69E-18 |
| ACTG1 | 1.03E-22 | -0.41394 | 0.997 | 1 | 1.85E-18 |
| PSMB10 | 1.21E-22 | -0.30421 | 0.687 | 0.932 | 2.19E-18 |
| S100A4 | 1.29E-22 | -0.36944 | 0.804 | 0.959 | 2.33E-18 |
| TSTA3 | 1.40E-22 | -0.36827 | 0.229 | 0.526 | 2.53E-18 |
| ANXA1 | 1.96E-22 | 0.866877 | 0.969 | 0.969 | 3.53E-18 |
| DCTN2 | 2.30E-22 | -0.36955 | 0.282 | 0.583 | 4.15E-18 |
| IFI6 | 2.43E-22 | -0.43688 | 0.416 | 0.717 | 4.38E-18 |
| TXN2 | 2.44E-22 | -0.35379 | 0.243 | 0.543 | 4.39E-18 |
| TRAF5 | 2.55E-22 | -0.50284 | 0.455 | 0.727 | 4.59E-18 |
| SMARCE1 | 3.14E-22 | -0.32548 | 0.489 | 0.776 | 5.66E-18 |
| FOSB | 3.59E-22 | 0.621439 | 0.849 | 0.682 | 6.47E-18 |
| RGS2 | 4.00E-22 | 0.602592 | 0.953 | 0.819 | 7.22E-18 |
| GRSF1 | 4.32E-22 | -0.30493 | 0.338 | 0.665 | 7.78E-18 |
| SNRPC | 4.71E-22 | -0.27315 | 0.251 | 0.55 | 8.49E-18 |
| ABRACL | 5.29E-22 | -0.3135 | 0.486 | 0.786 | 9.53E-18 |
| CTSB | 5.56E-22 | -0.27635 | 0.38 | 0.682 | 1.00E-17 |
| IER2 | 7.48E-22 | 0.74671 | 0.919 | 0.844 | 1.35E-17 |
| METTL3 | 7.90E-22 | -0.41954 | 0.145 | 0.424 | 1.42E-17 |
| MIR497HG | 8.39E-22 | -0.27906 | 0.391 | 0.698 | 1.51E-17 |
| PSMA4 | 8.84E-22 | -0.34825 | 0.506 | 0.786 | 1.59E-17 |
| AKAP5 | 9.28E-22 | -0.3212 | 0.123 | 0.4 | 1.67E-17 |
| ARPC1B | 1.13E-21 | -0.34341 | 0.807 | 0.956 | 2.04E-17 |
| PRDX3 | 1.38E-21 | -0.34046 | 0.45 | 0.722 | 2.48E-17 |
| PSMA5 | 1.50E-21 | -0.38088 | 0.578 | 0.826 | 2.71E-17 |
| TWF2 | 1.51E-21 | -0.35335 | 0.338 | 0.613 | 2.72E-17 |
| SLAMF1 | 2.08E-21 | -0.35126 | 0.271 | 0.572 | 3.75E-17 |
| UBASH3A | 3.27E-21 | -0.25797 | 0.285 | 0.578 | 5.89E-17 |
| ITGA1 | 3.33E-21 | -0.34761 | 0.631 | 0.88 | 5.99E-17 |
| TOX | 3.37E-21 | -0.37646 | 0.427 | 0.721 | 6.08E-17 |
| SAMD9 | 3.40E-21 | -0.34279 | 0.427 | 0.719 | 6.13E-17 |
| HLA-DRA | 3.50E-21 | -0.5703 | 0.785 | 0.943 | 6.31E-17 |
| ZYX | 3.91E-21 | -0.29378 | 0.5 | 0.824 | 7.05E-17 |
| SKA2 | 3.92E-21 | -0.28364 | 0.251 | 0.54 | 7.07E-17 |
| POLR2G | 4.19E-21 | -0.3207 | 0.455 | 0.751 | 7.54E-17 |
| RBCK1 | 4.29E-21 | -0.30483 | 0.391 | 0.67 | 7.74E-17 |
| TMBIM4 | 4.32E-21 | -0.32664 | 0.623 | 0.856 | 7.80E-17 |
| MTHFD1 | 5.64E-21 | -0.41622 | 0.257 | 0.545 | 1.02E-16 |
| KLF6 | 8.29E-21 | 0.506393 | 0.986 | 0.985 | 1.49E-16 |
| ACTR3 | 8.91E-21 | -0.40417 | 0.869 | 0.976 | 1.61E-16 |
| CCR5 | 1.12E-20 | -0.5154 | 0.226 | 0.49 | 2.03E-16 |
| AFAP1L2 | 1.14E-20 | -0.48667 | 0.126 | 0.385 | 2.05E-16 |
| ATP5A1 | 1.18E-20 | -0.40759 | 0.807 | 0.944 | 2.14E-16 |
| ATP6V0E2 | 1.21E-20 | -0.35282 | 0.313 | 0.605 | 2.19E-16 |
| TMPO | 1.23E-20 | -0.36776 | 0.363 | 0.646 | 2.21E-16 |
| COX5A | 1.36E-20 | -0.28832 | 0.458 | 0.745 | 2.44E-16 |
| LAT | 1.69E-20 | -0.35717 | 0.721 | 0.933 | 3.04E-16 |
| REEP5 | 1.87E-20 | -0.35558 | 0.709 | 0.907 | 3.36E-16 |
| NUDT21 | 1.90E-20 | -0.27344 | 0.282 | 0.58 | 3.43E-16 |
| TIMMDC1 | 2.06E-20 | -0.34261 | 0.218 | 0.503 | 3.72E-16 |
| ITM2A | 2.21E-20 | -0.44668 | 0.791 | 0.96 | 3.99E-16 |
| WARS | 2.30E-20 | -0.47907 | 0.246 | 0.512 | 4.15E-16 |
| LSP1 | 2.84E-20 | -0.36277 | 0.966 | 0.996 | 5.12E-16 |
| PDCD1 | 3.18E-20 | -0.51028 | 0.469 | 0.718 | 5.73E-16 |
| RTCB | 3.57E-20 | -0.32567 | 0.221 | 0.5 | 6.44E-16 |
| MCM3 | 4.62E-20 | -0.48746 | 0.223 | 0.503 | 8.33E-16 |
| SQOR | 4.72E-20 | -0.34739 | 0.391 | 0.678 | 8.50E-16 |
| GDI2 | 5.01E-20 | -0.37442 | 0.589 | 0.82 | 9.04E-16 |
| DAXX | 6.95E-20 | -0.3181 | 0.313 | 0.607 | 1.25E-15 |
| ITGB7 | 7.12E-20 | -0.35891 | 0.573 | 0.825 | 1.28E-15 |
| RASAL3 | 7.51E-20 | -0.27294 | 0.57 | 0.83 | 1.35E-15 |
| NMI | 7.55E-20 | -0.31731 | 0.254 | 0.533 | 1.36E-15 |
| PDIA6 | 7.66E-20 | -0.37087 | 0.628 | 0.847 | 1.38E-15 |
| RER1 | 7.95E-20 | -0.31142 | 0.369 | 0.637 | 1.43E-15 |
| CCDC69 | 8.77E-20 | -0.27454 | 0.341 | 0.632 | 1.58E-15 |
| TBC1D10C | 9.00E-20 | -0.34755 | 0.668 | 0.898 | 1.62E-15 |
| SLF1 | 9.03E-20 | -0.31866 | 0.408 | 0.697 | 1.63E-15 |
| TUBB | 9.03E-20 | -0.67935 | 0.612 | 0.853 | 1.63E-15 |
| CDK4 | 9.31E-20 | -0.27041 | 0.246 | 0.529 | 1.68E-15 |
| GMFG | 9.71E-20 | -0.25895 | 0.665 | 0.894 | 1.75E-15 |
| GBP3 | 1.26E-19 | -0.31345 | 0.277 | 0.561 | 2.26E-15 |
| RNASEK-C17orf49 | 1.32E-19 | -0.33797 | 0.799 | 0.951 | 2.38E-15 |
| EZR | 1.37E-19 | 0.600375 | 0.983 | 0.988 | 2.46E-15 |
| TNFRSF18 | 1.56E-19 | -0.313 | 0.179 | 0.442 | 2.81E-15 |
| MKI67 | 1.62E-19 | -0.4974 | 0.053 | 0.278 | 2.91E-15 |
| TMSB4X | 1.64E-19 | -0.3256 | 1 | 1 | 2.96E-15 |
| MAPKAPK3 | 1.65E-19 | -0.25409 | 0.182 | 0.457 | 2.98E-15 |
| TLR9 | 2.05E-19 | -0.26917 | 0.285 | 0.553 | 3.69E-15 |
| LBR | 2.12E-19 | -0.3218 | 0.612 | 0.835 | 3.81E-15 |
| IL32 | 2.25E-19 | -0.29439 | 1 | 1 | 4.05E-15 |
| SHISA5 | 2.27E-19 | -0.37646 | 0.718 | 0.916 | 4.10E-15 |
| LPXN | 2.30E-19 | -0.32913 | 0.712 | 0.921 | 4.15E-15 |
| H2AFY | 2.33E-19 | -0.27266 | 0.274 | 0.554 | 4.21E-15 |
| TOB1 | 2.37E-19 | 0.899364 | 0.916 | 0.815 | 4.27E-15 |
| PSMA6 | 2.73E-19 | -0.32812 | 0.556 | 0.832 | 4.92E-15 |
| SENP3-EIF4A1 | 2.86E-19 | 0.400731 | 0.997 | 0.989 | 5.15E-15 |
| C17orf62 | 3.24E-19 | -0.28484 | 0.514 | 0.795 | 5.84E-15 |
| PRKAG1 | 3.43E-19 | -0.31357 | 0.221 | 0.493 | 6.19E-15 |
| APOL6 | 3.75E-19 | -0.27418 | 0.464 | 0.736 | 6.76E-15 |
| DBNL | 4.06E-19 | -0.32541 | 0.444 | 0.711 | 7.32E-15 |
| SPNS1 | 4.26E-19 | -0.25139 | 0.578 | 0.852 | 7.69E-15 |
| TSPYL2 | 5.21E-19 | 0.863267 | 0.723 | 0.564 | 9.40E-15 |
| EIF2AK1 | 5.50E-19 | -0.29281 | 0.265 | 0.528 | 9.91E-15 |
| GBP4 | 5.71E-19 | -0.33563 | 0.503 | 0.761 | 1.03E-14 |
| TIGIT | 6.07E-19 | -0.45261 | 0.754 | 0.908 | 1.09E-14 |
| ARHGEF6 | 6.15E-19 | -0.25679 | 0.338 | 0.624 | 1.11E-14 |
| MRPS16 | 6.52E-19 | -0.26739 | 0.363 | 0.645 | 1.17E-14 |
| CBX5 | 6.52E-19 | -0.32305 | 0.246 | 0.509 | 1.18E-14 |
| MYO7A | 6.58E-19 | -0.42841 | 0.193 | 0.433 | 1.19E-14 |
| C2orf68 | 6.70E-19 | -0.28369 | 0.243 | 0.507 | 1.21E-14 |
| NR4A1 | 6.71E-19 | 1.041884 | 0.64 | 0.423 | 1.21E-14 |
| MR1 | 7.05E-19 | -0.30441 | 0.235 | 0.509 | 1.27E-14 |
| UTS2 | 7.16E-19 | -0.30242 | 0.26 | 0.526 | 1.29E-14 |
| WDR77 | 7.18E-19 | -0.36974 | 0.075 | 0.311 | 1.29E-14 |
| PDE4DIP | 7.20E-19 | -0.30233 | 0.444 | 0.73 | 1.30E-14 |
| TRAFD1 | 7.29E-19 | -0.28929 | 0.271 | 0.552 | 1.31E-14 |
| C14orf119 | 7.85E-19 | -0.31278 | 0.265 | 0.544 | 1.41E-14 |
| VPS25 | 9.36E-19 | -0.29449 | 0.142 | 0.397 | 1.69E-14 |
| PSMB8-AS1 | 9.70E-19 | -0.25947 | 0.785 | 0.957 | 1.75E-14 |
| ENTPD1 | 1.06E-18 | -0.64723 | 0.411 | 0.651 | 1.91E-14 |
| SLFN5 | 1.11E-18 | -0.3934 | 0.492 | 0.724 | 2.01E-14 |
| SEPT7 | 1.13E-18 | -0.32817 | 0.849 | 0.97 | 2.04E-14 |
| CTSC | 1.29E-18 | -0.39309 | 0.838 | 0.959 | 2.32E-14 |
| TYMS | 1.44E-18 | -0.54024 | 0.059 | 0.285 | 2.60E-14 |
| XIST | 1.76E-18 | -0.53824 | 0.553 | 0.735 | 3.17E-14 |
| CHMP4A | 2.12E-18 | -0.26487 | 0.466 | 0.724 | 3.81E-14 |
| DUSP2 | 2.69E-18 | 0.658214 | 0.955 | 0.917 | 4.84E-14 |
| NUBP2 | 2.80E-18 | -0.26095 | 0.184 | 0.442 | 5.06E-14 |
| PARP1 | 3.07E-18 | -0.27923 | 0.553 | 0.793 | 5.54E-14 |
| UBE2V1 | 3.28E-18 | -0.29734 | 0.684 | 0.88 | 5.92E-14 |
| SPN | 3.58E-18 | -0.28085 | 0.598 | 0.815 | 6.45E-14 |
| ZNF683 | 3.73E-18 | -0.65629 | 0.385 | 0.634 | 6.72E-14 |
| GADD45B | 3.73E-18 | 0.65475 | 0.598 | 0.411 | 6.73E-14 |
| TMEM189-UBE2V1 | 4.17E-18 | -0.29699 | 0.707 | 0.894 | 7.52E-14 |
| GZMH | 5.97E-18 | -0.37055 | 0.832 | 0.969 | 1.08E-13 |
| CTSA | 7.64E-18 | -0.34644 | 0.547 | 0.8 | 1.38E-13 |
| SAE1 | 7.82E-18 | -0.25393 | 0.274 | 0.547 | 1.41E-13 |
| TAP1 | 8.33E-18 | -0.28786 | 0.885 | 0.977 | 1.50E-13 |
| TIAM1 | 8.73E-18 | -0.32141 | 0.145 | 0.382 | 1.57E-13 |
| IFI16 | 8.81E-18 | -0.32514 | 0.849 | 0.961 | 1.59E-13 |
| CD3D | 1.02E-17 | -0.29884 | 0.994 | 1 | 1.84E-13 |
| FAM46C | 1.04E-17 | 0.615212 | 0.816 | 0.647 | 1.88E-13 |
| CISH | 1.18E-17 | -0.42519 | 0.24 | 0.509 | 2.13E-13 |
| SUPT16H | 1.19E-17 | -0.25452 | 0.447 | 0.712 | 2.14E-13 |
| MCM4 | 1.22E-17 | -0.48294 | 0.075 | 0.298 | 2.20E-13 |
| SF3B3 | 1.31E-17 | -0.25251 | 0.363 | 0.636 | 2.37E-13 |
| SDCBP | 1.34E-17 | 0.635606 | 0.913 | 0.856 | 2.41E-13 |
| WDR1 | 1.49E-17 | -0.35458 | 0.796 | 0.918 | 2.68E-13 |
| GIMAP4 | 1.52E-17 | -0.33208 | 0.676 | 0.873 | 2.74E-13 |
| CTSW | 1.61E-17 | -0.50929 | 0.872 | 0.922 | 2.90E-13 |
| KDM6B | 1.81E-17 | 0.397294 | 0.522 | 0.306 | 3.27E-13 |
| IRF2 | 1.84E-17 | -0.31414 | 0.397 | 0.64 | 3.31E-13 |
| PPP4C | 1.90E-17 | -0.27837 | 0.374 | 0.642 | 3.42E-13 |
| TSG101 | 2.01E-17 | -0.31861 | 0.316 | 0.586 | 3.62E-13 |
| MRPS18B | 2.24E-17 | -0.33081 | 0.237 | 0.487 | 4.03E-13 |
| CLK1 | 2.45E-17 | 0.570938 | 0.953 | 0.947 | 4.42E-13 |
| SNX14 | 2.51E-17 | -0.28406 | 0.302 | 0.563 | 4.53E-13 |
| ADORA2A | 2.61E-17 | -0.27214 | 0.243 | 0.501 | 4.70E-13 |
| CNN2 | 2.63E-17 | -0.30123 | 0.779 | 0.951 | 4.73E-13 |
| NME1 | 2.64E-17 | -0.25485 | 0.137 | 0.373 | 4.76E-13 |
| IL2RB | 2.74E-17 | -0.32533 | 0.791 | 0.952 | 4.94E-13 |
| MYL6 | 2.87E-17 | -0.28704 | 0.969 | 0.999 | 5.17E-13 |
| TALDO1 | 3.02E-17 | -0.25396 | 0.391 | 0.655 | 5.45E-13 |
| TANGO2 | 3.11E-17 | -0.36075 | 0.198 | 0.443 | 5.60E-13 |
| HADHA | 3.17E-17 | -0.34901 | 0.483 | 0.707 | 5.71E-13 |
| SP140 | 3.34E-17 | -0.26045 | 0.749 | 0.915 | 6.02E-13 |
| TNFRSF9 | 3.50E-17 | -0.64276 | 0.444 | 0.683 | 6.31E-13 |
| GLRX3 | 3.52E-17 | -0.27594 | 0.243 | 0.5 | 6.34E-13 |
| XRCC5 | 3.80E-17 | -0.3415 | 0.757 | 0.927 | 6.84E-13 |
| MCM5 | 4.18E-17 | -0.42091 | 0.26 | 0.508 | 7.54E-13 |
| SMC3 | 4.48E-17 | -0.25161 | 0.497 | 0.752 | 8.07E-13 |
| GPR171 | 4.57E-17 | -0.29806 | 0.637 | 0.857 | 8.25E-13 |
| KIAA0391 | 4.67E-17 | -0.32532 | 0.668 | 0.88 | 8.42E-13 |
| USO1 | 4.68E-17 | -0.28298 | 0.254 | 0.515 | 8.44E-13 |
| DCLRE1C | 5.51E-17 | -0.25632 | 0.162 | 0.408 | 9.94E-13 |
| IL7R | 5.84E-17 | 0.855884 | 0.832 | 0.796 | 1.05E-12 |
| TUBA4A | 6.40E-17 | 0.820994 | 0.905 | 0.91 | 1.15E-12 |
| MDM2 | 7.09E-17 | -0.30615 | 0.374 | 0.619 | 1.28E-12 |
| SUB1 | 7.16E-17 | -0.28299 | 0.874 | 0.971 | 1.29E-12 |
| SMC2 | 8.77E-17 | -0.27518 | 0.092 | 0.319 | 1.58E-12 |
| TIPRL | 9.13E-17 | -0.30553 | 0.246 | 0.497 | 1.65E-12 |
| JMJD4 | 9.32E-17 | -0.29283 | 0.106 | 0.328 | 1.68E-12 |
| FDFT1 | 1.07E-16 | -0.3194 | 0.483 | 0.717 | 1.94E-12 |
| CSRP1 | 1.11E-16 | -0.29722 | 0.24 | 0.491 | 2.00E-12 |
| CD3G | 1.14E-16 | -0.2839 | 0.916 | 0.985 | 2.05E-12 |
| LOC284454 | 1.15E-16 | 0.604218 | 0.601 | 0.394 | 2.08E-12 |
| CD101 | 1.16E-16 | -0.42713 | 0.285 | 0.529 | 2.09E-12 |
| SCAMP2 | 1.18E-16 | -0.30609 | 0.539 | 0.782 | 2.13E-12 |
| YWHAB | 1.19E-16 | -0.29259 | 0.919 | 0.982 | 2.14E-12 |
| GPI | 1.25E-16 | -0.28261 | 0.592 | 0.831 | 2.25E-12 |
| HLA-DRB5 | 1.27E-16 | -0.43216 | 0.869 | 0.967 | 2.29E-12 |
| TPM4 | 1.28E-16 | -0.36813 | 0.656 | 0.87 | 2.31E-12 |
| LINC-PINT | 1.34E-16 | 0.568951 | 0.905 | 0.852 | 2.41E-12 |
| CTNNA1 | 1.41E-16 | -0.41683 | 0.145 | 0.371 | 2.54E-12 |
| PSMB7 | 1.52E-16 | -0.27901 | 0.455 | 0.698 | 2.75E-12 |
| MRE11 | 1.55E-16 | -0.28613 | 0.131 | 0.362 | 2.80E-12 |
| SLC27A2 | 1.72E-16 | -0.31083 | 0.17 | 0.418 | 3.10E-12 |
| GOLIM4 | 1.81E-16 | -0.34776 | 0.117 | 0.335 | 3.26E-12 |
| FDPS | 1.86E-16 | -0.27374 | 0.33 | 0.589 | 3.35E-12 |
| LAG3 | 1.88E-16 | -0.25094 | 0.539 | 0.79 | 3.38E-12 |
| SIK1 | 1.90E-16 | 0.306422 | 0.5 | 0.272 | 3.43E-12 |
| SMARCAD1 | 2.07E-16 | -0.28513 | 0.268 | 0.515 | 3.73E-12 |
| SLC35A4 | 2.35E-16 | -0.26574 | 0.285 | 0.549 | 4.23E-12 |
| HAPLN3 | 2.55E-16 | -0.26629 | 0.142 | 0.372 | 4.60E-12 |
| GALM | 3.20E-16 | -0.28857 | 0.494 | 0.734 | 5.77E-12 |
| TSIX | 3.45E-16 | -0.41422 | 0.486 | 0.686 | 6.22E-12 |
| UNC13D | 3.78E-16 | -0.27587 | 0.324 | 0.567 | 6.82E-12 |
| NAA60 | 4.22E-16 | -0.29082 | 0.215 | 0.444 | 7.61E-12 |
| SPECC1L | 5.02E-16 | -0.304 | 0.436 | 0.665 | 9.05E-12 |
| PIGC | 5.92E-16 | -0.32356 | 0.19 | 0.432 | 1.07E-11 |
| GTF2A2 | 6.07E-16 | -0.25082 | 0.282 | 0.54 | 1.09E-11 |
| ERAP1 | 6.57E-16 | -0.29597 | 0.542 | 0.764 | 1.18E-11 |
| PHB | 7.61E-16 | -0.30203 | 0.282 | 0.516 | 1.37E-11 |
| RAB3GAP1 | 8.15E-16 | -0.3278 | 0.344 | 0.578 | 1.47E-11 |
| ETNK1 | 8.20E-16 | -0.31457 | 0.64 | 0.854 | 1.48E-11 |
| MAP4K1 | 9.87E-16 | -0.26665 | 0.656 | 0.861 | 1.78E-11 |
| DCTD | 9.96E-16 | -0.25288 | 0.257 | 0.505 | 1.80E-11 |
| OXNAD1 | 1.05E-15 | -0.278 | 0.606 | 0.798 | 1.89E-11 |
| ALOX5AP | 1.07E-15 | -0.43436 | 0.765 | 0.928 | 1.93E-11 |
| ACOT7 | 1.17E-15 | -0.34783 | 0.103 | 0.314 | 2.12E-11 |
| BTG1 | 1.20E-15 | 0.407426 | 0.989 | 0.996 | 2.17E-11 |
| RPL13 | 1.32E-15 | 0.262401 | 0.997 | 0.999 | 2.38E-11 |
| ENOSF1 | 1.41E-15 | -0.41426 | 0.184 | 0.418 | 2.53E-11 |
| LLPH | 1.41E-15 | -0.25903 | 0.709 | 0.903 | 2.55E-11 |
| ETV1 | 1.43E-15 | -0.30402 | 0.089 | 0.295 | 2.58E-11 |
| GOLGA7 | 1.49E-15 | -0.2669 | 0.427 | 0.683 | 2.68E-11 |
| RARRES3 | 1.50E-15 | -0.29827 | 0.793 | 0.944 | 2.70E-11 |
| COMMD8 | 1.62E-15 | -0.32088 | 0.324 | 0.57 | 2.91E-11 |
| PSMC3 | 1.75E-15 | -0.27146 | 0.413 | 0.662 | 3.16E-11 |
| RBM26 | 1.97E-15 | -0.25846 | 0.263 | 0.484 | 3.55E-11 |
| RHOC | 2.14E-15 | -0.2958 | 0.439 | 0.679 | 3.87E-11 |
| ZNF22 | 2.53E-15 | -0.26306 | 0.254 | 0.499 | 4.55E-11 |
| RHOA | 2.56E-15 | -0.32377 | 0.95 | 0.989 | 4.62E-11 |
| TMED3 | 2.70E-15 | -0.27194 | 0.131 | 0.354 | 4.86E-11 |
| ZFP36L2 | 2.87E-15 | 0.592519 | 0.992 | 0.971 | 5.17E-11 |
| SLC25A11 | 2.96E-15 | -0.29391 | 0.249 | 0.478 | 5.33E-11 |
| IFI35 | 2.99E-15 | -0.29453 | 0.349 | 0.593 | 5.38E-11 |
| ALG5 | 3.24E-15 | -0.28828 | 0.204 | 0.433 | 5.84E-11 |
| ATF4 | 3.64E-15 | 0.557073 | 0.818 | 0.748 | 6.57E-11 |
| TUBA1A | 3.69E-15 | 0.657914 | 0.883 | 0.792 | 6.64E-11 |
| AGTRAP | 4.03E-15 | -0.27511 | 0.184 | 0.422 | 7.26E-11 |
| SEMA4A | 4.03E-15 | -0.37816 | 0.176 | 0.397 | 7.26E-11 |
| CYTH4 | 4.15E-15 | -0.26917 | 0.433 | 0.668 | 7.48E-11 |
| TM7SF3 | 4.62E-15 | -0.31857 | 0.134 | 0.347 | 8.32E-11 |
| PSMD14 | 4.73E-15 | -0.34121 | 0.349 | 0.584 | 8.52E-11 |
| SRP68 | 4.78E-15 | -0.25373 | 0.204 | 0.45 | 8.63E-11 |
| RFX5 | 4.82E-15 | -0.27204 | 0.209 | 0.441 | 8.69E-11 |
| NFKBIA | 4.87E-15 | 0.631324 | 0.955 | 0.932 | 8.77E-11 |
| CD84 | 4.97E-15 | -0.31713 | 0.584 | 0.792 | 8.96E-11 |
| HERC6 | 5.02E-15 | -0.31335 | 0.14 | 0.359 | 9.04E-11 |
| HLA-DMB | 5.68E-15 | -0.33186 | 0.335 | 0.578 | 1.02E-10 |
| FANCI | 5.78E-15 | -0.2692 | 0.249 | 0.488 | 1.04E-10 |
| PABPC1 | 6.28E-15 | 0.478265 | 0.964 | 0.976 | 1.13E-10 |
| THYN1 | 6.57E-15 | -0.27526 | 0.254 | 0.491 | 1.18E-10 |
| CCL5 | 6.62E-15 | -0.30001 | 1 | 1 | 1.19E-10 |
| PDCD6 | 6.78E-15 | -0.27512 | 0.399 | 0.613 | 1.22E-10 |
| RNF167 | 7.19E-15 | -0.26816 | 0.601 | 0.81 | 1.30E-10 |
| SLC38A2 | 7.42E-15 | 0.640562 | 0.894 | 0.855 | 1.34E-10 |
| PDIA4 | 8.86E-15 | -0.2838 | 0.352 | 0.596 | 1.60E-10 |
| TRMT2A | 8.89E-15 | -0.26109 | 0.168 | 0.393 | 1.60E-10 |
| CAT | 8.98E-15 | -0.32596 | 0.257 | 0.483 | 1.62E-10 |
| SEPT1 | 9.36E-15 | -0.27196 | 0.844 | 0.957 | 1.69E-10 |
| C12orf75 | 1.05E-14 | -0.26455 | 0.587 | 0.834 | 1.89E-10 |
| LCK | 1.07E-14 | -0.28038 | 0.95 | 0.989 | 1.93E-10 |
| PRMT2 | 1.07E-14 | -0.26157 | 0.601 | 0.812 | 1.94E-10 |
| NUSAP1 | 1.16E-14 | -0.34042 | 0.193 | 0.416 | 2.09E-10 |
| CCDC141 | 1.22E-14 | -0.28506 | 0.332 | 0.561 | 2.20E-10 |
| TNIP3 | 1.59E-14 | -0.30677 | 0.48 | 0.686 | 2.87E-10 |
| COPB2 | 1.59E-14 | -0.26888 | 0.363 | 0.601 | 2.87E-10 |
| GIMAP7 | 1.65E-14 | -0.2949 | 0.673 | 0.856 | 2.98E-10 |
| COG4 | 1.71E-14 | -0.30077 | 0.243 | 0.469 | 3.09E-10 |
| IFITM1 | 1.77E-14 | -0.38 | 0.941 | 0.983 | 3.19E-10 |
| MCTP2 | 1.94E-14 | -0.37489 | 0.279 | 0.495 | 3.50E-10 |
| IFRD1 | 2.03E-14 | 0.663866 | 0.788 | 0.644 | 3.65E-10 |
| TUBB2A | 2.03E-14 | 0.44104 | 0.291 | 0.13 | 3.67E-10 |
| CD74 | 2.24E-14 | -0.36867 | 0.983 | 0.998 | 4.04E-10 |
| STT3A | 2.26E-14 | -0.32625 | 0.26 | 0.478 | 4.07E-10 |
| GALNT1 | 2.51E-14 | -0.25293 | 0.232 | 0.461 | 4.52E-10 |
| CD55 | 2.95E-14 | 0.72387 | 0.682 | 0.547 | 5.32E-10 |
| RSAD2 | 3.10E-14 | -0.40507 | 0.106 | 0.307 | 5.59E-10 |
| SMC1A | 3.24E-14 | -0.27727 | 0.408 | 0.653 | 5.83E-10 |
| ZBED2 | 3.58E-14 | -0.46157 | 0.115 | 0.308 | 6.46E-10 |
| UBL7 | 3.70E-14 | -0.27006 | 0.218 | 0.449 | 6.67E-10 |
| PYURF | 3.72E-14 | -0.27309 | 0.508 | 0.738 | 6.71E-10 |
| SETX | 3.95E-14 | -0.27183 | 0.634 | 0.82 | 7.12E-10 |
| SHMT2 | 4.30E-14 | -0.29362 | 0.173 | 0.387 | 7.74E-10 |
| DNAJB6 | 4.66E-14 | 0.441491 | 0.835 | 0.757 | 8.40E-10 |
| HSD17B10 | 5.27E-14 | -0.28126 | 0.388 | 0.591 | 9.50E-10 |
| ZFAND5 | 5.85E-14 | 0.567674 | 0.827 | 0.701 | 1.06E-09 |
| MAD2L1 | 6.00E-14 | -0.25624 | 0.078 | 0.27 | 1.08E-09 |
| DENND2D | 6.06E-14 | -0.38275 | 0.587 | 0.777 | 1.09E-09 |
| UBB | 6.13E-14 | 0.397038 | 1 | 1 | 1.11E-09 |
| JUN | 6.22E-14 | 0.630784 | 0.913 | 0.873 | 1.12E-09 |
| EYA3 | 6.41E-14 | -0.26031 | 0.223 | 0.431 | 1.15E-09 |
| XPO1 | 6.46E-14 | -0.26155 | 0.313 | 0.546 | 1.16E-09 |
| CCND2 | 7.15E-14 | -0.33372 | 0.729 | 0.863 | 1.29E-09 |
| TCEAL8 | 7.32E-14 | -0.27521 | 0.084 | 0.271 | 1.32E-09 |
| TUBA1C | 7.82E-14 | 0.56399 | 0.676 | 0.546 | 1.41E-09 |
| PEF1 | 8.48E-14 | -0.28882 | 0.288 | 0.521 | 1.53E-09 |
| DYNLT1 | 8.80E-14 | -0.25145 | 0.528 | 0.726 | 1.59E-09 |
| PVRIG | 1.03E-13 | -0.25401 | 0.62 | 0.826 | 1.86E-09 |
| TRIM59 | 1.13E-13 | -0.27461 | 0.332 | 0.557 | 2.04E-09 |
| AMD1 | 1.17E-13 | 0.647725 | 0.743 | 0.634 | 2.11E-09 |
| MFF | 1.17E-13 | -0.25147 | 0.344 | 0.575 | 2.12E-09 |
| SLC9A3R1 | 1.19E-13 | -0.28954 | 0.693 | 0.873 | 2.15E-09 |
| HLA-DRB6 | 1.28E-13 | -0.33111 | 0.86 | 0.968 | 2.31E-09 |
| CDKN1A | 1.44E-13 | 0.534919 | 0.564 | 0.373 | 2.59E-09 |
| USB1 | 1.61E-13 | -0.25787 | 0.254 | 0.474 | 2.90E-09 |
| GART | 1.65E-13 | -0.27867 | 0.232 | 0.447 | 2.98E-09 |
| SAR1B | 1.70E-13 | -0.31663 | 0.271 | 0.484 | 3.07E-09 |
| FYN | 2.17E-13 | 0.548139 | 0.939 | 0.963 | 3.91E-09 |
| CASC4 | 2.18E-13 | -0.28591 | 0.366 | 0.582 | 3.93E-09 |
| SPPL2A | 2.35E-13 | -0.25889 | 0.43 | 0.645 | 4.23E-09 |
| NEU1 | 2.35E-13 | 0.697005 | 0.651 | 0.474 | 4.24E-09 |
| ITGB2 | 2.43E-13 | -0.28061 | 0.858 | 0.963 | 4.38E-09 |
| ENC1 | 2.61E-13 | 0.566753 | 0.271 | 0.121 | 4.71E-09 |
| MYO5B | 2.86E-13 | -0.34975 | 0.112 | 0.296 | 5.16E-09 |
| STMN1 | 2.97E-13 | -0.71716 | 0.324 | 0.561 | 5.35E-09 |
| TESPA1 | 3.36E-13 | -0.28548 | 0.254 | 0.467 | 6.06E-09 |
| ARL6IP5 | 3.73E-13 | -0.27138 | 0.874 | 0.969 | 6.72E-09 |
| PSME1 | 3.78E-13 | -0.25396 | 0.916 | 0.988 | 6.81E-09 |
| SNX1 | 3.93E-13 | -0.26774 | 0.408 | 0.623 | 7.09E-09 |
| RUNX2 | 3.95E-13 | -0.25999 | 0.265 | 0.466 | 7.13E-09 |
| EIF4A3 | 3.98E-13 | 0.571759 | 0.796 | 0.696 | 7.17E-09 |
| GABARAP | 4.19E-13 | -0.25798 | 0.793 | 0.933 | 7.55E-09 |
| CTLA4 | 4.53E-13 | -0.31043 | 0.413 | 0.615 | 8.17E-09 |
| LDHB | 4.76E-13 | -0.28607 | 0.858 | 0.963 | 8.58E-09 |
| UBE2F-SCLY | 4.79E-13 | -0.26079 | 0.31 | 0.53 | 8.64E-09 |
| PDE4B | 5.36E-13 | 0.515019 | 0.894 | 0.848 | 9.65E-09 |
| MLH1 | 8.21E-13 | -0.26176 | 0.201 | 0.403 | 1.48E-08 |
| CD164 | 8.32E-13 | -0.28393 | 0.802 | 0.945 | 1.50E-08 |
| EIF2B1 | 8.64E-13 | -0.27847 | 0.288 | 0.508 | 1.56E-08 |
| CD244 | 8.67E-13 | -0.27497 | 0.397 | 0.607 | 1.56E-08 |
| PLK3 | 8.69E-13 | 0.548137 | 0.589 | 0.414 | 1.57E-08 |
| IFI44L | 9.32E-13 | -0.44432 | 0.179 | 0.372 | 1.68E-08 |
| FYB1 | 1.02E-12 | -0.29547 | 0.955 | 0.994 | 1.84E-08 |
| HNRNPH1 | 1.08E-12 | 0.287192 | 0.994 | 1 | 1.95E-08 |
| HNRNPA2B1 | 1.10E-12 | -0.27419 | 0.941 | 0.991 | 1.98E-08 |
| VPS35 | 1.15E-12 | -0.25235 | 0.316 | 0.529 | 2.08E-08 |
| PEX11B | 1.17E-12 | -0.30341 | 0.106 | 0.287 | 2.11E-08 |
| TSPAN17 | 1.66E-12 | -0.25201 | 0.117 | 0.299 | 2.99E-08 |
| CRYBG1 | 1.67E-12 | 0.818048 | 0.704 | 0.617 | 3.02E-08 |
| HSPA1L | 1.71E-12 | 1.063157 | 0.687 | 0.578 | 3.08E-08 |
| TMEM106C | 1.76E-12 | -0.31566 | 0.235 | 0.443 | 3.17E-08 |
| VCAM1 | 2.39E-12 | -0.63289 | 0.422 | 0.589 | 4.31E-08 |
| DUT | 2.54E-12 | -0.25251 | 0.277 | 0.488 | 4.58E-08 |
| SNHG12 | 2.62E-12 | 0.513488 | 0.712 | 0.563 | 4.73E-08 |
| WDR7 | 2.68E-12 | -0.28862 | 0.182 | 0.383 | 4.83E-08 |
| SMC4 | 2.76E-12 | -0.27769 | 0.511 | 0.724 | 4.97E-08 |
| EWSR1 | 2.98E-12 | -0.25374 | 0.793 | 0.936 | 5.38E-08 |
| FOSL2 | 3.21E-12 | 0.479102 | 0.617 | 0.432 | 5.78E-08 |
| JMJD6 | 3.70E-12 | 0.591449 | 0.746 | 0.647 | 6.66E-08 |
| NT5C2 | 3.90E-12 | -0.25602 | 0.277 | 0.473 | 7.03E-08 |
| LIMD2 | 4.37E-12 | -0.27617 | 0.737 | 0.925 | 7.88E-08 |
| UBLCP1 | 6.61E-12 | -0.27158 | 0.346 | 0.548 | 1.19E-07 |
| TXNDC15 | 6.93E-12 | -0.26583 | 0.209 | 0.4 | 1.25E-07 |
| ANXA2 | 6.94E-12 | -0.26652 | 0.642 | 0.846 | 1.25E-07 |
| ARL4A | 7.64E-12 | 0.450215 | 0.439 | 0.276 | 1.38E-07 |
| CD247 | 8.76E-12 | -0.26741 | 0.723 | 0.879 | 1.58E-07 |
| UAP1 | 8.99E-12 | 0.570929 | 0.48 | 0.305 | 1.62E-07 |
| HLA-DPA1 | 1.16E-11 | -0.2974 | 0.955 | 0.993 | 2.09E-07 |
| RGCC | 1.24E-11 | 0.502544 | 0.81 | 0.702 | 2.24E-07 |
| EOMES | 1.65E-11 | -0.27974 | 0.377 | 0.587 | 2.98E-07 |
| HSPD1 | 1.73E-11 | 0.856525 | 0.877 | 0.828 | 3.13E-07 |
| CHMP1B | 1.83E-11 | 0.683874 | 0.709 | 0.618 | 3.30E-07 |
| TPM3 | 2.28E-11 | -0.25347 | 0.964 | 0.996 | 4.11E-07 |
| SAT1 | 2.51E-11 | 0.628073 | 0.902 | 0.93 | 4.52E-07 |
| ADGRG1 | 2.64E-11 | -0.32447 | 0.302 | 0.489 | 4.76E-07 |
| MX1 | 4.66E-11 | -0.32599 | 0.405 | 0.609 | 8.41E-07 |
| PTGER4 | 6.65E-11 | 0.664862 | 0.793 | 0.728 | 1.20E-06 |
| CD200R1 | 7.33E-11 | -0.35877 | 0.187 | 0.364 | 1.32E-06 |
| TIPARP | 7.81E-11 | 0.532679 | 0.561 | 0.405 | 1.41E-06 |
| SCCPDH | 8.28E-11 | -0.2888 | 0.151 | 0.325 | 1.49E-06 |
| HLA-DRB1 | 9.55E-11 | -0.284 | 0.897 | 0.984 | 1.72E-06 |
| MFSD11 | 1.05E-10 | 0.38327 | 0.897 | 0.896 | 1.89E-06 |
| RPL13A | 1.05E-10 | 0.290911 | 1 | 0.999 | 1.90E-06 |
| FAM53C | 1.39E-10 | 0.577799 | 0.539 | 0.382 | 2.50E-06 |
| PMAIP1 | 2.02E-10 | 0.423874 | 0.682 | 0.522 | 3.63E-06 |
| KIAA1551 | 2.03E-10 | -0.31588 | 0.869 | 0.95 | 3.65E-06 |
| TNPO1 | 2.05E-10 | -0.30154 | 0.302 | 0.491 | 3.70E-06 |
| FAM177A1 | 2.30E-10 | 0.562663 | 0.682 | 0.581 | 4.14E-06 |
| ZC3H12A | 2.33E-10 | 0.60421 | 0.609 | 0.483 | 4.20E-06 |
| CCDC173 | 2.71E-10 | 0.331298 | 0.263 | 0.126 | 4.89E-06 |
| KLRC4 | 2.79E-10 | -0.3083 | 0.492 | 0.66 | 5.02E-06 |
| CD44 | 2.92E-10 | 0.34671 | 0.992 | 0.989 | 5.27E-06 |
| KLF3 | 4.12E-10 | 0.472516 | 0.291 | 0.158 | 7.43E-06 |
| HEXIM1 | 4.87E-10 | 0.401792 | 0.528 | 0.369 | 8.79E-06 |
| CPSF3 | 5.26E-10 | -0.26097 | 0.173 | 0.345 | 9.48E-06 |
| ACSL4 | 5.30E-10 | -0.25864 | 0.226 | 0.394 | 9.56E-06 |
| RPS27 | 7.21E-10 | 0.269668 | 0.869 | 0.832 | 1.30E-05 |
| STAT4 | 9.01E-10 | 0.414996 | 0.93 | 0.918 | 1.62E-05 |
| SQLE | 1.24E-09 | -0.28451 | 0.173 | 0.328 | 2.24E-05 |
| IDI2-AS1 | 1.89E-09 | 0.351273 | 0.592 | 0.488 | 3.40E-05 |
| LITAF | 2.01E-09 | 0.499948 | 0.922 | 0.934 | 3.62E-05 |
| CAPG | 2.22E-09 | -0.2764 | 0.52 | 0.713 | 4.00E-05 |
| PFKFB3 | 3.09E-09 | 0.545495 | 0.701 | 0.58 | 5.57E-05 |
| MAFF | 3.46E-09 | 0.498267 | 0.441 | 0.301 | 6.23E-05 |
| CNOT6L | 4.43E-09 | 0.522323 | 0.88 | 0.868 | 7.99E-05 |
| AVPI1 | 6.13E-09 | 0.282593 | 0.254 | 0.13 | 0.000111 |
| ZEB2 | 6.85E-09 | 0.551106 | 0.785 | 0.741 | 0.000123 |
| KCNQ1OT1 | 8.46E-09 | 0.436083 | 0.374 | 0.236 | 0.000152 |
| KRT86 | 8.49E-09 | -0.4971 | 0.14 | 0.28 | 0.000153 |
| SKIL | 8.70E-09 | 0.630508 | 0.821 | 0.77 | 0.000157 |
| VRK1 | 9.08E-09 | -0.26295 | 0.198 | 0.359 | 0.000164 |
| SRSF3 | 1.23E-08 | 0.365941 | 0.936 | 0.97 | 0.000222 |
| DENND4A | 1.36E-08 | 0.548399 | 0.67 | 0.557 | 0.000245 |
| DDIT4 | 1.54E-08 | 0.633915 | 0.855 | 0.902 | 0.000278 |
| MARCKSL1 | 1.54E-08 | 0.360306 | 0.321 | 0.196 | 0.000278 |
| EIF5 | 1.94E-08 | 0.37771 | 0.911 | 0.906 | 0.000349 |
| DDX3X | 2.51E-08 | 0.485266 | 0.947 | 0.96 | 0.000453 |
| SNHG1 | 2.93E-08 | 0.645633 | 0.522 | 0.413 | 0.000528 |
| PPP1CB | 3.33E-08 | 0.569122 | 0.927 | 0.95 | 0.000601 |
| HSPA6 | 3.67E-08 | 0.941127 | 0.355 | 0.217 | 0.000662 |
| ZMPSTE24 | 4.12E-08 | -0.26995 | 0.221 | 0.37 | 0.000743 |
| CTBS | 6.25E-08 | -0.25797 | 0.257 | 0.418 | 0.001127 |
| WDR47 | 6.63E-08 | 0.434615 | 0.349 | 0.224 | 0.001196 |
| IDI1 | 6.74E-08 | 0.544368 | 0.83 | 0.812 | 0.001216 |
| ANKRD37 | 7.45E-08 | 0.450433 | 0.464 | 0.326 | 0.001342 |
| KLF2 | 1.30E-07 | 0.318289 | 0.346 | 0.224 | 0.002338 |
| TMEM2 | 1.49E-07 | 0.635104 | 0.751 | 0.709 | 0.002691 |
| TAGLN2 | 1.73E-07 | 0.328753 | 0.969 | 0.976 | 0.003123 |
| RNF125 | 1.84E-07 | 0.590123 | 0.704 | 0.678 | 0.003326 |
| SYTL3 | 2.95E-07 | 0.519653 | 0.796 | 0.813 | 0.005314 |
| NXF1 | 3.94E-07 | 0.478929 | 0.746 | 0.68 | 0.00711 |
| BCAS2 | 4.40E-07 | 0.438368 | 0.704 | 0.658 | 0.007937 |
| ATF3 | 5.34E-07 | 0.462223 | 0.383 | 0.249 | 0.009622 |
| NFKBIZ | 7.15E-07 | 0.58672 | 0.712 | 0.622 | 0.012894 |
| RALGAPA1 | 7.56E-07 | 0.314021 | 0.634 | 0.555 | 0.013632 |
| MCM7 | 1.03E-06 | -0.3514 | 0.369 | 0.518 | 0.018538 |
| SATB1 | 1.09E-06 | 0.446136 | 0.494 | 0.39 | 0.019619 |
| EIF4A2 | 1.25E-06 | 0.337303 | 0.969 | 0.975 | 0.022617 |
| LRRN3 | 1.27E-06 | -0.31162 | 0.154 | 0.274 | 0.022977 |
| ATP2B1 | 1.57E-06 | 0.573085 | 0.634 | 0.592 | 0.028286 |
| RGS1 | 1.60E-06 | 0.33677 | 0.983 | 0.988 | 0.028835 |
| HSPA5 | 1.61E-06 | 0.274575 | 0.941 | 0.95 | 0.029013 |
| EGR1 | 1.62E-06 | 0.664324 | 0.547 | 0.46 | 0.02922 |
| LTB | 1.64E-06 | -0.26902 | 0.427 | 0.589 | 0.029483 |
| DCTN6 | 1.93E-06 | 0.535675 | 0.628 | 0.55 | 0.034746 |
| BRD2 | 1.97E-06 | 0.439752 | 0.911 | 0.894 | 0.035526 |
| CD69 | 2.02E-06 | 0.450507 | 0.983 | 0.98 | 0.036421 |
| PRMT9 | 2.03E-06 | 0.339933 | 0.439 | 0.31 | 0.036659 |
| CHD1 | 2.09E-06 | 0.462259 | 0.807 | 0.79 | 0.037638 |
| MYLIP | 2.11E-06 | 0.45156 | 0.447 | 0.342 | 0.038063 |
| DEDD2 | 2.15E-06 | 0.486532 | 0.659 | 0.58 | 0.038792 |
| IRF4 | 2.32E-06 | 0.612127 | 0.642 | 0.526 | 0.041837 |
| HSPA1B | 2.33E-06 | 0.761349 | 0.732 | 0.67 | 0.041941 |
| FTL | 2.53E-06 | 0.256125 | 0.997 | 0.998 | 0.045559 |
| TXNIP | 2.62E-06 | -0.38481 | 0.933 | 0.948 | 0.047225 |
| SNHG16 | 2.95E-06 | 0.381129 | 0.458 | 0.368 | 0.053237 |
| PIK3R1 | 3.09E-06 | 0.521454 | 0.768 | 0.762 | 0.055663 |
| LOC100130476 | 4.27E-06 | 0.441387 | 0.626 | 0.571 | 0.076903 |
| DENND2C | 5.02E-06 | 0.425838 | 0.687 | 0.679 | 0.090506 |
| HERPUD1 | 5.16E-06 | 0.447832 | 0.88 | 0.915 | 0.093096 |
| DNAJB9 | 5.76E-06 | 0.450298 | 0.578 | 0.507 | 0.103876 |
| KPNA2 | 6.81E-06 | 0.429454 | 0.684 | 0.608 | 0.122845 |
| PNRC1 | 8.34E-06 | 0.417931 | 0.885 | 0.891 | 0.150333 |
| GLA | 8.59E-06 | 0.540506 | 0.436 | 0.33 | 0.154927 |
| SMAD7 | 9.22E-06 | 0.408717 | 0.335 | 0.234 | 0.166274 |
| DDX3Y | 9.65E-06 | 0.477301 | 0.271 | 0.179 | 0.173982 |
| SLC25A4 | 1.01E-05 | 0.37733 | 0.341 | 0.239 | 0.182633 |
| RASGEF1B | 1.08E-05 | 0.338438 | 0.506 | 0.385 | 0.194713 |
| VPS37B | 1.08E-05 | 0.264111 | 0.578 | 0.498 | 0.194748 |
| SC5D | 1.22E-05 | 0.494214 | 0.472 | 0.364 | 0.219981 |
| TSC22D2 | 1.58E-05 | 0.28218 | 0.402 | 0.299 | 0.284919 |
| SESN2 | 1.60E-05 | 0.358947 | 0.282 | 0.187 | 0.289218 |
| ELF1 | 1.89E-05 | 0.340245 | 0.933 | 0.963 | 0.341082 |
| TMEM71 | 1.92E-05 | 0.411816 | 0.455 | 0.357 | 0.346899 |
| MPP7 | 1.93E-05 | 0.301804 | 0.268 | 0.18 | 0.347078 |
| GTF2B | 2.26E-05 | 0.453223 | 0.696 | 0.665 | 0.407709 |
| SLC16A1 | 2.68E-05 | 0.377643 | 0.453 | 0.352 | 0.482504 |
| SNHG15 | 2.87E-05 | 0.457239 | 0.469 | 0.375 | 0.516729 |
| ZNF10 | 3.06E-05 | 0.311484 | 0.341 | 0.245 | 0.552396 |
| CCNH | 3.11E-05 | 0.448237 | 0.763 | 0.759 | 0.561073 |
| SERPINB9 | 3.57E-05 | 0.383473 | 0.916 | 0.932 | 0.642909 |
| B4GALT1 | 3.71E-05 | 0.36242 | 0.765 | 0.792 | 0.668427 |
| HSPE1-MOB4 | 3.97E-05 | 0.431062 | 0.866 | 0.887 | 0.715215 |
| OAT | 4.21E-05 | 0.323676 | 0.615 | 0.533 | 0.759382 |
| BCL6 | 5.14E-05 | 0.406273 | 0.324 | 0.234 | 0.927273 |
| HSPE1 | 5.22E-05 | 0.519645 | 0.757 | 0.757 | 0.94042 |
| SLC7A5 | 5.25E-05 | 0.324935 | 0.673 | 0.618 | 0.94647 |
| STK4 | 7.30E-05 | 0.286212 | 0.947 | 0.967 | 1 |
| NCL | 7.90E-05 | 0.255071 | 0.939 | 0.969 | 1 |
| PDCD4 | 8.49E-05 | 0.299644 | 0.941 | 0.969 | 1 |
| TC2N | 9.00E-05 | 0.406744 | 0.679 | 0.636 | 1 |
| ZFAND2A | 0.000128 | 0.368945 | 0.422 | 0.332 | 1 |
| RANBP2 | 0.000134 | 0.456261 | 0.76 | 0.727 | 1 |
| C3orf58 | 0.000151 | 0.301363 | 0.318 | 0.226 | 1 |
| REL | 0.000155 | 0.387033 | 0.763 | 0.716 | 1 |
| SGK1 | 0.000156 | 0.506992 | 0.332 | 0.242 | 1 |
| F11R | 0.000162 | 0.297827 | 0.385 | 0.55 | 1 |
| CCL4L1 | 0.000167 | -0.43365 | 0.835 | 0.915 | 1 |
| IFNGR1 | 0.000169 | 0.418568 | 0.553 | 0.491 | 1 |
| RBM39 | 0.00018 | 0.307715 | 0.972 | 0.989 | 1 |
| ITPRIP | 0.000187 | 0.447549 | 0.525 | 0.432 | 1 |
| SYAP1 | 0.000193 | 0.470377 | 0.634 | 0.598 | 1 |
| ZC3HAV1 | 0.00027 | 0.39744 | 0.913 | 0.917 | 1 |
| MCL1 | 0.000277 | 0.302279 | 0.919 | 0.946 | 1 |
| TOB2 | 0.000293 | 0.433029 | 0.598 | 0.558 | 1 |
| SELENOK | 0.000299 | 0.304374 | 0.76 | 0.79 | 1 |
| NASP | 0.000317 | 0.33274 | 0.799 | 0.774 | 1 |
| NUFIP2 | 0.000381 | 0.306875 | 0.547 | 0.468 | 1 |
| DNAJB4 | 0.000438 | 0.404793 | 0.36 | 0.271 | 1 |
| PPP1R15B | 0.000473 | 0.478692 | 0.534 | 0.469 | 1 |
| CCNL1 | 0.000502 | 0.348965 | 0.874 | 0.925 | 1 |
| MGAT4A | 0.000541 | 0.387082 | 0.693 | 0.675 | 1 |
| MAP3K8 | 0.000544 | 0.396408 | 0.528 | 0.452 | 1 |
| ETF1 | 0.000578 | 0.308888 | 0.67 | 0.584 | 1 |
| USP36 | 0.000594 | 0.41111 | 0.623 | 0.577 | 1 |
| ANKRD28 | 0.000658 | 0.427611 | 0.556 | 0.489 | 1 |
| PIP4K2A | 0.000727 | 0.377109 | 0.916 | 0.968 | 1 |
| SRSF6 | 0.000788 | 0.390397 | 0.872 | 0.869 | 1 |
| NOP58 | 0.000809 | 0.407902 | 0.88 | 0.9 | 1 |
| APBA2 | 0.000943 | 0.393741 | 0.419 | 0.354 | 1 |
| ARID5A | 0.000989 | 0.44084 | 0.743 | 0.767 | 1 |
| SRSF5 | 0.001001 | 0.26777 | 0.969 | 0.98 | 1 |
| SAFB2 | 0.00108 | 0.355613 | 0.69 | 0.666 | 1 |
| SLC2A3 | 0.001117 | 0.358861 | 0.888 | 0.886 | 1 |
| AUTS2 | 0.001276 | 0.338031 | 0.372 | 0.293 | 1 |
| ITGA5 | 0.001718 | 0.40602 | 0.304 | 0.241 | 1 |
| POLR2A | 0.001834 | 0.318738 | 0.729 | 0.744 | 1 |
| PIM2 | 0.002039 | 0.43439 | 0.821 | 0.839 | 1 |
| PGAP1 | 0.002063 | 0.303417 | 0.268 | 0.196 | 1 |
| GAS5 | 0.002098 | 0.318883 | 0.779 | 0.777 | 1 |
| ARID5B | 0.002396 | 0.339535 | 0.779 | 0.792 | 1 |
| SOCS3 | 0.002428 | 0.307056 | 0.385 | 0.321 | 1 |
| TRA2A | 0.003057 | 0.311236 | 0.891 | 0.95 | 1 |
| PPP2R5C | 0.003177 | 0.348403 | 0.885 | 0.924 | 1 |
| SPDYA | 0.003413 | 0.262223 | 0.573 | 0.55 | 1 |
| LYAR | 0.003484 | 0.273029 | 0.534 | 0.494 | 1 |
| RAP2B | 0.003565 | 0.332407 | 0.528 | 0.482 | 1 |
| HNRNPUL1 | 0.003581 | 0.285847 | 0.704 | 0.692 | 1 |
| GNAS | 0.003611 | 0.357419 | 0.936 | 0.95 | 1 |
| RNF138 | 0.003766 | 0.481064 | 0.556 | 0.511 | 1 |
| JMJD1C | 0.003789 | 0.406567 | 0.838 | 0.845 | 1 |
| DUSP6 | 0.003866 | 0.357147 | 0.279 | 0.22 | 1 |
| TAGAP | 0.004158 | 0.447673 | 0.799 | 0.834 | 1 |
| NXT1 | 0.004257 | 0.25523 | 0.411 | 0.351 | 1 |
| INSIG1 | 0.004714 | 0.350262 | 0.503 | 0.445 | 1 |
| CHORDC1 | 0.004853 | 0.542141 | 0.662 | 0.632 | 1 |
| BRE-AS1 | 0.005004 | 0.42711 | 0.425 | 0.387 | 1 |
| GABARAPL1 | 0.005833 | 0.350751 | 0.76 | 0.764 | 1 |
| CCR7 | 0.006367 | 0.64132 | 0.567 | 0.582 | 1 |
| FUS | 0.006385 | 0.255128 | 0.866 | 0.917 | 1 |
| EML4 | 0.006883 | 0.337162 | 0.897 | 0.925 | 1 |
| SMCHD1 | 0.007296 | 0.311482 | 0.872 | 0.9 | 1 |
| FKBP4 | 0.00755 | 0.43422 | 0.394 | 0.345 | 1 |
| RELL1 | 0.008357 | 0.375557 | 0.48 | 0.422 | 1 |
| DDIT3 | 0.008588 | 0.370047 | 0.595 | 0.564 | 1 |
| ADRB2 | 0.0089 | 0.469236 | 0.363 | 0.321 | 1 |
| HECA | 0.009018 | 0.265531 | 0.475 | 0.413 | 1 |
| UFSP2 | 0.009279 | 0.302705 | 0.5 | 0.445 | 1 |
| SBDS | 0.009364 | 0.341937 | 0.64 | 0.642 | 1 |
| BBIP1 | 0.009529 | 0.30109 | 0.81 | 0.836 | 1 |
| UBE2B | 0.010036 | 0.270905 | 0.754 | 0.745 | 1 |
| DNAJA4 | 0.011873 | 0.420707 | 0.296 | 0.244 | 1 |
| ODC1 | 0.012496 | 0.266551 | 0.634 | 0.602 | 1 |
| SORL1 | 0.012601 | 0.471007 | 0.447 | 0.421 | 1 |
| SLC25A3 | 0.012639 | 0.262822 | 0.855 | 0.936 | 1 |
| TCP1 | 0.014012 | 0.412435 | 0.81 | 0.848 | 1 |
| HSPB1 | 0.014873 | 0.437246 | 0.48 | 0.609 | 1 |
| RNF19A | 0.017733 | 0.386131 | 0.86 | 0.925 | 1 |
| MXD1 | 0.018976 | 0.269589 | 0.307 | 0.253 | 1 |
| IQGAP2 | 0.019097 | 0.401681 | 0.595 | 0.611 | 1 |
| ERN1 | 0.01979 | 0.306183 | 0.411 | 0.371 | 1 |
| SNHG5 | 0.021714 | 0.354025 | 0.615 | 0.603 | 1 |
| IRF1 | 0.022452 | 0.262642 | 0.93 | 0.959 | 1 |
| NOP56 | 0.025006 | 0.288364 | 0.704 | 0.707 | 1 |
| RUNX3 | 0.026322 | 0.287825 | 0.807 | 0.866 | 1 |
| TMEM39A | 0.027323 | 0.328703 | 0.402 | 0.359 | 1 |
| NR1D2 | 0.029288 | 0.333161 | 0.251 | 0.206 | 1 |
| TNF | 0.03003 | 0.289502 | 0.399 | 0.359 | 1 |
| DUSP10 | 0.031439 | 0.251291 | 0.701 | 0.675 | 1 |
| DNTTIP2 | 0.03299 | 0.324141 | 0.707 | 0.707 | 1 |
| BAG3 | 0.033432 | 0.392423 | 0.274 | 0.22 | 1 |
| GRPEL1 | 0.033735 | 0.279097 | 0.455 | 0.415 | 1 |
| FKBP5 | 0.039673 | 0.262174 | 0.589 | 0.727 | 1 |
| DDX24 | 0.040951 | 0.325626 | 0.874 | 0.918 | 1 |
| PARP8 | 0.041158 | 0.454676 | 0.796 | 0.874 | 1 |
| APLP2 | 0.045673 | 0.264792 | 0.478 | 0.445 | 1 |
| RAB9A | 0.045804 | 0.298714 | 0.374 | 0.33 | 1 |
| RNF168 | 0.04928 | 0.38484 | 0.559 | 0.536 | 1 |
| EHD1 | 0.04969 | 0.342472 | 0.676 | 0.721 | 1 |
| TBCC | 0.052407 | 0.301724 | 0.648 | 0.658 | 1 |
| S1PR1 | 0.05281 | 0.262266 | 0.299 | 0.27 | 1 |
| ISCA1 | 0.0558 | 0.260646 | 0.45 | 0.415 | 1 |
| NFE2L2 | 0.059265 | 0.347826 | 0.659 | 0.657 | 1 |
| PTP4A1 | 0.060366 | 0.320249 | 0.735 | 0.755 | 1 |
| RCHY1 | 0.061881 | 0.303852 | 0.299 | 0.263 | 1 |
| ZBTB1 | 0.063402 | 0.299791 | 0.729 | 0.737 | 1 |
| RORA | 0.063413 | 0.311799 | 0.679 | 0.693 | 1 |
| CHD2 | 0.065102 | 0.263127 | 0.883 | 0.91 | 1 |
| NUP98 | 0.065512 | 0.398076 | 0.637 | 0.664 | 1 |
| ATF7IP | 0.078406 | 0.279278 | 0.905 | 0.944 | 1 |
| RASA3 | 0.080913 | 0.288972 | 0.391 | 0.364 | 1 |
| PRNP | 0.081243 | 0.335515 | 0.645 | 0.672 | 1 |
| AHNAK | 0.084343 | 0.357594 | 0.86 | 0.92 | 1 |
| GPBP1 | 0.086986 | 0.26546 | 0.835 | 0.864 | 1 |
| WDR74 | 0.089781 | 0.410888 | 0.494 | 0.499 | 1 |
| RBMX | 0.090815 | 0.251012 | 0.81 | 0.836 | 1 |
| MORF4L2 | 0.091176 | 0.298532 | 0.656 | 0.645 | 1 |
| PPP1R10 | 0.094416 | 0.263943 | 0.567 | 0.554 | 1 |
| TSPYL1 | 0.094486 | 0.377765 | 0.729 | 0.788 | 1 |
| ISG20 | 0.096888 | 0.255881 | 0.726 | 0.82 | 1 |
| HBP1 | 0.09848 | 0.372547 | 0.634 | 0.644 | 1 |
| ARF4 | 0.099831 | 0.250026 | 0.69 | 0.716 | 1 |
| SENP7 | 0.101778 | 0.269588 | 0.497 | 0.59 | 1 |
| AHR | 0.106426 | 0.407614 | 0.517 | 0.509 | 1 |
| GLUL | 0.109036 | 0.455192 | 0.506 | 0.479 | 1 |
| TBC1D15 | 0.11288 | 0.293201 | 0.542 | 0.521 | 1 |
| CLDND1 | 0.117703 | 0.349624 | 0.757 | 0.8 | 1 |
| FOXP1 | 0.123093 | 0.310158 | 0.74 | 0.749 | 1 |
| ALG13 | 0.129857 | 0.308243 | 0.528 | 0.518 | 1 |
| PER2 | 0.135615 | 0.255592 | 0.265 | 0.234 | 1 |
| PRKCQ | 0.138207 | 0.260637 | 0.634 | 0.738 | 1 |
| NAA50 | 0.141519 | 0.421081 | 0.743 | 0.805 | 1 |
| KDM2A | 0.144192 | 0.323426 | 0.631 | 0.668 | 1 |
| ARL4C | 0.150895 | 0.287423 | 0.701 | 0.735 | 1 |
| GNL3 | 0.156289 | 0.314833 | 0.525 | 0.531 | 1 |
| GSPT1 | 0.161326 | 0.301168 | 0.612 | 0.612 | 1 |
| MED29 | 0.18215 | 0.362482 | 0.458 | 0.458 | 1 |
| KCNA3 | 0.1843 | 0.319891 | 0.397 | 0.395 | 1 |
| CACYBP | 0.215209 | 0.34825 | 0.712 | 0.745 | 1 |
| FAM129A | 0.228699 | 0.311487 | 0.721 | 0.776 | 1 |
| EEF2 | 0.233733 | 0.267546 | 0.709 | 0.748 | 1 |
| ODF2L | 0.253609 | 0.273411 | 0.601 | 0.614 | 1 |
| MAPK1IP1L | 0.268749 | 0.267546 | 0.83 | 0.891 | 1 |
| SPOCK2 | 0.270866 | 0.28232 | 0.746 | 0.81 | 1 |
| CD28 | 0.278038 | 0.2594 | 0.45 | 0.463 | 1 |
| PLIN2 | 0.284814 | 0.286721 | 0.626 | 0.647 | 1 |
| ELP2 | 0.293477 | 0.302508 | 0.5 | 0.509 | 1 |
| SAR1A | 0.320459 | 0.265811 | 0.83 | 0.875 | 1 |
| CDK16 | 0.323677 | 0.291216 | 0.304 | 0.302 | 1 |
| EPC1 | 0.357021 | 0.251284 | 0.659 | 0.696 | 1 |
| PDE4D | 0.360959 | 0.259807 | 0.662 | 0.707 | 1 |
| WSB1 | 0.381483 | 0.284768 | 0.821 | 0.908 | 1 |
| ANKRD12 | 0.386312 | 0.275135 | 0.832 | 0.908 | 1 |
| STIP1 | 0.394535 | 0.269111 | 0.656 | 0.731 | 1 |
| PAF1 | 0.396172 | 0.256969 | 0.466 | 0.474 | 1 |
| MRPL10 | 0.420052 | 0.258511 | 0.525 | 0.611 | 1 |
| MED21 | 0.426361 | 0.264367 | 0.344 | 0.335 | 1 |
| TGIF1 | 0.446937 | 0.290682 | 0.38 | 0.388 | 1 |
| ZNF394 | 0.450137 | 0.277628 | 0.425 | 0.443 | 1 |
| SLC2A1 | 0.457342 | 0.263199 | 0.534 | 0.547 | 1 |
| TRA2B | 0.466015 | 0.252436 | 0.684 | 0.761 | 1 |
| PNP | 0.473498 | 0.254622 | 0.539 | 0.543 | 1 |
| CDC14A | 0.504668 | 0.316736 | 0.589 | 0.611 | 1 |
| PRDM2 | 0.611902 | 0.263121 | 0.601 | 0.694 | 1 |
| RHOH | 0.613896 | 0.294956 | 0.835 | 0.916 | 1 |
| SQSTM1 | 0.665162 | 0.264933 | 0.838 | 0.9 | 1 |
| ICAM1 | 0.686584 | 0.26937 | 0.338 | 0.346 | 1 |
| MBOAT1 | 0.689507 | 0.30772 | 0.321 | 0.333 | 1 |
| NCOA2 | 0.690636 | 0.323997 | 0.461 | 0.495 | 1 |
| SLC1A5 | 0.696992 | 0.258159 | 0.483 | 0.521 | 1 |
| OFD1 | 0.707981 | 0.30168 | 0.645 | 0.713 | 1 |
| IL18RAP | 0.730389 | 0.282985 | 0.352 | 0.377 | 1 |
| SLFN11 | 0.744737 | 0.341783 | 0.388 | 0.421 | 1 |
| STAT5A | 0.80883 | 0.251949 | 0.553 | 0.599 | 1 |
| CDC42EP3 | 0.809458 | 0.344429 | 0.486 | 0.53 | 1 |
| GPR132 | 0.814465 | 0.293189 | 0.478 | 0.502 | 1 |
| PPP1R16B | 0.834627 | 0.278344 | 0.682 | 0.718 | 1 |
| PTGES3 | 0.868807 | 0.264041 | 0.869 | 0.919 | 1 |
| PPP1R2 | 0.897084 | 0.267887 | 0.782 | 0.845 | 1 |
| ELMSAN1 | 0.901666 | 0.375115 | 0.598 | 0.657 | 1 |
| DOK2 | 0.917556 | 0.292649 | 0.707 | 0.77 | 1 |
| GOLGB1 | 0.948295 | 0.250416 | 0.679 | 0.727 | 1 |
| EP300 | 0.988155 | 0.3917 | 0.522 | 0.554 | 1 |
| NABP1 | 0.989985 | 0.2812 | 0.48 | 0.543 | 1 |
| **Cluster2** | | | | | |
| GeneSymbol | p_val | logFC | pct.1 | pct.2 | p_val_adj |
| CXCR4 | 5.39E-18 | 0.475089 | 1 | 0.99 | 9.73E-14 |
| TNFAIP3 | 9.28E-14 | 0.429526 | 0.984 | 0.977 | 1.67E-09 |
| PDCD1 | 5.49E-13 | -0.60023 | 0.443 | 0.693 | 9.90E-09 |
| ZFP36 | 7.04E-13 | 0.408758 | 0.979 | 0.972 | 1.27E-08 |
| FAM46C | 1.19E-12 | 0.434165 | 0.865 | 0.66 | 2.14E-08 |
| PTPN6 | 2.83E-12 | -0.52549 | 0.505 | 0.726 | 5.10E-08 |
| DNAJB1 | 3.26E-12 | 0.426621 | 0.984 | 0.917 | 5.88E-08 |
| CXCL13 | 1.76E-11 | -0.88776 | 0.469 | 0.697 | 3.17E-07 |
| NR4A2 | 3.14E-11 | 0.40972 | 0.948 | 0.776 | 5.65E-07 |
| BTG1 | 5.42E-11 | 0.348308 | 0.995 | 0.994 | 9.77E-07 |
| DUSP1 | 8.64E-11 | 0.425611 | 0.974 | 0.944 | 1.56E-06 |
| JUNB | 1.94E-10 | 0.328728 | 0.979 | 0.94 | 3.50E-06 |
| ZNF331 | 3.84E-10 | 0.300502 | 0.87 | 0.693 | 6.92E-06 |
| FCRL3 | 4.48E-10 | -0.48581 | 0.104 | 0.326 | 8.08E-06 |
| ACTG1 | 4.64E-10 | -0.34504 | 1 | 0.999 | 8.36E-06 |
| NR4A3 | 7.13E-10 | 0.423949 | 0.573 | 0.356 | 1.29E-05 |
| GIMAP6 | 3.33E-09 | -0.39458 | 0.297 | 0.536 | 6.00E-05 |
| SARAF | 9.42E-09 | 0.254215 | 1 | 0.997 | 0.00017 |
| SEMA4A | 1.05E-08 | -0.42498 | 0.161 | 0.373 | 0.00019 |
| SAMHD1 | 1.40E-08 | -0.33476 | 0.375 | 0.615 | 0.000252 |
| LMNA | 1.71E-08 | 0.348844 | 0.615 | 0.429 | 0.000309 |
| MYADM | 1.76E-08 | 0.365052 | 0.823 | 0.669 | 0.000317 |
| CCR1 | 2.07E-08 | -0.3996 | 0.161 | 0.367 | 0.000373 |
| NR4A1 | 2.19E-08 | 0.326626 | 0.646 | 0.447 | 0.000394 |
| CSNK1G1 | 2.66E-08 | -0.29081 | 0.359 | 0.575 | 0.000479 |
| TSC22D3 | 2.98E-08 | 0.475502 | 0.984 | 0.972 | 0.000537 |
| SIRPG | 3.70E-08 | -0.26262 | 0.552 | 0.765 | 0.000667 |
| UBC | 4.15E-08 | 0.263305 | 1 | 1 | 0.000748 |
| OAS1 | 4.44E-08 | -0.35234 | 0.193 | 0.404 | 0.000801 |
| ENOSF1 | 4.46E-08 | -0.50924 | 0.193 | 0.391 | 0.000804 |
| ITM2A | 4.80E-08 | -0.43186 | 0.88 | 0.928 | 0.000866 |
| TOX | 6.89E-08 | -0.35757 | 0.495 | 0.679 | 0.001243 |
| LINC-PINT | 7.06E-08 | 0.350216 | 0.885 | 0.86 | 0.001272 |
| YPEL5 | 7.37E-08 | 0.311507 | 0.958 | 0.907 | 0.001328 |
| RGCC | 8.45E-08 | 0.320449 | 0.87 | 0.706 | 0.001523 |
| TNFRSF1A | 8.52E-08 | -0.34583 | 0.188 | 0.379 | 0.001536 |
| ETV1 | 9.91E-08 | -0.34034 | 0.094 | 0.271 | 0.001786 |
| SQOR | 1.00E-07 | -0.28871 | 0.417 | 0.642 | 0.001807 |
| FOS | 1.14E-07 | 0.268441 | 0.932 | 0.831 | 0.002058 |
| DNAJA1 | 1.17E-07 | 0.411586 | 0.969 | 0.909 | 0.002113 |
| CREM | 1.19E-07 | 0.429649 | 0.901 | 0.834 | 0.002142 |
| DNAJB9 | 1.31E-07 | 0.404477 | 0.667 | 0.503 | 0.002364 |
| HSPH1 | 1.84E-07 | 0.310308 | 0.875 | 0.778 | 0.003316 |
| BTG2 | 2.03E-07 | 0.341386 | 0.875 | 0.774 | 0.003657 |
| MTCH2 | 2.26E-07 | -0.32138 | 0.26 | 0.468 | 0.004069 |
| FUT8 | 2.79E-07 | -0.25901 | 0.354 | 0.54 | 0.005027 |
| CISH | 2.83E-07 | -0.31235 | 0.26 | 0.475 | 0.005108 |
| PPP1R15A | 4.47E-07 | 0.275072 | 0.927 | 0.84 | 0.008051 |
| SAMD9L | 4.56E-07 | -0.26343 | 0.505 | 0.688 | 0.008226 |
| CD27 | 6.64E-07 | -0.37423 | 0.661 | 0.833 | 0.011972 |
| TYMS | 7.45E-07 | -0.51949 | 0.099 | 0.254 | 0.013427 |
| HSP90AB1 | 9.82E-07 | 0.273621 | 1 | 0.992 | 0.0177 |
| PLSCR1 | 1.05E-06 | -0.30738 | 0.359 | 0.543 | 0.018858 |
| IFI16 | 1.08E-06 | -0.25012 | 0.87 | 0.946 | 0.019557 |
| SNRNP27 | 1.16E-06 | -0.25538 | 0.349 | 0.546 | 0.020914 |
| XAF1 | 1.19E-06 | -0.34682 | 0.474 | 0.655 | 0.021415 |
| CD2BP2 | 1.24E-06 | -0.26268 | 0.375 | 0.57 | 0.022434 |
| GIMAP4 | 1.31E-06 | -0.30054 | 0.693 | 0.848 | 0.023669 |
| EZH2 | 1.64E-06 | -0.25402 | 0.276 | 0.483 | 0.02961 |
| DTX3L | 1.68E-06 | -0.39378 | 0.328 | 0.501 | 0.030337 |
| PDE4B | 1.72E-06 | 0.380727 | 0.896 | 0.853 | 0.030949 |
| IKZF3 | 1.86E-06 | -0.33933 | 0.802 | 0.917 | 0.03347 |
| CAP1 | 1.99E-06 | -0.28878 | 0.974 | 0.961 | 0.035849 |
| CMTM6 | 2.06E-06 | -0.36123 | 0.661 | 0.779 | 0.037063 |
| SRSF2 | 2.19E-06 | 0.283644 | 0.969 | 0.975 | 0.039516 |
| KLF6 | 2.25E-06 | 0.298279 | 0.995 | 0.984 | 0.040558 |
| MCM5 | 2.35E-06 | -0.38158 | 0.281 | 0.477 | 0.042384 |
| SP100 | 2.49E-06 | -0.27694 | 0.901 | 0.94 | 0.044903 |
| DOCK2 | 2.81E-06 | -0.2779 | 0.568 | 0.744 | 0.050698 |
| ATP6V1G2-DDX39B | 2.98E-06 | -0.25666 | 0.557 | 0.716 | 0.053658 |
| CD27-AS1 | 3.20E-06 | -0.30502 | 0.609 | 0.783 | 0.057677 |
| CCNA2 | 3.47E-06 | -0.3051 | 0.151 | 0.303 | 0.062487 |
| PKM | 3.76E-06 | -0.38611 | 0.854 | 0.917 | 0.067729 |
| HSPA8 | 4.36E-06 | 0.325085 | 1 | 0.999 | 0.078589 |
| PSMB8 | 4.75E-06 | -0.30867 | 0.828 | 0.922 | 0.085625 |
| RGS1 | 4.98E-06 | 0.365782 | 0.984 | 0.987 | 0.089786 |
| SLFN5 | 5.08E-06 | -0.31533 | 0.547 | 0.691 | 0.091672 |
| DGKA | 6.14E-06 | -0.25814 | 0.557 | 0.716 | 0.110757 |
| MYO5B | 6.37E-06 | -0.2568 | 0.125 | 0.273 | 0.114867 |
| EXOSC9 | 6.83E-06 | -0.27773 | 0.318 | 0.495 | 0.123102 |
| FTH1 | 6.95E-06 | 0.270696 | 1 | 0.999 | 0.125273 |
| EOMES | 7.01E-06 | -0.39521 | 0.406 | 0.559 | 0.126306 |
| ELF1 | 7.30E-06 | 0.276553 | 0.964 | 0.955 | 0.131637 |
| SLC43A3 | 7.61E-06 | -0.26488 | 0.177 | 0.338 | 0.137208 |
| SLC2A3 | 8.09E-06 | 0.399936 | 0.932 | 0.88 | 0.145934 |
| SPG21 | 8.49E-06 | -0.27292 | 0.281 | 0.454 | 0.153088 |
| PFKFB3 | 8.75E-06 | 0.441473 | 0.688 | 0.596 | 0.157758 |
| RPA2 | 8.88E-06 | -0.28711 | 0.443 | 0.615 | 0.160158 |
| MTHFD1 | 9.58E-06 | -0.26422 | 0.323 | 0.503 | 0.172742 |
| HNRNPR | 9.86E-06 | -0.29161 | 0.708 | 0.838 | 0.177767 |
| LOC284454 | 1.02E-05 | 0.283411 | 0.562 | 0.423 | 0.184076 |
| ANXA1 | 1.02E-05 | 0.34525 | 0.964 | 0.97 | 0.18468 |
| SRSF1 | 1.04E-05 | -0.2745 | 0.578 | 0.719 | 0.187957 |
| MIR155HG | 1.31E-05 | -0.31071 | 0.38 | 0.525 | 0.236522 |
| FOSB | 1.32E-05 | 0.337945 | 0.797 | 0.708 | 0.238853 |
| TUBA1A | 1.33E-05 | 0.26813 | 0.891 | 0.801 | 0.239035 |
| PSMA4 | 1.45E-05 | -0.29157 | 0.609 | 0.74 | 0.26159 |
| IFRD1 | 1.61E-05 | 0.295473 | 0.755 | 0.664 | 0.289809 |
| JUN | 2.24E-05 | 0.403906 | 0.901 | 0.879 | 0.403883 |
| HAVCR2 | 2.35E-05 | -0.3028 | 0.589 | 0.746 | 0.422831 |
| ILK | 2.55E-05 | -0.25096 | 0.505 | 0.654 | 0.460148 |
| DUSP2 | 2.76E-05 | 0.37105 | 0.948 | 0.922 | 0.497941 |
| LANCL1 | 2.77E-05 | -0.30248 | 0.198 | 0.345 | 0.499387 |
| TNFRSF9 | 3.01E-05 | -0.30349 | 0.458 | 0.654 | 0.542828 |
| LYST | 3.20E-05 | -0.25385 | 0.823 | 0.902 | 0.576202 |
| HLA-DQA1 | 3.39E-05 | -0.26743 | 0.521 | 0.688 | 0.611015 |
| TOB1 | 3.45E-05 | 0.330397 | 0.88 | 0.831 | 0.622218 |
| METTL3 | 3.47E-05 | -0.2553 | 0.229 | 0.381 | 0.626368 |
| DMTF1 | 3.48E-05 | -0.28895 | 0.562 | 0.701 | 0.627977 |
| TUBB | 3.76E-05 | -0.57293 | 0.693 | 0.815 | 0.677096 |
| CCL3 | 4.07E-05 | -0.3871 | 0.552 | 0.702 | 0.734043 |
| MCM7 | 4.24E-05 | -0.57512 | 0.344 | 0.504 | 0.764127 |
| CBX5 | 4.27E-05 | -0.29456 | 0.328 | 0.468 | 0.769027 |
| FKBP1A | 4.40E-05 | -0.30941 | 0.781 | 0.872 | 0.792919 |
| BRD2 | 4.41E-05 | 0.317421 | 0.911 | 0.896 | 0.795507 |
| RPS6KA1 | 4.44E-05 | -0.256 | 0.318 | 0.476 | 0.799998 |
| NFKBIZ | 4.48E-05 | 0.419029 | 0.75 | 0.627 | 0.807411 |
| FANCI | 4.76E-05 | -0.30316 | 0.307 | 0.453 | 0.858945 |
| HARS2 | 4.79E-05 | -0.25169 | 0.167 | 0.317 | 0.862826 |
| STMN1 | 5.00E-05 | -0.8243 | 0.38 | 0.527 | 0.900808 |
| NOP58 | 5.16E-05 | 0.31231 | 0.911 | 0.893 | 0.930456 |
| NBN | 6.28E-05 | -0.29176 | 0.344 | 0.488 | 1 |
| PIK3R1 | 6.31E-05 | 0.415145 | 0.812 | 0.757 | 1 |
| ACTR3 | 6.61E-05 | -0.26759 | 0.932 | 0.955 | 1 |
| ACAT1 | 8.61E-05 | -0.26224 | 0.229 | 0.371 | 1 |
| JMJD6 | 9.18E-05 | 0.279832 | 0.745 | 0.658 | 1 |
| FAM53C | 9.21E-05 | 0.278271 | 0.536 | 0.4 | 1 |
| RGS2 | 0.000111 | 0.250954 | 0.917 | 0.839 | 1 |
| RBBP4 | 0.000121 | -0.26191 | 0.599 | 0.748 | 1 |
| ARID5B | 0.000127 | 0.420963 | 0.818 | 0.785 | 1 |
| SNHG16 | 0.000132 | 0.254939 | 0.484 | 0.375 | 1 |
| XIAP | 0.000137 | -0.28427 | 0.25 | 0.383 | 1 |
| CD83 | 0.000179 | 0.287307 | 0.479 | 0.357 | 1 |
| HSP90AA1 | 0.000188 | 0.336221 | 0.995 | 0.997 | 1 |
| ICMT | 0.000192 | -0.26454 | 0.151 | 0.275 | 1 |
| AKIRIN1 | 0.000195 | 0.272768 | 0.745 | 0.662 | 1 |
| RORA | 0.000201 | 0.375367 | 0.75 | 0.682 | 1 |
| TTN | 0.000278 | -0.3199 | 0.63 | 0.752 | 1 |
| R3HDM1 | 0.00028 | -0.27761 | 0.328 | 0.445 | 1 |
| MX1 | 0.000362 | -0.27229 | 0.448 | 0.58 | 1 |
| TMPO | 0.000374 | -0.26094 | 0.464 | 0.6 | 1 |
| CD69 | 0.000451 | 0.366771 | 0.974 | 0.981 | 1 |
| UBASH3A | 0.000453 | -0.25954 | 0.401 | 0.53 | 1 |
| CD55 | 0.000483 | 0.29932 | 0.651 | 0.566 | 1 |
| APOBEC3G | 0.000532 | -0.30435 | 0.948 | 0.948 | 1 |
| FAM111A | 0.000534 | -0.27297 | 0.344 | 0.465 | 1 |
| PDE4D | 0.000662 | 0.348411 | 0.729 | 0.693 | 1 |
| WSB1 | 0.000695 | 0.256541 | 0.906 | 0.887 | 1 |
| APOBEC3C | 0.000844 | -0.25218 | 0.938 | 0.935 | 1 |
| BIRC3 | 0.000941 | 0.289618 | 0.891 | 0.883 | 1 |
| SRSF6 | 0.001114 | 0.32524 | 0.865 | 0.87 | 1 |
| MCM3 | 0.001148 | -0.35241 | 0.344 | 0.455 | 1 |
| MAFF | 0.00128 | 0.251966 | 0.417 | 0.32 | 1 |
| GRPEL1 | 0.001396 | 0.259915 | 0.505 | 0.413 | 1 |
| SDCBP | 0.001848 | 0.26463 | 0.859 | 0.869 | 1 |
| TIPARP | 0.001885 | 0.361895 | 0.516 | 0.428 | 1 |
| DENR | 0.002251 | -0.25764 | 0.49 | 0.594 | 1 |
| PRMT9 | 0.002614 | 0.293187 | 0.422 | 0.327 | 1 |
| NFKBIA | 0.002617 | 0.309403 | 0.932 | 0.937 | 1 |
| IFI44L | 0.002885 | -0.38273 | 0.245 | 0.341 | 1 |
| DPYD | 0.002894 | -0.25899 | 0.417 | 0.528 | 1 |
| MAP3K8 | 0.002966 | 0.305366 | 0.542 | 0.459 | 1 |
| SKIL | 0.003111 | 0.303795 | 0.786 | 0.781 | 1 |
| DUSP10 | 0.003123 | 0.381467 | 0.729 | 0.674 | 1 |
| KRT86 | 0.00316 | -0.2755 | 0.161 | 0.261 | 1 |
| HLA-DRA | 0.003351 | -0.29412 | 0.875 | 0.913 | 1 |
| GPR183 | 0.003462 | 0.465982 | 0.766 | 0.757 | 1 |
| PCNA | 0.005012 | -0.27679 | 0.354 | 0.466 | 1 |
| DDX3Y | 0.005305 | 0.396806 | 0.26 | 0.191 | 1 |
| EIF4A3 | 0.005827 | 0.268712 | 0.74 | 0.715 | 1 |
| IL7R | 0.006346 | 0.43431 | 0.76 | 0.809 | 1 |
| OAT | 0.006959 | 0.319238 | 0.583 | 0.546 | 1 |
| ETF1 | 0.007774 | 0.287586 | 0.641 | 0.598 | 1 |
| PON2 | 0.009474 | -0.25057 | 0.219 | 0.305 | 1 |
| SLC7A5 | 0.009479 | 0.276773 | 0.651 | 0.627 | 1 |
| PLK3 | 0.013826 | 0.304572 | 0.495 | 0.446 | 1 |
| ADGRG1 | 0.015739 | -0.26159 | 0.37 | 0.459 | 1 |
| PRR5L | 0.016424 | -0.26374 | 0.208 | 0.292 | 1 |
| MSL2 | 0.017731 | 0.342743 | 0.375 | 0.319 | 1 |
| RLF | 0.018174 | 0.291341 | 0.562 | 0.54 | 1 |
| CDK17 | 0.01841 | 0.262909 | 0.568 | 0.528 | 1 |
| HECA | 0.032296 | 0.258645 | 0.464 | 0.422 | 1 |
| DDX6 | 0.033839 | 0.302542 | 0.776 | 0.795 | 1 |
| HSPA1B | 0.035079 | 0.352346 | 0.698 | 0.682 | 1 |
| G3BP2 | 0.036158 | 0.279545 | 0.885 | 0.902 | 1 |
| TANK | 0.036167 | 0.260651 | 0.87 | 0.902 | 1 |
| LPCAT1 | 0.066892 | 0.292031 | 0.307 | 0.272 | 1 |
| TMEM123 | 0.07202 | 0.259198 | 0.698 | 0.711 | 1 |
| ADIPOR1 | 0.076413 | 0.266929 | 0.62 | 0.632 | 1 |
| PMAIP1 | 0.099023 | 0.342176 | 0.583 | 0.553 | 1 |
| UHRF2 | 0.101425 | 0.27134 | 0.49 | 0.475 | 1 |
| PIM3 | 0.13455 | 0.257294 | 0.438 | 0.415 | 1 |
| JMJD1C | 0.137334 | 0.253424 | 0.781 | 0.852 | 1 |
| ATP2B1 | 0.164808 | 0.351252 | 0.552 | 0.607 | 1 |
| PLIN2 | 0.175972 | 0.383562 | 0.641 | 0.642 | 1 |
| ZFAND2A | 0.192987 | 0.308856 | 0.375 | 0.349 | 1 |
| TUBA1B | 0.194382 | -0.26656 | 0.943 | 0.92 | 1 |
| FCGR3A | 0.275361 | 0.287317 | 0.224 | 0.28 | 1 |
| CCL4L1 | 0.276848 | 0.258894 | 0.818 | 0.908 | 1 |
| SLU7 | 0.311907 | 0.262674 | 0.609 | 0.634 | 1 |
| KLHL24 | 0.320739 | 0.279625 | 0.432 | 0.432 | 1 |
| GEM | 0.465583 | 0.344345 | 0.255 | 0.235 | 1 |
| ANKRD28 | 0.52903 | 0.305049 | 0.474 | 0.508 | 1 |
| SGTB | 0.59663 | 0.254263 | 0.245 | 0.276 | 1 |
| KAT6A | 0.668606 | 0.260502 | 0.547 | 0.574 | 1 |
| EP300 | 0.692646 | 0.359334 | 0.531 | 0.55 | 1 |
| EGR2 | 0.803517 | 0.292205 | 0.302 | 0.308 | 1 |
| C1orf131 | 0.83985 | 0.260936 | 0.26 | 0.29 | 1 |
| IFNG | 0.860121 | 0.536829 | 0.771 | 0.789 | 1 |
| PELI1 | 0.898073 | 0.272661 | 0.453 | 0.488 | 1 |
| **Cluster3** | | | | | |
| GeneSymbol | p_val | logFC | pct.1 | pct.2 | p_val_adj |
| ARAF | 1.39E-06 | -0.26374 | 0.301 | 0.497 | 0.025107 |
| SLC43A3 | 1.44E-05 | -0.31094 | 0.177 | 0.337 | 0.259804 |
| TMEM173 | 2.02E-05 | -0.25971 | 0.409 | 0.592 | 0.363329 |
| MCM7 | 0.000103 | -0.47762 | 0.349 | 0.503 | 1 |
| CXCL13 | 0.000147 | -0.52744 | 0.548 | 0.686 | 1 |
| IPO7 | 0.000253 | -0.27675 | 0.14 | 0.253 | 1 |
| TKT | 0.000269 | -0.29275 | 0.269 | 0.399 | 1 |
| ACAD8 | 0.000337 | -0.26568 | 0.237 | 0.362 | 1 |
| ENOSF1 | 0.000471 | -0.31511 | 0.253 | 0.382 | 1 |
| ZNF331 | 0.000618 | 0.283123 | 0.78 | 0.705 | 1 |
| ILF2 | 0.000672 | -0.25681 | 0.694 | 0.793 | 1 |
| DDX1 | 0.000803 | -0.28653 | 0.333 | 0.449 | 1 |
| CCR1 | 0.001032 | -0.34524 | 0.242 | 0.356 | 1 |
| FCRL3 | 0.001364 | -0.25815 | 0.194 | 0.314 | 1 |
| YARS | 0.001971 | -0.25305 | 0.672 | 0.752 | 1 |
| REL | 0.002032 | 0.288742 | 0.79 | 0.718 | 1 |
| AFAP1L2 | 0.003036 | -0.2991 | 0.237 | 0.34 | 1 |
| MCM4 | 0.003147 | -0.43757 | 0.172 | 0.26 | 1 |
| CTDSPL2 | 0.00324 | -0.25485 | 0.242 | 0.349 | 1 |
| OAS2 | 0.005123 | -0.32724 | 0.462 | 0.54 | 1 |
| SMAD4 | 0.005771 | -0.27282 | 0.204 | 0.292 | 1 |
| STMN1 | 0.005897 | -0.78413 | 0.435 | 0.519 | 1 |
| MCM3 | 0.005941 | -0.28222 | 0.36 | 0.453 | 1 |
| TMEM106C | 0.006484 | -0.28707 | 0.306 | 0.41 | 1 |
| SIPA1L1 | 0.008027 | -0.25278 | 0.269 | 0.354 | 1 |
| GTF3A | 0.00931 | 0.252947 | 0.855 | 0.824 | 1 |
| HLA-DQA1 | 0.012184 | -0.26999 | 0.618 | 0.675 | 1 |
| MED29 | 0.01857 | -0.25531 | 0.387 | 0.467 | 1 |
| STX11 | 0.021242 | 0.308441 | 0.409 | 0.342 | 1 |
| PCNA | 0.024403 | -0.36035 | 0.392 | 0.46 | 1 |
| CRYBG1 | 0.025202 | -0.30455 | 0.575 | 0.644 | 1 |
| GSKIP | 0.037391 | -0.25286 | 0.274 | 0.345 | 1 |
| CCL4 | 0.040614 | 0.321519 | 0.968 | 0.965 | 1 |
| TUBB | 0.041604 | -0.50536 | 0.753 | 0.807 | 1 |
| CCNA2 | 0.047251 | -0.25816 | 0.237 | 0.292 | 1 |
| GNLY | 0.057296 | -0.31084 | 0.683 | 0.764 | 1 |
| SLAMF7 | 0.057555 | 0.267216 | 0.812 | 0.786 | 1 |
| CCL4L1 | 0.058644 | 0.373132 | 0.909 | 0.896 | 1 |
| ABHD14B | 0.069128 | 0.273179 | 0.5 | 0.468 | 1 |
| TUBA1B | 0.079817 | -0.47815 | 0.909 | 0.924 | 1 |
| RGCC | 0.085309 | 0.264087 | 0.72 | 0.726 | 1 |
| SESN1 | 0.106945 | 0.305126 | 0.312 | 0.282 | 1 |
| PLXDC1 | 0.122069 | 0.257147 | 0.323 | 0.285 | 1 |
| PTGER2 | 0.265291 | 0.29082 | 0.328 | 0.308 | 1 |
| MCM5 | 0.273497 | -0.3339 | 0.441 | 0.455 | 1 |
| ZNF800 | 0.296917 | 0.253849 | 0.57 | 0.595 | 1 |
| RNF216 | 0.439924 | 0.263971 | 0.602 | 0.633 | 1 |
| LMNA | 0.487748 | -0.53244 | 0.452 | 0.451 | 1 |
| SCAMP1 | 0.567857 | 0.267082 | 0.312 | 0.312 | 1 |
| CCR7 | 0.723481 | 0.393975 | 0.548 | 0.582 | 1 |
| TNF | 0.773518 | 0.437588 | 0.36 | 0.368 | 1 |
| ADA2 | 0.993301 | 0.262169 | 0.473 | 0.536 | 1 |
| **Cluster4** | | | | | |
| GeneSymbol | p_val | logFC | pct.1 | pct.2 | p_val_adj |
| TBC1D15 | 3.03E-06 | -0.28687 | 0.377 | 0.545 | 0.054698 |
| CLEC2B | 1.06E-05 | 0.294801 | 0.927 | 0.865 | 0.191398 |
| IDI1 | 3.98E-05 | -0.42698 | 0.743 | 0.825 | 0.717267 |
| APLP2 | 0.000172 | -0.28826 | 0.325 | 0.469 | 1 |
| PPIL2 | 0.000274 | -0.32542 | 0.246 | 0.368 | 1 |
| IRF4 | 0.00029 | -0.4874 | 0.429 | 0.567 | 1 |
| TTN | 0.000302 | -0.42083 | 0.66 | 0.748 | 1 |
| LTB | 0.000366 | 0.377682 | 0.639 | 0.543 | 1 |
| PON2 | 0.000405 | -0.2823 | 0.183 | 0.31 | 1 |
| TUBA1B | 0.00054 | -0.4226 | 0.895 | 0.926 | 1 |
| ADA | 0.00058 | 0.318048 | 0.602 | 0.508 | 1 |
| GIMAP7 | 0.0006 | 0.259769 | 0.859 | 0.811 | 1 |
| TUBA1C | 0.000709 | -0.25584 | 0.482 | 0.586 | 1 |
| FCRL6 | 0.000774 | 0.410239 | 0.576 | 0.5 | 1 |
| ANKRD28 | 0.00136 | -0.29665 | 0.382 | 0.52 | 1 |
| ZNF644 | 0.001679 | -0.28562 | 0.602 | 0.696 | 1 |
| RNF19A | 0.001692 | -0.25824 | 0.864 | 0.917 | 1 |
| ENTPD1 | 0.001946 | -0.48401 | 0.524 | 0.609 | 1 |
| HMGB2 | 0.002301 | -0.31219 | 0.859 | 0.929 | 1 |
| TMEM263 | 0.002429 | -0.27269 | 0.272 | 0.384 | 1 |
| EIF4A3 | 0.003155 | -0.33673 | 0.681 | 0.722 | 1 |
| TCP1 | 0.003431 | -0.29855 | 0.78 | 0.847 | 1 |
| ARID5A | 0.00373 | -0.3156 | 0.702 | 0.77 | 1 |
| PCNA | 0.003947 | -0.29053 | 0.372 | 0.463 | 1 |
| HSP90AB1 | 0.004205 | -0.2787 | 1 | 0.992 | 1 |
| MVP | 0.00452 | 0.254671 | 0.743 | 0.694 | 1 |
| TUBB4B | 0.004641 | -0.36638 | 0.586 | 0.694 | 1 |
| KPNA2 | 0.004993 | -0.29289 | 0.529 | 0.637 | 1 |
| TSPYL2 | 0.005388 | -0.38886 | 0.513 | 0.61 | 1 |
| FUS | 0.006126 | -0.26703 | 0.895 | 0.907 | 1 |
| TAOK3 | 0.006279 | 0.269473 | 0.67 | 0.627 | 1 |
| CACYBP | 0.006509 | -0.26942 | 0.675 | 0.746 | 1 |
| MCM4 | 0.007137 | -0.34019 | 0.178 | 0.259 | 1 |
| HSPH1 | 0.007871 | -0.45537 | 0.728 | 0.797 | 1 |
| SYAP1 | 0.008072 | -0.26131 | 0.518 | 0.617 | 1 |
| FKBP5 | 0.008852 | -0.28157 | 0.639 | 0.704 | 1 |
| SERTAD1 | 0.008992 | -0.28234 | 0.445 | 0.531 | 1 |
| SAMHD1 | 0.010249 | 0.251361 | 0.649 | 0.579 | 1 |
| RBMX | 0.01054 | -0.25998 | 0.827 | 0.831 | 1 |
| GLA | 0.010625 | -0.33455 | 0.272 | 0.364 | 1 |
| LMNA | 0.010914 | -0.39615 | 0.377 | 0.46 | 1 |
| ADSL | 0.016919 | 0.250096 | 0.435 | 0.372 | 1 |
| CRYBG1 | 0.01712 | -0.38828 | 0.581 | 0.643 | 1 |
| OSTC | 0.017263 | 0.254225 | 0.592 | 0.55 | 1 |
| FCGR3A | 0.02109 | 0.303922 | 0.34 | 0.265 | 1 |
| IL6ST | 0.02525 | -0.26063 | 0.597 | 0.649 | 1 |
| MCM7 | 0.02678 | -0.5506 | 0.435 | 0.492 | 1 |
| PDHB | 0.028046 | -0.26338 | 0.492 | 0.542 | 1 |
| NEU1 | 0.029384 | -0.32064 | 0.455 | 0.52 | 1 |
| RBM12 | 0.0332 | -0.31244 | 0.785 | 0.803 | 1 |
| INPP4A | 0.033857 | 0.258621 | 0.44 | 0.396 | 1 |
| STMN1 | 0.037641 | -0.67157 | 0.487 | 0.513 | 1 |
| TOX | 0.03993 | -0.30059 | 0.639 | 0.66 | 1 |
| CENPF | 0.042263 | -0.30607 | 0.204 | 0.265 | 1 |
| CRTAM | 0.043338 | -0.35814 | 0.717 | 0.751 | 1 |
| CREM | 0.043342 | -0.40631 | 0.806 | 0.846 | 1 |
| ENOSF1 | 0.045539 | -0.30648 | 0.314 | 0.375 | 1 |
| MCM6 | 0.049728 | -0.31741 | 0.445 | 0.511 | 1 |
| CD300A | 0.05417 | 0.27508 | 0.267 | 0.219 | 1 |
| ZNF292 | 0.055336 | -0.26117 | 0.67 | 0.698 | 1 |
| GPR183 | 0.076275 | -0.42243 | 0.717 | 0.763 | 1 |
| ZFP36 | 0.083393 | -0.33208 | 0.974 | 0.973 | 1 |
| CD200R1 | 0.092612 | -0.25056 | 0.283 | 0.331 | 1 |
| CSNK1G1 | 0.097059 | -0.28384 | 0.524 | 0.553 | 1 |
| FTH1 | 0.097146 | -0.25097 | 1 | 0.999 | 1 |
| DNAJB1 | 0.1094 | -0.30986 | 0.911 | 0.927 | 1 |
| GBP3 | 0.150595 | 0.266496 | 0.518 | 0.497 | 1 |
| HADHA | 0.1666 | 0.258309 | 0.66 | 0.658 | 1 |
| TUBB | 0.184724 | -0.39535 | 0.801 | 0.801 | 1 |
| TSC22D3 | 0.197834 | -0.26629 | 0.995 | 0.971 | 1 |
| DENND2D | 0.19848 | 0.269985 | 0.738 | 0.735 | 1 |
| HSPA1L | 0.215098 | -0.27196 | 0.565 | 0.607 | 1 |
| CD83 | 0.230804 | -0.30477 | 0.346 | 0.375 | 1 |
| CXCL13 | 0.236927 | -0.46697 | 0.665 | 0.671 | 1 |
| BTG2 | 0.286867 | -0.29735 | 0.785 | 0.786 | 1 |
| KIFAP3 | 0.304566 | 0.296668 | 0.33 | 0.333 | 1 |
| SLBP | 0.306523 | -0.27104 | 0.628 | 0.634 | 1 |
| HSP90AA1 | 0.314884 | -0.26429 | 1 | 0.996 | 1 |
| COPG1 | 0.410941 | 0.271416 | 0.44 | 0.461 | 1 |
| NR4A1 | 0.422207 | -0.27759 | 0.45 | 0.473 | 1 |
| CD55 | 0.435136 | -0.25155 | 0.545 | 0.58 | 1 |
| IFI44L | 0.436392 | -0.30061 | 0.298 | 0.334 | 1 |
| KLRC1 | 0.507568 | 0.334534 | 0.513 | 0.493 | 1 |
| EGR2 | 0.525397 | -0.33812 | 0.298 | 0.309 | 1 |
| ZNF791 | 0.623053 | -0.2921 | 0.424 | 0.417 | 1 |
| TNFRSF9 | 0.643822 | -0.29693 | 0.66 | 0.627 | 1 |
| HSPB1 | 0.788768 | -0.2547 | 0.607 | 0.577 | 1 |
| **Cluster5** | | | | | |
| GeneSymbol | p_val | logFC | pct.1 | pct.2 | p_val_adj |
| NR4A2 | 2.29E-17 | -0.82029 | 0.637 | 0.821 | 4.12E-13 |
| ZFP36 | 4.14E-17 | -0.9247 | 0.951 | 0.977 | 7.47E-13 |
| CREM | 5.24E-17 | -0.80561 | 0.7 | 0.864 | 9.45E-13 |
| DNAJB1 | 1.17E-16 | -0.95732 | 0.865 | 0.935 | 2.12E-12 |
| DNAJA1 | 1.12E-15 | -0.73336 | 0.803 | 0.934 | 2.02E-11 |
| HSPA8 | 1.98E-13 | -0.63361 | 1 | 0.999 | 3.56E-09 |
| TNFAIP3 | 8.40E-13 | -0.62692 | 0.955 | 0.982 | 1.51E-08 |
| PER1 | 9.19E-13 | -0.35905 | 0.305 | 0.557 | 1.66E-08 |
| CXCR4 | 2.52E-12 | -0.61114 | 0.978 | 0.994 | 4.54E-08 |
| NR4A1 | 2.77E-12 | -0.89971 | 0.265 | 0.502 | 5.00E-08 |
| STAT1 | 1.20E-11 | 0.448003 | 0.964 | 0.807 | 2.17E-07 |
| HSPH1 | 2.08E-11 | -0.89467 | 0.628 | 0.814 | 3.75E-07 |
| RGS2 | 2.11E-11 | -0.68419 | 0.735 | 0.866 | 3.80E-07 |
| LMNA | 2.82E-11 | -0.96108 | 0.26 | 0.481 | 5.08E-07 |
| CDKN1A | 4.90E-11 | -0.51956 | 0.22 | 0.446 | 8.83E-07 |
| SENP3-EIF4A1 | 8.39E-11 | -0.37649 | 0.987 | 0.992 | 1.51E-06 |
| CD27 | 9.53E-11 | 0.389623 | 0.937 | 0.793 | 1.72E-06 |
| NR4A3 | 1.21E-10 | -0.43229 | 0.197 | 0.41 | 2.19E-06 |
| ZNF331 | 1.46E-10 | -0.52479 | 0.529 | 0.742 | 2.63E-06 |
| DUSP1 | 1.63E-10 | -0.66851 | 0.901 | 0.955 | 2.94E-06 |
| IFITM1 | 1.68E-10 | 0.344525 | 0.996 | 0.97 | 3.03E-06 |
| CD27-AS1 | 2.07E-10 | 0.344441 | 0.915 | 0.739 | 3.73E-06 |
| MYADM | 2.69E-10 | -0.57446 | 0.525 | 0.713 | 4.85E-06 |
| SERTAD1 | 2.76E-10 | -0.54138 | 0.336 | 0.55 | 4.98E-06 |
| GIMAP6 | 3.67E-10 | 0.325384 | 0.704 | 0.478 | 6.62E-06 |
| SAMD9L | 3.67E-10 | 0.334373 | 0.834 | 0.64 | 6.62E-06 |
| SRSF2 | 3.92E-10 | -0.45331 | 0.964 | 0.975 | 7.07E-06 |
| YPEL5 | 4.41E-10 | -0.62714 | 0.883 | 0.918 | 7.95E-06 |
| TUBB4B | 4.88E-10 | -0.64198 | 0.534 | 0.705 | 8.79E-06 |
| CSRNP1 | 9.02E-10 | -0.43242 | 0.336 | 0.54 | 1.63E-05 |
| CKS2 | 1.13E-09 | -0.33594 | 0.323 | 0.522 | 2.03E-05 |
| RGS1 | 1.67E-09 | -0.47933 | 0.987 | 0.987 | 3.02E-05 |
| EIF1 | 1.72E-09 | -0.38828 | 1 | 0.999 | 3.11E-05 |
| SDCBP | 5.91E-09 | -0.54645 | 0.821 | 0.876 | 0.000107 |
| FAM53C | 7.50E-09 | -0.53021 | 0.26 | 0.441 | 0.000135 |
| LY6E | 9.86E-09 | 0.307203 | 0.978 | 0.881 | 0.000178 |
| NFKBIA | 1.05E-08 | -0.60924 | 0.919 | 0.94 | 0.00019 |
| SLFN5 | 1.39E-08 | 0.297347 | 0.812 | 0.652 | 0.00025 |
| SRGN | 1.46E-08 | -0.27879 | 1 | 0.998 | 0.000263 |
| HSPD1 | 1.49E-08 | -0.78596 | 0.803 | 0.844 | 0.000268 |
| GIMAP1 | 2.15E-08 | 0.312799 | 0.578 | 0.395 | 0.000387 |
| HSP90AB1 | 2.37E-08 | -0.55275 | 0.987 | 0.994 | 0.000427 |
| CD83 | 2.38E-08 | -0.7746 | 0.206 | 0.397 | 0.00043 |
| CXCR6 | 3.05E-08 | 0.351669 | 0.946 | 0.838 | 0.00055 |
| SRSF7 | 4.54E-08 | -0.37373 | 0.991 | 0.995 | 0.000818 |
| GPR34 | 5.64E-08 | 0.261505 | 0.256 | 0.121 | 0.001017 |
| LOC284454 | 5.91E-08 | -0.40509 | 0.287 | 0.463 | 0.001065 |
| BTG2 | 6.38E-08 | -0.6793 | 0.709 | 0.798 | 0.001151 |
| OAS2 | 6.77E-08 | 0.397334 | 0.7 | 0.505 | 0.00122 |
| PLEKHF1 | 8.93E-08 | 0.269427 | 0.498 | 0.329 | 0.001609 |
| XAF1 | 9.61E-08 | 0.443891 | 0.771 | 0.613 | 0.001733 |
| IDI1 | 9.63E-08 | -0.43446 | 0.722 | 0.831 | 0.001736 |
| ZC3H12A | 1.03E-07 | -0.45216 | 0.372 | 0.532 | 0.001866 |
| ADORA2A | 1.17E-07 | 0.301679 | 0.596 | 0.421 | 0.002106 |
| UBB | 1.86E-07 | -0.28762 | 1 | 1 | 0.003348 |
| PPP1R15A | 1.92E-07 | -0.522 | 0.803 | 0.858 | 0.003463 |
| JUNB | 2.15E-07 | -0.5914 | 0.933 | 0.947 | 0.00388 |
| LCP2 | 2.35E-07 | 0.306857 | 0.978 | 0.9 | 0.004242 |
| PTGER4 | 2.46E-07 | -0.45667 | 0.655 | 0.756 | 0.004429 |
| REL | 2.57E-07 | -0.41919 | 0.605 | 0.745 | 0.004628 |
| IFI44L | 2.71E-07 | 0.634066 | 0.466 | 0.309 | 0.004888 |
| EIF4A3 | 2.86E-07 | -0.48619 | 0.628 | 0.732 | 0.005158 |
| ZEB2 | 2.95E-07 | -0.46589 | 0.659 | 0.765 | 0.005325 |
| APOBEC3C | 3.22E-07 | 0.256535 | 0.987 | 0.928 | 0.005805 |
| FAM46C | 3.85E-07 | -0.43585 | 0.547 | 0.705 | 0.006936 |
| DTX3L | 4.25E-07 | 0.362222 | 0.623 | 0.458 | 0.00767 |
| SLC7A5 | 4.34E-07 | -0.41547 | 0.538 | 0.644 | 0.007821 |
| HSPA1B | 4.80E-07 | -0.76334 | 0.574 | 0.701 | 0.008659 |
| SPECC1L | 5.54E-07 | 0.266603 | 0.749 | 0.594 | 0.009996 |
| DDX3X | 5.55E-07 | -0.43018 | 0.964 | 0.956 | 0.010009 |
| GTF2B | 6.10E-07 | -0.39761 | 0.556 | 0.69 | 0.011002 |
| SLC16A1 | 6.26E-07 | -0.40494 | 0.242 | 0.394 | 0.011288 |
| OAS1 | 7.72E-07 | 0.332195 | 0.529 | 0.356 | 0.013913 |
| TXNIP | 1.00E-06 | 0.265694 | 0.982 | 0.939 | 0.018037 |
| IRF4 | 1.04E-06 | -0.51325 | 0.426 | 0.571 | 0.01883 |
| RSAD2 | 1.07E-06 | 0.306681 | 0.395 | 0.242 | 0.019237 |
| VRK3 | 1.18E-06 | 0.283477 | 0.583 | 0.428 | 0.021227 |
| TSC22D3 | 1.19E-06 | -0.57534 | 0.982 | 0.973 | 0.021444 |
| PDE4DIP | 1.20E-06 | 0.310915 | 0.821 | 0.644 | 0.021672 |
| C14orf119 | 1.35E-06 | 0.264502 | 0.628 | 0.461 | 0.024374 |
| HSPE1 | 1.42E-06 | -0.41133 | 0.668 | 0.771 | 0.025517 |
| DUSP10 | 1.58E-06 | -0.39255 | 0.561 | 0.699 | 0.028526 |
| LDHA | 1.73E-06 | -0.32686 | 1 | 0.994 | 0.031191 |
| SAMD9 | 1.78E-06 | 0.324168 | 0.807 | 0.632 | 0.032072 |
| HSP90AA1 | 1.89E-06 | -0.66587 | 0.996 | 0.996 | 0.034101 |
| IFIT1 | 1.91E-06 | 0.325568 | 0.251 | 0.131 | 0.034388 |
| SRSF3 | 2.13E-06 | -0.31916 | 0.951 | 0.964 | 0.038365 |
| TOX | 2.14E-06 | 0.250573 | 0.789 | 0.637 | 0.038572 |
| HAVCR2 | 2.26E-06 | 0.365323 | 0.843 | 0.709 | 0.04082 |
| ATF4 | 2.29E-06 | -0.35827 | 0.668 | 0.778 | 0.041347 |
| IFI44 | 2.33E-06 | 0.38755 | 0.623 | 0.46 | 0.042045 |
| SNHG12 | 2.34E-06 | -0.36192 | 0.484 | 0.613 | 0.042216 |
| SLC38A2 | 2.38E-06 | -0.35908 | 0.785 | 0.876 | 0.042863 |
| FOSL2 | 2.49E-06 | -0.39321 | 0.341 | 0.493 | 0.044864 |
| MX1 | 2.69E-06 | 0.292599 | 0.686 | 0.545 | 0.04842 |
| ARHGAP30 | 2.85E-06 | 0.287767 | 0.839 | 0.728 | 0.051466 |
| GIMAP4 | 2.96E-06 | 0.294469 | 0.946 | 0.812 | 0.053413 |
| NFX1 | 3.62E-06 | 0.334685 | 0.587 | 0.443 | 0.065181 |
| BRD2 | 4.11E-06 | -0.3778 | 0.857 | 0.904 | 0.074172 |
| CD55 | 4.64E-06 | -0.51226 | 0.48 | 0.591 | 0.083725 |
| FBXO6 | 5.08E-06 | 0.263956 | 0.453 | 0.307 | 0.091643 |
| EZR | 6.33E-06 | -0.36355 | 0.982 | 0.988 | 0.114206 |
| ENDOD1 | 6.73E-06 | 0.250362 | 0.336 | 0.206 | 0.121409 |
| PFKFB3 | 7.23E-06 | -0.48812 | 0.502 | 0.623 | 0.130399 |
| DUSP2 | 7.64E-06 | -0.53087 | 0.919 | 0.926 | 0.137813 |
| IL2RB | 7.88E-06 | 0.269126 | 0.987 | 0.906 | 0.142043 |
| GIMAP1-GIMAP5 | 8.16E-06 | 0.266524 | 0.861 | 0.777 | 0.147145 |
| SLAMF6 | 8.30E-06 | 0.279707 | 0.753 | 0.613 | 0.149663 |
| ITM2A | 8.39E-06 | 0.251121 | 0.987 | 0.913 | 0.151245 |
| HSPA1L | 8.62E-06 | -0.79804 | 0.498 | 0.618 | 0.155422 |
| ATF3 | 9.27E-06 | -0.39403 | 0.152 | 0.298 | 0.167091 |
| CASP1 | 9.51E-06 | 0.26722 | 0.951 | 0.805 | 0.171514 |
| RNF213 | 9.56E-06 | 0.281746 | 0.996 | 0.956 | 0.172283 |
| MAFF | 9.84E-06 | -0.44258 | 0.22 | 0.349 | 0.177344 |
| ANXA1 | 1.01E-05 | -0.65692 | 0.964 | 0.97 | 0.182786 |
| RGCC | 1.10E-05 | -0.43226 | 0.641 | 0.739 | 0.19773 |
| TUBA4A | 1.24E-05 | -0.4729 | 0.91 | 0.909 | 0.223045 |
| NASP | 1.32E-05 | -0.39182 | 0.731 | 0.787 | 0.237448 |
| IFRD1 | 1.44E-05 | -0.39898 | 0.578 | 0.69 | 0.25882 |
| TUBA1A | 1.46E-05 | -0.34318 | 0.717 | 0.826 | 0.262525 |
| NXT1 | 1.68E-05 | -0.26357 | 0.247 | 0.382 | 0.303298 |
| DENND2C | 1.74E-05 | -0.34132 | 0.605 | 0.693 | 0.3128 |
| CLK1 | 1.90E-05 | -0.37274 | 0.933 | 0.951 | 0.343394 |
| MTO1 | 1.95E-05 | 0.298874 | 0.502 | 0.356 | 0.352061 |
| FCRL3 | 2.05E-05 | 0.286342 | 0.417 | 0.282 | 0.370069 |
| TOB1 | 2.08E-05 | -0.55218 | 0.744 | 0.852 | 0.374758 |
| PEX1 | 2.34E-05 | 0.263311 | 0.345 | 0.223 | 0.421678 |
| AMD1 | 2.38E-05 | -0.35032 | 0.543 | 0.676 | 0.4282 |
| BCAS2 | 2.38E-05 | -0.39112 | 0.601 | 0.679 | 0.429525 |
| METTL3 | 2.63E-05 | 0.254066 | 0.489 | 0.344 | 0.473909 |
| TRIM22 | 2.88E-05 | 0.278923 | 0.946 | 0.874 | 0.518344 |
| GRPEL1 | 2.88E-05 | -0.31054 | 0.305 | 0.443 | 0.518878 |
| NFKBIZ | 2.96E-05 | -0.45648 | 0.529 | 0.659 | 0.53337 |
| CD82 | 3.20E-05 | 0.275924 | 0.91 | 0.837 | 0.577032 |
| ACP5 | 3.35E-05 | 0.307996 | 0.722 | 0.591 | 0.603537 |
| DUSP4 | 3.95E-05 | -0.26443 | 0.744 | 0.828 | 0.711678 |
| WDR47 | 4.26E-05 | -0.32506 | 0.148 | 0.267 | 0.767371 |
| ETF1 | 4.28E-05 | -0.2837 | 0.489 | 0.621 | 0.772297 |
| DENND2D | 4.31E-05 | 0.257682 | 0.834 | 0.72 | 0.777009 |
| GLA | 4.50E-05 | -0.53618 | 0.251 | 0.369 | 0.811195 |
| SGK1 | 4.55E-05 | -0.47122 | 0.157 | 0.278 | 0.819835 |
| ZNF766 | 4.58E-05 | 0.28784 | 0.354 | 0.24 | 0.825436 |
| IFI6 | 4.98E-05 | 0.282133 | 0.753 | 0.636 | 0.896912 |
| DENND4A | 5.31E-05 | -0.29366 | 0.484 | 0.597 | 0.956921 |
| MAP3K8 | 5.85E-05 | -0.39746 | 0.359 | 0.486 | 1 |
| BIRC3 | 5.92E-05 | -0.36563 | 0.852 | 0.889 | 1 |
| PIP4K2C | 6.06E-05 | 0.25243 | 0.48 | 0.351 | 1 |
| ARHGEF3 | 6.57E-05 | 0.262289 | 0.888 | 0.804 | 1 |
| FBXO44 | 7.23E-05 | 0.290379 | 0.516 | 0.389 | 1 |
| TUBA1C | 7.49E-05 | -0.26733 | 0.471 | 0.59 | 1 |
| PTP4A1 | 8.38E-05 | -0.30354 | 0.7 | 0.759 | 1 |
| TSPYL2 | 8.58E-05 | -0.53683 | 0.507 | 0.613 | 1 |
| FASLG | 0.000114 | 0.279735 | 0.776 | 0.632 | 1 |
| FAM177A1 | 0.00012 | -0.25544 | 0.529 | 0.614 | 1 |
| LINC-PINT | 0.000121 | -0.35716 | 0.839 | 0.867 | 1 |
| SERPINB9 | 0.000125 | -0.40102 | 0.933 | 0.928 | 1 |
| PIK3R1 | 0.00013 | -0.455 | 0.717 | 0.77 | 1 |
| ZFAND5 | 0.000131 | -0.32922 | 0.664 | 0.739 | 1 |
| UAP1 | 0.000155 | -0.36907 | 0.238 | 0.36 | 1 |
| PMAIP1 | 0.000158 | -0.35037 | 0.462 | 0.571 | 1 |
| HERPUD1 | 0.000187 | -0.37071 | 0.897 | 0.909 | 1 |
| TRMT13 | 0.000215 | 0.294036 | 0.359 | 0.258 | 1 |
| FTH1 | 0.000216 | -0.39689 | 1 | 0.999 | 1 |
| EGR1 | 0.000222 | -0.49265 | 0.377 | 0.495 | 1 |
| DCTN6 | 0.00026 | -0.40589 | 0.498 | 0.578 | 1 |
| JUN | 0.000281 | -0.48467 | 0.848 | 0.887 | 1 |
| KRT86 | 0.000305 | 0.396963 | 0.345 | 0.234 | 1 |
| TUBA1B | 0.000315 | -0.62762 | 0.915 | 0.923 | 1 |
| PLK3 | 0.000321 | -0.34332 | 0.354 | 0.467 | 1 |
| OAT | 0.000328 | -0.37706 | 0.475 | 0.562 | 1 |
| CHD1 | 0.000365 | -0.25803 | 0.74 | 0.803 | 1 |
| CHMP1B | 0.000407 | -0.38656 | 0.578 | 0.647 | 1 |
| LASP1 | 0.000455 | 0.290826 | 0.785 | 0.683 | 1 |
| TIPARP | 0.000458 | -0.32247 | 0.341 | 0.454 | 1 |
| CD69 | 0.000598 | -0.52717 | 0.991 | 0.979 | 1 |
| TRAF5 | 0.000662 | 0.299379 | 0.776 | 0.651 | 1 |
| SLBP | 0.000724 | -0.31246 | 0.57 | 0.644 | 1 |
| RNF125 | 0.00073 | -0.37911 | 0.646 | 0.689 | 1 |
| SARAF | 0.000758 | -0.2715 | 1 | 0.997 | 1 |
| GAS5 | 0.000912 | -0.27764 | 0.758 | 0.781 | 1 |
| KPNA2 | 0.000983 | -0.39277 | 0.583 | 0.631 | 1 |
| ARL2BP | 0.000999 | 0.268229 | 0.632 | 0.517 | 1 |
| FOS | 0.001021 | -0.48468 | 0.816 | 0.848 | 1 |
| DEDD2 | 0.001039 | -0.38433 | 0.525 | 0.609 | 1 |
| TYW1 | 0.001047 | 0.281687 | 0.332 | 0.24 | 1 |
| PCNA | 0.001068 | -0.44875 | 0.381 | 0.464 | 1 |
| SAT1 | 0.001301 | -0.39039 | 0.91 | 0.926 | 1 |
| ZFAND2A | 0.001353 | -0.35639 | 0.269 | 0.365 | 1 |
| JMJD6 | 0.00137 | -0.30237 | 0.578 | 0.682 | 1 |
| MORF4L2 | 0.001386 | -0.30006 | 0.578 | 0.658 | 1 |
| DDIT4 | 0.001551 | -0.33256 | 0.888 | 0.892 | 1 |
| SNHG1 | 0.001571 | -0.30839 | 0.363 | 0.448 | 1 |
| TMEM2 | 0.001751 | -0.40624 | 0.655 | 0.728 | 1 |
| SNHG15 | 0.001798 | -0.32223 | 0.323 | 0.407 | 1 |
| RBL2 | 0.002116 | 0.255514 | 0.924 | 0.852 | 1 |
| GPR183 | 0.002289 | -0.59927 | 0.726 | 0.762 | 1 |
| CD200R1 | 0.002303 | 0.375534 | 0.399 | 0.314 | 1 |
| ELMSAN1 | 0.002362 | -0.28271 | 0.57 | 0.656 | 1 |
| NOP56 | 0.002408 | -0.30614 | 0.677 | 0.711 | 1 |
| ELF1 | 0.002674 | -0.27687 | 0.964 | 0.955 | 1 |
| LANCL1 | 0.002877 | 0.258596 | 0.408 | 0.316 | 1 |
| IER2 | 0.0032 | -0.41661 | 0.843 | 0.863 | 1 |
| GADD45B | 0.003538 | -0.34446 | 0.395 | 0.46 | 1 |
| FOSB | 0.003942 | -0.35175 | 0.704 | 0.721 | 1 |
| LDLR | 0.00409 | -0.29593 | 0.283 | 0.362 | 1 |
| KLF6 | 0.004346 | -0.27976 | 0.991 | 0.985 | 1 |
| ARID5B | 0.00441 | -0.35237 | 0.771 | 0.792 | 1 |
| TCP1 | 0.005761 | -0.3028 | 0.848 | 0.838 | 1 |
| USP47 | 0.005953 | 0.251178 | 0.726 | 0.655 | 1 |
| HSPA4 | 0.006588 | -0.26021 | 0.484 | 0.57 | 1 |
| TFRC | 0.008615 | -0.26143 | 0.722 | 0.749 | 1 |
| EGR2 | 0.009478 | -0.4255 | 0.247 | 0.317 | 1 |
| HIF1A | 0.009927 | -0.27855 | 0.839 | 0.835 | 1 |
| CNOT6L | 0.010964 | -0.26347 | 0.83 | 0.877 | 1 |
| NEU1 | 0.011201 | -0.43491 | 0.457 | 0.521 | 1 |
| PPP1CB | 0.012142 | -0.2596 | 0.946 | 0.944 | 1 |
| DNAJB9 | 0.012297 | -0.26286 | 0.457 | 0.533 | 1 |
| AKAP5 | 0.014103 | 0.26583 | 0.413 | 0.328 | 1 |
| DNAJC9 | 0.014714 | -0.26481 | 0.422 | 0.478 | 1 |
| KANSL2 | 0.015006 | -0.25907 | 0.457 | 0.509 | 1 |
| CHORDC1 | 0.015409 | -0.26654 | 0.565 | 0.65 | 1 |
| SLC1A5 | 0.017203 | -0.25713 | 0.462 | 0.521 | 1 |
| NOP58 | 0.018958 | -0.25293 | 0.892 | 0.896 | 1 |
| RNF168 | 0.019151 | -0.33607 | 0.489 | 0.55 | 1 |
| ZFP36L2 | 0.021143 | -0.29317 | 0.969 | 0.977 | 1 |
| H2AFZ | 0.024653 | -0.27192 | 0.892 | 0.9 | 1 |
| INSIG1 | 0.024891 | -0.28163 | 0.404 | 0.466 | 1 |
| SOCS3 | 0.026195 | -0.26551 | 0.287 | 0.342 | 1 |
| ZBTB1 | 0.026644 | -0.25615 | 0.709 | 0.739 | 1 |
| MCM7 | 0.028314 | -0.72046 | 0.475 | 0.487 | 1 |
| NFE2L2 | 0.031022 | -0.30632 | 0.646 | 0.659 | 1 |
| DNAJA4 | 0.031281 | -0.3223 | 0.206 | 0.263 | 1 |
| SKIL | 0.03173 | -0.2981 | 0.776 | 0.782 | 1 |
| PLIN2 | 0.033832 | -0.33837 | 0.61 | 0.647 | 1 |
| SLC2A3 | 0.035025 | -0.28372 | 0.897 | 0.885 | 1 |
| HMGB2 | 0.036407 | -0.29031 | 0.91 | 0.922 | 1 |
| HSPA6 | 0.037077 | -1.18277 | 0.197 | 0.254 | 1 |
| ATP2B1 | 0.038021 | -0.27421 | 0.574 | 0.605 | 1 |
| CACYBP | 0.040899 | -0.29917 | 0.753 | 0.735 | 1 |
| ICAM1 | 0.041391 | -0.373 | 0.305 | 0.351 | 1 |
| P4HA1 | 0.044731 | -0.25059 | 0.354 | 0.389 | 1 |
| MCM6 | 0.047765 | -0.35785 | 0.493 | 0.505 | 1 |
| GNAS | 0.063125 | -0.28888 | 0.978 | 0.942 | 1 |
| SELL | 0.072787 | 0.358754 | 0.637 | 0.583 | 1 |
| LTB | 0.073217 | 0.293405 | 0.592 | 0.548 | 1 |
| CCL3 | 0.073689 | -0.30374 | 0.776 | 0.67 | 1 |
| MCM4 | 0.075925 | -0.31807 | 0.215 | 0.255 | 1 |
| CRYBG1 | 0.091537 | -0.31513 | 0.619 | 0.639 | 1 |
| ANKRD28 | 0.108332 | -0.27285 | 0.48 | 0.507 | 1 |
| STMN1 | 0.118667 | -0.83604 | 0.516 | 0.509 | 1 |
| DNAJB4 | 0.122307 | -0.31173 | 0.256 | 0.296 | 1 |
| IFNG | 0.127006 | -0.51982 | 0.794 | 0.786 | 1 |
| CCL4 | 0.145253 | -0.44743 | 0.973 | 0.964 | 1 |
| TUBB | 0.15784 | -0.39696 | 0.91 | 0.784 | 1 |
| PNP | 0.165943 | -0.28109 | 0.538 | 0.543 | 1 |
| CRTAM | 0.21495 | -0.36622 | 0.762 | 0.745 | 1 |
| NUSAP1 | 0.375879 | -0.28532 | 0.363 | 0.368 | 1 |
| FKBP5 | 0.377286 | -0.30238 | 0.744 | 0.689 | 1 |
| TNF | 0.409553 | -0.26481 | 0.345 | 0.371 | 1 |
| IL7R | 0.439082 | -0.3269 | 0.848 | 0.797 | 1 |
| CCL4L1 | 0.482144 | -0.38289 | 0.924 | 0.893 | 1 |
| MCM5 | 0.50555 | -0.3498 | 0.475 | 0.45 | 1 |
| GLUL | 0.579296 | -0.31426 | 0.502 | 0.482 | 1 |
| EP300 | 0.651305 | -0.26167 | 0.543 | 0.548 | 1 |
| ENOSF1 | 0.743166 | -0.34408 | 0.386 | 0.365 | 1 |
| HSPB1 | 0.753396 | -0.29965 | 0.601 | 0.578 | 1 |
| **Cluster6** | | | | | |
| GeneSymbol | p_val | logFC | pct.1 | pct.2 | p_val_adj |
| CXCR4 | 8.66E-13 | -0.78415 | 0.975 | 0.993 | 1.56E-08 |
| CD27 | 2.56E-10 | 0.384198 | 0.969 | 0.796 | 4.62E-06 |
| ZFP36 | 1.08E-09 | -0.82516 | 0.969 | 0.974 | 1.94E-05 |
| CD27-AS1 | 1.17E-09 | 0.346359 | 0.944 | 0.743 | 2.10E-05 |
| SIT1 | 1.62E-09 | 0.274927 | 0.925 | 0.695 | 2.92E-05 |
| FUT8 | 1.97E-09 | 0.331714 | 0.712 | 0.497 | 3.56E-05 |
| GDI2 | 4.08E-09 | 0.339996 | 0.912 | 0.754 | 7.36E-05 |
| SMARCAD1 | 4.95E-09 | 0.268095 | 0.681 | 0.437 | 8.92E-05 |
| PRDX3 | 5.14E-09 | 0.347471 | 0.844 | 0.643 | 9.26E-05 |
| PDCD1 | 5.29E-09 | 0.406357 | 0.844 | 0.645 | 9.54E-05 |
| MYO5B | 1.16E-08 | 0.345136 | 0.438 | 0.236 | 0.000209 |
| HAVCR2 | 1.30E-08 | 0.414915 | 0.9 | 0.709 | 0.000234 |
| ITGAE | 2.34E-08 | 0.328789 | 0.994 | 0.896 | 0.000422 |
| ENTPD1 | 2.62E-08 | 0.464682 | 0.794 | 0.578 | 0.000473 |
| CXCL13 | 3.57E-08 | 0.49393 | 0.85 | 0.651 | 0.000644 |
| NUCB1 | 3.67E-08 | 0.284795 | 0.825 | 0.597 | 0.000662 |
| OAS1 | 3.69E-08 | 0.463011 | 0.556 | 0.361 | 0.000666 |
| FKBP1A | 3.74E-08 | 0.320437 | 0.975 | 0.849 | 0.000675 |
| PDCD10 | 3.92E-08 | 0.305086 | 0.681 | 0.466 | 0.000707 |
| MTCH2 | 3.93E-08 | 0.305493 | 0.656 | 0.421 | 0.000709 |
| ENTPD1-AS1 | 4.86E-08 | 0.253563 | 0.631 | 0.402 | 0.000876 |
| CD82 | 6.89E-08 | 0.39026 | 0.95 | 0.836 | 0.001243 |
| APOBEC3C | 7.66E-08 | 0.293927 | 1 | 0.929 | 0.001381 |
| TMEM189-UBE2V1 | 1.28E-07 | 0.265303 | 0.969 | 0.841 | 0.0023 |
| ATP8B4 | 1.89E-07 | 0.265899 | 0.538 | 0.33 | 0.003405 |
| FASLG | 1.92E-07 | 0.308532 | 0.812 | 0.634 | 0.003463 |
| PMPCA | 3.25E-07 | 0.279664 | 0.5 | 0.306 | 0.005867 |
| CCR1 | 3.57E-07 | 0.392682 | 0.512 | 0.325 | 0.00644 |
| PTPN6 | 3.62E-07 | 0.317535 | 0.881 | 0.68 | 0.006533 |
| SIRPG | 4.96E-07 | 0.285765 | 0.888 | 0.725 | 0.008936 |
| ACTG1 | 4.96E-07 | 0.281027 | 1 | 0.999 | 0.008951 |
| IL2RB | 5.01E-07 | 0.325888 | 0.981 | 0.91 | 0.00903 |
| JUNB | 5.66E-07 | -0.6183 | 0.9 | 0.95 | 0.010201 |
| DDX60 | 5.98E-07 | 0.266219 | 0.706 | 0.501 | 0.010784 |
| DPP3 | 7.18E-07 | 0.253445 | 0.506 | 0.322 | 0.01295 |
| DUSP1 | 1.14E-06 | -0.65264 | 0.912 | 0.952 | 0.020485 |
| COPZ1 | 1.38E-06 | 0.27764 | 0.838 | 0.651 | 0.024912 |
| CD200R1 | 1.42E-06 | 0.292331 | 0.494 | 0.308 | 0.025627 |
| TNFAIP3 | 1.52E-06 | -0.50826 | 0.969 | 0.979 | 0.0274 |
| OAS2 | 1.66E-06 | 0.355419 | 0.681 | 0.515 | 0.029981 |
| PKM | 1.76E-06 | 0.309054 | 1 | 0.9 | 0.031748 |
| APOBEC3G | 1.80E-06 | 0.322995 | 0.981 | 0.945 | 0.032391 |
| SYT11 | 2.41E-06 | 0.262215 | 0.694 | 0.505 | 0.043423 |
| IKZF3 | 3.01E-06 | 0.300819 | 0.994 | 0.894 | 0.054325 |
| LMNA | 3.48E-06 | -0.92273 | 0.319 | 0.465 | 0.06282 |
| RPL3 | 3.56E-06 | -0.2918 | 0.994 | 0.999 | 0.064126 |
| GPR183 | 3.73E-06 | -0.62737 | 0.662 | 0.768 | 0.067185 |
| CXCR6 | 4.40E-06 | 0.316714 | 0.975 | 0.839 | 0.079281 |
| HNRNPH1 | 4.60E-06 | -0.26658 | 1 | 0.999 | 0.082898 |
| EIF1 | 4.65E-06 | -0.34881 | 1 | 0.999 | 0.083806 |
| CSF1 | 4.67E-06 | 0.279387 | 0.512 | 0.343 | 0.084199 |
| ANXA5 | 5.38E-06 | 0.261662 | 0.962 | 0.853 | 0.096965 |
| PAPSS1 | 6.62E-06 | 0.282007 | 0.425 | 0.263 | 0.119307 |
| SEC61A1 | 6.75E-06 | 0.25524 | 0.781 | 0.636 | 0.121724 |
| TIGIT | 9.18E-06 | 0.31267 | 0.95 | 0.867 | 0.165524 |
| S100PBP | 1.06E-05 | 0.262024 | 0.575 | 0.392 | 0.190697 |
| MYADM | 1.23E-05 | -0.56858 | 0.594 | 0.697 | 0.22196 |
| TNFSF4 | 1.30E-05 | 0.319608 | 0.588 | 0.392 | 0.234354 |
| VCAM1 | 1.32E-05 | 0.321493 | 0.719 | 0.535 | 0.238253 |
| SARAF | 1.36E-05 | -0.34715 | 1 | 0.997 | 0.245985 |
| PTPN7 | 1.76E-05 | 0.261894 | 0.969 | 0.865 | 0.317457 |
| XIST | 2.03E-05 | 0.287609 | 0.794 | 0.685 | 0.365379 |
| CHST12 | 2.10E-05 | 0.272536 | 0.8 | 0.651 | 0.377906 |
| SRSF2 | 2.25E-05 | -0.36928 | 0.969 | 0.974 | 0.405878 |
| DZIP3 | 2.33E-05 | 0.282289 | 0.619 | 0.452 | 0.419271 |
| DUSP2 | 2.61E-05 | -0.50517 | 0.9 | 0.928 | 0.470867 |
| TNFRSF9 | 2.79E-05 | 0.396724 | 0.75 | 0.618 | 0.50367 |
| MKRN2 | 3.35E-05 | 0.25498 | 0.675 | 0.499 | 0.604051 |
| FOS | 4.25E-05 | -0.82096 | 0.781 | 0.85 | 0.765747 |
| SF3A3 | 4.46E-05 | 0.316572 | 0.625 | 0.469 | 0.804259 |
| SRSF7 | 4.57E-05 | -0.34823 | 1 | 0.994 | 0.823268 |
| TUBB4B | 4.63E-05 | -0.54165 | 0.612 | 0.689 | 0.835473 |
| ZNF331 | 4.89E-05 | -0.45201 | 0.588 | 0.727 | 0.881432 |
| NBR1 | 4.95E-05 | 0.258061 | 0.719 | 0.542 | 0.892597 |
| NR4A3 | 6.33E-05 | -0.42722 | 0.25 | 0.396 | 1 |
| BTG1 | 6.91E-05 | -0.36139 | 1 | 0.994 | 1 |
| AFAP1L2 | 6.96E-05 | 0.375748 | 0.456 | 0.315 | 1 |
| ANXA1 | 7.67E-05 | -0.5948 | 0.969 | 0.969 | 1 |
| TPM4 | 8.18E-05 | 0.314159 | 0.931 | 0.812 | 1 |
| CD101 | 8.37E-05 | 0.253126 | 0.638 | 0.458 | 1 |
| SEMA4A | 0.000102 | 0.341688 | 0.481 | 0.334 | 1 |
| NFKBIZ | 0.00011 | -0.65615 | 0.562 | 0.65 | 1 |
| NDFIP2 | 0.000111 | 0.308128 | 0.731 | 0.6 | 1 |
| LAYN | 0.000113 | 0.294129 | 0.45 | 0.314 | 1 |
| IFI44L | 0.000119 | 0.420819 | 0.456 | 0.316 | 1 |
| HSP90AA1 | 0.000138 | -0.71958 | 1 | 0.996 | 1 |
| SERTAD1 | 0.000147 | -0.48224 | 0.419 | 0.532 | 1 |
| YPEL5 | 0.000147 | -0.50854 | 0.906 | 0.914 | 1 |
| NR4A2 | 0.000172 | -0.33822 | 0.7 | 0.806 | 1 |
| TUG1 | 0.000175 | 0.334987 | 0.462 | 0.318 | 1 |
| EFTUD2 | 0.000185 | 0.309947 | 0.581 | 0.46 | 1 |
| SAMD9 | 0.000189 | 0.257879 | 0.769 | 0.643 | 1 |
| TSC22D3 | 0.00021 | -0.5765 | 0.975 | 0.974 | 1 |
| GADD45B | 0.000238 | -0.38088 | 0.331 | 0.464 | 1 |
| TTN | 0.000241 | 0.333105 | 0.85 | 0.725 | 1 |
| SNHG1 | 0.000243 | -0.38688 | 0.306 | 0.451 | 1 |
| LINC-PINT | 0.000245 | -0.42573 | 0.838 | 0.866 | 1 |
| KRT86 | 0.00027 | 0.361362 | 0.362 | 0.237 | 1 |
| FTH1 | 0.000289 | -0.4417 | 1 | 0.999 | 1 |
| BTG2 | 0.000291 | -0.61614 | 0.719 | 0.793 | 1 |
| TCEAL8 | 0.000305 | 0.270437 | 0.338 | 0.219 | 1 |
| DNAJB1 | 0.000313 | -0.72889 | 0.9 | 0.928 | 1 |
| LRRN3 | 0.000327 | 0.250084 | 0.356 | 0.236 | 1 |
| CCR5 | 0.000338 | 0.260927 | 0.55 | 0.42 | 1 |
| RGCC | 0.000347 | -0.33849 | 0.638 | 0.735 | 1 |
| KIDINS220 | 0.00035 | 0.269417 | 0.556 | 0.415 | 1 |
| TOR1AIP1 | 0.000352 | 0.278037 | 0.775 | 0.655 | 1 |
| ELL2 | 0.000374 | -0.30101 | 0.175 | 0.3 | 1 |
| COG2 | 0.000409 | 0.264344 | 0.362 | 0.244 | 1 |
| CCDC141 | 0.00055 | 0.276278 | 0.625 | 0.499 | 1 |
| ZFAND5 | 0.000581 | -0.36297 | 0.612 | 0.741 | 1 |
| IER2 | 0.000593 | -0.53316 | 0.838 | 0.863 | 1 |
| ZFP36L2 | 0.000606 | -0.35504 | 0.956 | 0.978 | 1 |
| SKIL | 0.000614 | -0.51131 | 0.756 | 0.784 | 1 |
| PNRC1 | 0.000628 | -0.3878 | 0.856 | 0.894 | 1 |
| CREM | 0.000972 | -0.54438 | 0.825 | 0.843 | 1 |
| UQCRC2 | 0.001008 | 0.284556 | 0.806 | 0.721 | 1 |
| FYN | 0.001243 | -0.32221 | 0.956 | 0.958 | 1 |
| CD83 | 0.001323 | -0.39717 | 0.275 | 0.382 | 1 |
| HEXIM1 | 0.001346 | -0.30752 | 0.312 | 0.413 | 1 |
| CSRNP1 | 0.001503 | -0.43317 | 0.431 | 0.521 | 1 |
| UBXN2B | 0.00155 | 0.269668 | 0.344 | 0.24 | 1 |
| ATP2B1 | 0.001569 | -0.40889 | 0.519 | 0.61 | 1 |
| DNAJA1 | 0.001632 | -0.44892 | 0.888 | 0.919 | 1 |
| KLF6 | 0.001744 | -0.38014 | 0.988 | 0.985 | 1 |
| IL7R | 0.001746 | -0.73398 | 0.838 | 0.8 | 1 |
| ECE1 | 0.001783 | 0.266357 | 0.494 | 0.396 | 1 |
| PPP1R15A | 0.001829 | -0.41686 | 0.838 | 0.852 | 1 |
| FAM129A | 0.001842 | -0.31271 | 0.75 | 0.765 | 1 |
| NFKBIA | 0.001965 | -0.34029 | 0.925 | 0.938 | 1 |
| OXA1L | 0.002023 | 0.259608 | 0.556 | 0.459 | 1 |
| ZEB2 | 0.002037 | -0.44807 | 0.731 | 0.753 | 1 |
| JMJD6 | 0.002077 | -0.48538 | 0.631 | 0.672 | 1 |
| STAT4 | 0.002155 | -0.26243 | 0.9 | 0.923 | 1 |
| DBN1 | 0.002183 | 0.308991 | 0.262 | 0.174 | 1 |
| TCTA | 0.002233 | 0.269045 | 0.394 | 0.297 | 1 |
| CALU | 0.002713 | 0.253006 | 0.506 | 0.39 | 1 |
| CRYBG1 | 0.002767 | -0.4006 | 0.575 | 0.643 | 1 |
| FAM46C | 0.002849 | -0.44074 | 0.638 | 0.688 | 1 |
| PDE4B | 0.003184 | -0.29467 | 0.806 | 0.863 | 1 |
| FOSB | 0.003366 | -0.43993 | 0.694 | 0.721 | 1 |
| JUN | 0.003554 | -0.46229 | 0.875 | 0.882 | 1 |
| TUBA1A | 0.003885 | -0.35784 | 0.769 | 0.816 | 1 |
| TAGLN2 | 0.004036 | -0.26976 | 0.969 | 0.975 | 1 |
| LOC284454 | 0.004165 | -0.36531 | 0.35 | 0.449 | 1 |
| B4GALT1 | 0.004247 | -0.29744 | 0.756 | 0.789 | 1 |
| HSPH1 | 0.004264 | -0.67273 | 0.75 | 0.793 | 1 |
| TBCC | 0.004399 | -0.28842 | 0.606 | 0.662 | 1 |
| IP6K2 | 0.005022 | 0.250729 | 0.719 | 0.618 | 1 |
| SLC38A2 | 0.005268 | -0.35924 | 0.825 | 0.867 | 1 |
| KLF2 | 0.005769 | -0.28406 | 0.175 | 0.259 | 1 |
| TUBA4A | 0.006977 | -0.51325 | 0.931 | 0.906 | 1 |
| DENND2C | 0.007046 | -0.31421 | 0.644 | 0.685 | 1 |
| BCAS2 | 0.007507 | -0.36284 | 0.644 | 0.671 | 1 |
| AMD1 | 0.008099 | -0.3326 | 0.6 | 0.664 | 1 |
| BBIP1 | 0.009345 | -0.27157 | 0.812 | 0.832 | 1 |
| PIK3R1 | 0.009623 | -0.42759 | 0.75 | 0.764 | 1 |
| SLU7 | 0.010053 | 0.28227 | 0.719 | 0.622 | 1 |
| DDX1 | 0.010755 | 0.358165 | 0.519 | 0.427 | 1 |
| CDKN1A | 0.010982 | -0.36451 | 0.35 | 0.422 | 1 |
| RNF125 | 0.012157 | -0.48495 | 0.694 | 0.682 | 1 |
| TUBA1B | 0.013171 | -0.34667 | 0.938 | 0.921 | 1 |
| SLFN5 | 0.013825 | 0.292807 | 0.75 | 0.666 | 1 |
| NR4A1 | 0.016169 | -0.72598 | 0.412 | 0.476 | 1 |
| KCNA3 | 0.01688 | -0.31782 | 0.331 | 0.402 | 1 |
| LITAF | 0.017371 | -0.29543 | 0.931 | 0.931 | 1 |
| UAP1 | 0.018052 | -0.3057 | 0.269 | 0.351 | 1 |
| STMN1 | 0.018859 | -0.36307 | 0.656 | 0.494 | 1 |
| TYMS | 0.019357 | -0.26123 | 0.325 | 0.226 | 1 |
| HMGB2 | 0.020041 | -0.27714 | 0.938 | 0.919 | 1 |
| STK26 | 0.020833 | -0.27128 | 0.325 | 0.387 | 1 |
| SAFB2 | 0.021316 | -0.25118 | 0.625 | 0.676 | 1 |
| CD55 | 0.0219 | -0.32361 | 0.525 | 0.581 | 1 |
| TOB1 | 0.022925 | -0.47639 | 0.794 | 0.842 | 1 |
| HOPX | 0.02432 | 0.290804 | 0.688 | 0.628 | 1 |
| KPNA2 | 0.025971 | -0.36748 | 0.588 | 0.629 | 1 |
| SYTL3 | 0.02701 | -0.26973 | 0.812 | 0.809 | 1 |
| PFKFB3 | 0.03231 | -0.34526 | 0.544 | 0.613 | 1 |
| DENND4A | 0.036121 | -0.2719 | 0.544 | 0.585 | 1 |
| S1PR1 | 0.037265 | -0.29451 | 0.231 | 0.281 | 1 |
| HSPA8 | 0.038738 | -0.38831 | 1 | 0.999 | 1 |
| PLK3 | 0.038852 | -0.30774 | 0.394 | 0.458 | 1 |
| SOCS3 | 0.044928 | -0.27216 | 0.281 | 0.341 | 1 |
| TSPYL2 | 0.046176 | -0.32322 | 0.556 | 0.603 | 1 |
| SORL1 | 0.05186 | -0.41022 | 0.4 | 0.429 | 1 |
| ATF3 | 0.052002 | -0.42132 | 0.225 | 0.284 | 1 |
| SAT1 | 0.055237 | -0.36241 | 0.944 | 0.922 | 1 |
| NEU1 | 0.056431 | -0.349 | 0.456 | 0.518 | 1 |
| KLRC1 | 0.056939 | 0.31757 | 0.556 | 0.489 | 1 |
| IFRD1 | 0.061952 | -0.29299 | 0.625 | 0.68 | 1 |
| DDIT4 | 0.062134 | -0.31421 | 0.906 | 0.89 | 1 |
| KCNQ1OT1 | 0.063462 | -0.29451 | 0.212 | 0.272 | 1 |
| PPP1CB | 0.069036 | -0.28024 | 0.962 | 0.943 | 1 |
| SMAD7 | 0.069483 | -0.25937 | 0.206 | 0.262 | 1 |
| LY9 | 0.069585 | -0.30104 | 0.488 | 0.52 | 1 |
| TSPYL1 | 0.070343 | -0.26707 | 0.744 | 0.779 | 1 |
| HSPE1-MOB4 | 0.071064 | -0.2604 | 0.919 | 0.878 | 1 |
| AHNAK | 0.077123 | -0.29925 | 0.95 | 0.902 | 1 |
| SNHG16 | 0.077207 | -0.28679 | 0.356 | 0.391 | 1 |
| EIF4A3 | 0.078093 | -0.28689 | 0.681 | 0.721 | 1 |
| LRRC8C | 0.080598 | -0.25988 | 0.462 | 0.503 | 1 |
| SNHG12 | 0.092366 | -0.28494 | 0.575 | 0.598 | 1 |
| USP36 | 0.107992 | -0.27828 | 0.556 | 0.59 | 1 |
| HSPE1 | 0.116027 | -0.33623 | 0.775 | 0.755 | 1 |
| NXF1 | 0.121082 | -0.30214 | 0.7 | 0.694 | 1 |
| TIPARP | 0.1258 | -0.25401 | 0.394 | 0.443 | 1 |
| MSL2 | 0.12628 | -0.27982 | 0.288 | 0.33 | 1 |
| ANKRD36 | 0.15787 | -0.2971 | 0.838 | 0.822 | 1 |
| SLC2A3 | 0.163734 | -0.26005 | 0.881 | 0.887 | 1 |
| DNAJB9 | 0.165381 | -0.31427 | 0.512 | 0.524 | 1 |
| HSPA1L | 0.17433 | -0.8388 | 0.6 | 0.602 | 1 |
| ARL4C | 0.176915 | -0.2519 | 0.738 | 0.727 | 1 |
| LOC100130476 | 0.188949 | -0.27433 | 0.581 | 0.583 | 1 |
| BRE-AS1 | 0.191442 | -0.28743 | 0.381 | 0.396 | 1 |
| HSPD1 | 0.20415 | -0.5543 | 0.862 | 0.836 | 1 |
| GPR132 | 0.211175 | -0.31778 | 0.488 | 0.498 | 1 |
| CHMP1B | 0.214267 | -0.27852 | 0.644 | 0.637 | 1 |
| CNOT6L | 0.244824 | -0.256 | 0.881 | 0.869 | 1 |
| REV3L | 0.247329 | -0.25323 | 0.444 | 0.463 | 1 |
| SYPL1 | 0.260045 | -0.28843 | 0.306 | 0.33 | 1 |
| JMJD1C | 0.277591 | -0.29051 | 0.919 | 0.836 | 1 |
| RNF168 | 0.32843 | -0.26564 | 0.531 | 0.542 | 1 |
| SPSB3 | 0.4048 | -0.29388 | 0.644 | 0.594 | 1 |
| EGR1 | 0.409513 | -0.40782 | 0.494 | 0.477 | 1 |
| TMEM2 | 0.410903 | -0.26605 | 0.75 | 0.715 | 1 |
| NCOA2 | 0.477188 | -0.32646 | 0.519 | 0.484 | 1 |
| TNF | 0.531088 | -0.28343 | 0.369 | 0.367 | 1 |
| CCR7 | 0.550575 | -0.63228 | 0.6 | 0.576 | 1 |
| SATB1 | 0.589712 | -0.32906 | 0.419 | 0.412 | 1 |
| FOXP1 | 0.590445 | -0.2503 | 0.825 | 0.739 | 1 |
| HBP1 | 0.602725 | -0.30303 | 0.681 | 0.637 | 1 |
| SELL | 0.789078 | -0.26337 | 0.625 | 0.586 | 1 |
| FCGR3A | 0.846449 | -0.36571 | 0.3 | 0.271 | 1 |
| EP300 | 0.901637 | -0.38882 | 0.594 | 0.542 | 1 |
| DNAJA4 | 0.909935 | -0.29583 | 0.269 | 0.254 | 1 |
| HSPA1B | 0.91422 | -0.61724 | 0.719 | 0.68 | 1 |
| ARRDC3 | 0.920371 | -0.30593 | 0.744 | 0.672 | 1 |
| PCNA | 0.967852 | -0.29643 | 0.5 | 0.448 | 1 |
| **Cluster7** | | | | | |
| GeneSymbol | p_val | logFC | pct.1 | pct.2 | p_val_adj |
| DNAJB1 | 1.55E-35 | -1.81819 | 0.798 | 0.94 | 2.80E-31 |
| TNFAIP3 | 1.00E-27 | -1.26647 | 0.982 | 0.978 | 1.81E-23 |
| NR4A2 | 2.95E-27 | -1.17592 | 0.494 | 0.83 | 5.31E-23 |
| HSPA8 | 4.15E-24 | -1.03257 | 1 | 0.999 | 7.49E-20 |
| CCR1 | 9.68E-24 | 0.665639 | 0.673 | 0.306 | 1.75E-19 |
| S100PBP | 7.12E-23 | 0.414244 | 0.762 | 0.369 | 1.28E-18 |
| BTG2 | 4.93E-22 | -1.11958 | 0.53 | 0.815 | 8.89E-18 |
| SRSF7 | 8.45E-21 | -0.6933 | 1 | 0.994 | 1.52E-16 |
| ETV1 | 3.19E-20 | 0.390663 | 0.542 | 0.217 | 5.76E-16 |
| HSP90AA1 | 3.87E-20 | -1.24556 | 0.994 | 0.997 | 6.97E-16 |
| PPP1R15A | 1.25E-19 | -0.92422 | 0.643 | 0.874 | 2.24E-15 |
| EIF1 | 1.63E-19 | -0.61976 | 1 | 0.999 | 2.95E-15 |
| CXCR6 | 2.30E-18 | 0.656896 | 0.976 | 0.838 | 4.15E-14 |
| RPS6KA1 | 2.75E-18 | 0.400181 | 0.756 | 0.424 | 4.95E-14 |
| DNAJA1 | 8.24E-18 | -0.9801 | 0.839 | 0.925 | 1.49E-13 |
| HSP90AB1 | 8.72E-18 | -0.9457 | 0.988 | 0.993 | 1.57E-13 |
| GIMAP6 | 9.55E-18 | 0.433593 | 0.833 | 0.472 | 1.72E-13 |
| TUBB4B | 1.54E-17 | -0.97877 | 0.458 | 0.707 | 2.78E-13 |
| MR1 | 2.00E-17 | 0.432191 | 0.756 | 0.414 | 3.61E-13 |
| PPP1CC | 3.54E-17 | 0.452529 | 0.863 | 0.529 | 6.38E-13 |
| SLFN5 | 5.10E-17 | 0.413918 | 0.935 | 0.644 | 9.20E-13 |
| PTMA | 9.84E-17 | -0.37432 | 1 | 1 | 1.77E-12 |
| ZNF331 | 1.41E-16 | -1.21572 | 0.524 | 0.735 | 2.54E-12 |
| TRAF3IP3 | 1.59E-16 | 0.413443 | 1 | 0.8 | 2.87E-12 |
| CREM | 1.66E-16 | -1.23001 | 0.714 | 0.856 | 2.99E-12 |
| ZBP1 | 3.72E-16 | 0.256237 | 0.75 | 0.447 | 6.71E-12 |
| BTLA | 5.03E-16 | 0.36111 | 0.423 | 0.164 | 9.07E-12 |
| PPM1M | 5.63E-16 | 0.4395 | 0.738 | 0.425 | 1.01E-11 |
| CXCL13 | 5.95E-16 | 0.608994 | 0.893 | 0.645 | 1.07E-11 |
| TTC24 | 7.76E-16 | 0.319864 | 0.589 | 0.294 | 1.40E-11 |
| TMSB4X | 8.38E-16 | 0.39918 | 1 | 1 | 1.51E-11 |
| FCRL3 | 9.14E-16 | 0.469759 | 0.583 | 0.268 | 1.65E-11 |
| UBC | 1.12E-15 | -0.46872 | 1 | 1 | 2.02E-11 |
| HSPH1 | 1.19E-15 | -1.14528 | 0.619 | 0.809 | 2.14E-11 |
| FOS | 1.25E-15 | -1.45752 | 0.696 | 0.86 | 2.26E-11 |
| PLEKHF1 | 1.30E-15 | 0.25995 | 0.643 | 0.319 | 2.35E-11 |
| RGS2 | 3.74E-15 | -0.92783 | 0.708 | 0.864 | 6.73E-11 |
| CD101 | 4.41E-15 | 0.43301 | 0.732 | 0.447 | 7.95E-11 |
| FAM166A | 5.78E-15 | -0.31409 | 0.262 | 0.547 | 1.04E-10 |
| COQ5 | 6.93E-15 | 0.275123 | 0.458 | 0.194 | 1.25E-10 |
| LMNA | 7.47E-15 | -0.99405 | 0.179 | 0.482 | 1.35E-10 |
| UBASH3A | 9.61E-15 | 0.347545 | 0.792 | 0.483 | 1.73E-10 |
| KLRC4 | 1.09E-14 | 0.418656 | 0.839 | 0.599 | 1.96E-10 |
| ZFP36 | 1.10E-14 | -1.12923 | 0.982 | 0.972 | 1.99E-10 |
| CXCR4 | 1.51E-14 | -0.89041 | 0.988 | 0.992 | 2.72E-10 |
| CCDC141 | 1.83E-14 | 0.396199 | 0.798 | 0.479 | 3.30E-10 |
| NR4A3 | 1.86E-14 | -0.61191 | 0.113 | 0.412 | 3.36E-10 |
| NEDD8 | 2.26E-14 | 0.316568 | 0.881 | 0.658 | 4.07E-10 |
| TOB1 | 3.40E-14 | -1.08118 | 0.714 | 0.851 | 6.13E-10 |
| ATP5E | 3.66E-14 | 0.256596 | 1 | 0.951 | 6.59E-10 |
| NR4A1 | 4.03E-14 | -0.99771 | 0.196 | 0.501 | 7.26E-10 |
| CD2 | 4.04E-14 | 0.408105 | 1 | 0.988 | 7.28E-10 |
| C2orf68 | 4.04E-14 | 0.272393 | 0.72 | 0.419 | 7.28E-10 |
| LIMK1 | 4.56E-14 | 0.33389 | 0.488 | 0.232 | 8.22E-10 |
| GIMAP4 | 6.06E-14 | 0.37194 | 0.976 | 0.813 | 1.09E-09 |
| PARP9 | 8.98E-14 | 0.269739 | 0.94 | 0.673 | 1.62E-09 |
| ARHGAP27 | 1.00E-13 | 0.302503 | 0.631 | 0.343 | 1.81E-09 |
| YPEL5 | 1.03E-13 | -0.9667 | 0.899 | 0.915 | 1.85E-09 |
| TNFSF4 | 1.12E-13 | 0.254516 | 0.696 | 0.379 | 2.02E-09 |
| KLF6 | 1.22E-13 | -0.73929 | 0.982 | 0.986 | 2.21E-09 |
| SARAF | 1.29E-13 | -0.56314 | 1 | 0.997 | 2.32E-09 |
| JUNB | 1.45E-13 | -0.94196 | 0.94 | 0.945 | 2.62E-09 |
| IKZF3 | 1.47E-13 | 0.45013 | 0.994 | 0.894 | 2.65E-09 |
| ZNF302 | 1.48E-13 | 0.291829 | 0.458 | 0.204 | 2.67E-09 |
| CCR5 | 1.52E-13 | 0.376701 | 0.708 | 0.401 | 2.75E-09 |
| LCP2 | 1.55E-13 | 0.309351 | 1 | 0.901 | 2.79E-09 |
| CD3D | 1.56E-13 | 0.401981 | 1 | 0.999 | 2.82E-09 |
| GVINP1 | 1.57E-13 | 0.409276 | 0.714 | 0.42 | 2.83E-09 |
| FAM46C | 1.77E-13 | -0.82082 | 0.47 | 0.708 | 3.18E-09 |
| NCKAP1L | 2.25E-13 | 0.277322 | 0.804 | 0.498 | 4.05E-09 |
| TUBA1A | 2.61E-13 | -0.78325 | 0.661 | 0.829 | 4.70E-09 |
| FTH1 | 3.51E-13 | -0.61534 | 1 | 0.999 | 6.32E-09 |
| APOBEC3D | 3.82E-13 | 0.279045 | 0.839 | 0.562 | 6.89E-09 |
| CSRNP1 | 3.97E-13 | -0.71864 | 0.256 | 0.541 | 7.16E-09 |
| SERTAD1 | 4.37E-13 | -0.73435 | 0.28 | 0.549 | 7.87E-09 |
| DUSP1 | 4.46E-13 | -1.16681 | 0.946 | 0.948 | 8.03E-09 |
| SIT1 | 5.60E-13 | 0.347732 | 0.935 | 0.693 | 1.01E-08 |
| UTS2 | 5.77E-13 | 0.25154 | 0.738 | 0.437 | 1.04E-08 |
| COTL1 | 7.93E-13 | 0.41195 | 1 | 0.95 | 1.43E-08 |
| DDX60 | 9.03E-13 | 0.40511 | 0.774 | 0.492 | 1.63E-08 |
| SIRPG | 1.36E-12 | 0.310962 | 0.952 | 0.717 | 2.45E-08 |
| ITFG1 | 1.73E-12 | 0.295961 | 0.679 | 0.388 | 3.11E-08 |
| CYTH4 | 1.97E-12 | 0.360665 | 0.821 | 0.593 | 3.55E-08 |
| IFIT5 | 2.13E-12 | 0.314602 | 0.435 | 0.198 | 3.84E-08 |
| SAMD9L | 2.24E-12 | 0.29552 | 0.935 | 0.636 | 4.05E-08 |
| HAVCR2 | 2.45E-12 | 0.418893 | 0.94 | 0.703 | 4.42E-08 |
| XPNPEP3 | 2.78E-12 | 0.252762 | 0.893 | 0.624 | 5.01E-08 |
| SLAMF6 | 2.83E-12 | 0.259765 | 0.875 | 0.605 | 5.11E-08 |
| CD84 | 2.92E-12 | 0.368302 | 0.952 | 0.723 | 5.26E-08 |
| TXNIP | 3.30E-12 | 0.613145 | 0.976 | 0.941 | 5.94E-08 |
| HLA-DQA1 | 3.35E-12 | 0.306631 | 0.899 | 0.642 | 6.03E-08 |
| METTL3 | 3.41E-12 | 0.253537 | 0.625 | 0.334 | 6.15E-08 |
| OXNAD1 | 6.70E-12 | 0.334226 | 0.929 | 0.737 | 1.21E-07 |
| FKBP5 | 7.97E-12 | 0.390932 | 0.905 | 0.673 | 1.44E-07 |
| APOBEC3C | 8.12E-12 | 0.354071 | 1 | 0.928 | 1.46E-07 |
| CD52 | 9.46E-12 | 0.389278 | 1 | 0.981 | 1.71E-07 |
| ENTPD1 | 1.00E-11 | 0.504103 | 0.827 | 0.573 | 1.81E-07 |
| DEF6 | 1.09E-11 | 0.324727 | 0.923 | 0.7 | 1.96E-07 |
| SRSF2 | 1.28E-11 | -0.57146 | 0.982 | 0.973 | 2.31E-07 |
| CDKN1A | 1.42E-11 | -0.66193 | 0.19 | 0.44 | 2.56E-07 |
| MYL6 | 1.62E-11 | 0.29598 | 1 | 0.992 | 2.92E-07 |
| CD27 | 1.74E-11 | 0.433445 | 0.958 | 0.796 | 3.13E-07 |
| TRPS1 | 1.95E-11 | 0.266286 | 0.667 | 0.39 | 3.51E-07 |
| SEMA4D | 1.96E-11 | 0.274936 | 0.946 | 0.751 | 3.53E-07 |
| ITGAE | 2.01E-11 | 0.366885 | 0.988 | 0.896 | 3.61E-07 |
| ITGA1 | 2.07E-11 | 0.450834 | 0.935 | 0.813 | 3.74E-07 |
| DGKA | 2.12E-11 | 0.273682 | 0.911 | 0.673 | 3.81E-07 |
| ENTPD1-AS1 | 2.74E-11 | 0.26938 | 0.673 | 0.396 | 4.95E-07 |
| WASHC5 | 3.04E-11 | 0.265088 | 0.542 | 0.284 | 5.48E-07 |
| DMTF1 | 3.35E-11 | 0.324622 | 0.881 | 0.662 | 6.04E-07 |
| TRG-AS1 | 3.82E-11 | 0.299336 | 0.75 | 0.494 | 6.89E-07 |
| EOMES | 4.91E-11 | 0.356224 | 0.798 | 0.512 | 8.84E-07 |
| SIK1 | 5.41E-11 | -0.31026 | 0.101 | 0.346 | 9.76E-07 |
| LOC100130872 | 5.93E-11 | 0.255371 | 0.423 | 0.206 | 1.07E-06 |
| USO1 | 7.12E-11 | 0.273343 | 0.708 | 0.43 | 1.28E-06 |
| ANXA1 | 8.18E-11 | -1.04505 | 0.976 | 0.968 | 1.48E-06 |
| RASAL3 | 9.14E-11 | 0.293033 | 0.952 | 0.753 | 1.65E-06 |
| LOC284454 | 9.34E-11 | -0.5184 | 0.208 | 0.465 | 1.68E-06 |
| KLRB1 | 1.11E-10 | 0.558409 | 0.667 | 0.405 | 2.00E-06 |
| DENND2D | 1.13E-10 | 0.326416 | 0.917 | 0.715 | 2.04E-06 |
| TOX | 1.54E-10 | 0.345677 | 0.881 | 0.632 | 2.77E-06 |
| GMIP | 1.59E-10 | 0.251635 | 0.69 | 0.45 | 2.86E-06 |
| ARPC2 | 1.61E-10 | 0.258587 | 1 | 0.997 | 2.89E-06 |
| ARPC4 | 2.05E-10 | 0.264157 | 1 | 0.87 | 3.70E-06 |
| FAM219B | 2.13E-10 | 0.328616 | 0.464 | 0.231 | 3.85E-06 |
| ADD1 | 2.28E-10 | 0.333433 | 0.744 | 0.518 | 4.10E-06 |
| AP3M1 | 2.34E-10 | 0.270594 | 0.643 | 0.368 | 4.22E-06 |
| GZMA | 2.48E-10 | 0.375808 | 1 | 0.98 | 4.48E-06 |
| CD27-AS1 | 2.56E-10 | 0.380008 | 0.917 | 0.745 | 4.61E-06 |
| GALM | 2.91E-10 | 0.301396 | 0.893 | 0.658 | 5.25E-06 |
| PDE4B | 4.24E-10 | -0.70264 | 0.81 | 0.863 | 7.65E-06 |
| CNDP2 | 4.51E-10 | 0.250472 | 0.75 | 0.507 | 8.12E-06 |
| TTN | 4.63E-10 | 0.367795 | 0.899 | 0.719 | 8.35E-06 |
| PTPN6 | 4.69E-10 | 0.287394 | 0.899 | 0.677 | 8.46E-06 |
| FTX | 4.77E-10 | 0.335607 | 0.423 | 0.212 | 8.59E-06 |
| TUBA1B | 4.84E-10 | -0.8234 | 0.899 | 0.925 | 8.73E-06 |
| MDM4 | 5.20E-10 | 0.327506 | 0.935 | 0.712 | 9.38E-06 |
| MAP4K1 | 5.26E-10 | 0.273048 | 0.952 | 0.801 | 9.48E-06 |
| ZNF737 | 5.54E-10 | 0.280766 | 0.351 | 0.158 | 9.99E-06 |
| RNF167 | 6.27E-10 | 0.275977 | 0.952 | 0.743 | 1.13E-05 |
| CDC123 | 7.64E-10 | 0.30252 | 0.821 | 0.558 | 1.38E-05 |
| RNASEL | 7.82E-10 | 0.255572 | 0.345 | 0.157 | 1.41E-05 |
| LAMTOR1 | 7.83E-10 | 0.250118 | 0.75 | 0.501 | 1.41E-05 |
| TRIP11 | 8.44E-10 | 0.291047 | 0.631 | 0.393 | 1.52E-05 |
| SEMA4A | 8.86E-10 | 0.277566 | 0.571 | 0.323 | 1.60E-05 |
| SOCS3 | 8.97E-10 | -0.5223 | 0.137 | 0.357 | 1.62E-05 |
| ASB2 | 9.22E-10 | 0.279346 | 0.524 | 0.296 | 1.66E-05 |
| FAM160B1 | 9.49E-10 | 0.262463 | 0.738 | 0.477 | 1.71E-05 |
| IER2 | 9.69E-10 | -0.77224 | 0.839 | 0.863 | 1.75E-05 |
| IFRD1 | 1.00E-09 | -0.72202 | 0.524 | 0.692 | 1.81E-05 |
| ESYT1 | 1.00E-09 | 0.300129 | 0.905 | 0.696 | 1.81E-05 |
| GIMAP7 | 1.06E-09 | 0.288351 | 0.97 | 0.799 | 1.91E-05 |
| CD3G | 1.22E-09 | 0.279477 | 0.994 | 0.968 | 2.20E-05 |
| SENP3-EIF4A1 | 1.28E-09 | -0.3881 | 1 | 0.99 | 2.32E-05 |
| RGCC | 1.33E-09 | -0.75214 | 0.577 | 0.742 | 2.40E-05 |
| UCP2 | 1.37E-09 | 0.378432 | 1 | 0.89 | 2.46E-05 |
| ACTR2 | 1.37E-09 | 0.298303 | 0.994 | 0.869 | 2.47E-05 |
| GIMAP2 | 1.43E-09 | 0.335371 | 0.649 | 0.409 | 2.57E-05 |
| CD244 | 1.52E-09 | 0.346788 | 0.774 | 0.537 | 2.74E-05 |
| RPL3 | 1.56E-09 | -0.37319 | 1 | 0.998 | 2.82E-05 |
| DZIP3 | 1.64E-09 | 0.311299 | 0.696 | 0.442 | 2.96E-05 |
| CCDC69 | 1.83E-09 | 0.319908 | 0.792 | 0.543 | 3.31E-05 |
| PMAIP1 | 1.89E-09 | -0.77159 | 0.387 | 0.576 | 3.41E-05 |
| VIPAS39 | 1.93E-09 | 0.302208 | 0.31 | 0.134 | 3.49E-05 |
| CD69 | 1.96E-09 | -0.7168 | 0.97 | 0.982 | 3.54E-05 |
| PSMB9 | 2.03E-09 | 0.278241 | 1 | 0.941 | 3.66E-05 |
| MYADM | 2.25E-09 | -0.80363 | 0.583 | 0.699 | 4.05E-05 |
| ADGRG1 | 2.61E-09 | 0.385459 | 0.661 | 0.424 | 4.71E-05 |
| GIMAP1-GIMAP5 | 2.90E-09 | 0.294867 | 0.94 | 0.771 | 5.22E-05 |
| PPIP5K2 | 2.91E-09 | 0.268865 | 0.69 | 0.457 | 5.24E-05 |
| FOSL2 | 3.42E-09 | -0.55895 | 0.274 | 0.495 | 6.17E-05 |
| APBB1IP | 3.55E-09 | 0.281288 | 0.964 | 0.808 | 6.39E-05 |
| SLC2A3 | 3.78E-09 | -0.64277 | 0.81 | 0.895 | 6.82E-05 |
| CELF2 | 3.94E-09 | 0.275256 | 0.982 | 0.91 | 7.11E-05 |
| METTL8 | 4.48E-09 | 0.286242 | 0.548 | 0.312 | 8.07E-05 |
| FOSB | 4.70E-09 | -0.68822 | 0.625 | 0.729 | 8.48E-05 |
| PSMB8 | 4.79E-09 | 0.321172 | 0.994 | 0.901 | 8.64E-05 |
| GZMB | 4.97E-09 | 0.39274 | 1 | 0.951 | 8.95E-05 |
| H3F3B | 5.04E-09 | -0.30544 | 1 | 0.999 | 9.09E-05 |
| HSPD1 | 5.82E-09 | -1.00715 | 0.81 | 0.842 | 0.000105 |
| GPR171 | 5.93E-09 | 0.320835 | 0.976 | 0.79 | 0.000107 |
| TMBIM4 | 6.06E-09 | 0.274235 | 0.964 | 0.788 | 0.000109 |
| ADA2 | 6.19E-09 | 0.273581 | 0.702 | 0.509 | 0.000112 |
| CD83 | 6.50E-09 | -0.57928 | 0.167 | 0.394 | 0.000117 |
| ZNF3 | 8.00E-09 | 0.272642 | 0.405 | 0.216 | 0.000144 |
| DAD1 | 8.23E-09 | 0.255537 | 0.911 | 0.735 | 0.000148 |
| AFAP1L2 | 8.30E-09 | 0.328611 | 0.536 | 0.305 | 0.00015 |
| EIF4E2 | 1.01E-08 | 0.267453 | 0.702 | 0.456 | 0.000182 |
| JUN | 1.05E-08 | -0.7588 | 0.851 | 0.885 | 0.000189 |
| FKBP1A | 1.06E-08 | 0.28935 | 0.994 | 0.846 | 0.000192 |
| NFKBIZ | 1.15E-08 | -0.75777 | 0.494 | 0.658 | 0.000208 |
| GIMAP5 | 1.19E-08 | 0.268713 | 0.935 | 0.726 | 0.000214 |
| INPP4B | 1.24E-08 | 0.266878 | 0.935 | 0.744 | 0.000223 |
| PRMT2 | 1.37E-08 | 0.259659 | 0.946 | 0.746 | 0.000247 |
| LLPH | 1.38E-08 | 0.273933 | 0.976 | 0.848 | 0.00025 |
| SPN | 1.39E-08 | 0.252146 | 0.935 | 0.749 | 0.00025 |
| SNX17 | 1.47E-08 | 0.273373 | 0.833 | 0.6 | 0.000266 |
| MYL12A | 1.58E-08 | 0.258039 | 1 | 0.989 | 0.000284 |
| FUT8 | 1.79E-08 | 0.270945 | 0.72 | 0.495 | 0.000322 |
| RAB27A | 1.84E-08 | 0.320468 | 0.929 | 0.712 | 0.000332 |
| CKS2 | 1.94E-08 | -0.41814 | 0.321 | 0.515 | 0.000351 |
| GMFG | 2.01E-08 | 0.265808 | 0.964 | 0.831 | 0.000363 |
| TLK1 | 2.40E-08 | 0.286541 | 0.881 | 0.733 | 0.000432 |
| H2AFZ | 2.40E-08 | -0.54608 | 0.929 | 0.895 | 0.000433 |
| MSN | 2.44E-08 | 0.264342 | 1 | 0.981 | 0.00044 |
| CAP1 | 2.64E-08 | 0.268349 | 1 | 0.958 | 0.000477 |
| SPPL2A | 2.69E-08 | 0.258352 | 0.786 | 0.577 | 0.000484 |
| EGR1 | 2.85E-08 | -0.61079 | 0.298 | 0.499 | 0.000515 |
| STAT1 | 2.92E-08 | 0.34192 | 0.97 | 0.813 | 0.000526 |
| RASGEF1B | 2.93E-08 | -0.44027 | 0.214 | 0.434 | 0.000527 |
| GBP1 | 3.75E-08 | 0.251376 | 0.917 | 0.668 | 0.000675 |
| SEPT1 | 4.98E-08 | 0.25476 | 0.994 | 0.925 | 0.000898 |
| SH3BGRL3 | 5.42E-08 | 0.25409 | 1 | 0.998 | 0.000977 |
| ARGLU1 | 5.51E-08 | 0.268539 | 0.994 | 0.901 | 0.000992 |
| APOBEC3G | 5.99E-08 | 0.28 | 0.994 | 0.943 | 0.00108 |
| ZEB1 | 7.24E-08 | 0.274118 | 0.708 | 0.495 | 0.001305 |
| HSPA5 | 7.68E-08 | -0.45014 | 0.952 | 0.947 | 0.001384 |
| TMSB10 | 9.62E-08 | 0.297894 | 1 | 1 | 0.001734 |
| HSPA1B | 1.01E-07 | -1.05137 | 0.607 | 0.692 | 0.001821 |
| C17orf62 | 1.26E-07 | 0.256427 | 0.905 | 0.714 | 0.002269 |
| ATP5G2 | 1.29E-07 | 0.260738 | 0.964 | 0.809 | 0.002317 |
| IFI6 | 1.30E-07 | 0.300263 | 0.881 | 0.626 | 0.002335 |
| ANAPC16 | 1.55E-07 | 0.262229 | 0.982 | 0.848 | 0.002798 |
| RBPJ | 1.61E-07 | 0.327908 | 0.97 | 0.846 | 0.002898 |
| PTPN7 | 1.76E-07 | 0.275766 | 0.976 | 0.863 | 0.003181 |
| NHLRC2 | 1.81E-07 | 0.257286 | 0.429 | 0.24 | 0.003269 |
| IFIT3 | 1.93E-07 | 0.291909 | 0.31 | 0.152 | 0.003479 |
| CISH | 1.97E-07 | 0.315155 | 0.637 | 0.429 | 0.003559 |
| MIR155HG | 2.03E-07 | 0.313131 | 0.702 | 0.486 | 0.003664 |
| TES | 2.08E-07 | 0.257867 | 0.714 | 0.516 | 0.003747 |
| ZNF101 | 2.16E-07 | 0.293575 | 0.595 | 0.403 | 0.003889 |
| PRMT9 | 2.51E-07 | -0.44595 | 0.179 | 0.356 | 0.004518 |
| ZNF791 | 2.58E-07 | 0.289947 | 0.607 | 0.396 | 0.004653 |
| MCTP2 | 2.74E-07 | 0.268027 | 0.637 | 0.426 | 0.004945 |
| TUBA1C | 2.79E-07 | -0.39972 | 0.411 | 0.593 | 0.005026 |
| CTSS | 3.31E-07 | 0.256026 | 0.905 | 0.688 | 0.005966 |
| CD200R1 | 3.33E-07 | 0.276467 | 0.506 | 0.305 | 0.006008 |
| EIF4A3 | 3.39E-07 | -0.62234 | 0.643 | 0.726 | 0.006105 |
| SLC38A2 | 3.89E-07 | -0.6181 | 0.857 | 0.864 | 0.00702 |
| PER1 | 3.96E-07 | -0.40443 | 0.393 | 0.537 | 0.007133 |
| TMEM173 | 4.27E-07 | 0.279508 | 0.744 | 0.552 | 0.007697 |
| IQGAP1 | 4.28E-07 | 0.276092 | 0.982 | 0.945 | 0.007721 |
| HSPA1L | 4.37E-07 | -1.08023 | 0.482 | 0.616 | 0.007887 |
| INPP1 | 4.88E-07 | 0.25898 | 0.381 | 0.209 | 0.008797 |
| ATF3 | 5.00E-07 | -0.58255 | 0.119 | 0.296 | 0.009011 |
| ITGB7 | 5.03E-07 | 0.263678 | 0.899 | 0.755 | 0.009066 |
| PIK3IP1 | 5.17E-07 | 0.289044 | 0.958 | 0.85 | 0.009329 |
| TPM4 | 5.51E-07 | 0.251138 | 0.97 | 0.806 | 0.009926 |
| DUSP2 | 5.57E-07 | -0.84334 | 0.911 | 0.927 | 0.010049 |
| NAA35 | 6.81E-07 | 0.252252 | 0.417 | 0.245 | 0.012277 |
| ITPR1 | 7.42E-07 | 0.259929 | 0.72 | 0.513 | 0.013375 |
| S100A4 | 8.51E-07 | 0.344077 | 0.976 | 0.919 | 0.015337 |
| PDCD1 | 9.91E-07 | 0.267506 | 0.827 | 0.645 | 0.017871 |
| TAGLN2 | 1.30E-06 | -0.44598 | 0.982 | 0.974 | 0.02338 |
| FAM53C | 1.63E-06 | -0.51344 | 0.262 | 0.434 | 0.029465 |
| NAMPT | 1.71E-06 | -0.57082 | 0.512 | 0.639 | 0.030809 |
| SDCBP | 2.00E-06 | -0.65539 | 0.863 | 0.869 | 0.036091 |
| LYST | 2.24E-06 | 0.259127 | 0.994 | 0.882 | 0.040368 |
| ACTR3 | 2.26E-06 | 0.266878 | 1 | 0.947 | 0.040708 |
| DNAJB9 | 2.29E-06 | -0.74877 | 0.423 | 0.534 | 0.041282 |
| ARL4A | 3.33E-06 | -0.35047 | 0.167 | 0.327 | 0.060016 |
| BTG1 | 3.34E-06 | -0.4208 | 1 | 0.994 | 0.0603 |
| SELENOK | 3.50E-06 | -0.35986 | 0.732 | 0.789 | 0.063036 |
| SGK1 | 4.83E-06 | -0.47164 | 0.119 | 0.278 | 0.086987 |
| SNHG1 | 5.71E-06 | -0.56365 | 0.31 | 0.451 | 0.103022 |
| ZFAND5 | 1.18E-05 | -0.53368 | 0.655 | 0.737 | 0.212541 |
| KPNA2 | 1.21E-05 | -0.72207 | 0.56 | 0.632 | 0.21799 |
| KIAA1551 | 1.25E-05 | 0.292152 | 0.988 | 0.926 | 0.226235 |
| MFSD11 | 1.33E-05 | -0.34975 | 0.893 | 0.896 | 0.238918 |
| CCL3 | 1.61E-05 | 0.353173 | 0.827 | 0.668 | 0.289452 |
| RGS1 | 1.66E-05 | -0.42191 | 0.988 | 0.986 | 0.299214 |
| ELL2 | 1.96E-05 | -0.25757 | 0.155 | 0.303 | 0.354084 |
| DNAJB6 | 2.07E-05 | -0.34651 | 0.762 | 0.775 | 0.372397 |
| LINC-PINT | 2.26E-05 | -0.48552 | 0.851 | 0.865 | 0.407317 |
| NEU1 | 2.42E-05 | -0.62113 | 0.393 | 0.526 | 0.436059 |
| GADD45B | 2.53E-05 | -0.48206 | 0.351 | 0.463 | 0.45663 |
| CACYBP | 2.70E-05 | -0.51622 | 0.696 | 0.742 | 0.487127 |
| MYLIP | 2.76E-05 | -0.43914 | 0.244 | 0.379 | 0.498238 |
| NASP | 3.07E-05 | -0.37569 | 0.708 | 0.788 | 0.553362 |
| PLK3 | 3.23E-05 | -0.57424 | 0.357 | 0.463 | 0.582611 |
| DNAJB4 | 3.44E-05 | -0.43355 | 0.155 | 0.306 | 0.620264 |
| TSC22D2 | 3.75E-05 | -0.28297 | 0.19 | 0.336 | 0.676863 |
| IER5 | 4.44E-05 | -0.26484 | 0.149 | 0.291 | 0.79955 |
| UBB | 6.24E-05 | -0.2718 | 1 | 1 | 1 |
| SRSF3 | 6.43E-05 | -0.3506 | 0.994 | 0.959 | 1 |
| LDHA | 6.52E-05 | -0.31868 | 1 | 0.994 | 1 |
| JOSD1 | 7.34E-05 | -0.31068 | 0.173 | 0.308 | 1 |
| TIPARP | 8.77E-05 | -0.56781 | 0.333 | 0.451 | 1 |
| BTG3 | 9.10E-05 | -0.38166 | 0.5 | 0.597 | 1 |
| AMD1 | 0.000103 | -0.60944 | 0.649 | 0.659 | 1 |
| EIF5 | 0.00011 | -0.39451 | 0.935 | 0.904 | 1 |
| ATF4 | 0.000117 | -0.4346 | 0.738 | 0.766 | 1 |
| SRSF6 | 0.000129 | -0.55018 | 0.863 | 0.87 | 1 |
| SQSTM1 | 0.000132 | -0.43352 | 0.893 | 0.886 | 1 |
| LOC100130476 | 0.000151 | -0.42472 | 0.494 | 0.593 | 1 |
| JMJD6 | 0.000152 | -0.53512 | 0.643 | 0.671 | 1 |
| DEDD2 | 0.000167 | -0.51135 | 0.53 | 0.605 | 1 |
| TSC22D3 | 0.000182 | -0.6537 | 0.994 | 0.972 | 1 |
| NOP58 | 0.000183 | -0.43743 | 0.911 | 0.894 | 1 |
| HSPA6 | 0.000243 | -0.99354 | 0.131 | 0.26 | 1 |
| ZC3H12A | 0.00026 | -0.60284 | 0.435 | 0.519 | 1 |
| EZR | 0.000276 | -0.35103 | 0.994 | 0.986 | 1 |
| BRD2 | 0.000306 | -0.54454 | 0.923 | 0.895 | 1 |
| DUSP4 | 0.00031 | -0.59198 | 0.821 | 0.816 | 1 |
| SAMSN1 | 0.000316 | -0.39261 | 0.982 | 0.97 | 1 |
| VPS37B | 0.000335 | -0.29113 | 0.423 | 0.526 | 1 |
| BCL6 | 0.000356 | -0.37976 | 0.155 | 0.265 | 1 |
| RPS15A | 0.000366 | 0.262734 | 0.97 | 0.967 | 1 |
| G3BP2 | 0.000382 | -0.45902 | 0.923 | 0.898 | 1 |
| DDX3X | 0.000385 | -0.44424 | 0.982 | 0.954 | 1 |
| HSPE1 | 0.000553 | -0.49259 | 0.732 | 0.76 | 1 |
| CLK1 | 0.000556 | -0.43961 | 0.976 | 0.945 | 1 |
| CHD1 | 0.000758 | -0.37319 | 0.792 | 0.794 | 1 |
| PPP1CB | 0.000759 | -0.45989 | 0.976 | 0.941 | 1 |
| MAP3K8 | 0.000785 | -0.46228 | 0.381 | 0.478 | 1 |
| TUBA4A | 0.000985 | -0.52479 | 0.911 | 0.909 | 1 |
| ANKRD37 | 0.001598 | -0.34681 | 0.262 | 0.367 | 1 |
| GPR183 | 0.001617 | -0.8842 | 0.75 | 0.758 | 1 |
| BCAS2 | 0.00171 | -0.38688 | 0.637 | 0.672 | 1 |
| ATP1B3 | 0.001714 | -0.35117 | 0.869 | 0.84 | 1 |
| BIRC3 | 0.001823 | -0.28858 | 0.923 | 0.88 | 1 |
| HSPA9 | 0.00194 | -0.29658 | 0.845 | 0.855 | 1 |
| OAT | 0.002314 | -0.37732 | 0.488 | 0.558 | 1 |
| HBP1 | 0.00281 | -0.50831 | 0.595 | 0.647 | 1 |
| ISCA1 | 0.00342 | -0.29321 | 0.333 | 0.432 | 1 |
| GTF2B | 0.003517 | -0.43719 | 0.661 | 0.673 | 1 |
| SNHG12 | 0.003602 | -0.29712 | 0.524 | 0.604 | 1 |
| SAT1 | 0.003987 | -0.43345 | 0.964 | 0.919 | 1 |
| FCRL6 | 0.003992 | -0.48491 | 0.452 | 0.516 | 1 |
| MORF4L2 | 0.004395 | -0.2987 | 0.601 | 0.653 | 1 |
| MAFF | 0.004621 | -0.31512 | 0.244 | 0.342 | 1 |
| DUSP10 | 0.004791 | -0.45812 | 0.637 | 0.685 | 1 |
| TCP1 | 0.005083 | -0.45933 | 0.875 | 0.836 | 1 |
| SBDS | 0.005504 | -0.3952 | 0.625 | 0.643 | 1 |
| ETF1 | 0.005773 | -0.35474 | 0.554 | 0.608 | 1 |
| CHORDC1 | 0.005927 | -0.4218 | 0.631 | 0.639 | 1 |
| USP36 | 0.00664 | -0.33223 | 0.53 | 0.593 | 1 |
| UAP1 | 0.006941 | -0.40533 | 0.274 | 0.351 | 1 |
| ALOX5AP | 0.006962 | 0.268253 | 0.958 | 0.885 | 1 |
| EIF4A2 | 0.007353 | -0.25291 | 0.988 | 0.972 | 1 |
| MRNIP | 0.007472 | -0.27229 | 0.768 | 0.765 | 1 |
| NFKBIA | 0.008221 | -0.46801 | 0.964 | 0.934 | 1 |
| TNF | 0.008393 | -0.53417 | 0.31 | 0.374 | 1 |
| ZEB2 | 0.00877 | -0.31682 | 0.738 | 0.752 | 1 |
| DENND2C | 0.009491 | -0.34802 | 0.69 | 0.68 | 1 |
| NOP56 | 0.009823 | -0.29843 | 0.714 | 0.706 | 1 |
| PTGES3 | 0.00991 | -0.32687 | 0.929 | 0.906 | 1 |
| TBCC | 0.011906 | -0.32441 | 0.625 | 0.66 | 1 |
| SLC3A2 | 0.012048 | -0.37142 | 0.893 | 0.835 | 1 |
| DNTTIP2 | 0.012886 | -0.37237 | 0.714 | 0.706 | 1 |
| GSPT1 | 0.013363 | -0.42982 | 0.619 | 0.611 | 1 |
| IFNGR1 | 0.015263 | -0.43221 | 0.476 | 0.508 | 1 |
| PPP1R15B | 0.015501 | -0.31924 | 0.417 | 0.491 | 1 |
| ICOS | 0.016899 | -0.36194 | 0.798 | 0.797 | 1 |
| PTGER4 | 0.01857 | -0.53609 | 0.774 | 0.739 | 1 |
| PNRC1 | 0.020222 | -0.32703 | 0.905 | 0.888 | 1 |
| HEXIM1 | 0.020603 | -0.32227 | 0.351 | 0.409 | 1 |
| CD44 | 0.021952 | -0.253 | 1 | 0.988 | 1 |
| TOB2 | 0.022294 | -0.37193 | 0.548 | 0.569 | 1 |
| SLC16A1 | 0.02332 | -0.26219 | 0.321 | 0.38 | 1 |
| CD9 | 0.027056 | -0.30364 | 0.232 | 0.298 | 1 |
| RBMX | 0.027076 | -0.4028 | 0.833 | 0.83 | 1 |
| NXT1 | 0.02861 | -0.26969 | 0.327 | 0.368 | 1 |
| RELL1 | 0.029667 | -0.37494 | 0.387 | 0.44 | 1 |
| PFKFB3 | 0.030275 | -0.46111 | 0.583 | 0.609 | 1 |
| SLC25A4 | 0.033127 | -0.35232 | 0.214 | 0.267 | 1 |
| NFE2L2 | 0.033306 | -0.30051 | 0.631 | 0.66 | 1 |
| CHMP1B | 0.035052 | -0.38238 | 0.625 | 0.639 | 1 |
| ITGA5 | 0.036171 | -0.30008 | 0.202 | 0.261 | 1 |
| RALGAPA1 | 0.041657 | -0.30182 | 0.583 | 0.571 | 1 |
| AHSA1 | 0.041956 | -0.38683 | 0.673 | 0.65 | 1 |
| EP300 | 0.046236 | -0.3585 | 0.661 | 0.535 | 1 |
| DCTN6 | 0.046819 | -0.42408 | 0.548 | 0.569 | 1 |
| CCR7 | 0.050737 | -0.48727 | 0.56 | 0.581 | 1 |
| ADRB2 | 0.051459 | -0.27238 | 0.28 | 0.336 | 1 |
| CNOT6L | 0.051851 | -0.32157 | 0.899 | 0.867 | 1 |
| LITAF | 0.054733 | -0.36822 | 0.988 | 0.925 | 1 |
| TYMS | 0.055089 | -0.43037 | 0.321 | 0.226 | 1 |
| ATP2B1 | 0.056736 | -0.39384 | 0.619 | 0.599 | 1 |
| MCL1 | 0.058741 | -0.27929 | 0.97 | 0.937 | 1 |
| IRF4 | 0.05926 | -0.34184 | 0.5 | 0.557 | 1 |
| S1PR1 | 0.059588 | -0.29002 | 0.238 | 0.281 | 1 |
| REL | 0.061347 | -0.383 | 0.708 | 0.728 | 1 |
| SC5D | 0.062395 | -0.27913 | 0.339 | 0.393 | 1 |
| PTP4A1 | 0.067115 | -0.34969 | 0.762 | 0.75 | 1 |
| TSPYL2 | 0.068853 | -0.59173 | 0.595 | 0.599 | 1 |
| PIK3R1 | 0.07044 | -0.41749 | 0.798 | 0.759 | 1 |
| SATB1 | 0.070446 | -0.2839 | 0.381 | 0.416 | 1 |
| HSPE1-MOB4 | 0.070448 | -0.30502 | 0.887 | 0.882 | 1 |
| BRE-AS1 | 0.070587 | -0.2745 | 0.351 | 0.4 | 1 |
| UBE2B | 0.075613 | -0.28524 | 0.774 | 0.744 | 1 |
| DPP4 | 0.085279 | -0.26036 | 0.208 | 0.256 | 1 |
| DDIT3 | 0.085682 | -0.29823 | 0.577 | 0.57 | 1 |
| FAM177A1 | 0.090065 | -0.30218 | 0.577 | 0.606 | 1 |
| SKIL | 0.091925 | -0.43299 | 0.78 | 0.781 | 1 |
| NAA50 | 0.100187 | -0.31348 | 0.833 | 0.787 | 1 |
| HERPUD1 | 0.102644 | -0.34216 | 0.976 | 0.9 | 1 |
| ENOSF1 | 0.102973 | -0.33999 | 0.47 | 0.356 | 1 |
| HAUS3 | 0.103884 | -0.38791 | 0.583 | 0.579 | 1 |
| DENND4A | 0.105052 | -0.32921 | 0.56 | 0.584 | 1 |
| ITPRIP | 0.108044 | -0.36936 | 0.435 | 0.454 | 1 |
| BHLHE40 | 0.108112 | -0.28401 | 0.774 | 0.779 | 1 |
| RANBP2 | 0.110876 | -0.3133 | 0.744 | 0.733 | 1 |
| PHACTR2 | 0.111873 | -0.2716 | 0.512 | 0.396 | 1 |
| HMGB2 | 0.113202 | -0.32785 | 0.97 | 0.915 | 1 |
| ZFAND2A | 0.11884 | -0.40222 | 0.333 | 0.354 | 1 |
| LAMTOR3 | 0.13838 | -0.2634 | 0.494 | 0.385 | 1 |
| TAGAP | 0.149495 | -0.4034 | 0.851 | 0.823 | 1 |
| ZC3HAV1 | 0.153534 | -0.34489 | 0.958 | 0.911 | 1 |
| GLA | 0.159437 | -0.43032 | 0.327 | 0.356 | 1 |
| ZBTB1 | 0.159534 | -0.35319 | 0.768 | 0.731 | 1 |
| INSIG1 | 0.169056 | -0.28931 | 0.44 | 0.459 | 1 |
| NUSAP1 | 0.170876 | -0.26839 | 0.464 | 0.357 | 1 |
| ZFP36L2 | 0.182544 | -0.28794 | 0.988 | 0.974 | 1 |
| IL7R | 0.183709 | -0.76753 | 0.893 | 0.794 | 1 |
| STMN1 | 0.187327 | -0.52897 | 0.607 | 0.499 | 1 |
| ARF4 | 0.201546 | -0.34186 | 0.768 | 0.704 | 1 |
| TUBB | 0.236041 | -0.48986 | 0.935 | 0.786 | 1 |
| GNL3 | 0.237758 | -0.25414 | 0.53 | 0.53 | 1 |
| SMAD7 | 0.250967 | -0.29469 | 0.238 | 0.258 | 1 |
| PAF1 | 0.267295 | -0.26812 | 0.482 | 0.471 | 1 |
| PLIN2 | 0.271425 | -0.39969 | 0.649 | 0.641 | 1 |
| KCNQ1OT1 | 0.273461 | -0.28214 | 0.25 | 0.268 | 1 |
| PRNP | 0.292175 | -0.40315 | 0.738 | 0.658 | 1 |
| LDLRAD4 | 0.294369 | -0.2627 | 0.679 | 0.566 | 1 |
| RNF138 | 0.306401 | -0.28817 | 0.524 | 0.52 | 1 |
| AHR | 0.312427 | -0.30178 | 0.518 | 0.509 | 1 |
| TMEM2 | 0.33537 | -0.27523 | 0.738 | 0.716 | 1 |
| HSPB1 | 0.33651 | -0.40434 | 0.613 | 0.577 | 1 |
| ANKRD28 | 0.358546 | -0.34826 | 0.53 | 0.501 | 1 |
| MED21 | 0.377052 | -0.25316 | 0.339 | 0.337 | 1 |
| MFSD14A | 0.377618 | -0.28925 | 0.78 | 0.696 | 1 |
| EGR2 | 0.387671 | -0.31438 | 0.292 | 0.309 | 1 |
| CCT4 | 0.392413 | -0.27583 | 0.762 | 0.681 | 1 |
| MAT2A | 0.40367 | -0.27793 | 0.839 | 0.758 | 1 |
| BIRC2 | 0.404434 | -0.30262 | 0.649 | 0.635 | 1 |
| JMJD1C | 0.408933 | -0.3111 | 0.887 | 0.839 | 1 |
| NXF1 | 0.437084 | -0.2527 | 0.762 | 0.687 | 1 |
| MCM3 | 0.448897 | -0.25057 | 0.506 | 0.435 | 1 |
| IL21R | 0.464341 | -0.31934 | 0.69 | 0.649 | 1 |
| CD28 | 0.498763 | -0.26813 | 0.488 | 0.457 | 1 |
| IFNG | 0.503056 | -0.60146 | 0.804 | 0.785 | 1 |
| GAS5 | 0.505965 | -0.26269 | 0.833 | 0.771 | 1 |
| GPR65 | 0.511185 | -0.28259 | 0.738 | 0.68 | 1 |
| DNAJA4 | 0.520353 | -0.33407 | 0.25 | 0.256 | 1 |
| HECA | 0.524201 | -0.29431 | 0.494 | 0.419 | 1 |
| TSPYL1 | 0.562278 | -0.29673 | 0.869 | 0.765 | 1 |
| TOPORS | 0.58199 | -0.26805 | 0.613 | 0.524 | 1 |
| P2RY10 | 0.597232 | -0.25051 | 0.81 | 0.736 | 1 |
| TBCD | 0.602237 | -0.26245 | 0.893 | 0.754 | 1 |
| SRRT | 0.608456 | -0.28057 | 0.702 | 0.627 | 1 |
| DOK2 | 0.630774 | -0.27205 | 0.81 | 0.75 | 1 |
| CD55 | 0.639893 | -0.31411 | 0.625 | 0.57 | 1 |
| OSER1 | 0.64929 | -0.28202 | 0.464 | 0.436 | 1 |
| ZNF92 | 0.697996 | -0.25443 | 0.327 | 0.31 | 1 |
| MRPL18 | 0.701865 | -0.25618 | 0.488 | 0.442 | 1 |
| HIF1A | 0.708526 | -0.268 | 0.929 | 0.825 | 1 |
| SORL1 | 0.710903 | -0.37196 | 0.446 | 0.424 | 1 |
| ABCE1 | 0.718601 | -0.2738 | 0.542 | 0.489 | 1 |
| RNF139 | 0.736923 | -0.32268 | 0.506 | 0.478 | 1 |
| WDR74 | 0.789663 | -0.28721 | 0.548 | 0.493 | 1 |
| NUP98 | 0.844416 | -0.25555 | 0.72 | 0.651 | 1 |
| ELMSAN1 | 0.919155 | -0.35005 | 0.72 | 0.635 | 1 |
| SLBP | 0.936922 | -0.28346 | 0.708 | 0.625 | 1 |
| PCNA | 0.939741 | -0.34043 | 0.506 | 0.447 | 1 |
| KIF22 | 0.95122 | -0.27048 | 0.506 | 0.431 | 1 |
| MCM7 | 0.965672 | -0.38818 | 0.565 | 0.476 | 1 |
| KLRC1 | 0.974747 | -0.29318 | 0.548 | 0.489 | 1 |
| RORA | 0.995699 | -0.27048 | 0.756 | 0.683 | 1 |
| **Cluster8** | | | | | |
| GeneSymbol | p_val | logFC | pct.1 | pct.2 | p_val_adj |
| TYMSOS | 3.82E-158 | 0.57976 | 0.69 | 0.038 | 6.89E-154 |
| PCLAF | 7.72E-108 | 1.28906 | 0.738 | 0.11 | 1.39E-103 |
| UHRF1 | 8.53E-100 | 0.435359 | 0.494 | 0.034 | 1.54E-95 |
| TYMS | 7.40E-99 | 1.805817 | 0.81 | 0.171 | 1.33E-94 |
| ZWINT | 7.25E-98 | 1.399087 | 0.667 | 0.094 | 1.31E-93 |
| MCM2 | 9.84E-94 | 1.072131 | 0.756 | 0.135 | 1.77E-89 |
| PKMYT1 | 1.16E-92 | 0.794494 | 0.565 | 0.06 | 2.09E-88 |
| CLSPN | 3.29E-92 | 0.854779 | 0.685 | 0.107 | 5.92E-88 |
| TK1 | 3.61E-92 | 1.253148 | 0.679 | 0.104 | 6.51E-88 |
| PAQR4 | 1.04E-91 | 0.395546 | 0.542 | 0.049 | 1.88E-87 |
| MIR3917 | 3.50E-90 | 0.686235 | 0.81 | 0.191 | 6.31E-86 |
| FAM111B | 1.19E-88 | 0.970993 | 0.5 | 0.046 | 2.14E-84 |
| MKI67 | 2.50E-88 | 1.330769 | 0.786 | 0.166 | 4.50E-84 |
| MCM4 | 9.43E-88 | 1.365976 | 0.81 | 0.186 | 1.70E-83 |
| RAD51AP1 | 5.13E-87 | 0.695187 | 0.488 | 0.043 | 9.25E-83 |
| CDC45 | 1.81E-84 | 0.768354 | 0.47 | 0.041 | 3.25E-80 |
| DTL | 3.27E-84 | 0.885053 | 0.583 | 0.077 | 5.90E-80 |
| HELLS | 3.93E-79 | 0.482562 | 0.685 | 0.12 | 7.08E-75 |
| UBE2C | 6.33E-79 | 0.96601 | 0.429 | 0.035 | 1.14E-74 |
| MELK | 1.88E-77 | 0.876257 | 0.488 | 0.052 | 3.39E-73 |
| MCM10 | 3.66E-76 | 0.529525 | 0.411 | 0.033 | 6.60E-72 |
| CDC6 | 1.25E-75 | 0.623713 | 0.399 | 0.03 | 2.25E-71 |
| GINS2 | 6.13E-75 | 0.828433 | 0.53 | 0.07 | 1.10E-70 |
| SPC25 | 1.24E-74 | 0.551289 | 0.363 | 0.023 | 2.23E-70 |
| NCAPG2 | 1.47E-73 | 0.773727 | 0.643 | 0.119 | 2.66E-69 |
| KIF15 | 1.90E-71 | 0.841739 | 0.452 | 0.049 | 3.42E-67 |
| ENOSF1 | 1.08E-70 | 1.48446 | 0.869 | 0.311 | 1.94E-66 |
| GTSE1 | 3.22E-70 | 0.381437 | 0.381 | 0.03 | 5.80E-66 |
| STMN1 | 2.47E-68 | 2.222223 | 0.917 | 0.463 | 4.45E-64 |
| CDCA8 | 6.81E-68 | 0.73554 | 0.393 | 0.035 | 1.23E-63 |
| SHCBP1 | 7.87E-68 | 0.569113 | 0.369 | 0.029 | 1.42E-63 |
| ATAD5 | 2.56E-66 | 0.783477 | 0.637 | 0.134 | 4.61E-62 |
| TOP2A | 3.32E-66 | 1.315667 | 0.661 | 0.147 | 5.99E-62 |
| WDHD1 | 7.45E-66 | 0.650083 | 0.619 | 0.118 | 1.34E-61 |
| HJURP | 1.36E-65 | 0.505547 | 0.321 | 0.02 | 2.44E-61 |
| CDCA5 | 8.11E-65 | 0.716979 | 0.369 | 0.032 | 1.46E-60 |
| FEN1 | 3.59E-64 | 1.063971 | 0.631 | 0.137 | 6.48E-60 |
| ESCO2 | 1.63E-63 | 0.477655 | 0.387 | 0.037 | 2.94E-59 |
| DIAPH3 | 2.85E-63 | 0.294791 | 0.31 | 0.019 | 5.13E-59 |
| BIRC5 | 2.24E-62 | 0.699018 | 0.393 | 0.041 | 4.05E-58 |
| UBE2T | 2.26E-62 | 0.625635 | 0.595 | 0.112 | 4.07E-58 |
| ASF1B | 3.04E-62 | 1.061678 | 0.565 | 0.112 | 5.47E-58 |
| ASPM | 7.22E-62 | 1.119733 | 0.571 | 0.108 | 1.30E-57 |
| MYBL2 | 3.51E-61 | 0.32397 | 0.268 | 0.012 | 6.32E-57 |
| HMGB3 | 8.78E-61 | 0.29766 | 0.387 | 0.039 | 1.58E-56 |
| PCNA-AS1 | 9.29E-61 | 0.804955 | 0.786 | 0.259 | 1.68E-56 |
| MCM7 | 2.56E-60 | 1.618751 | 0.899 | 0.438 | 4.62E-56 |
| CENPU | 4.13E-60 | 0.639275 | 0.637 | 0.143 | 7.44E-56 |
| CHEK1 | 1.02E-59 | 0.546825 | 0.518 | 0.086 | 1.83E-55 |
| KIF18B | 1.22E-59 | 0.30277 | 0.298 | 0.019 | 2.20E-55 |
| CDK1 | 5.59E-59 | 1.197787 | 0.565 | 0.114 | 1.01E-54 |
| CENPM | 1.60E-58 | 0.696042 | 0.625 | 0.149 | 2.89E-54 |
| DLGAP5 | 3.51E-58 | 0.710337 | 0.351 | 0.034 | 6.32E-54 |
| BRCA1 | 5.09E-58 | 0.519106 | 0.488 | 0.078 | 9.18E-54 |
| KNL1 | 2.16E-57 | 0.84527 | 0.518 | 0.092 | 3.89E-53 |
| CENPK | 4.80E-57 | 0.64293 | 0.798 | 0.249 | 8.65E-53 |
| POLD1 | 2.06E-56 | 0.513351 | 0.536 | 0.099 | 3.72E-52 |
| SKA3 | 2.58E-55 | 0.38749 | 0.28 | 0.018 | 4.64E-51 |
| RRM2 | 5.92E-55 | 2.084883 | 0.56 | 0.123 | 1.07E-50 |
| RAD51 | 8.57E-55 | 0.659174 | 0.387 | 0.048 | 1.54E-50 |
| CCNB2 | 1.17E-54 | 0.767176 | 0.351 | 0.037 | 2.11E-50 |
| POLQ | 2.21E-54 | 0.500184 | 0.345 | 0.035 | 3.99E-50 |
| ANLN | 3.24E-54 | 0.665996 | 0.369 | 0.043 | 5.84E-50 |
| C2orf48 | 4.60E-54 | 0.250843 | 0.262 | 0.016 | 8.29E-50 |
| SMC2 | 7.92E-54 | 0.934272 | 0.714 | 0.219 | 1.43E-49 |
| CDCA7L | 8.74E-54 | 0.541564 | 0.452 | 0.07 | 1.58E-49 |
| STIL | 1.11E-53 | 0.544694 | 0.399 | 0.052 | 2.01E-49 |
| TCF19 | 1.12E-53 | 0.887526 | 0.601 | 0.141 | 2.02E-49 |
| MAD2L1 | 1.37E-53 | 0.958392 | 0.655 | 0.18 | 2.46E-49 |
| GGH | 1.86E-53 | 0.636005 | 0.411 | 0.058 | 3.35E-49 |
| DSCC1 | 2.59E-53 | 0.347669 | 0.304 | 0.025 | 4.66E-49 |
| NCAPG | 3.70E-53 | 0.661657 | 0.47 | 0.079 | 6.68E-49 |
| GINS1 | 4.68E-53 | 0.468133 | 0.327 | 0.032 | 8.44E-49 |
| BUB1B | 5.24E-53 | 0.708136 | 0.44 | 0.07 | 9.45E-49 |
| CENPH | 6.67E-53 | 0.317111 | 0.399 | 0.052 | 1.20E-48 |
| CDT1 | 2.31E-52 | 0.267855 | 0.345 | 0.038 | 4.17E-48 |
| ACOT7 | 3.07E-52 | 0.661901 | 0.75 | 0.214 | 5.54E-48 |
| WDR34 | 1.01E-51 | 0.605909 | 0.518 | 0.106 | 1.82E-47 |
| PCNA | 2.73E-51 | 1.308213 | 0.851 | 0.407 | 4.93E-47 |
| TIMELESS | 4.03E-51 | 0.696319 | 0.506 | 0.099 | 7.26E-47 |
| CDCA7 | 4.19E-51 | 0.6849 | 0.583 | 0.135 | 7.55E-47 |
| MALAT1 | 4.61E-51 | -0.72433 | 1 | 1 | 8.32E-47 |
| MCM3 | 5.80E-51 | 1.151009 | 0.887 | 0.392 | 1.05E-46 |
| EXO1 | 6.12E-51 | 0.524757 | 0.315 | 0.03 | 1.10E-46 |
| DUT | 1.92E-50 | 0.977638 | 0.863 | 0.394 | 3.47E-46 |
| ORC6 | 2.03E-50 | 0.340437 | 0.381 | 0.052 | 3.65E-46 |
| RNASEH2A | 2.06E-50 | 0.561229 | 0.631 | 0.16 | 3.71E-46 |
| TMPO-AS1 | 4.58E-50 | 0.268489 | 0.827 | 0.289 | 8.25E-46 |
| TMEM106C | 6.39E-50 | 1.115492 | 0.821 | 0.35 | 1.15E-45 |
| KIFC1 | 7.38E-49 | 0.307961 | 0.268 | 0.02 | 1.33E-44 |
| E2F1 | 8.78E-49 | 0.269225 | 0.274 | 0.022 | 1.58E-44 |
| SKA1 | 1.15E-48 | 0.349298 | 0.274 | 0.022 | 2.06E-44 |
| WDR76 | 1.54E-48 | 0.539707 | 0.649 | 0.179 | 2.77E-44 |
| NUSAP1 | 2.91E-48 | 1.126673 | 0.792 | 0.319 | 5.25E-44 |
| CDKN3 | 3.00E-48 | 0.601983 | 0.488 | 0.095 | 5.41E-44 |
| MCM5 | 4.36E-48 | 1.199414 | 0.857 | 0.408 | 7.86E-44 |
| SGO1 | 5.33E-48 | 0.385664 | 0.375 | 0.053 | 9.61E-44 |
| TPX2 | 1.25E-47 | 0.818876 | 0.548 | 0.128 | 2.25E-43 |
| CKAP2L | 3.54E-47 | 0.642774 | 0.387 | 0.057 | 6.39E-43 |
| POC1A | 7.63E-47 | 0.359074 | 0.262 | 0.021 | 1.37E-42 |
| OIP5 | 1.12E-46 | 0.253969 | 0.339 | 0.041 | 2.03E-42 |
| RFC2 | 1.15E-46 | 0.711678 | 0.685 | 0.217 | 2.08E-42 |
| RANBP1 | 1.28E-46 | 0.685315 | 0.935 | 0.482 | 2.31E-42 |
| BARD1 | 3.20E-46 | 0.543766 | 0.714 | 0.218 | 5.76E-42 |
| KNTC1 | 5.16E-46 | 0.671474 | 0.667 | 0.212 | 9.30E-42 |
| TROAP | 6.15E-46 | 0.414776 | 0.286 | 0.028 | 1.11E-41 |
| KIF14 | 7.14E-46 | 0.477318 | 0.304 | 0.033 | 1.29E-41 |
| KIF11 | 2.23E-45 | 0.774314 | 0.518 | 0.118 | 4.02E-41 |
| DTYMK | 3.97E-45 | 0.358324 | 0.583 | 0.145 | 7.16E-41 |
| KIF4A | 4.71E-45 | 0.421922 | 0.28 | 0.027 | 8.49E-41 |
| CEP55 | 6.66E-45 | 0.34582 | 0.274 | 0.026 | 1.20E-40 |
| NUDT1 | 3.78E-44 | 0.282557 | 0.685 | 0.198 | 6.82E-40 |
| CDK2 | 6.52E-44 | 0.532123 | 0.625 | 0.176 | 1.18E-39 |
| KIF2C | 1.10E-43 | 0.628391 | 0.411 | 0.074 | 1.99E-39 |
| AURKB | 2.02E-43 | 0.694317 | 0.351 | 0.052 | 3.63E-39 |
| LIG1 | 2.97E-43 | 0.755855 | 0.726 | 0.266 | 5.36E-39 |
| CBX5 | 4.02E-43 | 0.489619 | 0.899 | 0.401 | 7.26E-39 |
| TUBB | 4.41E-43 | 1.76621 | 0.994 | 0.779 | 7.95E-39 |
| CIT | 6.48E-43 | 0.336941 | 0.333 | 0.045 | 1.17E-38 |
| CDCA2 | 6.69E-43 | 0.497696 | 0.298 | 0.035 | 1.21E-38 |
| C19orf48 | 7.27E-43 | 0.497037 | 0.601 | 0.165 | 1.31E-38 |
| GINS3 | 1.21E-42 | 0.339758 | 0.298 | 0.035 | 2.18E-38 |
| NRM | 1.71E-42 | 0.607345 | 0.56 | 0.141 | 3.09E-38 |
| CDC20 | 8.00E-42 | 0.588431 | 0.292 | 0.035 | 1.44E-37 |
| CDCA3 | 1.14E-41 | 0.385198 | 0.256 | 0.024 | 2.05E-37 |
| NUF2 | 1.87E-41 | 0.496748 | 0.351 | 0.055 | 3.37E-37 |
| CSNK1G1 | 4.43E-41 | 0.67249 | 0.923 | 0.507 | 7.99E-37 |
| NSD2 | 6.98E-41 | 0.464366 | 0.69 | 0.235 | 1.26E-36 |
| FANCI | 1.62E-40 | 0.857719 | 0.833 | 0.391 | 2.91E-36 |
| GAPDH | 1.67E-40 | 0.696123 | 1 | 0.999 | 3.01E-36 |
| CTNNAL1 | 3.38E-40 | 0.359456 | 0.536 | 0.133 | 6.10E-36 |
| KIF23 | 3.82E-40 | 0.765439 | 0.423 | 0.086 | 6.89E-36 |
| NCAPD3 | 4.34E-40 | 0.470503 | 0.631 | 0.194 | 7.83E-36 |
| MCM6 | 4.57E-40 | 0.900069 | 0.875 | 0.461 | 8.24E-36 |
| TMPO | 5.83E-40 | 0.918377 | 0.929 | 0.545 | 1.05E-35 |
| DEPDC1B | 1.06E-39 | 0.425071 | 0.298 | 0.039 | 1.91E-35 |
| CARHSP1 | 5.79E-39 | 0.33651 | 0.833 | 0.342 | 1.04E-34 |
| CENPJ | 6.32E-39 | 0.399169 | 0.411 | 0.081 | 1.14E-34 |
| DSN1 | 8.22E-39 | 0.466988 | 0.488 | 0.119 | 1.48E-34 |
| ITGB3BP | 9.47E-39 | 0.32525 | 0.518 | 0.132 | 1.71E-34 |
| PLK4 | 1.28E-38 | 0.466964 | 0.351 | 0.059 | 2.30E-34 |
| HMMR | 2.04E-38 | 0.382986 | 0.274 | 0.032 | 3.69E-34 |
| POLD3 | 2.62E-38 | 0.487199 | 0.548 | 0.152 | 4.72E-34 |
| LSM5 | 4.40E-38 | 0.312414 | 0.756 | 0.288 | 7.93E-34 |
| MTHFD1 | 5.59E-38 | 0.750492 | 0.863 | 0.439 | 1.01E-33 |
| AURKA | 7.81E-38 | 0.466124 | 0.333 | 0.053 | 1.41E-33 |
| DNAJC9 | 1.05E-37 | 0.741863 | 0.851 | 0.427 | 1.89E-33 |
| HIRIP3 | 1.28E-37 | 0.457607 | 0.565 | 0.16 | 2.30E-33 |
| POLE2 | 1.54E-37 | 0.343114 | 0.411 | 0.083 | 2.78E-33 |
| TTK | 2.49E-37 | 0.469669 | 0.256 | 0.029 | 4.49E-33 |
| KIF20A | 2.90E-37 | 0.323225 | 0.381 | 0.068 | 5.23E-33 |
| NDC80 | 3.53E-37 | 0.619325 | 0.595 | 0.189 | 6.36E-33 |
| CHAF1B | 4.02E-37 | 0.354086 | 0.345 | 0.058 | 7.24E-33 |
| SAE1 | 4.32E-37 | 0.728043 | 0.863 | 0.445 | 7.78E-33 |
| CHAF1A | 4.39E-37 | 0.285923 | 0.369 | 0.067 | 7.91E-33 |
| CIP2A | 1.01E-36 | 0.499309 | 0.47 | 0.115 | 1.81E-32 |
| POLA2 | 1.24E-36 | 0.579018 | 0.524 | 0.148 | 2.23E-32 |
| H2AFY | 1.39E-36 | 0.712418 | 0.869 | 0.45 | 2.50E-32 |
| CCNA2 | 2.07E-36 | 0.979698 | 0.649 | 0.244 | 3.73E-32 |
| BRCA2 | 2.37E-36 | 0.569283 | 0.435 | 0.101 | 4.27E-32 |
| SLC43A3 | 3.72E-36 | 0.662902 | 0.72 | 0.273 | 6.70E-32 |
| ZGRF1 | 4.78E-36 | 0.60027 | 0.405 | 0.09 | 8.61E-32 |
| C5orf66 | 4.85E-36 | 0.331154 | 0.774 | 0.315 | 8.74E-32 |
| GMNN | 1.53E-35 | 0.54835 | 0.619 | 0.21 | 2.76E-31 |
| RAD54L | 3.04E-35 | 0.414017 | 0.548 | 0.166 | 5.47E-31 |
| PRC1-AS1 | 4.34E-35 | 0.264195 | 0.399 | 0.084 | 7.82E-31 |
| CENPX | 5.99E-35 | 0.450215 | 0.744 | 0.309 | 1.08E-30 |
| HMGN2 | 7.24E-35 | 0.793437 | 0.994 | 0.901 | 1.31E-30 |
| DNMT1 | 1.36E-34 | 0.557039 | 0.952 | 0.624 | 2.46E-30 |
| RFC3 | 4.48E-34 | 0.520586 | 0.375 | 0.079 | 8.08E-30 |
| PRG2 | 5.50E-34 | 0.39337 | 0.601 | 0.193 | 9.92E-30 |
| FANCD2 | 7.55E-34 | 0.602007 | 0.619 | 0.227 | 1.36E-29 |
| ATAD2 | 9.86E-34 | 0.833347 | 0.72 | 0.329 | 1.78E-29 |
| RBBP8 | 1.34E-33 | 0.487911 | 0.583 | 0.2 | 2.42E-29 |
| SPAG5 | 1.36E-33 | 0.682141 | 0.381 | 0.084 | 2.46E-29 |
| RRM1 | 1.47E-33 | 0.887788 | 0.774 | 0.394 | 2.66E-29 |
| SSRP1 | 2.63E-33 | 0.331687 | 0.679 | 0.254 | 4.75E-29 |
| SLC29A1 | 3.12E-33 | 0.359967 | 0.292 | 0.045 | 5.62E-29 |
| PAICS | 4.76E-33 | 0.524218 | 0.524 | 0.157 | 8.58E-29 |
| PKM | 7.54E-33 | 0.75826 | 1 | 0.9 | 1.36E-28 |
| CDK4 | 7.56E-33 | 0.535333 | 0.833 | 0.426 | 1.36E-28 |
| SMC1A | 1.22E-32 | 0.679763 | 0.929 | 0.562 | 2.21E-28 |
| PRC1 | 1.56E-32 | 0.571847 | 0.488 | 0.141 | 2.82E-28 |
| RAD54B | 2.23E-32 | 0.307703 | 0.321 | 0.058 | 4.02E-28 |
| RAD18 | 4.03E-32 | 0.354569 | 0.47 | 0.127 | 7.27E-28 |
| DCTPP1 | 7.62E-32 | 0.374493 | 0.607 | 0.201 | 1.37E-27 |
| BTG1 | 1.01E-31 | -0.93478 | 0.994 | 0.995 | 1.83E-27 |
| PHGDH | 1.14E-31 | 0.348 | 0.292 | 0.047 | 2.05E-27 |
| MAGOHB | 1.20E-31 | 0.252959 | 0.589 | 0.191 | 2.16E-27 |
| TPI1 | 1.23E-31 | 0.673429 | 1 | 0.94 | 2.21E-27 |
| PHF19 | 1.37E-31 | 0.256162 | 0.506 | 0.142 | 2.47E-27 |
| BUB1 | 1.51E-31 | 0.679096 | 0.554 | 0.182 | 2.72E-27 |
| POLR3K | 1.84E-31 | 0.344322 | 0.583 | 0.189 | 3.31E-27 |
| ECT2 | 1.86E-31 | 0.412793 | 0.369 | 0.081 | 3.35E-27 |
| CENPF | 2.00E-31 | 0.880731 | 0.601 | 0.219 | 3.61E-27 |
| SHMT1 | 2.33E-31 | 0.506942 | 0.5 | 0.151 | 4.21E-27 |
| MRPS16 | 3.26E-31 | 0.456134 | 0.94 | 0.543 | 5.88E-27 |
| DHCR24 | 3.29E-31 | 0.335291 | 0.387 | 0.085 | 5.94E-27 |
| TOPBP1 | 3.42E-31 | 0.344983 | 0.661 | 0.248 | 6.17E-27 |
| CEP152 | 4.91E-31 | 0.472103 | 0.53 | 0.165 | 8.85E-27 |
| RFC5 | 9.93E-31 | 0.460742 | 0.506 | 0.151 | 1.79E-26 |
| CENPQ | 1.20E-30 | 0.298956 | 0.31 | 0.057 | 2.17E-26 |
| EEF1E1 | 1.30E-30 | 0.363914 | 0.685 | 0.274 | 2.35E-26 |
| SUV39H2 | 2.62E-30 | 0.401971 | 0.363 | 0.083 | 4.72E-26 |
| EZH2 | 3.56E-30 | 0.654179 | 0.792 | 0.421 | 6.42E-26 |
| BRIP1 | 4.08E-30 | 0.317612 | 0.298 | 0.054 | 7.36E-26 |
| CENPP | 5.89E-30 | 0.337529 | 0.393 | 0.094 | 1.06E-25 |
| SNRPD1 | 9.32E-30 | 0.346171 | 0.869 | 0.459 | 1.68E-25 |
| NCAPH2 | 9.36E-30 | 0.517483 | 0.804 | 0.395 | 1.69E-25 |
| RACGAP1 | 1.05E-29 | 0.412726 | 0.363 | 0.082 | 1.89E-25 |
| MMS22L | 1.29E-29 | 0.460691 | 0.476 | 0.14 | 2.33E-25 |
| POLA1 | 1.43E-29 | 0.480667 | 0.482 | 0.147 | 2.58E-25 |
| FANCL | 1.52E-29 | 0.389215 | 0.732 | 0.307 | 2.74E-25 |
| TFDP1 | 1.99E-29 | 0.416465 | 0.631 | 0.24 | 3.59E-25 |
| DEK | 2.08E-29 | 0.557906 | 0.994 | 0.783 | 3.75E-25 |
| PRDX3 | 2.10E-29 | 0.544519 | 0.952 | 0.63 | 3.78E-25 |
| MSH2 | 5.48E-29 | 0.616652 | 0.637 | 0.265 | 9.87E-25 |
| LSM2 | 8.93E-29 | 0.29973 | 0.863 | 0.416 | 1.61E-24 |
| HLA-E | 1.15E-28 | -0.52612 | 1 | 1 | 2.07E-24 |
| RAD51C | 1.18E-28 | 0.359074 | 0.565 | 0.198 | 2.12E-24 |
| TUBA1B | 3.20E-28 | 1.537038 | 0.976 | 0.916 | 5.77E-24 |
| ACTB | 3.55E-28 | 0.451595 | 1 | 1 | 6.40E-24 |
| FANCG | 3.98E-28 | 0.448044 | 0.464 | 0.141 | 7.18E-24 |
| INPP1 | 4.61E-28 | 0.31202 | 0.56 | 0.189 | 8.32E-24 |
| HAUS5 | 6.62E-28 | 0.311228 | 0.607 | 0.222 | 1.19E-23 |
| CENPE | 7.66E-28 | 0.566883 | 0.482 | 0.153 | 1.38E-23 |
| FBXO5 | 8.21E-28 | 0.483204 | 0.44 | 0.128 | 1.48E-23 |
| BLM | 1.02E-27 | 0.382938 | 0.512 | 0.166 | 1.85E-23 |
| GNG5 | 1.74E-27 | 0.353685 | 0.97 | 0.637 | 3.14E-23 |
| PARPBP | 1.75E-27 | 0.345814 | 0.345 | 0.079 | 3.15E-23 |
| NCAPH | 2.37E-27 | 0.64619 | 0.458 | 0.145 | 4.28E-23 |
| CCNF | 3.28E-27 | 0.284788 | 0.375 | 0.091 | 5.91E-23 |
| CENPN | 3.52E-27 | 0.526457 | 0.815 | 0.464 | 6.35E-23 |
| MSH6 | 5.85E-27 | 0.422196 | 0.661 | 0.277 | 1.05E-22 |
| SIVA1 | 6.62E-27 | 0.460638 | 0.881 | 0.508 | 1.19E-22 |
| TUBG1 | 6.69E-27 | 0.468116 | 0.595 | 0.24 | 1.21E-22 |
| RBL1 | 7.35E-27 | 0.344569 | 0.631 | 0.256 | 1.32E-22 |
| NUCKS1 | 1.22E-26 | 0.280429 | 0.905 | 0.505 | 2.20E-22 |
| MRPL37 | 1.49E-26 | 0.46872 | 0.738 | 0.342 | 2.69E-22 |
| CDCA4 | 1.92E-26 | 0.500633 | 0.571 | 0.222 | 3.46E-22 |
| ACTL6A | 3.02E-26 | 0.485227 | 0.768 | 0.388 | 5.44E-22 |
| KIF20B | 3.42E-26 | 0.570475 | 0.732 | 0.365 | 6.16E-22 |
| KIF22 | 4.36E-26 | 0.692854 | 0.768 | 0.401 | 7.86E-22 |
| EEF1E1-BLOC1S5 | 4.59E-26 | 0.368948 | 0.792 | 0.397 | 8.27E-22 |
| SNX10 | 7.01E-26 | 0.387532 | 0.696 | 0.3 | 1.26E-21 |
| RFC1 | 8.41E-26 | 0.336185 | 0.875 | 0.485 | 1.52E-21 |
| CCNE2 | 9.30E-26 | 0.587263 | 0.518 | 0.187 | 1.68E-21 |
| ANP32B | 9.54E-26 | 0.581195 | 0.881 | 0.55 | 1.72E-21 |
| RAN | 1.26E-25 | 0.549581 | 1 | 0.915 | 2.27E-21 |
| POLD2 | 2.00E-25 | 0.445439 | 0.565 | 0.214 | 3.61E-21 |
| CALM3 | 2.27E-25 | 0.428051 | 0.994 | 0.757 | 4.09E-21 |
| GOLIM4 | 3.12E-25 | 0.375643 | 0.613 | 0.251 | 5.63E-21 |
| SLC27A2 | 3.60E-25 | 0.379215 | 0.726 | 0.323 | 6.48E-21 |
| PSMC3 | 3.73E-25 | 0.567505 | 0.905 | 0.574 | 6.72E-21 |
| PRIM1 | 4.48E-25 | 0.371373 | 0.554 | 0.21 | 8.07E-21 |
| NME1 | 4.60E-25 | 0.313447 | 0.667 | 0.282 | 8.29E-21 |
| HAUS1 | 4.75E-25 | 0.306375 | 0.577 | 0.225 | 8.57E-21 |
| C1orf112 | 5.65E-25 | 0.286383 | 0.536 | 0.189 | 1.02E-20 |
| MAD2L2 | 6.64E-25 | 0.450613 | 0.78 | 0.391 | 1.20E-20 |
| ATP5G3 | 1.30E-24 | 0.471292 | 0.988 | 0.763 | 2.34E-20 |
| CDC23 | 1.30E-24 | 0.357403 | 0.53 | 0.191 | 2.35E-20 |
| SNRPD3 | 1.34E-24 | 0.339765 | 0.923 | 0.566 | 2.42E-20 |
| GINS4 | 1.42E-24 | 0.266885 | 0.304 | 0.067 | 2.55E-20 |
| ICMT | 1.46E-24 | 0.379177 | 0.577 | 0.225 | 2.63E-20 |
| RFC4 | 1.80E-24 | 0.577765 | 0.714 | 0.421 | 3.24E-20 |
| PFN1 | 1.92E-24 | 0.424084 | 1 | 1 | 3.47E-20 |
| HADH | 2.09E-24 | 0.471655 | 0.536 | 0.204 | 3.77E-20 |
| RPL13 | 2.67E-24 | -0.52312 | 1 | 0.999 | 4.82E-20 |
| SNRNP25 | 3.28E-24 | 0.304929 | 0.488 | 0.166 | 5.91E-20 |
| PARP2 | 3.83E-24 | 0.400447 | 0.387 | 0.111 | 6.90E-20 |
| LMNB1 | 4.01E-24 | 0.294089 | 0.458 | 0.147 | 7.23E-20 |
| TMEM237 | 4.34E-24 | 0.272808 | 0.256 | 0.049 | 7.82E-20 |
| MYO7A | 4.89E-24 | 0.342939 | 0.72 | 0.342 | 8.82E-20 |
| SGO2 | 5.89E-24 | 0.352492 | 0.286 | 0.062 | 1.06E-19 |
| UBR7 | 7.72E-24 | 0.51877 | 0.667 | 0.342 | 1.39E-19 |
| BLVRA | 1.02E-23 | 0.34947 | 0.601 | 0.243 | 1.83E-19 |
| PIDD1 | 1.70E-23 | 0.296935 | 0.363 | 0.101 | 3.06E-19 |
| SNRPB | 1.99E-23 | 0.512395 | 0.988 | 0.86 | 3.59E-19 |
| ACAT2 | 2.07E-23 | 0.53412 | 0.929 | 0.649 | 3.73E-19 |
| PRR11 | 2.55E-23 | 0.323545 | 0.298 | 0.07 | 4.60E-19 |
| NUDT21 | 4.08E-23 | 0.384703 | 0.845 | 0.478 | 7.36E-19 |
| HNRNPA2B1 | 4.32E-23 | 0.484314 | 1 | 0.978 | 7.78E-19 |
| CYB5B | 4.44E-23 | 0.437126 | 0.905 | 0.563 | 8.01E-19 |
| AFAP1L2 | 4.61E-23 | 0.485924 | 0.643 | 0.293 | 8.30E-19 |
| CASP2 | 5.08E-23 | 0.429548 | 0.857 | 0.502 | 9.16E-19 |
| TPGS2 | 5.95E-23 | 0.372412 | 0.774 | 0.416 | 1.07E-18 |
| RBBP7 | 6.89E-23 | 0.515634 | 0.94 | 0.62 | 1.24E-18 |
| HAT1 | 7.04E-23 | 0.621833 | 0.81 | 0.499 | 1.27E-18 |
| VDAC1 | 7.33E-23 | 0.427478 | 0.94 | 0.579 | 1.32E-18 |
| SLC25A5-AS1 | 8.65E-23 | 0.253865 | 0.929 | 0.559 | 1.56E-18 |
| YEATS4 | 9.62E-23 | 0.293012 | 0.583 | 0.235 | 1.74E-18 |
| HMGB2 | 9.90E-23 | 0.986333 | 0.988 | 0.913 | 1.79E-18 |
| MRPL51 | 1.09E-22 | 0.286953 | 0.839 | 0.47 | 1.97E-18 |
| RFWD3 | 1.51E-22 | 0.349094 | 0.589 | 0.249 | 2.72E-18 |
| CEP97 | 1.62E-22 | 0.301311 | 0.423 | 0.133 | 2.91E-18 |
| SMC3 | 1.75E-22 | 0.538464 | 0.97 | 0.666 | 3.16E-18 |
| INPP5F | 2.27E-22 | 0.350408 | 0.548 | 0.22 | 4.10E-18 |
| SYNC | 2.28E-22 | 0.336839 | 0.881 | 0.541 | 4.11E-18 |
| HPRT1 | 2.47E-22 | 0.537888 | 0.768 | 0.418 | 4.45E-18 |
| SNRPA | 2.89E-22 | 0.299031 | 0.679 | 0.327 | 5.20E-18 |
| CCL3 | 2.92E-22 | 0.956821 | 0.899 | 0.66 | 5.26E-18 |
| PMVK | 3.32E-22 | 0.279042 | 0.637 | 0.269 | 5.99E-18 |
| NCAPD2 | 3.52E-22 | 0.428552 | 0.571 | 0.252 | 6.34E-18 |
| TEX30 | 4.39E-22 | 0.381826 | 0.536 | 0.217 | 7.91E-18 |
| TTF2 | 4.88E-22 | 0.391927 | 0.655 | 0.309 | 8.81E-18 |
| SLC25A5 | 5.04E-22 | 0.496787 | 0.976 | 0.821 | 9.09E-18 |
| NDUFA6 | 5.11E-22 | 0.26794 | 0.887 | 0.562 | 9.21E-18 |
| ARPC5 | 6.80E-22 | 0.523829 | 0.994 | 0.878 | 1.23E-17 |
| SKA2 | 6.96E-22 | 0.283352 | 0.81 | 0.44 | 1.25E-17 |
| DLEU2 | 7.44E-22 | 0.259506 | 0.732 | 0.346 | 1.34E-17 |
| SDC4 | 9.29E-22 | 0.273246 | 0.512 | 0.199 | 1.67E-17 |
| ZWILCH | 9.32E-22 | 0.572708 | 0.512 | 0.204 | 1.68E-17 |
| LMCD1 | 9.94E-22 | 0.346687 | 0.333 | 0.09 | 1.79E-17 |
| FH | 1.17E-21 | 0.410885 | 0.536 | 0.217 | 2.11E-17 |
| HNRNPAB | 1.50E-21 | 0.260537 | 0.732 | 0.363 | 2.70E-17 |
| NME1-NME2 | 1.53E-21 | 0.414962 | 0.905 | 0.551 | 2.77E-17 |
| CBX3 | 1.59E-21 | 0.398374 | 0.893 | 0.585 | 2.87E-17 |
| CPSF3 | 1.77E-21 | 0.336421 | 0.625 | 0.271 | 3.19E-17 |
| RPA3 | 1.88E-21 | 0.268093 | 0.863 | 0.524 | 3.39E-17 |
| CXCL13 | 2.11E-21 | 1.104571 | 0.875 | 0.647 | 3.81E-17 |
| DERA | 2.16E-21 | 0.262407 | 0.518 | 0.197 | 3.89E-17 |
| C7orf25 | 2.49E-21 | 0.441891 | 0.988 | 0.774 | 4.49E-17 |
| SLBP | 2.51E-21 | 0.535464 | 0.899 | 0.604 | 4.52E-17 |
| SCCPDH | 2.87E-21 | 0.485927 | 0.577 | 0.254 | 5.17E-17 |
| ANXA5 | 3.32E-21 | 0.587917 | 0.982 | 0.85 | 5.98E-17 |
| PSMA2 | 4.29E-21 | 0.448858 | 0.988 | 0.794 | 7.73E-17 |
| LSM4 | 4.72E-21 | 0.316568 | 0.869 | 0.509 | 8.51E-17 |
| TALDO1 | 4.76E-21 | 0.391674 | 0.905 | 0.562 | 8.59E-17 |
| STOML2 | 4.80E-21 | 0.375595 | 0.774 | 0.401 | 8.65E-17 |
| APOBEC3B | 5.48E-21 | 0.32223 | 0.512 | 0.206 | 9.89E-17 |
| MCMBP | 5.83E-21 | 0.266724 | 0.696 | 0.344 | 1.05E-16 |
| C4orf46 | 6.27E-21 | 0.317865 | 0.339 | 0.097 | 1.13E-16 |
| SMC4 | 7.09E-21 | 0.695448 | 0.923 | 0.65 | 1.28E-16 |
| PSMD14 | 1.02E-20 | 0.463129 | 0.821 | 0.5 | 1.84E-16 |
| SFXN1 | 1.02E-20 | 0.348774 | 0.887 | 0.553 | 1.85E-16 |
| TXLNA | 1.24E-20 | 0.284754 | 0.81 | 0.463 | 2.24E-16 |
| HAUS4 | 1.29E-20 | 0.332057 | 0.458 | 0.166 | 2.33E-16 |
| SMS | 1.32E-20 | 0.261348 | 0.655 | 0.304 | 2.38E-16 |
| THOP1 | 1.45E-20 | 0.258084 | 0.405 | 0.129 | 2.62E-16 |
| FDPS | 1.64E-20 | 0.490215 | 0.827 | 0.499 | 2.96E-16 |
| DDX5 | 1.65E-20 | -0.45778 | 1 | 1 | 2.98E-16 |
| XPO1 | 1.77E-20 | 0.253216 | 0.821 | 0.458 | 3.20E-16 |
| NUP188 | 1.87E-20 | 0.306845 | 0.601 | 0.268 | 3.37E-16 |
| RBBP4 | 2.17E-20 | 0.545495 | 0.935 | 0.707 | 3.92E-16 |
| MYO1E | 2.89E-20 | 0.364768 | 0.464 | 0.177 | 5.20E-16 |
| USP39 | 3.11E-20 | 0.380824 | 0.756 | 0.418 | 5.60E-16 |
| GSTO1 | 3.22E-20 | 0.358395 | 0.881 | 0.526 | 5.80E-16 |
| AK2 | 3.54E-20 | 0.407647 | 0.857 | 0.537 | 6.39E-16 |
| FKBP1A | 4.78E-20 | 0.474651 | 0.988 | 0.847 | 8.62E-16 |
| SGCB | 6.98E-20 | 0.275343 | 0.339 | 0.097 | 1.26E-15 |
| HLTF | 7.20E-20 | 0.342944 | 0.661 | 0.327 | 1.30E-15 |
| MGME1 | 7.37E-20 | 0.316891 | 0.536 | 0.229 | 1.33E-15 |
| HSD17B10 | 1.06E-19 | 0.343192 | 0.875 | 0.509 | 1.90E-15 |
| FAM96A | 1.06E-19 | 0.296693 | 0.768 | 0.392 | 1.92E-15 |
| H2AFV | 1.12E-19 | 0.404436 | 0.911 | 0.629 | 2.01E-15 |
| USP1 | 1.17E-19 | 0.538068 | 0.732 | 0.435 | 2.11E-15 |
| SARAF | 1.23E-19 | -0.68037 | 1 | 0.997 | 2.22E-15 |
| NEMP1 | 1.31E-19 | 0.299656 | 0.435 | 0.157 | 2.36E-15 |
| RTCB | 1.85E-19 | 0.321658 | 0.762 | 0.403 | 3.33E-15 |
| ACADM | 2.07E-19 | 0.392949 | 0.661 | 0.331 | 3.73E-15 |
| TNFSF10 | 2.11E-19 | 0.267929 | 0.869 | 0.502 | 3.81E-15 |
| CORO1C | 2.15E-19 | 0.327412 | 0.554 | 0.238 | 3.88E-15 |
| AKR7A2 | 2.30E-19 | 0.292839 | 0.631 | 0.292 | 4.14E-15 |
| COX5A | 2.48E-19 | 0.381302 | 0.923 | 0.656 | 4.47E-15 |
| SPDL1 | 2.73E-19 | 0.32751 | 0.286 | 0.076 | 4.93E-15 |
| PDIA6 | 4.67E-19 | 0.504178 | 0.952 | 0.782 | 8.42E-15 |
| SNX1 | 4.69E-19 | 0.307819 | 0.875 | 0.542 | 8.45E-15 |
| SNRPC | 5.23E-19 | 0.263529 | 0.815 | 0.447 | 9.43E-15 |
| ATP6V1C2 | 5.92E-19 | 0.408613 | 0.905 | 0.647 | 1.07E-14 |
| IER3IP1 | 6.00E-19 | 0.298841 | 0.762 | 0.421 | 1.08E-14 |
| MIR497HG | 8.16E-19 | 0.359314 | 0.917 | 0.599 | 1.47E-14 |
| PRIM2 | 8.38E-19 | 0.285213 | 0.47 | 0.184 | 1.51E-14 |
| DBI | 1.10E-18 | 0.279118 | 0.958 | 0.654 | 1.98E-14 |
| EBP | 1.16E-18 | 0.416562 | 0.804 | 0.474 | 2.09E-14 |
| AP2S1 | 1.20E-18 | 0.251581 | 0.768 | 0.403 | 2.16E-14 |
| PPM1G | 1.21E-18 | 0.357031 | 0.917 | 0.606 | 2.19E-14 |
| CSRP1 | 1.24E-18 | 0.286372 | 0.762 | 0.399 | 2.24E-14 |
| NDFIP2 | 1.40E-18 | 0.55917 | 0.863 | 0.584 | 2.53E-14 |
| SLC1A4 | 1.60E-18 | 0.29096 | 0.452 | 0.177 | 2.88E-14 |
| LAP3 | 1.68E-18 | 0.299218 | 0.881 | 0.553 | 3.04E-14 |
| GGCT | 1.72E-18 | 0.290088 | 0.476 | 0.195 | 3.09E-14 |
| EXOSC8 | 1.72E-18 | 0.341571 | 0.821 | 0.518 | 3.10E-14 |
| ETV7 | 1.81E-18 | 0.271617 | 0.345 | 0.107 | 3.27E-14 |
| ENO1 | 2.31E-18 | 0.477264 | 1 | 0.974 | 4.17E-14 |
| COPS3 | 2.47E-18 | 0.396499 | 0.762 | 0.425 | 4.46E-14 |
| PTTG1 | 2.50E-18 | 0.300639 | 0.857 | 0.499 | 4.51E-14 |
| PSMD1 | 2.94E-18 | 0.412182 | 0.845 | 0.534 | 5.29E-14 |
| CYB561A3 | 3.08E-18 | 0.313757 | 0.53 | 0.227 | 5.55E-14 |
| VRK1 | 3.17E-18 | 0.463314 | 0.589 | 0.294 | 5.72E-14 |
| CALU | 3.19E-18 | 0.272444 | 0.696 | 0.367 | 5.75E-14 |
| RPL13A | 3.28E-18 | -0.57881 | 1 | 0.999 | 5.91E-14 |
| HNRNPR | 3.87E-18 | 0.459351 | 0.97 | 0.806 | 6.98E-14 |
| RPS3 | 4.68E-18 | -0.4651 | 1 | 1 | 8.44E-14 |
| YWHAH | 5.45E-18 | 0.289077 | 0.714 | 0.394 | 9.82E-14 |
| MLH1 | 5.75E-18 | 0.356043 | 0.643 | 0.327 | 1.04E-13 |
| NME2 | 6.95E-18 | 0.285446 | 0.792 | 0.432 | 1.25E-13 |
| SQLE | 7.42E-18 | 0.273914 | 0.565 | 0.263 | 1.34E-13 |
| NUP107 | 8.16E-18 | 0.364873 | 0.69 | 0.379 | 1.47E-13 |
| TIAM1 | 8.29E-18 | 0.272275 | 0.607 | 0.299 | 1.50E-13 |
| TXN2 | 9.07E-18 | 0.331559 | 0.774 | 0.445 | 1.63E-13 |
| EIF1 | 9.93E-18 | -0.62162 | 1 | 0.999 | 1.79E-13 |
| RAD21 | 1.42E-17 | 0.410581 | 0.94 | 0.732 | 2.57E-13 |
| PSMC3IP | 1.46E-17 | 0.28033 | 0.488 | 0.217 | 2.63E-13 |
| WDR54 | 1.49E-17 | 0.417311 | 0.685 | 0.373 | 2.69E-13 |
| H2AFZ | 1.50E-17 | 0.697263 | 0.97 | 0.89 | 2.70E-13 |
| CKAP5 | 1.64E-17 | 0.327494 | 0.637 | 0.321 | 2.96E-13 |
| GUSB | 2.35E-17 | 0.306168 | 0.756 | 0.419 | 4.23E-13 |
| RHOA | 2.37E-17 | 0.395164 | 1 | 0.978 | 4.27E-13 |
| LUC7L2 | 2.44E-17 | 0.259312 | 0.917 | 0.606 | 4.39E-13 |
| DDX39A | 2.57E-17 | 0.430316 | 0.935 | 0.664 | 4.64E-13 |
| NASP | 2.68E-17 | 0.371218 | 0.982 | 0.756 | 4.82E-13 |
| ARL6IP6 | 2.90E-17 | 0.375055 | 0.792 | 0.477 | 5.23E-13 |
| PRPSAP1 | 2.92E-17 | 0.275667 | 0.506 | 0.21 | 5.27E-13 |
| SRP9 | 3.05E-17 | 0.273412 | 0.946 | 0.664 | 5.49E-13 |
| MRPL13 | 3.15E-17 | 0.306888 | 0.69 | 0.359 | 5.68E-13 |
| MPDU1 | 3.46E-17 | 0.266083 | 0.661 | 0.348 | 6.24E-13 |
| PAK1 | 3.74E-17 | 0.256773 | 0.429 | 0.164 | 6.75E-13 |
| UCHL5 | 4.03E-17 | 0.270012 | 0.905 | 0.634 | 7.26E-13 |
| SARDH | 4.36E-17 | 0.273002 | 0.601 | 0.29 | 7.87E-13 |
| PIGX | 4.93E-17 | 0.252966 | 0.423 | 0.16 | 8.89E-13 |
| SUPT16H | 5.38E-17 | 0.452212 | 0.857 | 0.631 | 9.70E-13 |
| ZFP36L2 | 5.74E-17 | -0.86034 | 0.958 | 0.978 | 1.03E-12 |
| FIBP | 6.05E-17 | 0.300368 | 0.875 | 0.603 | 1.09E-12 |
| PA2G4 | 6.62E-17 | 0.438316 | 0.958 | 0.741 | 1.19E-12 |
| RECQL | 6.83E-17 | 0.319002 | 0.899 | 0.58 | 1.23E-12 |
| NR5A2 | 7.50E-17 | 0.314435 | 0.339 | 0.112 | 1.35E-12 |
| EFTUD2 | 7.59E-17 | 0.352831 | 0.75 | 0.44 | 1.37E-12 |
| ENTPD1 | 8.08E-17 | 0.377192 | 0.845 | 0.571 | 1.46E-12 |
| RPL3 | 8.78E-17 | -0.53196 | 1 | 0.998 | 1.58E-12 |
| BAZ1B | 9.61E-17 | 0.277978 | 0.78 | 0.456 | 1.73E-12 |
| CXCR4 | 1.00E-16 | -0.95059 | 1 | 0.991 | 1.81E-12 |
| PRKDC | 1.01E-16 | 0.374696 | 0.905 | 0.651 | 1.82E-12 |
| OLA1 | 1.03E-16 | 0.320239 | 0.655 | 0.335 | 1.85E-12 |
| HNRNPF | 1.07E-16 | 0.478535 | 0.982 | 0.838 | 1.93E-12 |
| CTPS1 | 1.11E-16 | 0.49228 | 0.518 | 0.24 | 2.01E-12 |
| CMAS | 1.12E-16 | 0.302086 | 0.56 | 0.26 | 2.02E-12 |
| PSMB2 | 1.14E-16 | 0.347223 | 0.946 | 0.687 | 2.05E-12 |
| ATP6V1G2-DDX39B | 1.23E-16 | 0.286163 | 0.917 | 0.673 | 2.22E-12 |
| CYC1 | 1.26E-16 | 0.253641 | 0.744 | 0.409 | 2.27E-12 |
| MXD3 | 1.41E-16 | 0.298299 | 0.893 | 0.612 | 2.54E-12 |
| GMPS | 1.41E-16 | 0.395509 | 0.661 | 0.376 | 2.54E-12 |
| AOAH | 1.68E-16 | -0.79752 | 0.738 | 0.861 | 3.02E-12 |
| SHMT2 | 1.79E-16 | 0.386816 | 0.601 | 0.311 | 3.22E-12 |
| TMX1 | 2.10E-16 | 0.386897 | 0.881 | 0.619 | 3.78E-12 |
| DUSP1 | 2.28E-16 | -1.11515 | 0.97 | 0.945 | 4.10E-12 |
| ZNF79 | 2.40E-16 | 0.322417 | 0.375 | 0.141 | 4.34E-12 |
| UNG | 2.66E-16 | 0.460802 | 0.5 | 0.232 | 4.80E-12 |
| MTHFD2 | 2.92E-16 | 0.309994 | 0.833 | 0.536 | 5.27E-12 |
| CD2BP2 | 3.00E-16 | 0.293709 | 0.845 | 0.514 | 5.41E-12 |
| CCT8 | 3.03E-16 | 0.342337 | 0.94 | 0.679 | 5.46E-12 |
| CSE1L | 3.41E-16 | 0.322698 | 0.792 | 0.486 | 6.15E-12 |
| BCAP29 | 3.51E-16 | 0.326378 | 0.655 | 0.348 | 6.33E-12 |
| PDCD1 | 3.55E-16 | 0.335322 | 0.917 | 0.635 | 6.41E-12 |
| RPA1 | 3.66E-16 | 0.513777 | 0.744 | 0.468 | 6.60E-12 |
| ERG28 | 3.72E-16 | 0.263057 | 0.702 | 0.372 | 6.70E-12 |
| CNP | 3.82E-16 | 0.339534 | 0.708 | 0.407 | 6.88E-12 |
| FIGNL1 | 3.94E-16 | 0.276871 | 0.292 | 0.091 | 7.10E-12 |
| FAM111A | 3.97E-16 | 0.387966 | 0.708 | 0.422 | 7.15E-12 |
| LAT2 | 4.07E-16 | 0.333741 | 0.494 | 0.218 | 7.34E-12 |
| MIS18BP1 | 4.16E-16 | 0.267258 | 0.81 | 0.503 | 7.51E-12 |
| SEPT11 | 4.50E-16 | 0.254863 | 0.72 | 0.394 | 8.12E-12 |
| TOX | 4.53E-16 | 0.381557 | 0.923 | 0.627 | 8.17E-12 |
| SNRNP40 | 4.80E-16 | 0.348257 | 0.756 | 0.476 | 8.65E-12 |
| ID3 | 4.84E-16 | 0.345217 | 0.56 | 0.265 | 8.73E-12 |
| DNAJC8 | 6.18E-16 | 0.282802 | 0.935 | 0.664 | 1.11E-11 |
| GSTP1 | 6.70E-16 | 0.28473 | 0.964 | 0.715 | 1.21E-11 |
| WARS | 8.89E-16 | 0.348249 | 0.714 | 0.425 | 1.60E-11 |
| AHCY | 9.57E-16 | 0.299351 | 0.339 | 0.118 | 1.72E-11 |
| CBFB | 1.02E-15 | 0.309031 | 0.732 | 0.424 | 1.83E-11 |
| CFL1 | 1.10E-15 | 0.262329 | 1 | 1 | 1.98E-11 |
| LIN52 | 1.36E-15 | 0.286865 | 0.44 | 0.187 | 2.46E-11 |
| PPIF | 1.56E-15 | 0.381527 | 0.554 | 0.263 | 2.82E-11 |
| DCTN2 | 1.60E-15 | 0.251895 | 0.815 | 0.484 | 2.88E-11 |
| NABP2 | 1.62E-15 | 0.261547 | 0.542 | 0.256 | 2.93E-11 |
| IDH2 | 1.65E-15 | 0.464344 | 0.988 | 0.893 | 2.97E-11 |
| ANP32E | 1.65E-15 | 0.455304 | 0.976 | 0.841 | 2.97E-11 |
| RTN3 | 1.75E-15 | 0.380874 | 0.786 | 0.474 | 3.15E-11 |
| ERI1 | 1.79E-15 | 0.278664 | 0.595 | 0.307 | 3.23E-11 |
| PARK7 | 1.95E-15 | 0.42235 | 0.982 | 0.873 | 3.52E-11 |
| TOR1A | 1.99E-15 | 0.284427 | 0.738 | 0.429 | 3.59E-11 |
| VPS29 | 2.50E-15 | 0.321715 | 0.899 | 0.645 | 4.50E-11 |
| ATP6V1B2 | 2.78E-15 | 0.325336 | 0.696 | 0.388 | 5.00E-11 |
| CENPL | 2.89E-15 | 0.30546 | 0.375 | 0.147 | 5.21E-11 |
| UQCRC1 | 3.35E-15 | 0.333957 | 0.881 | 0.599 | 6.04E-11 |
| GLRX3 | 3.65E-15 | 0.333092 | 0.72 | 0.413 | 6.58E-11 |
| SCD | 3.75E-15 | 0.26562 | 0.357 | 0.131 | 6.76E-11 |
| CST7 | 4.13E-15 | -0.60548 | 0.994 | 0.982 | 7.44E-11 |
| GPAA1 | 5.17E-15 | 0.33302 | 0.679 | 0.387 | 9.31E-11 |
| TSC22D3 | 5.35E-15 | -1.0139 | 0.982 | 0.973 | 9.65E-11 |
| NEDD1 | 6.29E-15 | 0.257316 | 0.649 | 0.351 | 1.13E-10 |
| NUP93 | 6.88E-15 | 0.282287 | 0.679 | 0.388 | 1.24E-10 |
| TNIP3 | 6.97E-15 | 0.319982 | 0.893 | 0.612 | 1.26E-10 |
| RPA2 | 7.09E-15 | 0.343539 | 0.851 | 0.566 | 1.28E-10 |
| PSMD8 | 8.01E-15 | 0.379295 | 0.946 | 0.719 | 1.44E-10 |
| TNFRSF9 | 8.82E-15 | 0.583791 | 0.81 | 0.611 | 1.59E-10 |
| RBPJ | 8.98E-15 | 0.365737 | 0.976 | 0.846 | 1.62E-10 |
| PON2 | 9.55E-15 | 0.514338 | 0.536 | 0.268 | 1.72E-10 |
| XRCC5 | 1.04E-14 | 0.371535 | 1 | 0.878 | 1.88E-10 |
| EXOSC9 | 1.05E-14 | 0.250678 | 0.732 | 0.445 | 1.89E-10 |
| PFKL | 1.05E-14 | 0.292109 | 0.744 | 0.452 | 1.89E-10 |
| CDC123 | 1.07E-14 | 0.301549 | 0.833 | 0.556 | 1.93E-10 |
| TIMMDC1 | 1.12E-14 | 0.316925 | 0.696 | 0.412 | 2.01E-10 |
| CD44 | 1.17E-14 | -0.6123 | 1 | 0.988 | 2.10E-10 |
| TNFRSF18 | 1.25E-14 | 0.257541 | 0.667 | 0.353 | 2.25E-10 |
| SRSF1 | 1.32E-14 | 0.324043 | 0.923 | 0.677 | 2.38E-10 |
| MRPL15 | 1.42E-14 | 0.303302 | 0.44 | 0.191 | 2.56E-10 |
| ARPC1A | 1.46E-14 | 0.295873 | 0.994 | 0.879 | 2.63E-10 |
| MASTL | 1.62E-14 | 0.41182 | 0.565 | 0.298 | 2.93E-10 |
| ALG5 | 1.83E-14 | 0.263286 | 0.649 | 0.353 | 3.29E-10 |
| CD59 | 2.12E-14 | 0.389394 | 0.536 | 0.265 | 3.82E-10 |
| HLA-DRA | 2.34E-14 | 0.451631 | 0.994 | 0.899 | 4.22E-10 |
| ILF2 | 2.36E-14 | 0.357051 | 0.952 | 0.763 | 4.25E-10 |
| AARS | 2.39E-14 | 0.280196 | 0.542 | 0.272 | 4.32E-10 |
| HAVCR2 | 2.52E-14 | 0.343437 | 0.935 | 0.704 | 4.53E-10 |
| PGD | 2.76E-14 | 0.281729 | 0.577 | 0.306 | 4.98E-10 |
| APOBEC3G | 2.80E-14 | 0.348824 | 1 | 0.942 | 5.05E-10 |
| CORO1A | 2.85E-14 | 0.335912 | 1 | 0.987 | 5.13E-10 |
| ARPC1B | 3.75E-14 | 0.338538 | 1 | 0.915 | 6.76E-10 |
| TBC1D4 | 3.80E-14 | 0.291261 | 0.738 | 0.461 | 6.85E-10 |
| PSMA4 | 3.99E-14 | 0.379029 | 0.923 | 0.702 | 7.19E-10 |
| HLA-DRB5 | 4.15E-14 | 0.512591 | 1 | 0.939 | 7.49E-10 |
| TACC3 | 4.60E-14 | 0.402555 | 0.595 | 0.325 | 8.29E-10 |
| PRPS2 | 7.22E-14 | 0.303932 | 0.601 | 0.329 | 1.30E-09 |
| JUNB | 7.64E-14 | -1.03817 | 0.893 | 0.951 | 1.38E-09 |
| POLR2G | 9.07E-14 | 0.274806 | 0.935 | 0.658 | 1.64E-09 |
| ATP5O | 9.79E-14 | 0.285799 | 0.952 | 0.669 | 1.76E-09 |
| UBL7 | 9.89E-14 | 0.285728 | 0.661 | 0.369 | 1.78E-09 |
| DONSON | 1.09E-13 | 0.319388 | 0.964 | 0.768 | 1.96E-09 |
| GLO1 | 1.11E-13 | 0.293581 | 0.702 | 0.426 | 2.00E-09 |
| PTPN7 | 1.15E-13 | 0.362108 | 0.988 | 0.862 | 2.07E-09 |
| PTPRC | 1.50E-13 | -0.34928 | 1 | 1 | 2.70E-09 |
| YWHAQ | 1.50E-13 | 0.423701 | 1 | 0.92 | 2.70E-09 |
| HADHA | 1.51E-13 | 0.267147 | 0.881 | 0.633 | 2.73E-09 |
| CCT4 | 1.63E-13 | 0.307913 | 0.899 | 0.666 | 2.94E-09 |
| RAD1 | 1.74E-13 | 0.271711 | 0.524 | 0.267 | 3.13E-09 |
| ETFA | 1.88E-13 | 0.261122 | 0.78 | 0.478 | 3.40E-09 |
| PMF1 | 1.96E-13 | 0.285498 | 0.899 | 0.633 | 3.53E-09 |
| PSMB3 | 2.15E-13 | 0.296253 | 0.976 | 0.761 | 3.87E-09 |
| PUF60 | 2.35E-13 | 0.298588 | 0.923 | 0.645 | 4.23E-09 |
| CSNK2B | 2.38E-13 | 0.303329 | 0.958 | 0.729 | 4.28E-09 |
| LY6G5B | 2.46E-13 | 0.298613 | 0.964 | 0.725 | 4.44E-09 |
| FOSB | 2.57E-13 | -0.85158 | 0.542 | 0.739 | 4.63E-09 |
| YPEL5 | 2.82E-13 | -0.98273 | 0.899 | 0.915 | 5.09E-09 |
| NUCB2 | 2.82E-13 | 0.383057 | 0.78 | 0.525 | 5.09E-09 |
| XRCC6 | 2.91E-13 | 0.349252 | 0.988 | 0.9 | 5.25E-09 |
| SUV39H1 | 2.99E-13 | 0.312031 | 0.262 | 0.087 | 5.40E-09 |
| TPM4 | 3.55E-13 | 0.363446 | 0.976 | 0.806 | 6.41E-09 |
| CCT2 | 3.68E-13 | 0.34201 | 0.881 | 0.657 | 6.63E-09 |
| FDFT1 | 3.70E-13 | 0.332202 | 0.881 | 0.641 | 6.68E-09 |
| PHB | 3.80E-13 | 0.258383 | 0.72 | 0.436 | 6.84E-09 |
| TMEM109 | 4.11E-13 | 0.386218 | 0.851 | 0.616 | 7.42E-09 |
| SIRPG | 4.17E-13 | 0.291541 | 0.946 | 0.717 | 7.53E-09 |
| KRT81 | 4.50E-13 | 0.373067 | 0.387 | 0.166 | 8.12E-09 |
| ITGAE | 4.63E-13 | 0.341966 | 1 | 0.894 | 8.35E-09 |
| PDIA4 | 4.67E-13 | 0.259102 | 0.792 | 0.515 | 8.43E-09 |
| DCAF12 | 4.92E-13 | 0.253975 | 0.542 | 0.267 | 8.86E-09 |
| CDC42SE2 | 5.31E-13 | -0.55435 | 0.994 | 0.981 | 9.58E-09 |
| HK1 | 5.55E-13 | 0.267044 | 0.857 | 0.608 | 1.00E-08 |
| PCMT1 | 5.92E-13 | 0.316145 | 0.899 | 0.645 | 1.07E-08 |
| ARF6 | 6.20E-13 | 0.280888 | 0.988 | 0.865 | 1.12E-08 |
| SEC11A | 6.20E-13 | 0.257987 | 0.875 | 0.593 | 1.12E-08 |
| PPA1 | 6.36E-13 | 0.31878 | 0.81 | 0.551 | 1.15E-08 |
| LBR | 6.46E-13 | 0.357983 | 0.952 | 0.767 | 1.16E-08 |
| GALM | 7.88E-13 | 0.362487 | 0.893 | 0.658 | 1.42E-08 |
| GDI2 | 7.91E-13 | 0.261397 | 0.964 | 0.748 | 1.43E-08 |
| PSMG2 | 8.00E-13 | 0.291137 | 0.714 | 0.457 | 1.44E-08 |
| DDB2 | 8.37E-13 | 0.362176 | 0.69 | 0.442 | 1.51E-08 |
| PTMA | 1.01E-12 | 0.29226 | 1 | 1 | 1.82E-08 |
| PGM1 | 1.17E-12 | 0.285177 | 0.542 | 0.286 | 2.11E-08 |
| BRIX1 | 1.79E-12 | 0.252075 | 0.631 | 0.36 | 3.23E-08 |
| ITM2A | 1.92E-12 | 0.349253 | 0.994 | 0.915 | 3.47E-08 |
| HLA-DQA1 | 1.93E-12 | 0.435708 | 0.881 | 0.644 | 3.47E-08 |
| NONO | 2.24E-12 | 0.271157 | 0.994 | 0.838 | 4.04E-08 |
| NDC1 | 2.43E-12 | 0.30366 | 0.333 | 0.136 | 4.39E-08 |
| BATF | 2.51E-12 | 0.269586 | 0.899 | 0.612 | 4.52E-08 |
| ZFP36 | 2.89E-12 | -0.91656 | 0.976 | 0.973 | 5.20E-08 |
| DBNL | 2.94E-12 | 0.271294 | 0.881 | 0.627 | 5.31E-08 |
| IL7R | 3.03E-12 | -1.65786 | 0.762 | 0.809 | 5.47E-08 |
| NCOA4 | 3.15E-12 | 0.289725 | 0.863 | 0.595 | 5.69E-08 |
| YWHAE | 4.08E-12 | 0.274229 | 0.964 | 0.767 | 7.35E-08 |
| NAP1L1 | 4.45E-12 | 0.264553 | 0.988 | 0.786 | 8.03E-08 |
| VPS25 | 4.85E-12 | 0.277112 | 0.571 | 0.315 | 8.74E-08 |
| FARSA | 5.85E-12 | 0.262175 | 0.625 | 0.362 | 1.06E-07 |
| NAGA | 6.47E-12 | 0.284376 | 0.44 | 0.21 | 1.17E-07 |
| CD82 | 6.72E-12 | 0.319917 | 0.982 | 0.832 | 1.21E-07 |
| CDK6 | 6.83E-12 | 0.335136 | 0.792 | 0.549 | 1.23E-07 |
| HMGN1 | 7.32E-12 | 0.280508 | 0.976 | 0.838 | 1.32E-07 |
| HSPA14 | 8.35E-12 | 0.250492 | 0.613 | 0.355 | 1.51E-07 |
| ACOT4 | 8.41E-12 | 0.263856 | 0.286 | 0.108 | 1.52E-07 |
| PPP1CA | 8.50E-12 | 0.306993 | 0.994 | 0.892 | 1.53E-07 |
| HMGB1 | 9.25E-12 | 0.313152 | 1 | 0.959 | 1.67E-07 |
| TXNIP | 9.33E-12 | -0.85932 | 0.97 | 0.942 | 1.68E-07 |
| RPL27A | 1.09E-11 | -0.27086 | 1 | 0.995 | 1.96E-07 |
| PSMC5 | 1.10E-11 | 0.259922 | 0.911 | 0.678 | 1.97E-07 |
| NELFCD | 1.40E-11 | 0.250544 | 0.863 | 0.59 | 2.53E-07 |
| CASP8AP2 | 1.45E-11 | 0.272664 | 0.589 | 0.342 | 2.61E-07 |
| RFX5 | 1.55E-11 | 0.267245 | 0.625 | 0.364 | 2.79E-07 |
| CMTM6 | 1.83E-11 | 0.329835 | 0.923 | 0.747 | 3.29E-07 |
| PTPN6 | 2.00E-11 | 0.389703 | 0.893 | 0.678 | 3.60E-07 |
| NAP1L4 | 2.26E-11 | 0.264644 | 0.964 | 0.828 | 4.07E-07 |
| SLC39A1 | 2.29E-11 | 0.265044 | 0.56 | 0.304 | 4.13E-07 |
| RALY | 2.48E-11 | 0.314986 | 0.94 | 0.767 | 4.46E-07 |
| TRAF5 | 2.80E-11 | 0.321922 | 0.845 | 0.647 | 5.04E-07 |
| STT3A | 3.70E-11 | 0.287979 | 0.649 | 0.406 | 6.67E-07 |
| LAYN | 4.10E-11 | 0.252246 | 0.548 | 0.302 | 7.40E-07 |
| RAB11A | 4.46E-11 | 0.262976 | 0.857 | 0.641 | 8.03E-07 |
| USP14 | 4.65E-11 | 0.26474 | 0.696 | 0.45 | 8.39E-07 |
| ZBED2 | 5.05E-11 | 0.54322 | 0.464 | 0.244 | 9.10E-07 |
| CD74 | 6.65E-11 | 0.322219 | 1 | 0.994 | 1.20E-06 |
| CCP110 | 6.90E-11 | 0.330975 | 0.429 | 0.215 | 1.24E-06 |
| RPS6 | 6.97E-11 | -0.36671 | 1 | 0.998 | 1.26E-06 |
| HACD3 | 8.82E-11 | 0.342025 | 0.601 | 0.367 | 1.59E-06 |
| CEP57 | 9.36E-11 | 0.286157 | 0.815 | 0.582 | 1.69E-06 |
| CD63 | 1.12E-10 | 0.31471 | 0.994 | 0.839 | 2.02E-06 |
| ATP5A1 | 1.17E-10 | 0.331949 | 0.988 | 0.906 | 2.10E-06 |
| PSMB7 | 1.26E-10 | 0.262606 | 0.857 | 0.621 | 2.26E-06 |
| CCT3 | 1.31E-10 | 0.257036 | 0.881 | 0.654 | 2.35E-06 |
| BHLHE40-AS1 | 1.47E-10 | 0.410539 | 0.762 | 0.534 | 2.66E-06 |
| C12orf75 | 1.65E-10 | 0.293077 | 0.952 | 0.76 | 2.97E-06 |
| IFI16 | 2.02E-10 | 0.25619 | 1 | 0.93 | 3.65E-06 |
| TPM3 | 2.25E-10 | 0.281645 | 1 | 0.988 | 4.05E-06 |
| MIF-AS1 | 2.26E-10 | 0.256897 | 0.994 | 0.895 | 4.08E-06 |
| MIF | 2.80E-10 | 0.256919 | 0.994 | 0.895 | 5.05E-06 |
| BUB3 | 2.96E-10 | 0.282176 | 0.976 | 0.845 | 5.34E-06 |
| TKT | 3.08E-10 | 0.255359 | 0.601 | 0.36 | 5.55E-06 |
| CHCHD2 | 3.15E-10 | 0.268488 | 1 | 0.911 | 5.68E-06 |
| GART | 3.48E-10 | 0.268296 | 0.613 | 0.376 | 6.27E-06 |
| ACTR3 | 3.60E-10 | 0.327509 | 0.982 | 0.949 | 6.49E-06 |
| PLSCR1 | 3.70E-10 | 0.282319 | 0.732 | 0.497 | 6.67E-06 |
| PDCD4 | 3.82E-10 | -0.61296 | 0.964 | 0.963 | 6.90E-06 |
| HDAC1 | 4.43E-10 | 0.269031 | 0.869 | 0.669 | 7.99E-06 |
| HAPLN3 | 4.60E-10 | 0.27528 | 0.53 | 0.298 | 8.29E-06 |
| HNRNPK | 4.73E-10 | 0.285106 | 1 | 0.918 | 8.53E-06 |
| UBE2N | 7.88E-10 | 0.271806 | 0.935 | 0.769 | 1.42E-05 |
| RNASEK-C17orf49 | 9.50E-10 | 0.291302 | 1 | 0.909 | 1.71E-05 |
| HINT1 | 1.19E-09 | 0.25593 | 0.988 | 0.856 | 2.14E-05 |
| SLC1A5 | 1.62E-09 | 0.251514 | 0.72 | 0.489 | 2.92E-05 |
| MYL6 | 1.76E-09 | 0.276128 | 1 | 0.992 | 3.17E-05 |
| RPS27 | 1.77E-09 | -0.31559 | 0.774 | 0.847 | 3.19E-05 |
| KLF6 | 2.11E-09 | -0.54923 | 0.97 | 0.987 | 3.81E-05 |
| TNFAIP3 | 2.60E-09 | -0.79467 | 0.988 | 0.977 | 4.69E-05 |
| LDHB | 2.77E-09 | 0.310074 | 0.976 | 0.936 | 4.99E-05 |
| ACTG1 | 3.01E-09 | 0.269345 | 1 | 0.999 | 5.42E-05 |
| FOS | 3.13E-09 | -1.03132 | 0.756 | 0.853 | 5.64E-05 |
| TIGIT | 3.49E-09 | 0.296346 | 0.976 | 0.863 | 6.28E-05 |
| PSMA1 | 3.89E-09 | 0.26508 | 0.982 | 0.915 | 7.01E-05 |
| PSMB9 | 4.18E-09 | 0.251807 | 1 | 0.941 | 7.54E-05 |
| GZMB | 5.56E-09 | 0.286574 | 1 | 0.951 | 0.0001 |
| FAM46C | 5.73E-09 | -0.81972 | 0.571 | 0.696 | 0.000103 |
| HIST1H4C | 6.75E-09 | 0.465485 | 0.935 | 0.746 | 0.000122 |
| LDHA | 6.81E-09 | 0.305069 | 1 | 0.994 | 0.000123 |
| TNFSF4 | 8.77E-09 | 0.554048 | 0.601 | 0.39 | 0.000158 |
| CCND2 | 9.05E-09 | 0.274634 | 0.929 | 0.823 | 0.000163 |
| ALDOA | 1.01E-08 | 0.269583 | 1 | 0.975 | 0.000181 |
| ACAA2 | 1.24E-08 | 0.283109 | 0.75 | 0.554 | 0.000224 |
| SNORA33 | 1.25E-08 | -0.32892 | 0.976 | 0.969 | 0.000225 |
| LINC-PINT | 1.35E-08 | -0.66119 | 0.827 | 0.867 | 0.000244 |
| TNS3 | 1.45E-08 | 0.299379 | 0.256 | 0.11 | 0.000261 |
| RPS12 | 2.29E-08 | -0.32418 | 0.976 | 0.969 | 0.000413 |
| STK17B | 2.69E-08 | -0.34887 | 1 | 0.999 | 0.000486 |
| APOBEC3C | 3.30E-08 | 0.262672 | 0.994 | 0.929 | 0.000595 |
| TC2N | 4.24E-08 | -0.64321 | 0.53 | 0.658 | 0.000765 |
| HPGD | 4.49E-08 | 0.379987 | 0.423 | 0.238 | 0.000809 |
| GEM | 5.65E-08 | 0.278183 | 0.399 | 0.219 | 0.001019 |
| LSP1 | 6.79E-08 | 0.279848 | 1 | 0.988 | 0.001224 |
| DUSP2 | 6.79E-08 | -0.87013 | 0.929 | 0.925 | 0.001225 |
| RPS18 | 7.11E-08 | -0.29848 | 1 | 0.996 | 0.001281 |
| HLA-DRB6 | 8.02E-08 | 0.264849 | 1 | 0.938 | 0.001445 |
| KRT86 | 8.45E-08 | 0.479437 | 0.399 | 0.232 | 0.001523 |
| RPL5 | 9.24E-08 | -0.36332 | 1 | 0.995 | 0.001667 |
| IER2 | 9.37E-08 | -0.74533 | 0.821 | 0.865 | 0.00169 |
| ANXA1 | 1.17E-07 | -0.80603 | 0.958 | 0.97 | 0.002106 |
| RPS14 | 1.20E-07 | -0.28996 | 1 | 0.998 | 0.002157 |
| RPL19 | 1.27E-07 | -0.30018 | 1 | 0.994 | 0.002286 |
| STAT4 | 1.35E-07 | -0.53959 | 0.917 | 0.922 | 0.002432 |
| PBXIP1 | 1.96E-07 | -0.61564 | 0.732 | 0.765 | 0.003532 |
| HLA-DRB1 | 2.29E-07 | 0.268485 | 1 | 0.961 | 0.004129 |
| COTL1 | 2.41E-07 | 0.322745 | 1 | 0.95 | 0.004344 |
| UBC | 2.72E-07 | -0.30311 | 1 | 1 | 0.004911 |
| CTSD | 4.07E-07 | 0.268975 | 0.97 | 0.886 | 0.007342 |
| CD53 | 4.53E-07 | -0.29905 | 1 | 0.991 | 0.00817 |
| CD9 | 1.73E-06 | 0.294505 | 0.44 | 0.275 | 0.031169 |
| GPR183 | 1.79E-06 | -1.25162 | 0.78 | 0.755 | 0.032278 |
| EEF1D | 2.02E-06 | -0.34209 | 0.976 | 0.956 | 0.036361 |
| EIF4A2 | 2.12E-06 | -0.43312 | 0.994 | 0.972 | 0.038184 |
| SRGN | 2.74E-06 | -0.25754 | 1 | 0.998 | 0.049365 |
| GZMK | 3.10E-06 | -0.56673 | 0.815 | 0.858 | 0.055916 |
| ITM2B | 3.76E-06 | -0.38089 | 0.988 | 0.984 | 0.067824 |
| ARL6IP1 | 3.91E-06 | 0.293385 | 0.988 | 0.911 | 0.070552 |
| PNRC1 | 4.90E-06 | -0.5784 | 0.887 | 0.89 | 0.088318 |
| CD55 | 4.99E-06 | -0.75475 | 0.464 | 0.589 | 0.089876 |
| MYADM | 5.50E-06 | -0.7081 | 0.619 | 0.695 | 0.099097 |
| DNAJB1 | 6.39E-06 | -0.9154 | 0.905 | 0.928 | 0.115222 |
| C1orf56 | 7.41E-06 | -0.27302 | 0.869 | 0.907 | 0.133572 |
| CCL5 | 8.55E-06 | -0.25774 | 1 | 1 | 0.154146 |
| H3F3B | 8.82E-06 | -0.25802 | 1 | 0.999 | 0.159014 |
| AHNAK | 9.92E-06 | -0.60926 | 0.917 | 0.906 | 0.178807 |
| RPL8 | 1.01E-05 | -0.27016 | 1 | 0.992 | 0.182002 |
| RPL7A | 1.24E-05 | -0.31521 | 0.976 | 0.957 | 0.223072 |
| PPP1R15A | 1.25E-05 | -0.60734 | 0.839 | 0.852 | 0.225493 |
| PIP4K2A | 1.51E-05 | -0.48282 | 0.97 | 0.955 | 0.271776 |
| IDS | 1.71E-05 | -0.44296 | 0.875 | 0.909 | 0.308579 |
| CD69 | 2.16E-05 | -0.56742 | 0.982 | 0.98 | 0.389977 |
| IFITM1 | 2.64E-05 | -0.31463 | 0.964 | 0.975 | 0.476475 |
| STOM | 2.81E-05 | -0.5749 | 0.881 | 0.882 | 0.506511 |
| SRSF5 | 3.05E-05 | -0.32492 | 0.994 | 0.976 | 0.550753 |
| SAT1 | 4.21E-05 | -0.6708 | 0.958 | 0.92 | 0.758271 |
| BTG2 | 4.21E-05 | -0.82513 | 0.792 | 0.785 | 0.758847 |
| PLXDC1 | 4.24E-05 | -0.44516 | 0.167 | 0.303 | 0.764519 |
| EEF1G | 5.41E-05 | -0.27612 | 0.97 | 0.934 | 0.974831 |
| MCL1 | 6.02E-05 | -0.46184 | 0.964 | 0.938 | 1 |
| ISG20 | 6.66E-05 | -0.43271 | 0.839 | 0.795 | 1 |
| RPL30 | 8.70E-05 | -0.25311 | 0.994 | 0.992 | 1 |
| GIMAP7 | 9.85E-05 | -0.47283 | 0.815 | 0.817 | 1 |
| SYTL3 | 9.95E-05 | -0.41478 | 0.804 | 0.81 | 1 |
| SELPLG | 0.000108 | -0.48958 | 0.952 | 0.916 | 1 |
| CLK1 | 0.000112 | -0.47436 | 0.946 | 0.949 | 1 |
| RPL10 | 0.000114 | -0.29942 | 1 | 0.997 | 1 |
| ITGA5 | 0.000127 | -0.35957 | 0.143 | 0.268 | 1 |
| MGAT4A | 0.000132 | -0.65264 | 0.655 | 0.681 | 1 |
| FYN | 0.000141 | -0.43787 | 0.97 | 0.956 | 1 |
| EML4 | 0.000141 | -0.46333 | 0.905 | 0.92 | 1 |
| FYB1 | 0.000145 | -0.27455 | 1 | 0.984 | 1 |
| PLAC8 | 0.000157 | -0.30141 | 0.149 | 0.261 | 1 |
| CNBP | 0.000169 | -0.25125 | 1 | 0.993 | 1 |
| CYTIP | 0.000169 | -0.31999 | 1 | 0.985 | 1 |
| TRIM22 | 0.000201 | -0.45759 | 0.893 | 0.883 | 1 |
| RBM39 | 0.000232 | -0.35793 | 0.994 | 0.984 | 1 |
| IFITM2 | 0.000237 | -0.32964 | 0.946 | 0.891 | 1 |
| ZNF331 | 0.000287 | -0.75514 | 0.696 | 0.715 | 1 |
| BBIP1 | 0.000292 | -0.48194 | 0.857 | 0.827 | 1 |
| LEPROTL1 | 0.000295 | -0.42374 | 0.946 | 0.931 | 1 |
| DDIT4 | 0.00031 | -0.55613 | 0.893 | 0.892 | 1 |
| PER1 | 0.000312 | -0.40944 | 0.458 | 0.53 | 1 |
| WIPF1 | 0.000346 | -0.30933 | 0.988 | 0.965 | 1 |
| PIK3IP1 | 0.000349 | -0.50341 | 0.851 | 0.862 | 1 |
| KLRK1 | 0.000371 | -0.26023 | 0.994 | 0.986 | 1 |
| TPT1 | 0.000387 | -0.27415 | 1 | 0.99 | 1 |
| LY9 | 0.00041 | -0.60757 | 0.476 | 0.522 | 1 |
| STK4 | 0.000416 | -0.34358 | 0.958 | 0.963 | 1 |
| NEU1 | 0.000435 | -0.63438 | 0.44 | 0.52 | 1 |
| JUN | 0.000483 | -0.52625 | 0.905 | 0.879 | 1 |
| TMEM2 | 0.000486 | -0.59882 | 0.679 | 0.723 | 1 |
| CD37 | 0.000536 | -0.32121 | 0.815 | 0.823 | 1 |
| KDM6B | 0.000537 | -0.25621 | 0.25 | 0.365 | 1 |
| RNF125 | 0.000562 | -0.52668 | 0.649 | 0.687 | 1 |
| PIM2 | 0.000711 | -0.61737 | 0.857 | 0.833 | 1 |
| IRF1 | 0.000715 | -0.43571 | 0.97 | 0.951 | 1 |
| KMT2E | 0.000729 | -0.35136 | 1 | 0.963 | 1 |
| FTH1 | 0.000779 | -0.43556 | 1 | 0.999 | 1 |
| TOB1 | 0.000814 | -0.78948 | 0.857 | 0.835 | 1 |
| PABPC1 | 0.000912 | -0.39089 | 0.994 | 0.971 | 1 |
| NR4A3 | 0.001007 | -0.4164 | 0.286 | 0.392 | 1 |
| PDE4B | 0.001103 | -0.53943 | 0.893 | 0.854 | 1 |
| SPOCK2 | 0.001196 | -0.45465 | 0.804 | 0.795 | 1 |
| TSPYL1 | 0.001306 | -0.53201 | 0.774 | 0.775 | 1 |
| SLC2A3 | 0.001346 | -0.46823 | 0.857 | 0.89 | 1 |
| C5orf56 | 0.001448 | -0.29788 | 0.863 | 0.834 | 1 |
| CCNL1 | 0.001564 | -0.38634 | 0.952 | 0.909 | 1 |
| IFNGR1 | 0.001636 | -0.53144 | 0.429 | 0.514 | 1 |
| AUTS2 | 0.001817 | -0.30575 | 0.22 | 0.32 | 1 |
| NOP53 | 0.001831 | -0.30406 | 0.601 | 0.623 | 1 |
| CNOT6L | 0.001854 | -0.49558 | 0.863 | 0.871 | 1 |
| ERN1 | 0.002008 | -0.28698 | 0.304 | 0.388 | 1 |
| TSPYL2 | 0.002028 | -0.6882 | 0.56 | 0.603 | 1 |
| SATB1 | 0.002132 | -0.48602 | 0.351 | 0.419 | 1 |
| SNURF | 0.002522 | -0.27249 | 0.774 | 0.748 | 1 |
| TMEM71 | 0.002683 | -0.33575 | 0.28 | 0.39 | 1 |
| ELF1 | 0.002975 | -0.35709 | 0.994 | 0.952 | 1 |
| RARRES3 | 0.003023 | -0.2593 | 0.964 | 0.905 | 1 |
| B4GALT1 | 0.003252 | -0.38343 | 0.798 | 0.785 | 1 |
| LMNA | 0.003477 | -0.88009 | 0.393 | 0.457 | 1 |
| IQGAP2 | 0.003607 | -0.53108 | 0.589 | 0.61 | 1 |
| JAML | 0.003799 | -0.42553 | 0.821 | 0.846 | 1 |
| ANKRD28 | 0.003831 | -0.27582 | 0.649 | 0.487 | 1 |
| S1PR1 | 0.004151 | -0.34621 | 0.214 | 0.283 | 1 |
| FCGR3A | 0.004485 | -0.59932 | 0.196 | 0.282 | 1 |
| SLAMF7 | 0.004544 | -0.52641 | 0.833 | 0.783 | 1 |
| PARP8 | 0.004736 | -0.50766 | 0.869 | 0.856 | 1 |
| RSRP1 | 0.005738 | -0.39074 | 0.929 | 0.908 | 1 |
| SORL1 | 0.005752 | -0.58497 | 0.393 | 0.43 | 1 |
| AKAP13 | 0.006164 | -0.29932 | 0.982 | 0.954 | 1 |
| RNF149 | 0.006363 | -0.41135 | 0.917 | 0.87 | 1 |
| LITAF | 0.006433 | -0.44473 | 0.946 | 0.93 | 1 |
| NR4A2 | 0.006768 | -0.4203 | 0.786 | 0.797 | 1 |
| NABP1 | 0.007409 | -0.50169 | 0.476 | 0.535 | 1 |
| TSPAN31 | 0.008145 | -0.27885 | 0.476 | 0.329 | 1 |
| DNAJB9 | 0.008196 | -0.5592 | 0.482 | 0.527 | 1 |
| VAMP2 | 0.008411 | -0.27621 | 0.94 | 0.855 | 1 |
| CCR7 | 0.008457 | -0.88978 | 0.601 | 0.576 | 1 |
| CHURC1 | 0.008744 | -0.25447 | 0.827 | 0.648 | 1 |
| GADD45B | 0.008924 | -0.46121 | 0.405 | 0.457 | 1 |
| FOSL2 | 0.009221 | -0.4666 | 0.429 | 0.478 | 1 |
| SRSF7 | 0.009265 | -0.26124 | 1 | 0.994 | 1 |
| BTN3A1 | 0.01021 | -0.43168 | 0.804 | 0.767 | 1 |
| UTRN | 0.010816 | -0.42478 | 0.917 | 0.873 | 1 |
| NDRG1 | 0.011415 | -0.32547 | 0.351 | 0.405 | 1 |
| GZMH | 0.011577 | -0.37184 | 0.976 | 0.935 | 1 |
| EGR1 | 0.011814 | -0.55191 | 0.435 | 0.484 | 1 |
| DNAJA1 | 0.012322 | -0.52413 | 0.952 | 0.912 | 1 |
| TGFBR3 | 0.014151 | -0.30639 | 0.232 | 0.305 | 1 |
| SKIL | 0.014231 | -0.63709 | 0.786 | 0.781 | 1 |
| ABLIM1 | 0.014725 | -0.36733 | 0.643 | 0.683 | 1 |
| ARL4C | 0.015578 | -0.38478 | 0.738 | 0.727 | 1 |
| JMJD1C | 0.016764 | -0.54151 | 0.917 | 0.836 | 1 |
| ITGB2-AS1 | 0.017518 | -0.29948 | 0.357 | 0.412 | 1 |
| SCML4 | 0.017788 | -0.34982 | 0.726 | 0.703 | 1 |
| SNRPN | 0.018097 | -0.35722 | 0.863 | 0.84 | 1 |
| RALGAPA1 | 0.019878 | -0.30509 | 0.56 | 0.574 | 1 |
| GIMAP1-GIMAP5 | 0.020197 | -0.44553 | 0.804 | 0.786 | 1 |
| MBP | 0.020567 | -0.39879 | 0.667 | 0.659 | 1 |
| PPP1R15B | 0.021787 | -0.26667 | 0.619 | 0.468 | 1 |
| TIPARP | 0.022226 | -0.49332 | 0.399 | 0.443 | 1 |
| REL | 0.022393 | -0.35711 | 0.72 | 0.727 | 1 |
| SERINC1 | 0.022503 | -0.39735 | 0.94 | 0.88 | 1 |
| SMAD7 | 0.023838 | -0.27465 | 0.19 | 0.264 | 1 |
| ITK | 0.0242 | -0.35659 | 0.946 | 0.905 | 1 |
| LINC00861 | 0.024208 | -0.45562 | 0.5 | 0.5 | 1 |
| KLF2 | 0.026796 | -0.30565 | 0.208 | 0.256 | 1 |
| FCRL6 | 0.027212 | -0.43511 | 0.458 | 0.515 | 1 |
| PPP2R2B | 0.027745 | -0.36739 | 0.393 | 0.453 | 1 |
| GZMM | 0.028086 | -0.37419 | 0.494 | 0.482 | 1 |
| ACAP1 | 0.029201 | -0.25968 | 0.952 | 0.901 | 1 |
| SLC38A1 | 0.029311 | -0.33657 | 0.976 | 0.951 | 1 |
| ADRB2 | 0.029735 | -0.43746 | 0.28 | 0.336 | 1 |
| C16orf54 | 0.033442 | -0.36586 | 0.863 | 0.796 | 1 |
| CIRBP | 0.03345 | -0.25957 | 0.905 | 0.854 | 1 |
| P2RY10 | 0.033678 | -0.32256 | 0.756 | 0.742 | 1 |
| ZBTB20 | 0.033802 | -0.32284 | 0.571 | 0.572 | 1 |
| CREM | 0.035031 | -0.66763 | 0.887 | 0.836 | 1 |
| ZC3HAV1 | 0.036585 | -0.36163 | 0.929 | 0.915 | 1 |
| PVRIG | 0.037857 | -0.34476 | 0.827 | 0.776 | 1 |
| PLK3 | 0.037942 | -0.38202 | 0.417 | 0.456 | 1 |
| PATL2 | 0.038705 | -0.57281 | 0.446 | 0.469 | 1 |
| MX2 | 0.039778 | -0.28445 | 0.292 | 0.34 | 1 |
| KLRG1 | 0.041524 | -0.36662 | 0.548 | 0.593 | 1 |
| ANKRD12 | 0.042013 | -0.37514 | 0.923 | 0.888 | 1 |
| CRYBG1 | 0.045866 | -0.43849 | 0.625 | 0.637 | 1 |
| CD48 | 0.046123 | -0.39847 | 0.827 | 0.775 | 1 |
| CHD2 | 0.049776 | -0.30443 | 0.935 | 0.901 | 1 |
| PLIN2 | 0.049819 | -0.26612 | 0.756 | 0.629 | 1 |
| RBL2 | 0.050389 | -0.42515 | 0.917 | 0.856 | 1 |
| RBM27 | 0.051143 | -0.29074 | 0.714 | 0.576 | 1 |
| FBXO32 | 0.052086 | -0.31287 | 0.274 | 0.315 | 1 |
| ADAM8 | 0.059748 | -0.27698 | 0.524 | 0.555 | 1 |
| HECA | 0.06013 | -0.42167 | 0.399 | 0.43 | 1 |
| ATF7IP | 0.062833 | -0.31347 | 0.982 | 0.93 | 1 |
| IL10RA | 0.0632 | -0.37048 | 0.923 | 0.859 | 1 |
| SELL | 0.063559 | -0.66764 | 0.696 | 0.578 | 1 |
| SYNE2 | 0.064629 | -0.27794 | 0.994 | 0.967 | 1 |
| AKNA | 0.064941 | -0.35512 | 0.815 | 0.779 | 1 |
| RORA | 0.066333 | -0.31522 | 0.655 | 0.694 | 1 |
| GABARAPL1 | 0.066387 | -0.41408 | 0.81 | 0.758 | 1 |
| A2M | 0.066479 | -0.31747 | 0.351 | 0.384 | 1 |
| FOXP1 | 0.06764 | -0.38565 | 0.726 | 0.75 | 1 |
| DEDD2 | 0.070593 | -0.40493 | 0.595 | 0.597 | 1 |
| CREBRF | 0.070901 | -0.31704 | 0.304 | 0.352 | 1 |
| PTGER4 | 0.07254 | -0.50126 | 0.744 | 0.742 | 1 |
| PFKFB3 | 0.073052 | -0.48302 | 0.613 | 0.606 | 1 |
| GIMAP5 | 0.07355 | -0.43378 | 0.786 | 0.743 | 1 |
| SYNE1 | 0.076343 | -0.47535 | 0.905 | 0.828 | 1 |
| CTSS | 0.078179 | -0.37902 | 0.75 | 0.706 | 1 |
| CCNH | 0.082414 | -0.42794 | 0.774 | 0.758 | 1 |
| N4BP2L2 | 0.083455 | -0.34461 | 0.899 | 0.857 | 1 |
| PHF1 | 0.084794 | -0.3535 | 0.345 | 0.36 | 1 |
| MYLIP | 0.085614 | -0.31817 | 0.333 | 0.369 | 1 |
| CSRNP1 | 0.087695 | -0.4925 | 0.494 | 0.514 | 1 |
| RGCC | 0.087947 | -0.46171 | 0.762 | 0.721 | 1 |
| KCNA3 | 0.089802 | -0.41029 | 0.375 | 0.398 | 1 |
| LOC100130476 | 0.09455 | -0.37103 | 0.595 | 0.581 | 1 |
| RBM6 | 0.097835 | -0.25616 | 0.935 | 0.902 | 1 |
| VSIR | 0.101771 | -0.43544 | 0.69 | 0.655 | 1 |
| LOC284454 | 0.110716 | -0.40468 | 0.435 | 0.44 | 1 |
| PTGER2 | 0.111688 | -0.38551 | 0.28 | 0.313 | 1 |
| DDX3X | 0.111688 | -0.36831 | 0.988 | 0.953 | 1 |
| PLEK | 0.114974 | -0.32069 | 0.518 | 0.519 | 1 |
| ATP2B1 | 0.118264 | -0.33996 | 0.732 | 0.586 | 1 |
| HEXIM1 | 0.119372 | -0.3053 | 0.375 | 0.407 | 1 |
| GOLGA8B | 0.119845 | -0.33299 | 0.631 | 0.641 | 1 |
| USP34 | 0.122801 | -0.28263 | 0.72 | 0.611 | 1 |
| TAGAP | 0.122979 | -0.38416 | 0.881 | 0.82 | 1 |
| PRMT9 | 0.125815 | -0.31547 | 0.292 | 0.343 | 1 |
| SERTAD1 | 0.129128 | -0.4602 | 0.542 | 0.519 | 1 |
| HSP90AA1 | 0.129631 | -0.35976 | 1 | 0.996 | 1 |
| GLUL | 0.1324 | -0.37984 | 0.44 | 0.49 | 1 |
| HSPA1L | 0.134202 | -0.56558 | 0.738 | 0.587 | 1 |
| HSPA6 | 0.139391 | -0.45756 | 0.298 | 0.241 | 1 |
| WSB1 | 0.141689 | -0.32 | 0.923 | 0.886 | 1 |
| TBCC | 0.14336 | -0.35818 | 0.714 | 0.65 | 1 |
| EVI2B | 0.145773 | -0.3031 | 0.935 | 0.858 | 1 |
| DOCK10 | 0.15162 | -0.35978 | 0.863 | 0.81 | 1 |
| FAM102A | 0.153534 | -0.31946 | 0.619 | 0.617 | 1 |
| PIK3R1 | 0.156631 | -0.57291 | 0.78 | 0.761 | 1 |
| MIAT | 0.157923 | -0.39734 | 0.815 | 0.768 | 1 |
| ATXN7 | 0.170372 | -0.3507 | 0.625 | 0.613 | 1 |
| PPP2R5C | 0.172775 | -0.27764 | 0.958 | 0.911 | 1 |
| KCNQ1OT1 | 0.176207 | -0.26656 | 0.232 | 0.27 | 1 |
| ZNF652 | 0.17676 | -0.27419 | 0.643 | 0.526 | 1 |
| CDC42SE1 | 0.176964 | -0.28905 | 0.952 | 0.951 | 1 |
| HERPUD1 | 0.180276 | -0.36838 | 0.97 | 0.901 | 1 |
| ATRX | 0.182812 | -0.28206 | 0.94 | 0.858 | 1 |
| OFD1 | 0.187216 | -0.36452 | 0.732 | 0.694 | 1 |
| BAZ2B | 0.187522 | -0.2787 | 0.387 | 0.415 | 1 |
| FCMR | 0.190695 | -0.41738 | 0.685 | 0.618 | 1 |
| USP15 | 0.190933 | -0.36037 | 0.863 | 0.804 | 1 |
| DDX27 | 0.191591 | -0.30877 | 0.577 | 0.572 | 1 |
| TSEN54 | 0.193723 | -0.32766 | 0.732 | 0.684 | 1 |
| BBX | 0.194834 | -0.3021 | 0.714 | 0.679 | 1 |
| TNF | 0.195432 | -0.33009 | 0.44 | 0.359 | 1 |
| ZAP70 | 0.198012 | -0.25974 | 0.946 | 0.857 | 1 |
| RNF213 | 0.201248 | -0.25948 | 0.994 | 0.958 | 1 |
| GSPT1 | 0.206759 | -0.26492 | 0.726 | 0.599 | 1 |
| HERC1 | 0.206921 | -0.31545 | 0.869 | 0.815 | 1 |
| GCC2 | 0.207006 | -0.3487 | 0.857 | 0.8 | 1 |
| TTC13 | 0.215216 | -0.2796 | 0.464 | 0.36 | 1 |
| SDCBP | 0.219968 | -0.33778 | 0.899 | 0.865 | 1 |
| RB1CC1 | 0.227038 | -0.26712 | 0.601 | 0.488 | 1 |
| PATJ | 0.228101 | -0.28119 | 0.333 | 0.344 | 1 |
| ZCCHC6 | 0.23065 | -0.25819 | 0.804 | 0.685 | 1 |
| SBDS | 0.237379 | -0.38867 | 0.708 | 0.634 | 1 |
| ZFAND5 | 0.251671 | -0.36864 | 0.756 | 0.725 | 1 |
| BTN3A3 | 0.253704 | -0.26093 | 0.685 | 0.682 | 1 |
| TSC1 | 0.254768 | -0.26392 | 0.405 | 0.336 | 1 |
| BCL6 | 0.257669 | -0.32062 | 0.244 | 0.255 | 1 |
| SNRK | 0.260701 | -0.33338 | 0.512 | 0.499 | 1 |
| APBA2 | 0.260852 | -0.29549 | 0.363 | 0.369 | 1 |
| BIN2 | 0.263075 | -0.28769 | 0.899 | 0.84 | 1 |
| KLHL28 | 0.265196 | -0.28659 | 0.607 | 0.495 | 1 |
| HSPA1B | 0.265492 | -0.29566 | 0.744 | 0.677 | 1 |
| HBP1 | 0.268284 | -0.45405 | 0.661 | 0.639 | 1 |
| RASA3 | 0.27129 | -0.27747 | 0.369 | 0.37 | 1 |
| JMJD6 | 0.273374 | -0.41042 | 0.726 | 0.662 | 1 |
| CCSER2 | 0.283991 | -0.35175 | 0.833 | 0.765 | 1 |
| STK38 | 0.287499 | -0.26797 | 0.536 | 0.453 | 1 |
| ODF2L | 0.294107 | -0.28808 | 0.613 | 0.611 | 1 |
| PPP1R16B | 0.294287 | -0.28061 | 0.726 | 0.708 | 1 |
| AMD1 | 0.297859 | -0.38409 | 0.673 | 0.656 | 1 |
| STK17A | 0.299249 | -0.29342 | 0.899 | 0.855 | 1 |
| SAMHD1 | 0.302349 | -0.29818 | 0.696 | 0.574 | 1 |
| DCTN6 | 0.309335 | -0.28667 | 0.655 | 0.557 | 1 |
| ZFP36L1 | 0.315243 | -0.26639 | 0.994 | 0.916 | 1 |
| CHD9 | 0.320931 | -0.33801 | 0.631 | 0.54 | 1 |
| LOC643733 | 0.326873 | -0.26645 | 0.399 | 0.4 | 1 |
| UBE2D1 | 0.328592 | -0.39533 | 0.381 | 0.385 | 1 |
| SEC24B | 0.338356 | -0.28726 | 0.53 | 0.455 | 1 |
| ZNF394 | 0.340915 | -0.34935 | 0.446 | 0.438 | 1 |
| RIPOR2 | 0.355567 | -0.5609 | 0.78 | 0.688 | 1 |
| GAS5 | 0.358713 | -0.25331 | 0.786 | 0.777 | 1 |
| SRSF6 | 0.362277 | -0.44451 | 0.899 | 0.866 | 1 |
| ATM | 0.363924 | -0.59969 | 0.756 | 0.671 | 1 |
| GLG1 | 0.370144 | -0.2751 | 0.893 | 0.825 | 1 |
| TBRG1 | 0.370613 | -0.25093 | 0.762 | 0.702 | 1 |
| CMTM7 | 0.372535 | -0.25364 | 0.649 | 0.535 | 1 |
| PTPN4 | 0.375186 | -0.26164 | 0.839 | 0.737 | 1 |
| RELL1 | 0.380891 | -0.28212 | 0.411 | 0.437 | 1 |
| CYTH1 | 0.386629 | -0.2864 | 0.708 | 0.673 | 1 |
| TMC8 | 0.387179 | -0.29529 | 0.821 | 0.767 | 1 |
| IFRD1 | 0.38797 | -0.49555 | 0.744 | 0.667 | 1 |
| DENND2D | 0.409963 | -0.29755 | 0.78 | 0.731 | 1 |
| FAM53C | 0.41339 | -0.3187 | 0.399 | 0.418 | 1 |
| RPL17-C18orf32 | 0.414705 | -0.25687 | 0.988 | 0.973 | 1 |
| BIRC2 | 0.417016 | -0.27848 | 0.72 | 0.627 | 1 |
| NCK1 | 0.431203 | -0.31744 | 0.542 | 0.461 | 1 |
| ZNF721 | 0.440937 | -0.28604 | 0.518 | 0.461 | 1 |
| HSPA8 | 0.441157 | -0.26681 | 1 | 0.999 | 1 |
| CDC14A | 0.442118 | -0.37355 | 0.649 | 0.601 | 1 |
| EEF2 | 0.446123 | -0.25694 | 0.786 | 0.735 | 1 |
| MAGED2 | 0.452421 | -0.3135 | 0.649 | 0.595 | 1 |
| SYTL2 | 0.456115 | -0.40197 | 0.768 | 0.684 | 1 |
| BTN3A2 | 0.458013 | -0.26386 | 0.875 | 0.8 | 1 |
| SC5D | 0.466923 | -0.30858 | 0.381 | 0.388 | 1 |
| GPCPD1 | 0.467839 | -0.293 | 0.655 | 0.641 | 1 |
| GPR171 | 0.483148 | -0.28528 | 0.857 | 0.804 | 1 |
| MRPL10 | 0.483164 | -0.2851 | 0.601 | 0.591 | 1 |
| SYNRG | 0.488158 | -0.28844 | 0.798 | 0.731 | 1 |
| RASGEF1B | 0.493732 | -0.2522 | 0.47 | 0.405 | 1 |
| TMEM43 | 0.495977 | -0.27848 | 0.685 | 0.635 | 1 |
| RBM4 | 0.502238 | -0.28299 | 0.869 | 0.803 | 1 |
| LTB | 0.508752 | -0.26654 | 0.655 | 0.543 | 1 |
| DOK2 | 0.512857 | -0.36264 | 0.851 | 0.746 | 1 |
| PARP15 | 0.513722 | -0.2816 | 0.53 | 0.47 | 1 |
| TTC39B | 0.514104 | -0.26063 | 0.28 | 0.283 | 1 |
| PDE4D | 0.515448 | -0.39106 | 0.821 | 0.683 | 1 |
| EVL | 0.523704 | -0.25737 | 0.982 | 0.933 | 1 |
| RNF166 | 0.525784 | -0.37095 | 0.679 | 0.614 | 1 |
| ICAM2 | 0.527318 | -0.27783 | 0.28 | 0.278 | 1 |
| AKIRIN1 | 0.528557 | -0.31343 | 0.714 | 0.667 | 1 |
| SNHG1 | 0.542346 | -0.40425 | 0.435 | 0.437 | 1 |
| DENND4A | 0.546169 | -0.32786 | 0.571 | 0.583 | 1 |
| TARP | 0.546451 | -0.30189 | 0.917 | 0.85 | 1 |
| DYNC1H1 | 0.548804 | -0.27243 | 0.952 | 0.842 | 1 |
| GBP5 | 0.553353 | -0.32458 | 0.952 | 0.892 | 1 |
| BSDC1 | 0.554786 | -0.26149 | 0.488 | 0.419 | 1 |
| ZBTB1 | 0.563233 | -0.27511 | 0.804 | 0.727 | 1 |
| KIAA1109 | 0.563346 | -0.32078 | 0.673 | 0.593 | 1 |
| XRN1 | 0.574479 | -0.2506 | 0.881 | 0.756 | 1 |
| TRAM1 | 0.581604 | -0.26637 | 0.917 | 0.843 | 1 |
| KAT6A | 0.581646 | -0.26207 | 0.637 | 0.564 | 1 |
| GATA3 | 0.583828 | -0.30349 | 0.625 | 0.601 | 1 |
| EP300 | 0.591456 | -0.33568 | 0.607 | 0.541 | 1 |
| CHD1 | 0.599813 | -0.30997 | 0.869 | 0.786 | 1 |
| GIMAP4 | 0.601409 | -0.32448 | 0.923 | 0.819 | 1 |
| PREX1 | 0.608778 | -0.25871 | 0.81 | 0.673 | 1 |
| TNFSF8 | 0.610354 | -0.34338 | 0.44 | 0.418 | 1 |
| CDC42EP3 | 0.622836 | -0.33687 | 0.536 | 0.519 | 1 |
| BRWD1 | 0.623484 | -0.27568 | 0.714 | 0.662 | 1 |
| MAP3K8 | 0.631439 | -0.42538 | 0.482 | 0.467 | 1 |
| WBP2 | 0.640305 | -0.27348 | 0.774 | 0.697 | 1 |
| ANKZF1 | 0.648662 | -0.26423 | 0.488 | 0.482 | 1 |
| PRKACB | 0.651935 | -0.29727 | 0.899 | 0.794 | 1 |
| TNFRSF14 | 0.657738 | -0.27353 | 0.625 | 0.536 | 1 |
| UBE2B | 0.658331 | -0.28147 | 0.798 | 0.741 | 1 |
| RAB9A | 0.665224 | -0.26591 | 0.339 | 0.34 | 1 |
| GLIPR1 | 0.666179 | -0.31102 | 0.929 | 0.846 | 1 |
| SMCHD1 | 0.676602 | -0.25138 | 0.94 | 0.888 | 1 |
| RNF216 | 0.676948 | -0.25786 | 0.69 | 0.622 | 1 |
| HSPH1 | 0.680387 | -0.56133 | 0.875 | 0.779 | 1 |
| PRDM2 | 0.695938 | -0.28505 | 0.702 | 0.671 | 1 |
| SPSB3 | 0.708771 | -0.254 | 0.696 | 0.588 | 1 |
| STAT6 | 0.709306 | -0.32227 | 0.488 | 0.447 | 1 |
| CD28 | 0.710693 | -0.31502 | 0.542 | 0.451 | 1 |
| NR4A1 | 0.720039 | -0.4305 | 0.476 | 0.47 | 1 |
| DDX24 | 0.726716 | -0.26175 | 0.946 | 0.904 | 1 |
| SETD2 | 0.728828 | -0.28936 | 0.714 | 0.627 | 1 |
| FAM177A1 | 0.732736 | -0.31264 | 0.661 | 0.596 | 1 |
| NFKBIZ | 0.734861 | -0.41992 | 0.696 | 0.635 | 1 |
| IKZF5 | 0.741018 | -0.27291 | 0.494 | 0.436 | 1 |
| AKAP9 | 0.748914 | -0.3175 | 0.893 | 0.817 | 1 |
| SERINC3 | 0.760067 | -0.26981 | 0.857 | 0.765 | 1 |
| TMC6 | 0.761736 | -0.28335 | 0.911 | 0.842 | 1 |
| CHMP1B | 0.763743 | -0.29922 | 0.69 | 0.632 | 1 |
| IL16 | 0.763976 | -0.2583 | 0.863 | 0.75 | 1 |
| PPM1K | 0.770298 | -0.34289 | 0.643 | 0.558 | 1 |
| ANKRD37 | 0.773709 | -0.2651 | 0.375 | 0.354 | 1 |
| UAP1 | 0.778444 | -0.31119 | 0.345 | 0.343 | 1 |
| MAFF | 0.778707 | -0.29779 | 0.351 | 0.329 | 1 |
| EPB41 | 0.787103 | -0.31759 | 0.649 | 0.553 | 1 |
| SSH2 | 0.788113 | -0.3295 | 0.649 | 0.543 | 1 |
| STK10 | 0.789096 | -0.25652 | 0.827 | 0.7 | 1 |
| NLRP1 | 0.794046 | -0.3571 | 0.72 | 0.642 | 1 |
| RHOF | 0.79489 | -0.25571 | 0.845 | 0.754 | 1 |
| ITPRIP | 0.795055 | -0.33583 | 0.47 | 0.45 | 1 |
| TUBA4A | 0.798666 | -0.32041 | 0.952 | 0.904 | 1 |
| SAR1A | 0.805992 | -0.32007 | 0.958 | 0.855 | 1 |
| PPP1R10 | 0.80982 | -0.27428 | 0.613 | 0.551 | 1 |
| KDM2A | 0.81151 | -0.25903 | 0.714 | 0.654 | 1 |
| ARID5B | 0.814735 | -0.42834 | 0.899 | 0.777 | 1 |
| NEAT1 | 0.814815 | -0.3485 | 0.982 | 0.915 | 1 |
| KDM5B | 0.819732 | -0.26049 | 0.542 | 0.487 | 1 |
| ARHGAP25 | 0.824748 | -0.27507 | 0.78 | 0.706 | 1 |
| PXN | 0.825001 | -0.25826 | 0.405 | 0.345 | 1 |
| FCHSD1 | 0.82587 | -0.28073 | 0.476 | 0.428 | 1 |
| RANBP2 | 0.826914 | -0.31186 | 0.833 | 0.723 | 1 |
| GIMAP1 | 0.831168 | -0.27437 | 0.452 | 0.416 | 1 |
| NXF1 | 0.832015 | -0.39117 | 0.738 | 0.689 | 1 |
| PDE7A | 0.83346 | -0.25398 | 0.649 | 0.572 | 1 |
| B4GALT3 | 0.834218 | -0.25481 | 0.435 | 0.39 | 1 |
| CLDND1 | 0.837669 | -0.35354 | 0.869 | 0.781 | 1 |
| ATP8A1 | 0.837752 | -0.28376 | 0.738 | 0.65 | 1 |
| TOB2 | 0.840866 | -0.32282 | 0.649 | 0.558 | 1 |
| VPS37B | 0.858742 | -0.25955 | 0.589 | 0.507 | 1 |
| SNHG12 | 0.877992 | -0.2551 | 0.637 | 0.591 | 1 |
| RHOH | 0.881537 | -0.25269 | 0.976 | 0.89 | 1 |
| P4HTM | 0.88598 | -0.2968 | 0.357 | 0.318 | 1 |
| DHX36 | 0.892665 | -0.26381 | 0.673 | 0.629 | 1 |
| ZEB2 | 0.892747 | -0.34578 | 0.827 | 0.742 | 1 |
| CCNL2 | 0.913695 | -0.3015 | 0.786 | 0.696 | 1 |
| NFKBIA | 0.929898 | -0.28169 | 0.964 | 0.934 | 1 |
| GPR65 | 0.932395 | -0.42015 | 0.744 | 0.679 | 1 |
| DNAJB4 | 0.933502 | -0.27987 | 0.298 | 0.29 | 1 |
| 6-Mar | 0.936366 | -0.27231 | 0.929 | 0.857 | 1 |
| TUBA1A | 0.943137 | -0.33218 | 0.899 | 0.802 | 1 |
| NAA50 | 0.943275 | -0.27344 | 0.827 | 0.788 | 1 |
| KLHL24 | 0.955484 | -0.26862 | 0.47 | 0.428 | 1 |
| EVI2A | 0.969062 | -0.28023 | 0.768 | 0.698 | 1 |
