# Supplementary material for: Dynamic Network Biomarker of Pre-Exhausted CD8+ T Cells Contributed to T Cell Exhaustion in Colorectal Cancer
Source: Front Immunol. 2021 Aug 9;12:691142. doi: 10.3389/fimmu.2021.691142 (PMC8381053; doi:10.3389/fimmu.2021.691142)
Supplement: Supplementary file 6 [file Table_3.docx]

| SUID | degree.layout | name | selected | shared name |
| --- | --- | --- | --- | --- |
| 3922 | 83 | HDAC1 | FALSE | "HDAC1" |
| 3923 | 1 | MAML1 | FALSE | "MAML1" |
| 3925 | 78 | PPP2R1A | FALSE | "PPP2R1A" |
| 3926 | 5 | RPL30 | FALSE | "RPL30" |
| 3928 | 65 | JUN | FALSE | "JUN" |
| 3929 | 3 | SPI1 | FALSE | "SPI1" |
| 3931 | 20 | HNRNPM | FALSE | "HNRNPM" |
| 3932 | 2 | WDR77 | FALSE | "WDR77" |
| 3934 | 1 | RBPJ | FALSE | "RBPJ" |
| 3936 | 13 | SRSF1 | FALSE | "SRSF1" |
| 3937 | 7 | TXNL4A | FALSE | "TXNL4A" |
| 3939 | 12 | PSMC2 | FALSE | "PSMC2" |
| 3940 | 5 | PSMD10 | FALSE | "PSMD10" |
| 3942 | 6 | SNORD61 | FALSE | "SNORD61" |
| 3944 | 50 | EIF3E | FALSE | "EIF3E" |
| 3945 | 5 | RPL24 | FALSE | "RPL24" |
| 3947 | 31 | POLR2K | FALSE | "POLR2K" |
| 3948 | 2 | URI1 | FALSE | "URI1" |
| 3950 | 33 | HIF1A | FALSE | "HIF1A" |
| 3951 | 4 | SNORD96A | FALSE | "SNORD96A" |
| 3953 | 79 | FOS | FALSE | "FOS" |
| 3954 | 3 | SIRT6 | FALSE | "SIRT6" |
| 3956 | 13 | PSMD14 | FALSE | "PSMD14" |
| 3957 | 8 | PSME1 | FALSE | "PSME1" |
| 3959 | 1 | TNFRSF11A | FALSE | "TNFRSF11A" |
| 3961 | 27 | RPN1 | FALSE | "RPN1" |
| 3962 | 4 | UBXN7 | FALSE | "UBXN7" |
| 3964 | 31 | CASP3 | FALSE | "CASP3" |
| 3965 | 1 | GAS2 | FALSE | "GAS2" |
| 3967 | 3 | NFATC3 | FALSE | "NFATC3" |
| 3969 | 25 | CCT4 | FALSE | "CCT4" |
| 3970 | 2 | PDCL2 | FALSE | "PDCL2" |
| 3972 | 51 | EIF3A | FALSE | "EIF3A" |
| 3973 | 6 | RPS24 | FALSE | "RPS24" |
| 3975 | 49 | ETF1 | FALSE | "ETF1" |
| 3976 | 3 | SMG9 | FALSE | "SMG9" |
| 3978 | 7 | RPS4X | FALSE | "RPS4X" |
| 3980 | 1 | RUNX1T1 | FALSE | "RUNX1T1" |
| 3982 | 17 | CANX | FALSE | "CANX" |
| 3983 | 6 | SSR1 | FALSE | "SSR1" |
| 3985 | 1 | ITGA1 | FALSE | "ITGA1" |
| 3987 | 4 | SRSF6 | FALSE | "SRSF6" |
| 3989 | 1 | TNPO1 | FALSE | "TNPO1" |
| 3991 | 1 | PCK1 | FALSE | "PCK1" |
| 3993 | 63 | EFTUD2 | FALSE | "EFTUD2" |
| 3994 | 5 | SNRPD2 | FALSE | "SNRPD2" |
| 3996 | 2 | RAC3 | FALSE | "RAC3" |
| 3998 | 62 | EIF4A3 | FALSE | "EIF4A3" |
| 3999 | 5 | RPL35A | FALSE | "RPL35A" |
| 4001 | 8 | RPS15A | FALSE | "RPS15A" |
| 4003 | 3 | UPF3A | FALSE | "UPF3A" |
| 4006 | 1 | RACGAP1 | FALSE | "RACGAP1" |
| 4008 | 17 | NFKBIA | FALSE | "NFKBIA" |
| 4009 | 2 | PIK3R1 | FALSE | "PIK3R1" |
| 4011 | 29 | MDM2 | FALSE | "MDM2" |
| 4012 | 9 | RPS14 | FALSE | "RPS14" |
| 4014 | 5 | SNRPB | FALSE | "SNRPB" |
| 4016 | 1 | HIST1H2BO | FALSE | "HIST1H2BO" |
| 4018 | 2 | MYB | FALSE | "MYB" |
| 4020 | 22 | IFNG | FALSE | "IFNG" |
| 4021 | 1 | MAP3K11 | FALSE | "MAP3K11" |
| 4023 | 7 | RPL7 | FALSE | "RPL7" |
| 4025 | 21 | CCT7 | FALSE | "CCT7" |
| 4026 | 2 | PAN2 | FALSE | "PAN2" |
| 4028 | 1 | PARN | FALSE | "PARN" |
| 4030 | 2 | EIF5 | FALSE | "EIF5" |
| 4032 | 12 | SF3A3 | FALSE | "SF3A3" |
| 4033 | 5 | SNRPB2 | FALSE | "SNRPB2" |
| 4036 | 5 | RPL41 | FALSE | "RPL41" |
| 4038 | 1 | CDC42 | FALSE | "CDC42" |
| 4041 | 12 | PSMC3 | FALSE | "PSMC3" |
| 4042 | 1 | SNCA | FALSE | "SNCA" |
| 4044 | 7 | RPS6 | FALSE | "RPS6" |
| 4046 | 1 | USP7 | FALSE | "USP7" |
| 4048 | 48 | POLR2B | FALSE | "POLR2B" |
| 4049 | 3 | TCEA2 | FALSE | "TCEA2" |
| 4052 | 2 | SENP1 | FALSE | "SENP1" |
| 4055 | 2 | TP63 | FALSE | "TP63" |
| 4057 | 3 | USP10 | FALSE | "USP10" |
| 4059 | 1 | PPP2R5E | FALSE | "PPP2R5E" |
| 4061 | 1 | ZGPAT | FALSE | "ZGPAT" |
| 4063 | 8 | RPL26 | FALSE | "RPL26" |
| 4065 | 3 | PIK3CA | FALSE | "PIK3CA" |
| 4067 | 1 | PIAS4 | FALSE | "PIAS4" |
| 4070 | 6 | MIR3652 | FALSE | "MIR3652" |
| 4072 | 1 | KLF13 | FALSE | "KLF13" |
| 4074 | 6 | RPL18 | FALSE | "RPL18" |
| 4076 | 2 | QARS | FALSE | "QARS" |
| 4080 | 3 | PPARGC1A | FALSE | "PPARGC1A" |
| 4082 | 8 | RPS15 | FALSE | "RPS15" |
| 4084 | 1 | UHRF1BP1 | FALSE | "UHRF1BP1" |
| 4086 | 10 | STAT1 | FALSE | "STAT1" |
| 4087 | 2 | ZNF148 | FALSE | "ZNF148" |
| 4089 | 2 | RAC2 | FALSE | "RAC2" |
| 4091 | 26 | CCT5 | FALSE | "CCT5" |
| 4092 | 5 | HSPA7 | FALSE | "HSPA7" |
| 4094 | 1 | GTF2I | FALSE | "GTF2I" |
| 4096 | 1 | PPP2R1B | FALSE | "PPP2R1B" |
| 4099 | 10 | RPS3 | FALSE | "RPS3" |
| 4101 | 6 | SF3B1 | FALSE | "SF3B1" |
| 4103 | 22 | CCT3 | FALSE | "CCT3" |
| 4104 | 6 | MKKS | FALSE | "MKKS" |
| 4106 | 6 | RPA1 | FALSE | "RPA1" |
| 4108 | 1 | TNS1 | FALSE | "TNS1" |
| 4110 | 1 | SAP30 | FALSE | "SAP30" |
| 4112 | 4 | SEC61A1 | FALSE | "SEC61A1" |
| 4113 | 2 | SSR2 | FALSE | "SSR2" |
| 4115 | 8 | RPS29 | FALSE | "RPS29" |
| 4117 | 1 | PPM1J | FALSE | "PPM1J" |
| 4119 | 3 | PRKCB | FALSE | "PRKCB" |
| 4121 | 8 | RPS23 | FALSE | "RPS23" |
| 4123 | 7 | RILP | FALSE | "RILP" |
| 4125 | 2 | RPS18 | FALSE | "RPS18" |
| 4127 | 15 | BIRC2 | FALSE | "BIRC2" |
| 4128 | 1 | BIRC7 | FALSE | "BIRC7" |
| 4130 | 1 | MRPS6 | FALSE | "MRPS6" |
| 4132 | 3 | TCF7L2 | FALSE | "TCF7L2" |
| 4134 | 1 | RAD54L | FALSE | "RAD54L" |
| 4137 | 24 | MCM7 | FALSE | "MCM7" |
| 4138 | 2 | RFC5 | FALSE | "RFC5" |
| 4140 | 3 | RIPK1 | FALSE | "RIPK1" |
| 4142 | 9 | UBE2E1 | FALSE | "UBE2E1" |
| 4144 | 1 | PAX5 | FALSE | "PAX5" |
| 4146 | 9 | TUBA1B | FALSE | "TUBA1B" |
| 4148 | 7 | RPL3L | FALSE | "RPL3L" |
| 4151 | 6 | RB1 | FALSE | "RB1" |
| 4154 | 1 | TAX1BP1 | FALSE | "TAX1BP1" |
| 4156 | 10 | TUBB4B | FALSE | "TUBB4B" |
| 4158 | 2 | SNAPC4 | FALSE | "SNAPC4" |
| 4160 | 9 | TUBB4A | FALSE | "TUBB4A" |
| 4163 | 1 | ORC1 | FALSE | "ORC1" |
| 4165 | 2 | IL2RA | FALSE | "IL2RA" |
| 4168 | 5 | RPL36 | FALSE | "RPL36" |
| 4170 | 1 | MCMDC2 | FALSE | "MCMDC2" |
| 4172 | 1 | IL9R | FALSE | "IL9R" |
| 4174 | 3 | NCOR2 | FALSE | "NCOR2" |
| 4177 | 5 | RPL14 | FALSE | "RPL14" |
| 4179 | 1 | DSG2 | FALSE | "DSG2" |
| 4181 | 1 | SGO1 | FALSE | "SGO1" |
| 4183 | 10 | TP53 | FALSE | "TP53" |
| 4185 | 7 | PFDN5 | FALSE | "PFDN5" |
| 4188 | 5 | FAU | FALSE | "FAU" |
| 4190 | 1 | DUOX2 | FALSE | "DUOX2" |
| 4192 | 1 | LAMTOR5 | FALSE | "LAMTOR5" |
| 4194 | 3 | PRDM1 | FALSE | "PRDM1" |
| 4196 | 1 | SRP68 | FALSE | "SRP68" |
| 4198 | 1 | HNRNPL | FALSE | "HNRNPL" |
| 4200 | 11 | PSMD11 | FALSE | "PSMD11" |
| 4201 | 8 | SEM1 | FALSE | "SEM1" |
| 4203 | 1 | NMU | FALSE | "NMU" |
| 4205 | 1 | RPRD2 | FALSE | "RPRD2" |
| 4207 | 3 | VDR | FALSE | "VDR" |
| 4209 | 7 | RPL27A | FALSE | "RPL27A" |
| 4211 | 6 | RPS21 | FALSE | "RPS21" |
| 4217 | 2 | USP20 | FALSE | "USP20" |
| 4220 | 2 | POLR3F | FALSE | "POLR3F" |
| 4222 | 3 | PRPF19 | FALSE | "PRPF19" |
| 4224 | 8 | TUBA1C | FALSE | "TUBA1C" |
| 4226 | 6 | RPS10-NUDT3 | FALSE | "RPS10-NUDT3" |
| 4228 | 2 | CASP9 | FALSE | "CASP9" |
| 4231 | 13 | PSMA4 | FALSE | "PSMA4" |
| 4232 | 12 | RLIM | FALSE | "RLIM" |
| 4234 | 7 | RPS13 | FALSE | "RPS13" |
| 4236 | 2 | PCBP2 | FALSE | "PCBP2" |
| 4238 | 1 | MRTO4 | FALSE | "MRTO4" |
| 4242 | 2 | MAPK12 | FALSE | "MAPK12" |
| 4244 | 1 | VLDLR | FALSE | "VLDLR" |
| 4246 | 3 | PCNA | FALSE | "PCNA" |
| 4248 | 5 | TERT | FALSE | "TERT" |
| 4251 | 10 | CCT8 | FALSE | "CCT8" |
| 4252 | 8 | STRN3 | FALSE | "STRN3" |
| 4255 | 8 | RPL11 | FALSE | "RPL11" |
| 4257 | 9 | HSP90AA1 | FALSE | "HSP90AA1" |
| 4259 | 1 | PLK1 | FALSE | "PLK1" |
| 4261 | 1 | KLF1 | FALSE | "KLF1" |
| 4265 | 1 | PIAS2 | FALSE | "PIAS2" |
| 4269 | 11 | CCT6A | FALSE | "CCT6A" |
| 4271 | 5 | RPL22 | FALSE | "RPL22" |
| 4273 | 2 | CFLAR | FALSE | "CFLAR" |
| 4276 | 5 | RAD23A | FALSE | "RAD23A" |
| 4278 | 7 | TCP1 | FALSE | "TCP1" |
| 4280 | 4 | SYK | FALSE | "SYK" |
| 4282 | 1 | SIKE1 | FALSE | "SIKE1" |
| 4284 | 1 | FOXO3 | FALSE | "FOXO3" |
| 4286 | 5 | RPL37 | FALSE | "RPL37" |
| 4288 | 1 | CDKN1A | FALSE | "CDKN1A" |
| 4290 | 3 | RPS6KB1 | FALSE | "RPS6KB1" |
| 4292 | 2 | JAK1 | FALSE | "JAK1" |
| 4294 | 1 | SMARCD1 | FALSE | "SMARCD1" |
| 4296 | 6 | SRSF11 | FALSE | "SRSF11" |
| 4299 | 1 | IMP3 | FALSE | "IMP3" |
| 4301 | 2 | SRRM2 | FALSE | "SRRM2" |
| 4304 | 8 | SNORA62 | FALSE | "SNORA62" |
| 4309 | 2 | WWTR1 | FALSE | "WWTR1" |
| 4312 | 7 | RPL35 | FALSE | "RPL35" |
| 4315 | 1 | TLE4 | FALSE | "TLE4" |
| 4318 | 2 | PRKCG | FALSE | "PRKCG" |
| 4320 | 5 | HSPA12A | FALSE | "HSPA12A" |
| 4322 | 1 | HIST1H2AA | FALSE | "HIST1H2AA" |
| 4325 | 1 | SMARCA1 | FALSE | "SMARCA1" |
| 4327 | 4 | RBM5 | FALSE | "RBM5" |
| 4329 | 8 | RBX1 | FALSE | "RBX1" |
| 4331 | 1 | TNC | FALSE | "TNC" |
| 4333 | 1 | PPP3CA | FALSE | "PPP3CA" |
| 4335 | 1 | MRPL11 | FALSE | "MRPL11" |
| 4337 | 5 | FARS2 | FALSE | "FARS2" |
| 4341 | 2 | YAP1 | FALSE | "YAP1" |
| 4344 | 1 | POLA1 | FALSE | "POLA1" |
| 4346 | 1 | XBP1 | FALSE | "XBP1" |
| 4350 | 1 | TLE1 | FALSE | "TLE1" |
| 4356 | 1 | MYD88 | FALSE | "MYD88" |
| 4358 | 2 | STAT4 | FALSE | "STAT4" |
| 4362 | 5 | HSPA1L | FALSE | "HSPA1L" |
| 4365 | 6 | ILK | FALSE | "ILK" |
| 4367 | 8 | PPP4C | FALSE | "PPP4C" |
| 4369 | 2 | PRKACA | FALSE | "PRKACA" |
| 4371 | 10 | PSMD6 | FALSE | "PSMD6" |
| 4372 | 8 | UBE2C | FALSE | "UBE2C" |
| 4374 | 1 | TRPC6 | FALSE | "TRPC6" |
| 4378 | 1 | SLMAP | FALSE | "SLMAP" |
| 4380 | 1 | IL1RL1 | FALSE | "IL1RL1" |
| 4382 | 7 | RPS7 | FALSE | "RPS7" |
| 4387 | 7 | RPL23 | FALSE | "RPL23" |
| 4392 | 22 | CCT2 | FALSE | "CCT2" |
| 4393 | 5 | GRPEL2 | FALSE | "GRPEL2" |
| 4396 | 1 | ZNRD1 | FALSE | "ZNRD1" |
| 4399 | 1 | HTR3A | FALSE | "HTR3A" |
| 4401 | 1 | CD74 | FALSE | "CD74" |
| 4404 | 14 | PSMC5 | FALSE | "PSMC5" |
| 4406 | 1 | TOP2B | FALSE | "TOP2B" |
| 4408 | 1 | TGFB1I1 | FALSE | "TGFB1I1" |
| 4411 | 1 | LDB1 | FALSE | "LDB1" |
| 4413 | 1 | RLN3 | FALSE | "RLN3" |
| 4418 | 1 | PGM1 | FALSE | "PGM1" |
| 4422 | 1 | PRKD1 | FALSE | "PRKD1" |
| 4424 | 1 | SUV39H1 | FALSE | "SUV39H1" |
| 4426 | 4 | SSRP1 | FALSE | "SSRP1" |
| 4428 | 2 | MYBL2 | FALSE | "MYBL2" |
| 4430 | 1 | HSF4 | FALSE | "HSF4" |
| 4432 | 5 | SF3A2 | FALSE | "SF3A2" |
| 4435 | 3 | SF1 | FALSE | "SF1" |
| 4437 | 5 | TRAF3IP3 | FALSE | "TRAF3IP3" |
| 4439 | 3 | SUPT16H | FALSE | "SUPT16H" |
| 4441 | 8 | RPS12 | FALSE | "RPS12" |
| 4443 | 2 | EIF3J | FALSE | "EIF3J" |
| 4445 | 5 | RPL34 | FALSE | "RPL34" |
| 4448 | 1 | ZBP1 | FALSE | "ZBP1" |
| 4451 | 1 | POLE2 | FALSE | "POLE2" |
| 4453 | 1 | IL23R | FALSE | "IL23R" |
| 4455 | 2 | TLR3 | FALSE | "TLR3" |
| 4460 | 1 | GAPDH | FALSE | "GAPDH" |
| 4465 | 1 | SIVA1 | FALSE | "SIVA1" |
| 4467 | 2 | MDK | FALSE | "MDK" |
| 4469 | 2 | TUSC3 | FALSE | "TUSC3" |
| 4473 | 4 | NCBP1 | FALSE | "NCBP1" |
| 4475 | 1 | HCLS1 | FALSE | "HCLS1" |
| 4477 | 5 | RPL32 | FALSE | "RPL32" |
| 4480 | 3 | SDC2 | FALSE | "SDC2" |
| 4486 | 1 | MOGS | FALSE | "MOGS" |
| 4489 | 1 | LIFR | FALSE | "LIFR" |
| 4493 | 1 | DFFB | FALSE | "DFFB" |
| 4495 | 3 | RAD1 | FALSE | "RAD1" |
| 4498 | 1 | GANAB | FALSE | "GANAB" |
| 4500 | 5 | RPL37A | FALSE | "RPL37A" |
| 4506 | 1 | ROCK1 | FALSE | "ROCK1" |
| 4509 | 5 | RPL27 | FALSE | "RPL27" |
| 4512 | 1 | STRIP2 | FALSE | "STRIP2" |
| 4516 | 1 | GRIPAP1 | FALSE | "GRIPAP1" |
| 4518 | 1 | UCP1 | FALSE | "UCP1" |
| 4521 | 1 | RPS6KA5 | FALSE | "RPS6KA5" |
| 4523 | 1 | PRKCQ | FALSE | "PRKCQ" |
| 4526 | 2 | EIF3K | FALSE | "EIF3K" |
| 4528 | 5 | RPL39 | FALSE | "RPL39" |
| 4530 | 2 | SMARCC1 | FALSE | "SMARCC1" |
| 4534 | 1 | YBX3 | FALSE | "YBX3" |
| 4536 | 5 | RPL28 | FALSE | "RPL28" |
| 4538 | 4 | TOP1 | FALSE | "TOP1" |
| 4540 | 1 | PTGDS | FALSE | "PTGDS" |
| 4542 | 8 | TUBB6 | FALSE | "TUBB6" |
| 4544 | 2 | TNPO3 | FALSE | "TNPO3" |
| 4547 | 7 | RPL12 | FALSE | "RPL12" |
| 4549 | 1 | SNAPC3 | FALSE | "SNAPC3" |
| 4554 | 2 | HRH1 | FALSE | "HRH1" |
| 4559 | 1 | SPARC | FALSE | "SPARC" |
| 4562 | 1 | OSGEP | FALSE | "OSGEP" |
| 4564 | 3 | STAR | FALSE | "STAR" |
| 4569 | 3 | PRKCD | FALSE | "PRKCD" |
| 4573 | 2 | ID1 | FALSE | "ID1" |
| 4578 | 2 | TAF4B | FALSE | "TAF4B" |
| 4580 | 1 | RNF14 | FALSE | "RNF14" |
| 4582 | 3 | EIF4B | FALSE | "EIF4B" |
| 4584 | 1 | ISG15 | FALSE | "ISG15" |
| 4589 | 1 | RASSF5 | FALSE | "RASSF5" |
| 4594 | 7 | PSMD8 | FALSE | "PSMD8" |
| 4597 | 1 | PRLR | FALSE | "PRLR" |
| 4599 | 1 | SEL1L2 | FALSE | "SEL1L2" |
| 4603 | 1 | POLA2 | FALSE | "POLA2" |
| 4605 | 5 | SNRPA | FALSE | "SNRPA" |
| 4610 | 4 | SMAD3 | FALSE | "SMAD3" |
| 4614 | 9 | RPS5 | FALSE | "RPS5" |
| 4618 | 2 | PSMB5 | FALSE | "PSMB5" |
| 4623 | 1 | KDM1A | FALSE | "KDM1A" |
| 4626 | 1 | EIF4E3 | FALSE | "EIF4E3" |
| 4628 | 3 | RUNX2 | FALSE | "RUNX2" |
| 4640 | 7 | UBLCP1 | FALSE | "UBLCP1" |
| 4642 | 1 | RNF31 | FALSE | "RNF31" |
| 4644 | 1 | UCN | FALSE | "UCN" |
| 4646 | 2 | PDF | FALSE | "PDF" |
| 4648 | 1 | SPTA1 | FALSE | "SPTA1" |
| 4652 | 3 | TRADD | FALSE | "TRADD" |
| 4656 | 2 | MAPK10 | FALSE | "MAPK10" |
| 4659 | 5 | HSPA2 | FALSE | "HSPA2" |
| 4664 | 3 | HSPB1 | FALSE | "HSPB1" |
| 4666 | 2 | RFC4 | FALSE | "RFC4" |
| 4676 | 1 | TREX1 | FALSE | "TREX1" |
| 4679 | 1 | RUVBL1 | FALSE | "RUVBL1" |
| 4682 | 2 | SMARCC2 | FALSE | "SMARCC2" |
| 4688 | 1 | MNAT1 | FALSE | "MNAT1" |
| 4693 | 1 | POLR3G | FALSE | "POLR3G" |
| 4696 | 1 | RALGDS | FALSE | "RALGDS" |
| 4699 | 1 | PRIM1 | FALSE | "PRIM1" |
| 4701 | 1 | SSU72 | FALSE | "SSU72" |
| 4706 | 4 | PRKCA | FALSE | "PRKCA" |
| 4708 | 1 | TNNC1 | FALSE | "TNNC1" |
| 4713 | 1 | RWDD3 | FALSE | "RWDD3" |
| 4717 | 1 | HARS2 | FALSE | "HARS2" |
| 4719 | 1 | HK3 | FALSE | "HK3" |
| 4722 | 3 | MET | FALSE | "MET" |
| 4724 | 1 | KRIT1 | FALSE | "KRIT1" |
| 4727 | 1 | ZMAT2 | FALSE | "ZMAT2" |
| 4733 | 4 | GMPS | FALSE | "GMPS" |
| 4736 | 1 | ZC3H13 | FALSE | "ZC3H13" |
| 4741 | 1 | GRM5 | FALSE | "GRM5" |
| 4743 | 1 | IRF9 | FALSE | "IRF9" |
| 4746 | 1 | GSPT2 | FALSE | "GSPT2" |
| 4750 | 1 | SFSWAP | FALSE | "SFSWAP" |
| 4752 | 1 | KAT8 | FALSE | "KAT8" |
| 4759 | 1 | HIST1H2AE | FALSE | "HIST1H2AE" |
| 4762 | 2 | RFC3 | FALSE | "RFC3" |
| 4764 | 1 | SVIP | FALSE | "SVIP" |
| 4766 | 2 | TJP2 | FALSE | "TJP2" |
| 4771 | 1 | PSMB4 | FALSE | "PSMB4" |
| 4774 | 1 | NPPA | FALSE | "NPPA" |
| 4780 | 1 | USP33 | FALSE | "USP33" |
| 4783 | 2 | WWOX | FALSE | "WWOX" |
| 4785 | 3 | KRAS | FALSE | "KRAS" |
| 4789 | 2 | MAPK13 | FALSE | "MAPK13" |
| 4792 | 1 | OPRD1 | FALSE | "OPRD1" |
| 4794 | 5 | RPL31 | FALSE | "RPL31" |
| 4798 | 1 | UHRF2 | FALSE | "UHRF2" |
| 4808 | 2 | RHOC | FALSE | "RHOC" |
| 4816 | 1 | RPL13 | FALSE | "RPL13" |
| 4819 | 1 | UNC45B | FALSE | "UNC45B" |
| 4822 | 3 | MMP2 | FALSE | "MMP2" |
| 4826 | 2 | MEF2C | FALSE | "MEF2C" |
| 4831 | 3 | UPF1 | FALSE | "UPF1" |
| 4834 | 1 | HLF | FALSE | "HLF" |
| 4836 | 1 | RYR2 | FALSE | "RYR2" |
| 4839 | 3 | EEF1G | FALSE | "EEF1G" |
| 4842 | 1 | YWHAH | FALSE | "YWHAH" |
| 4844 | 3 | HDAC3 | FALSE | "HDAC3" |
| 4848 | 1 | TSFM | FALSE | "TSFM" |
| 4850 | 1 | TNRC6A | FALSE | "TNRC6A" |
| 4852 | 1 | NEDD8-MDP1 | FALSE | "NEDD8-MDP1" |
| 4858 | 1 | WDR18 | FALSE | "WDR18" |
| 4861 | 1 | BIRC5 | FALSE | "BIRC5" |
| 4863 | 1 | KRT74 | FALSE | "KRT74" |
| 4869 | 1 | PPP3R1 | FALSE | "PPP3R1" |
| 4871 | 1 | MKL1 | FALSE | "MKL1" |
| 4873 | 1 | KLF6 | FALSE | "KLF6" |
| 4878 | 1 | KAT6A | FALSE | "KAT6A" |
| 4881 | 1 | RIOK2 | FALSE | "RIOK2" |
| 4887 | 3 | UBE3A | FALSE | "UBE3A" |
| 4890 | 1 | IKZF1 | FALSE | "IKZF1" |
| 4907 | 2 | PTPA | FALSE | "PTPA" |
| 4910 | 2 | MMP1 | FALSE | "MMP1" |
| 4913 | 2 | JUNB | FALSE | "JUNB" |
| 4918 | 1 | SEC24D | FALSE | "SEC24D" |
| 4924 | 2 | TBL1XR1 | FALSE | "TBL1XR1" |
| 4926 | 1 | MRPS5 | FALSE | "MRPS5" |
| 4934 | 1 | PTGES3 | FALSE | "PTGES3" |
| 4936 | 1 | PHF21A | FALSE | "PHF21A" |
| 4942 | 3 | PABPN1 | FALSE | "PABPN1" |
| 4945 | 2 | KRT5 | FALSE | "KRT5" |
| 4952 | 1 | SFI1 | FALSE | "SFI1" |
| 4954 | 1 | CD1D | FALSE | "CD1D" |
| 4956 | 1 | IGBP1 | FALSE | "IGBP1" |
| 4961 | 1 | SH3KBP1 | FALSE | "SH3KBP1" |
| 4963 | 2 | LGALS3 | FALSE | "LGALS3" |
| 4970 | 1 | HIST1H2AJ | FALSE | "HIST1H2AJ" |
| 4973 | 1 | PEX19 | FALSE | "PEX19" |
| 4976 | 2 | RNMT | FALSE | "RNMT" |
| 4982 | 1 | HNRNPH1 | FALSE | "HNRNPH1" |
| 4984 | 1 | TBK1 | FALSE | "TBK1" |
| 4990 | 2 | RBM8A | FALSE | "RBM8A" |
| 4992 | 2 | CASP6 | FALSE | "CASP6" |
| 4996 | 1 | IMPDH2 | FALSE | "IMPDH2" |
| 4999 | 1 | RRM2 | FALSE | "RRM2" |
| 5002 | 2 | SNRPN | FALSE | "SNRPN" |
| 5004 | 1 | TOP2A | FALSE | "TOP2A" |
| 5007 | 1 | HERC5 | FALSE | "HERC5" |
| 5009 | 1 | RPSAP58 | FALSE | "RPSAP58" |
| 5011 | 1 | HDAC6 | FALSE | "HDAC6" |
| 5013 | 1 | HIST1H2BB | FALSE | "HIST1H2BB" |
| 5020 | 1 | HR | FALSE | "HR" |
| 5022 | 2 | RPS6KA1 | FALSE | "RPS6KA1" |
| 5024 | 2 | UGT1A6 | FALSE | "UGT1A6" |
| 5034 | 3 | RAF1 | FALSE | "RAF1" |
| 5039 | 2 | EIF3L | FALSE | "EIF3L" |
| 5042 | 1 | IL6ST | FALSE | "IL6ST" |
| 5045 | 2 | POLR3K | FALSE | "POLR3K" |
| 5048 | 2 | HNRNPUL1 | FALSE | "HNRNPUL1" |
| 5052 | 1 | PSMB10 | FALSE | "PSMB10" |
| 5054 | 2 | TPR | FALSE | "TPR" |
| 5056 | 2 | RANGAP1 | FALSE | "RANGAP1" |
| 5058 | 1 | DSG1 | FALSE | "DSG1" |
| 5061 | 2 | PAPOLA | FALSE | "PAPOLA" |
| 5065 | 1 | MED21 | FALSE | "MED21" |
| 5068 | 2 | EIF5B | FALSE | "EIF5B" |
| 5070 | 2 | TGM2 | FALSE | "TGM2" |
| 5072 | 1 | RAD51 | FALSE | "RAD51" |
| 5076 | 1 | HIST1H2BH | FALSE | "HIST1H2BH" |
| 5079 | 1 | MMP3 | FALSE | "MMP3" |
| 5081 | 1 | PSMB9 | FALSE | "PSMB9" |
| 5083 | 1 | RRM1 | FALSE | "RRM1" |
| 5085 | 1 | UNC5A | FALSE | "UNC5A" |
| 5088 | 1 | KAT5 | FALSE | "KAT5" |
| 5091 | 1 | HTT | FALSE | "HTT" |
| 5093 | 1 | SLC2A7 | FALSE | "SLC2A7" |
| 5095 | 1 | PTGES | FALSE | "PTGES" |
| 5102 | 1 | PRDM4 | FALSE | "PRDM4" |
| 5104 | 1 | LIF | FALSE | "LIF" |
| 5108 | 1 | TNRC6B | FALSE | "TNRC6B" |
| 5115 | 1 | HLA-C | FALSE | "HLA-C" |
| 5117 | 2 | TCEA1 | FALSE | "TCEA1" |
| 5124 | 1 | POLR3H | FALSE | "POLR3H" |
| 5129 | 1 | TUBG2 | FALSE | "TUBG2" |
| 5132 | 1 | TPO | FALSE | "TPO" |
| 5136 | 1 | TGFBR2 | FALSE | "TGFBR2" |
| 5142 | 1 | PPP4R2 | FALSE | "PPP4R2" |
| 5163 | 1 | PUF60 | FALSE | "PUF60" |
| 5165 | 1 | TUFM | FALSE | "TUFM" |
| 5167 | 1 | MECOM | FALSE | "MECOM" |
| 5169 | 1 | GNRH1 | FALSE | "GNRH1" |
| 5177 | 2 | VMP1 | FALSE | "VMP1" |
| 5179 | 1 | HFM1 | FALSE | "HFM1" |
| 5186 | 1 | PHF12 | FALSE | "PHF12" |
| 5188 | 1 | SOCS1 | FALSE | "SOCS1" |
| 5191 | 3 | NR3C1 | FALSE | "NR3C1" |
| 5195 | 1 | MMP14 | FALSE | "MMP14" |
| 5200 | 2 | MAZ | FALSE | "MAZ" |
| 5203 | 1 | EIF4E2 | FALSE | "EIF4E2" |
| 5206 | 1 | SSNA1 | FALSE | "SSNA1" |
| 5216 | 1 | KRT71 | FALSE | "KRT71" |
| 5221 | 3 | POLR3A | FALSE | "POLR3A" |
| 5229 | 1 | RBL2 | FALSE | "RBL2" |
| 5232 | 1 | MRPS18A | FALSE | "MRPS18A" |
| 5238 | 1 | GSK3B | FALSE | "GSK3B" |
| 5246 | 1 | IFNGR1 | FALSE | "IFNGR1" |
| 5248 | 1 | MCM8 | FALSE | "MCM8" |
| 5254 | 1 | SMC4 | FALSE | "SMC4" |
| 5262 | 1 | HIST4H4 | FALSE | "HIST4H4" |
| 5276 | 1 | XPA | FALSE | "XPA" |
| 5278 | 2 | TRIP12 | FALSE | "TRIP12" |
| 5287 | 1 | OXT | FALSE | "OXT" |
| 5289 | 1 | NOTCH4 | FALSE | "NOTCH4" |
| 5293 | 1 | TARS | FALSE | "TARS" |
| 5297 | 1 | TXNDC9 | FALSE | "TXNDC9" |
| 5304 | 1 | MRPL22 | FALSE | "MRPL22" |
| 5308 | 1 | CTTN | FALSE | "CTTN" |
| 5324 | 1 | TRPA1 | FALSE | "TRPA1" |
| 5332 | 1 | HGF | FALSE | "HGF" |
| 5334 | 1 | STAT2 | FALSE | "STAT2" |
| 5336 | 1 | IL13RA1 | FALSE | "IL13RA1" |
| 5346 | 1 | PPP2R5C | FALSE | "PPP2R5C" |
| 5348 | 1 | POLE | FALSE | "POLE" |
| 5354 | 1 | REST | FALSE | "REST" |
| 5359 | 1 | PRDM6 | FALSE | "PRDM6" |
| 5371 | 1 | MDM4 | FALSE | "MDM4" |
| 5374 | 1 | PPOX | FALSE | "PPOX" |
| 5381 | 1 | WDR73 | FALSE | "WDR73" |
| 5391 | 1 | TGS1 | FALSE | "TGS1" |
| 5394 | 1 | USP18 | FALSE | "USP18" |
| 5404 | 1 | IL3RA | FALSE | "IL3RA" |
| 5410 | 1 | OLR1 | FALSE | "OLR1" |
| 5414 | 1 | IGFBP1 | FALSE | "IGFBP1" |
| 5416 | 1 | DAPK1 | FALSE | "DAPK1" |
| 5419 | 1 | PRKCSH | FALSE | "PRKCSH" |
| 5422 | 1 | RIPK3 | FALSE | "RIPK3" |
| 5427 | 1 | ORC6 | FALSE | "ORC6" |
| 5430 | 1 | HDAC8 | FALSE | "HDAC8" |
| 5432 | 1 | IL4R | FALSE | "IL4R" |
| 5436 | 1 | MAP3K7 | FALSE | "MAP3K7" |
| 5439 | 1 | TBL1Y | FALSE | "TBL1Y" |
| 5443 | 2 | HMOX1 | FALSE | "HMOX1" |
| 5445 | 1 | FOSL1 | FALSE | "FOSL1" |
| 5451 | 1 | ENPEP | FALSE | "ENPEP" |
| 5454 | 1 | CD3D | FALSE | "CD3D" |
| 5458 | 3 | ILF2 | FALSE | "ILF2" |
| 5459 | 1 | ZFR | FALSE | "ZFR" |
| 5464 | 1 | MYBL1 | FALSE | "MYBL1" |
| 5474 | 1 | USP19 | FALSE | "USP19" |
| 5476 | 1 | ORC3 | FALSE | "ORC3" |
| 5479 | 1 | NR1D2 | FALSE | "NR1D2" |
| 5481 | 2 | MED1 | FALSE | "MED1" |
| 5483 | 1 | PPP1CC | FALSE | "PPP1CC" |
| 5492 | 1 | TUBG1 | FALSE | "TUBG1" |
| 5496 | 1 | RPA4 | FALSE | "RPA4" |
| 5501 | 1 | MAL | FALSE | "MAL" |
| 5505 | 1 | GATA2 | FALSE | "GATA2" |
| 5507 | 1 | HES1 | FALSE | "HES1" |
| 5513 | 1 | RPS27L | FALSE | "RPS27L" |
| 5516 | 1 | SUZ12 | FALSE | "SUZ12" |
| 5524 | 1 | IL27RA | FALSE | "IL27RA" |
| 5528 | 1 | RERE | FALSE | "RERE" |
| 5537 | 1 | IL3 | FALSE | "IL3" |
| 5545 | 1 | GATA4 | FALSE | "GATA4" |
| 5552 | 2 | MAFB | FALSE | "MAFB" |
| 5558 | 1 | LATS2 | FALSE | "LATS2" |
| 5565 | 1 | GCG | FALSE | "GCG" |
| 5569 | 1 | SAA2 | FALSE | "SAA2" |
| 5573 | 1 | ZMYM2 | FALSE | "ZMYM2" |
| 5576 | 1 | MMP13 | FALSE | "MMP13" |
| 5578 | 1 | PSMB8 | FALSE | "PSMB8" |
| 5582 | 1 | TNFRSF1B | FALSE | "TNFRSF1B" |
| 5586 | 1 | HIST2H2AA4 | FALSE | "HIST2H2AA4" |
| 5588 | 1 | ZFR2 | FALSE | "ZFR2" |
| 5591 | 1 | HIST1H2AM | FALSE | "HIST1H2AM" |
| 5593 | 1 | MRPL12 | FALSE | "MRPL12" |
| 5602 | 1 | WDR38 | FALSE | "WDR38" |
