# Supplementary material for: Dynamic Network Biomarker of Pre-Exhausted CD8+ T Cells Contributed to T Cell Exhaustion in Colorectal Cancer
Source: Front Immunol. 2021 Aug 9;12:691142. doi: 10.3389/fimmu.2021.691142 (PMC8381053; doi:10.3389/fimmu.2021.691142)
Supplement: Supplementary file 7 [file Table_4.docx]

|  | baseMean | log2FoldChange | lfcSE | stat | pvalue | padj |
| --- | --- | --- | --- | --- | --- | --- |
| KIFC1 | 16.14221298 | -25.75021194 | 0.864899375 | -29.77249456 | 8.87E-195 | 9.99E-191 |
| SPC25 | 33.42088736 | -10.85429717 | 0.674410019 | -16.09450759 | 2.79E-58 | 1.57E-54 |
| SHCBP1 | 33.54083176 | -9.870832624 | 0.670693199 | -14.71735906 | 4.99E-49 | 1.87E-45 |
| HJURP | 29.73370083 | -10.75857282 | 0.751730537 | -14.3117411 | 1.85E-46 | 5.20E-43 |
| DIAPH3 | 13.14862849 | -10.64217646 | 0.757892087 | -14.0418097 | 8.65E-45 | 1.95E-41 |
| KIF18B | 15.44726662 | -10.35034362 | 0.76911136 | -13.45753574 | 2.78E-41 | 4.93E-38 |
| CDCA2 | 30.58055982 | -10.29061126 | 0.765080542 | -13.45036331 | 3.06E-41 | 4.93E-38 |
| ANLN | 43.31661846 | -9.212586406 | 0.689069198 | -13.36960995 | 9.10E-41 | 1.28E-37 |
| SKA3 | 21.25383101 | -10.58675777 | 0.80536932 | -13.14522109 | 1.81E-39 | 2.27E-36 |
| HMMR | 20.53281208 | -10.82136738 | 0.82864078 | -13.05917793 | 5.63E-39 | 6.35E-36 |
| E2F1 | 13.21720298 | -10.19598587 | 0.796019868 | -12.80870777 | 1.47E-37 | 1.50E-34 |
| SLC29A1 | 20.95683832 | -9.536648954 | 0.745942994 | -12.78468868 | 2.00E-37 | 1.87E-34 |
| KIF15 | 57.12047605 | -7.562902896 | 0.609464917 | -12.40908653 | 2.33E-35 | 2.02E-32 |
| C2orf48 | 13.2799142 | -10.1441277 | 0.817951146 | -12.40187479 | 2.55E-35 | 2.05E-32 |
| RAD54L | 24.65982288 | -5.972222954 | 0.486262384 | -12.28189378 | 1.13E-34 | 8.51E-32 |
| E2F7 | 13.2926404 | -8.722644116 | 0.711264563 | -12.26357191 | 1.42E-34 | 1.00E-31 |
| DLGAP5 | 44.44458612 | -8.719809461 | 0.712575201 | -12.23703751 | 1.97E-34 | 1.31E-31 |
| RRM2 | 336.1754093 | -6.939851292 | 0.571225776 | -12.14905136 | 5.80E-34 | 3.63E-31 |
| PBK | 27.94510943 | -11.31152832 | 0.93720075 | -12.06948278 | 1.53E-33 | 9.08E-31 |
| TRIP13 | 18.85740093 | -11.06668783 | 0.936079751 | -11.82237712 | 2.99E-32 | 1.68E-29 |
| TEDC2 | 9.7448395 | -11.75751283 | 1.016709941 | -11.56427448 | 6.25E-31 | 3.35E-28 |
| NEIL3 | 15.15731942 | -10.09764421 | 0.891576694 | -11.3256036 | 9.80E-30 | 5.02E-27 |
| E2F8 | 10.09993 | -10.99959139 | 0.972088601 | -11.31542061 | 1.10E-29 | 5.39E-27 |
| KIF4A | 24.01662873 | -8.706257071 | 0.789929405 | -11.02156347 | 3.01E-28 | 1.41E-25 |
| TONSL | 10.62486844 | -8.704890885 | 0.792613789 | -10.98251256 | 4.64E-28 | 2.09E-25 |
| MYBL2 | 17.13097899 | -9.133887345 | 0.837005584 | -10.91257636 | 1.00E-27 | 4.35E-25 |
| DDIAS | 17.42455693 | -9.395533654 | 0.86277512 | -10.8898987 | 1.29E-27 | 5.37E-25 |
| CLSPN | 66.67781613 | -5.009065566 | 0.460670956 | -10.873413 | 1.54E-27 | 6.21E-25 |
| FAM111B | 74.60257501 | -6.395907151 | 0.593245235 | -10.78121959 | 4.22E-27 | 1.64E-24 |
| POLQ | 30.1774093 | -7.599070994 | 0.705383843 | -10.77295868 | 4.62E-27 | 1.73E-24 |
| CDC20 | 33.79832288 | -8.40963834 | 0.792425002 | -10.61253534 | 2.61E-26 | 9.47E-24 |
| PLK1 | 21.00636741 | -9.209117342 | 0.876604904 | -10.50543671 | 8.15E-26 | 2.87E-23 |
| MIS18A | 7.677101672 | -8.190698108 | 0.786314287 | -10.41657038 | 2.08E-25 | 7.11E-23 |
| TUBA1B | 1010.942142 | -2.633324889 | 0.254695505 | -10.33911019 | 4.69E-25 | 1.55E-22 |
| HIST1H4C | 38.85697869 | -2.67694122 | 0.262713671 | -10.18957714 | 2.21E-24 | 7.10E-22 |
| STMN1 | 732.0447362 | -3.488824601 | 0.343146845 | -10.16714755 | 2.78E-24 | 8.70E-22 |
| KIF23 | 61.94251342 | -6.631146445 | 0.654630805 | -10.12959732 | 4.08E-24 | 1.24E-21 |
| MCM10 | 32.13960069 | -6.545362864 | 0.649499726 | -10.07754523 | 6.94E-24 | 2.06E-21 |
| DEPDC1 | 9.803260782 | -11.64419557 | 1.156831715 | -10.06559158 | 7.84E-24 | 2.26E-21 |
| E2F2 | 11.80178977 | -8.237163465 | 0.82323552 | -10.00584069 | 1.44E-23 | 4.05E-21 |
| MIR3917 | 49.99646787 | -3.625993851 | 0.363007706 | -9.988751726 | 1.71E-23 | 4.69E-21 |
| POC1A | 18.68746926 | -8.487149326 | 0.8510857 | -9.972144203 | 2.02E-23 | 5.41E-21 |
| UBE2C | 70.85784659 | -6.675849061 | 0.675735314 | -9.879384606 | 5.11E-23 | 1.34E-20 |
| KIF2C | 40.35407929 | -6.360224517 | 0.647588415 | -9.821399476 | 9.11E-23 | 2.31E-20 |
| CDCA3 | 19.84487433 | -8.132486699 | 0.828150397 | -9.820060129 | 9.23E-23 | 2.31E-20 |
| GINS1 | 26.83383987 | -7.192253839 | 0.740625377 | -9.711055086 | 2.71E-22 | 6.57E-20 |
| TOP2A | 149.5892834 | -4.964098885 | 0.511249983 | -9.709729191 | 2.74E-22 | 6.57E-20 |
| ORC6 | 18.92280388 | -6.363582642 | 0.659034551 | -9.655916566 | 4.64E-22 | 1.09E-19 |
| GTSE1 | 20.92582239 | -6.403541382 | 0.66711654 | -9.598834687 | 8.09E-22 | 1.86E-19 |
| C21orf58 | 8.720681601 | -6.095526578 | 0.636522715 | -9.57629074 | 1.01E-21 | 2.27E-19 |
| CDT1 | 13.38912536 | -6.396139711 | 0.668962143 | -9.561288006 | 1.16E-21 | 2.57E-19 |
| TTK | 25.75351362 | -7.729480595 | 0.817612986 | -9.453715547 | 3.27E-21 | 7.08E-19 |
| TYMS | 260.9692803 | -4.093219047 | 0.433463859 | -9.443045745 | 3.62E-21 | 7.70E-19 |
| CKAP2L | 43.58979773 | -6.815888982 | 0.722403903 | -9.435011294 | 3.91E-21 | 8.15E-19 |
| APOBEC3B | 17.52179925 | -4.408169331 | 0.469166448 | -9.395747189 | 5.68E-21 | 1.16E-18 |
| PKMYT1 | 56.23483555 | -5.20969993 | 0.558116284 | -9.334434568 | 1.02E-20 | 2.04E-18 |
| DMC1 | 6.691280119 | -9.971673772 | 1.076594898 | -9.262233909 | 2.00E-20 | 3.96E-18 |
| APOBEC3A-B | 5.179303193 | -5.006118693 | 0.545354927 | -9.179560762 | 4.33E-20 | 8.41E-18 |
| GINS3 | 19.63208129 | -6.978282844 | 0.766192789 | -9.107737565 | 8.41E-20 | 1.61E-17 |
| RAD51 | 43.25203951 | -6.262770406 | 0.691471139 | -9.057168196 | 1.34E-19 | 2.51E-17 |
| STIL | 34.76668403 | -6.165259027 | 0.682015734 | -9.039760692 | 1.57E-19 | 2.90E-17 |
| TUBB | 1061.005314 | -2.33964291 | 0.25911135 | -9.029488336 | 1.72E-19 | 3.13E-17 |
| MCM8 | 6.140686169 | -9.239560491 | 1.025872524 | -9.006538603 | 2.13E-19 | 3.80E-17 |
| PON3 | 9.164785342 | -9.9705648 | 1.109137318 | -8.989477354 | 2.48E-19 | 4.37E-17 |
| CENPW | 8.189379082 | -5.330441612 | 0.594954011 | -8.95941789 | 3.26E-19 | 5.66E-17 |
| FSD1 | 8.740216517 | -8.446201924 | 0.943158482 | -8.955230843 | 3.39E-19 | 5.79E-17 |
| ZWINT | 147.9221019 | -4.490567673 | 0.505188165 | -8.888901172 | 6.17E-19 | 1.04E-16 |
| TYMSOS | 36.48197651 | -4.094462181 | 0.471870236 | -8.677093546 | 4.06E-18 | 6.63E-16 |
| CCNB1 | 18.80360874 | -6.900926739 | 0.810269839 | -8.516825388 | 1.64E-17 | 2.64E-15 |
| ESCO2 | 28.60078438 | -6.034720652 | 0.717260955 | -8.413563581 | 3.98E-17 | 6.31E-15 |
| CREG1 | 7.589647471 | -6.420127369 | 0.764460911 | -8.398241533 | 4.53E-17 | 7.09E-15 |
| SKA1 | 18.69730775 | -7.023405439 | 0.836594857 | -8.395229041 | 4.65E-17 | 7.17E-15 |
| PCLAF | 130.1619782 | -3.989242808 | 0.478276281 | -8.340875275 | 7.37E-17 | 1.12E-14 |
| TK1 | 130.1449006 | -4.285867596 | 0.518536606 | -8.265313467 | 1.39E-16 | 2.09E-14 |
| DERL3 | 3.515773817 | -4.272388894 | 0.517370273 | -8.257894045 | 1.48E-16 | 2.20E-14 |
| CCNB2 | 50.03079812 | -6.15293937 | 0.756428362 | -8.134199722 | 4.15E-16 | 6.07E-14 |
| IL7R | 96.89845218 | 3.039682442 | 0.379757675 | 8.004268629 | 1.20E-15 | 1.71E-13 |
| CDCA8 | 50.42504857 | -5.622524089 | 0.703937065 | -7.987253929 | 1.38E-15 | 1.94E-13 |
| TSPAN4 | 10.7309809 | -7.519648873 | 0.951071225 | -7.906504452 | 2.65E-15 | 3.68E-13 |
| ERI2 | 10.079931 | -7.152924009 | 0.917647022 | -7.7948534 | 6.45E-15 | 8.86E-13 |
| TRAIP | 6.199498347 | -9.55483201 | 1.228136566 | -7.779942621 | 7.26E-15 | 9.85E-13 |
| PAK6 | 6.669324707 | -6.064860006 | 0.786680441 | -7.70943281 | 1.26E-14 | 1.69E-12 |
| ENOSF1 | 241.7701977 | -2.8491203 | 0.369681958 | -7.706949822 | 1.29E-14 | 1.71E-12 |
| ASPM | 102.1632851 | -4.520103431 | 0.587100305 | -7.699030972 | 1.37E-14 | 1.80E-12 |
| PCNA-AS1 | 84.12687337 | -2.984866803 | 0.394901716 | -7.558505527 | 4.08E-14 | 5.28E-12 |
| SGO1 | 22.27640962 | -5.327358333 | 0.708324304 | -7.521072341 | 5.43E-14 | 6.88E-12 |
| AURKA | 29.0668471 | -5.707755479 | 0.761025393 | -7.500085456 | 6.38E-14 | 7.98E-12 |
| PCNA | 281.0033883 | -2.830536644 | 0.377786395 | -7.49242609 | 6.76E-14 | 8.37E-12 |
| ASF1B | 97.34503277 | -4.131511666 | 0.554719686 | -7.447926887 | 9.48E-14 | 1.16E-11 |
| KNSTRN | 10.13013207 | -6.611570792 | 0.903445584 | -7.318172683 | 2.51E-13 | 3.04E-11 |
| TUBGCP5 | 15.21054521 | -4.968491201 | 0.683297559 | -7.27134341 | 3.56E-13 | 4.27E-11 |
| DUT | 149.674319 | -2.464279237 | 0.344460778 | -7.15401983 | 8.43E-13 | 9.99E-11 |
| SPAG5 | 50.55840438 | -5.21315533 | 0.733630278 | -7.105970797 | 1.19E-12 | 1.40E-10 |
| KIF20A | 9.328902703 | -4.686653156 | 0.661485893 | -7.085038711 | 1.39E-12 | 1.61E-10 |
| HELLS | 33.48183245 | -3.334227733 | 0.474201266 | -7.031250169 | 2.05E-12 | 2.35E-10 |
| LRRCC1 | 17.11325972 | -6.112437863 | 0.870010846 | -7.025703059 | 2.13E-12 | 2.42E-10 |
| CEP55 | 19.00907642 | -5.93268579 | 0.851552876 | -6.966902416 | 3.24E-12 | 3.61E-10 |
| UHRF1 | 25.55692971 | -4.170213089 | 0.59953949 | -6.955693762 | 3.51E-12 | 3.87E-10 |
| SGO1-AS1 | 8.603781373 | -5.389872543 | 0.776038816 | -6.945364627 | 3.77E-12 | 4.13E-10 |
| CENPS-CORT | 5.373757687 | -5.808196664 | 0.838685512 | -6.925357097 | 4.35E-12 | 4.71E-10 |
| LOC642846 | 2.418655202 | -3.810420993 | 0.555132739 | -6.863981759 | 6.70E-12 | 7.18E-10 |
| BRIP1 | 19.96885586 | -5.083971721 | 0.754666013 | -6.736717478 | 1.62E-11 | 1.72E-09 |
| CDK1 | 128.4040112 | -4.044139736 | 0.601271491 | -6.725979525 | 1.74E-11 | 1.84E-09 |
| ST3GAL4 | 12.05238401 | -6.038781387 | 0.926964956 | -6.514573551 | 7.29E-11 | 7.60E-09 |
| MCM4 | 189.2725322 | -2.800077337 | 0.43360687 | -6.457640615 | 1.06E-10 | 1.10E-08 |
| KNL1 | 71.29215389 | -3.883160069 | 0.608450094 | -6.382051886 | 1.75E-10 | 1.79E-08 |
| TMEM106C | 194.3684779 | -2.477192065 | 0.388402652 | -6.377896888 | 1.80E-10 | 1.82E-08 |
| NUP155 | 19.9801833 | -3.885139997 | 0.609998828 | -6.3690942 | 1.90E-10 | 1.91E-08 |
| NET1 | 8.932651131 | -5.541798438 | 0.870652469 | -6.365109658 | 1.95E-10 | 1.95E-08 |
| ATP23 | 10.1939114 | -5.618881552 | 0.885064099 | -6.348558887 | 2.17E-10 | 2.15E-08 |
| NUSAP1 | 170.7089983 | -2.51809127 | 0.399119009 | -6.309123876 | 2.81E-10 | 2.75E-08 |
| CHEK2 | 5.578561193 | -5.824134058 | 0.934941739 | -6.229408545 | 4.68E-10 | 4.55E-08 |
| MELK | 69.47606364 | -4.098897011 | 0.665355761 | -6.160459187 | 7.25E-10 | 6.98E-08 |
| HMGB3 | 17.21733272 | -4.183050369 | 0.686247191 | -6.095544611 | 1.09E-09 | 1.04E-07 |
| BUB1B | 52.55891902 | -4.112874138 | 0.675194969 | -6.091387419 | 1.12E-09 | 1.06E-07 |
| NUF2 | 32.4116377 | -4.730283374 | 0.778793473 | -6.073861095 | 1.25E-09 | 1.17E-07 |
| MND1 | 7.76823506 | -5.584422459 | 0.921211931 | -6.06203879 | 1.34E-09 | 1.25E-07 |
| SPC24 | 4.350449195 | -5.577846485 | 0.923814311 | -6.037843772 | 1.56E-09 | 1.44E-07 |
| FOXM1 | 14.44168496 | -4.649519485 | 0.773199091 | -6.013353532 | 1.82E-09 | 1.65E-07 |
| KIAA1841 | 7.614486471 | -5.27966744 | 0.878438524 | -6.010286771 | 1.85E-09 | 1.66E-07 |
| GUSBP4 | 3.622691329 | -4.347115538 | 0.72664686 | -5.982432149 | 2.20E-09 | 1.95E-07 |
| KIAA0895 | 2.815812174 | -4.968334381 | 0.831052182 | -5.978366325 | 2.25E-09 | 1.98E-07 |
| DTL | 71.73489072 | -3.430012316 | 0.575284117 | -5.962292748 | 2.49E-09 | 2.16E-07 |
| ATAD2 | 111.9900405 | -2.469042004 | 0.415147671 | -5.947382529 | 2.72E-09 | 2.34E-07 |
| ALYREF | 6.30670428 | -4.337619767 | 0.735684025 | -5.896036371 | 3.72E-09 | 3.15E-07 |
| NCAPG2 | 65.57935737 | -3.054774744 | 0.523990667 | -5.829826629 | 5.55E-09 | 4.56E-07 |
| SPDL1 | 23.21417204 | -4.76228327 | 0.817812656 | -5.823195857 | 5.77E-09 | 4.71E-07 |
| CENPN | 60.61141304 | -2.073310541 | 0.35939986 | -5.768812872 | 7.98E-09 | 6.42E-07 |
| TRIM24 | 6.495229256 | -4.76388937 | 0.829859052 | -5.7406006 | 9.43E-09 | 7.51E-07 |
| TMEM177 | 5.708247885 | -5.65271474 | 0.984783372 | -5.740059085 | 9.46E-09 | 7.51E-07 |
| TROAP | 22.74959386 | -4.656537521 | 0.811894921 | -5.735394323 | 9.73E-09 | 7.66E-07 |
| CCNA2 | 103.7021649 | -2.799771936 | 0.488272875 | -5.734031279 | 9.81E-09 | 7.67E-07 |
| CDCA5 | 49.98292203 | -4.389001875 | 0.768918358 | -5.708020663 | 1.14E-08 | 8.76E-07 |
| CENPS | 5.403202594 | -5.1011495 | 0.893889961 | -5.706686192 | 1.15E-08 | 8.77E-07 |
| MCM7 | 437.7554707 | -2.018792955 | 0.354453822 | -5.695503424 | 1.23E-08 | 9.30E-07 |
| MKI67 | 174.0980812 | -2.615307368 | 0.459721077 | -5.688900293 | 1.28E-08 | 9.54E-07 |
| DCLRE1B | 16.87505182 | -4.613847953 | 0.813870356 | -5.669020769 | 1.44E-08 | 1.06E-06 |
| MTFR2 | 5.495421943 | -5.821551958 | 1.028299719 | -5.661337692 | 1.50E-08 | 1.11E-06 |
| CD200 | 5.548940341 | -5.048179533 | 0.894543788 | -5.64330064 | 1.67E-08 | 1.22E-06 |
| BICD2 | 4.138511579 | -5.41138582 | 0.979118517 | -5.526793466 | 3.26E-08 | 2.31E-06 |
| CENPM | 58.96041782 | -2.86164376 | 0.517808154 | -5.526455571 | 3.27E-08 | 2.31E-06 |
| CMSS1 | 9.774654182 | -4.057389519 | 0.737444027 | -5.501962683 | 3.76E-08 | 2.63E-06 |
| SLC12A4 | 2.591311886 | -4.124518365 | 0.752574954 | -5.480541632 | 4.24E-08 | 2.95E-06 |
| PNPO | 4.023056156 | -4.638904702 | 0.847192914 | -5.475617921 | 4.36E-08 | 3.00E-06 |
| BIRC5 | 47.08819083 | -4.101804092 | 0.749115777 | -5.475527573 | 4.36E-08 | 3.00E-06 |
| IL2RA | 10.01016682 | -3.054215424 | 0.560419403 | -5.449874523 | 5.04E-08 | 3.44E-06 |
| MAD2L1 | 108.3843742 | -2.693698479 | 0.496338304 | -5.427142041 | 5.73E-08 | 3.89E-06 |
| LIG1 | 95.51913054 | -2.350857411 | 0.433817426 | -5.419001799 | 5.99E-08 | 4.04E-06 |
| AGMAT | 3.436862966 | -3.935627031 | 0.727599587 | -5.409056164 | 6.34E-08 | 4.25E-06 |
| CDC6 | 41.49528547 | -4.014877791 | 0.74394899 | -5.396711128 | 6.79E-08 | 4.52E-06 |
| MCM2 | 112.6623099 | -2.63520811 | 0.488416484 | -5.395411898 | 6.84E-08 | 4.53E-06 |
| TEDC1 | 5.239088629 | -5.81014745 | 1.077166881 | -5.393915792 | 6.89E-08 | 4.54E-06 |
| DHFR | 10.49119724 | -2.426191264 | 0.45012255 | -5.390068248 | 7.04E-08 | 4.61E-06 |
| CUL7 | 3.18576857 | -4.381010146 | 0.819490972 | -5.346013922 | 8.99E-08 | 5.85E-06 |
| ST7 | 11.40655421 | -4.641217491 | 0.869011076 | -5.340803611 | 9.25E-08 | 5.99E-06 |
| GEN1 | 9.682559157 | -4.061088051 | 0.763366974 | -5.319968231 | 1.04E-07 | 6.68E-06 |
| CDC25C | 9.197577331 | -3.468121921 | 0.653650215 | -5.305776456 | 1.12E-07 | 7.16E-06 |
| PELP1 | 4.482624574 | -3.049520033 | 0.574806679 | -5.305296797 | 1.12E-07 | 7.16E-06 |
| C18orf54 | 5.330505857 | -5.682932643 | 1.074171814 | -5.290524818 | 1.22E-07 | 7.67E-06 |
| KIF14 | 27.8878333 | -4.448860327 | 0.8412614 | -5.288321001 | 1.23E-07 | 7.72E-06 |
| SAC3D1 | 3.413953316 | -3.373337586 | 0.638068081 | -5.286798836 | 1.24E-07 | 7.75E-06 |
| HIST1H1D | 20.14309568 | -2.279556009 | 0.431281582 | -5.285539898 | 1.25E-07 | 7.76E-06 |
| TCEAL1 | 5.569763386 | -5.673838567 | 1.08545726 | -5.227141389 | 1.72E-07 | 1.05E-05 |
| ATG4C | 4.518263328 | -5.295107349 | 1.017332228 | -5.204894925 | 1.94E-07 | 1.18E-05 |
| ATAD3A | 8.060960124 | -4.069942426 | 0.783973552 | -5.191428223 | 2.09E-07 | 1.26E-05 |
| RMI1 | 19.43103773 | -3.844522018 | 0.743475264 | -5.17101537 | 2.33E-07 | 1.39E-05 |
| ATAD5 | 70.54056184 | -2.6943393 | 0.522077072 | -5.160807556 | 2.46E-07 | 1.46E-05 |
| CRYL1 | 9.164897046 | -3.28627506 | 0.637640211 | -5.153807747 | 2.55E-07 | 1.51E-05 |
| SLC2A13 | 3.681191738 | -4.259067788 | 0.827886332 | -5.144507916 | 2.68E-07 | 1.57E-05 |
| SNUPN | 10.81848217 | -4.056470204 | 0.788893614 | -5.141973685 | 2.72E-07 | 1.59E-05 |
| KIF22 | 129.6341507 | -2.087568377 | 0.407929258 | -5.11747646 | 3.10E-07 | 1.80E-05 |
| CTNNAL1 | 32.2861626 | -2.966866364 | 0.581807138 | -5.099398361 | 3.41E-07 | 1.97E-05 |
| ADPRH | 10.88096122 | -4.598947883 | 0.902250965 | -5.097193645 | 3.45E-07 | 1.97E-05 |
| PIDD1 | 21.04648949 | -3.507770463 | 0.694155734 | -5.05329034 | 4.34E-07 | 2.45E-05 |
| ESPL1 | 4.752913158 | -5.686904412 | 1.129821621 | -5.03345334 | 4.82E-07 | 2.69E-05 |
| CIP2A | 39.19734025 | -3.153353556 | 0.627392031 | -5.026129439 | 5.00E-07 | 2.78E-05 |
| FEN1 | 117.9557957 | -2.720443158 | 0.541532283 | -5.023602921 | 5.07E-07 | 2.80E-05 |
| NCAPH | 61.43484883 | -3.108349547 | 0.619497782 | -5.01753136 | 5.23E-07 | 2.86E-05 |
| CENPF | 104.2213844 | -2.724614426 | 0.54358005 | -5.012351774 | 5.38E-07 | 2.93E-05 |
| DDX12P | 7.30282167 | -2.709222879 | 0.542856817 | -4.990676723 | 6.02E-07 | 3.26E-05 |
| NEK2 | 7.193340631 | -6.024748163 | 1.220805131 | -4.935061304 | 8.01E-07 | 4.26E-05 |
| FIGNL1 | 22.1320709 | -4.098296571 | 0.8307574 | -4.933205012 | 8.09E-07 | 4.28E-05 |
| REXO5 | 3.298915714 | -5.139672287 | 1.042548568 | -4.929911606 | 8.23E-07 | 4.33E-05 |
| TRIB3 | 8.605164428 | -4.053702292 | 0.825687796 | -4.909485538 | 9.13E-07 | 4.78E-05 |
| MICA | 7.98385408 | -3.571515258 | 0.728963138 | -4.899445628 | 9.61E-07 | 5.01E-05 |
| TICRR | 4.239918875 | -5.117959403 | 1.044910129 | -4.89799004 | 9.68E-07 | 5.03E-05 |
| AARS2 | 6.895190154 | -3.924139112 | 0.801842653 | -4.893901687 | 9.89E-07 | 5.11E-05 |
| UBP1 | 11.92492796 | -3.325027387 | 0.679710694 | -4.891827386 | 9.99E-07 | 5.14E-05 |
| ZNF615 | 3.87150444 | -4.449805525 | 0.911969088 | -4.879338109 | 1.06E-06 | 5.45E-05 |
| SMC2 | 113.7182895 | -2.241312947 | 0.460082267 | -4.871548217 | 1.11E-06 | 5.64E-05 |
| FBXO43 | 5.983039392 | -5.720025617 | 1.178231503 | -4.854755284 | 1.21E-06 | 6.12E-05 |
| CUTALP | 10.95186724 | 2.837685815 | 0.585148138 | 4.849516956 | 1.24E-06 | 6.25E-05 |
| GINS2 | 69.36943993 | -2.887161387 | 0.59778553 | -4.82976125 | 1.37E-06 | 6.87E-05 |
| ZNF420 | 8.199146548 | -4.13827333 | 0.857211623 | -4.82759825 | 1.38E-06 | 6.89E-05 |
| CHAC2 | 8.729591073 | -4.547764156 | 0.945818131 | -4.808286084 | 1.52E-06 | 7.52E-05 |
| MTBP | 12.1694999 | -3.994248688 | 0.831377556 | -4.804373966 | 1.55E-06 | 7.62E-05 |
| EXO1 | 34.22704333 | -4.179139642 | 0.869949259 | -4.803888959 | 1.56E-06 | 7.62E-05 |
| XRCC2 | 6.788681636 | -5.776150738 | 1.20308235 | -4.801126654 | 1.58E-06 | 7.69E-05 |
| ACTG2 | 7.528950663 | -4.313573019 | 0.899285199 | -4.796668538 | 1.61E-06 | 7.83E-05 |
| RRM1 | 178.3312219 | -2.015548211 | 0.420974099 | -4.787820003 | 1.69E-06 | 8.15E-05 |
| PPP1R3B | 2.39176645 | 4.810027386 | 1.006150408 | 4.780624594 | 1.75E-06 | 8.41E-05 |
| PLEKHM2 | 3.69384814 | -4.72466204 | 0.99132896 | -4.765988113 | 1.88E-06 | 9.01E-05 |
| RAD51AP1 | 48.70979466 | -3.239915291 | 0.679950171 | -4.764930472 | 1.89E-06 | 9.01E-05 |
| FANCD2 | 65.67602397 | -2.444100843 | 0.513876625 | -4.756201634 | 1.97E-06 | 9.34E-05 |
| RPS28 | 1.553002889 | -2.109879314 | 0.444977847 | -4.741537877 | 2.12E-06 | 1.00E-04 |
| ORC1 | 16.835368 | -4.603955815 | 0.973502325 | -4.729270488 | 2.25E-06 | 0.000105315 |
| NDC80 | 68.75494338 | -2.422047266 | 0.512423126 | -4.726654875 | 2.28E-06 | 0.000106239 |
| TMEM185B | 2.940934845 | -4.52580979 | 0.960034946 | -4.714213592 | 2.43E-06 | 0.000111754 |
| PLXNB2 | 2.795935162 | -4.743914448 | 1.006376687 | -4.713855664 | 2.43E-06 | 0.000111754 |
| DSCC1 | 20.28142552 | -4.039564775 | 0.857664827 | -4.709957371 | 2.48E-06 | 0.00011345 |
| IFT46 | 3.610562711 | -5.11824673 | 1.08724137 | -4.70755333 | 2.51E-06 | 0.000114331 |
| METTL4 | 12.59997163 | -3.425324729 | 0.730865502 | -4.686669053 | 2.78E-06 | 0.000126124 |
| UBE2T | 56.45153864 | -2.482553069 | 0.531334632 | -4.672296736 | 2.98E-06 | 0.000134738 |
| OIP5 | 14.9488894 | -3.558591624 | 0.767728001 | -4.635224482 | 3.57E-06 | 0.000159372 |
| CD40LG | 0.9531731 | 2.370672867 | 0.515861931 | 4.595556922 | 4.32E-06 | 0.000189902 |
| DEAF1 | 2.261912682 | -3.906805954 | 0.851191423 | -4.589808884 | 4.44E-06 | 0.000194447 |
| ZNF761 | 9.23119076 | -3.001324354 | 0.655058388 | -4.581766157 | 4.61E-06 | 0.000201067 |
| PANX1 | 7.718957816 | -4.297443362 | 0.938061555 | -4.581195488 | 4.62E-06 | 0.000201067 |
| ZNF93 | 1.930552391 | -4.243919141 | 0.928324593 | -4.571589693 | 4.84E-06 | 0.000208654 |
| EID3 | 3.654580939 | -4.604400357 | 1.007299848 | -4.571032515 | 4.85E-06 | 0.000208654 |
| C4orf46 | 24.96559765 | -3.438549751 | 0.753965378 | -4.560620221 | 5.10E-06 | 0.000215976 |
| DPY19L4 | 2.311072839 | -4.260031532 | 0.9352477 | -4.554976751 | 5.24E-06 | 0.000221025 |
| SMAD5 | 9.205229518 | -3.582943167 | 0.78893593 | -4.54148814 | 5.59E-06 | 0.0002339 |
| ATP11A | 4.667474311 | -4.420114239 | 0.977912489 | -4.519948656 | 6.19E-06 | 0.000257096 |
| TPM1 | 2.556644537 | -4.393804742 | 0.974216262 | -4.510091765 | 6.48E-06 | 0.000268347 |
| GAR1 | 6.665566855 | -3.876010636 | 0.860892077 | -4.502318862 | 6.72E-06 | 0.000277334 |
| KHK | 3.271002228 | -5.04785041 | 1.126563584 | -4.480750558 | 7.44E-06 | 0.000302417 |
| TTLL4 | 8.722644414 | -3.76715073 | 0.84087899 | -4.480015286 | 7.46E-06 | 0.000302417 |
| TDRKH | 13.8052569 | -3.949821031 | 0.882909639 | -4.473641307 | 7.69E-06 | 0.000309352 |
| ORC5 | 17.73137368 | -2.788421488 | 0.624261955 | -4.466749043 | 7.94E-06 | 0.000317218 |
| CCNE2 | 60.5144275 | -2.58267713 | 0.578866732 | -4.461609187 | 8.13E-06 | 0.000323776 |
| TCF19 | 98.82813439 | -2.494667888 | 0.559971329 | -4.454992175 | 8.39E-06 | 0.000332749 |
| TUBG1 | 47.51822422 | -2.1547266 | 0.48437246 | -4.44849115 | 8.65E-06 | 0.000341776 |
| POLD1 | 42.63384346 | -2.561667262 | 0.577258259 | -4.437645064 | 9.09E-06 | 0.000356949 |
| ZNF85 | 10.24791584 | -3.700745272 | 0.835064305 | -4.431688975 | 9.35E-06 | 0.000363158 |
| GALE | 7.507579714 | -4.802292971 | 1.088109457 | -4.413428208 | 1.02E-05 | 0.000391151 |
| LIN9 | 4.226545388 | -4.536454288 | 1.031954141 | -4.395984385 | 1.10E-05 | 0.000421052 |
| CCR2 | 1.356851066 | 3.745668506 | 0.852239422 | 4.395089467 | 1.11E-05 | 0.000421362 |
| NELFB | 3.230192442 | -4.740009431 | 1.07877302 | -4.393889486 | 1.11E-05 | 0.000422269 |
| CDPF1 | 3.226985842 | -3.923119908 | 0.893243814 | -4.391992249 | 1.12E-05 | 0.000423351 |
| CCDC34 | 5.512411655 | -3.857949378 | 0.87842878 | -4.391874976 | 1.12E-05 | 0.000423351 |
| COL9A2 | 2.636541618 | -4.423045242 | 1.009624979 | -4.380879372 | 1.18E-05 | 0.000443806 |
| NUDT8 | 5.360403237 | -4.125544408 | 0.943842557 | -4.3710091 | 1.24E-05 | 0.000462811 |
| PRDM10 | 7.743371535 | -3.622177136 | 0.828837773 | -4.3701883 | 1.24E-05 | 0.000463015 |
| PYCR3 | 4.020195664 | -5.144510846 | 1.178460404 | -4.365450744 | 1.27E-05 | 0.000471604 |
| RNASEL | 4.314994898 | -3.101604087 | 0.7111279 | -4.361527776 | 1.29E-05 | 0.000478562 |
| WDR76 | 56.93278964 | -2.171858516 | 0.499227926 | -4.350434746 | 1.36E-05 | 0.000501776 |
| NSDHL | 11.50296632 | -4.347764711 | 1.000239418 | -4.346724026 | 1.38E-05 | 0.000508669 |
| IL10 | 3.698398846 | 5.368473351 | 1.241000979 | 4.32592193 | 1.52E-05 | 0.000554951 |
| SLC41A3 | 9.986120925 | -3.292913517 | 0.761314758 | -4.325298415 | 1.52E-05 | 0.000554951 |
| SYCP2 | 5.575187219 | -4.005102838 | 0.926096563 | -4.324714071 | 1.53E-05 | 0.000554951 |
| TMCO6 | 3.507690411 | -2.458406059 | 0.568859331 | -4.32164144 | 1.55E-05 | 0.000560928 |
| COQ3 | 2.768277981 | -4.652422844 | 1.078433331 | -4.314056984 | 1.60E-05 | 0.000577106 |
| CBX1 | 6.768933077 | -2.778583628 | 0.644092868 | -4.313948758 | 1.60E-05 | 0.000577106 |
| LDLRAP1 | 5.609959685 | -2.582035694 | 0.598820389 | -4.311870041 | 1.62E-05 | 0.000580704 |
| HSDL1 | 5.481298372 | -3.601530093 | 0.836117822 | -4.307443279 | 1.65E-05 | 0.000590564 |
| ZGRF1 | 47.72775732 | -2.978710318 | 0.692404766 | -4.301978359 | 1.69E-05 | 0.000601504 |
| FSBP | 4.233550096 | -5.350664525 | 1.245507157 | -4.295972525 | 1.74E-05 | 0.000616081 |
| ADAM9 | 1.881724188 | -4.147149815 | 0.970651864 | -4.27254093 | 1.93E-05 | 0.000677329 |
| TIMELESS | 60.9135516 | -2.704995727 | 0.633174484 | -4.272117395 | 1.94E-05 | 0.000677329 |
| AURKB | 48.7922192 | -3.458917944 | 0.81168477 | -4.261405499 | 2.03E-05 | 0.00070827 |
| CDKN3 | 50.47429103 | -2.7627549 | 0.648620883 | -4.259429467 | 2.05E-05 | 0.000710323 |
| C11orf80 | 12.02128689 | -2.434451257 | 0.574309287 | -4.238920237 | 2.25E-05 | 0.0007713 |
| C1R | 4.824815773 | -5.614172862 | 1.325239925 | -4.236344497 | 2.27E-05 | 0.000777826 |
| ALMS1 | 7.231750231 | -3.746812295 | 0.886609778 | -4.225999294 | 2.38E-05 | 0.00080952 |
| MIR3658 | 2.936631618 | -3.346386064 | 0.79217824 | -4.224284254 | 2.40E-05 | 0.000813252 |
| ARHGAP10 | 12.67367177 | -3.067801766 | 0.726372255 | -4.2234567 | 2.41E-05 | 0.000813794 |
| NRM | 63.11895276 | -2.349208076 | 0.556687506 | -4.219976287 | 2.44E-05 | 0.000823985 |
| TWSG1 | 9.518919632 | -3.715136849 | 0.881260157 | -4.215709539 | 2.49E-05 | 0.000837215 |
| CDC45 | 58.9277929 | -2.901406145 | 0.689473049 | -4.208150192 | 2.57E-05 | 0.000860575 |
| KIF4B | 1.093698937 | -3.600985769 | 0.861383049 | -4.180469736 | 2.91E-05 | 0.000966602 |
| POMZP3 | 1.974669835 | -3.593116924 | 0.861957158 | -4.16855628 | 3.07E-05 | 0.001015533 |
| ZFP91-CNTF | 6.691274601 | -3.20308246 | 0.770891138 | -4.155038633 | 3.25E-05 | 0.001074317 |
| SMARCD1 | 5.917959807 | -2.344209314 | 0.565968649 | -4.141941995 | 3.44E-05 | 0.00113423 |
| USP37 | 42.1939208 | -2.077035676 | 0.503236953 | -4.127351265 | 3.67E-05 | 0.001194653 |
| CENPU | 58.15766524 | -2.150602645 | 0.52229249 | -4.117621226 | 3.83E-05 | 0.001242621 |
| PARP2 | 36.44669481 | -3.064340663 | 0.745088839 | -4.112718512 | 3.91E-05 | 0.001265669 |
| GGT1 | 11.63784662 | -3.212891293 | 0.783415184 | -4.101134826 | 4.11E-05 | 0.001315613 |
| PIMREG | 4.598306632 | -5.677304572 | 1.388155469 | -4.08981897 | 4.32E-05 | 0.001369798 |
| LINC01260 | 2.027735739 | 4.219111466 | 1.032339668 | 4.086941145 | 4.37E-05 | 0.001382998 |
| RECQL4 | 3.664205509 | -4.355860982 | 1.066091962 | -4.085821052 | 4.39E-05 | 0.001385794 |
| GBE1 | 11.52955995 | -3.430493227 | 0.839914156 | -4.084337906 | 4.42E-05 | 0.00138735 |
| DIAPH3-AS1 | 2.041519923 | -4.505931749 | 1.10324222 | -4.084263335 | 4.42E-05 | 0.00138735 |
| LONP1 | 4.472523629 | -2.73531686 | 0.670114464 | -4.081865127 | 4.47E-05 | 0.001393982 |
| PUS3 | 1.945354006 | -4.21575179 | 1.036379098 | -4.067769986 | 4.75E-05 | 0.00147286 |
| GORAB | 2.79781006 | -4.126759552 | 1.015001477 | -4.065767041 | 4.79E-05 | 0.001481486 |
| TPX2 | 78.89317224 | -2.462630287 | 0.606527129 | -4.060214574 | 4.90E-05 | 0.001513006 |
| ACER3 | 6.574716267 | -3.493786768 | 0.860686544 | -4.059302185 | 4.92E-05 | 0.00151478 |
| INTS12 | 10.76556603 | -3.16617624 | 0.782063872 | -4.048488047 | 5.15E-05 | 0.001582164 |
| TNFAIP1 | 7.207876085 | -2.865710021 | 0.709518717 | -4.038949153 | 5.37E-05 | 0.001643418 |
| NCAPG | 53.73853289 | -2.596657514 | 0.643182451 | -4.037202059 | 5.41E-05 | 0.001650815 |
| RCCD1 | 2.268815111 | -4.637101796 | 1.15343139 | -4.020266689 | 5.81E-05 | 0.001755502 |
| DGCR6 | 2.653131249 | -3.826975559 | 0.953026514 | -4.015602403 | 5.93E-05 | 0.0017858 |
| UCK2 | 16.62376597 | -2.93953104 | 0.732978411 | -4.01039239 | 6.06E-05 | 0.00181806 |
| GSTCD | 3.132824167 | -4.872483106 | 1.21504691 | -4.010119335 | 6.07E-05 | 0.00181806 |
| CCDC51 | 4.285022415 | -4.521372147 | 1.127987546 | -4.008352895 | 6.11E-05 | 0.001826851 |
| SGO2 | 24.42430204 | -3.441373147 | 0.862615137 | -3.989465291 | 6.62E-05 | 0.001968151 |
| SLC4A8 | 4.90606662 | -3.926712403 | 0.988046792 | -3.974217046 | 7.06E-05 | 0.002082105 |
| PLXDC1 | 10.59224067 | 3.270529352 | 0.824895897 | 3.964778299 | 7.35E-05 | 0.002160576 |
| WDR34 | 50.74411295 | -2.409905377 | 0.60851237 | -3.96032274 | 7.48E-05 | 0.002189854 |
| GATB | 5.975148847 | -3.605477592 | 0.912108202 | -3.952905568 | 7.72E-05 | 0.002250815 |
| CCSAP | 1.774072609 | -4.158196086 | 1.05203614 | -3.952522094 | 7.73E-05 | 0.002250815 |
| LRP8 | 5.953812158 | -3.67671753 | 0.931928543 | -3.945278378 | 7.97E-05 | 0.002308029 |
| CDCA7 | 66.25628749 | -2.194669968 | 0.556596672 | -3.943016692 | 8.05E-05 | 0.002323938 |
| SMTN | 4.394736361 | -4.972067186 | 1.263245832 | -3.935945847 | 8.29E-05 | 0.002387324 |
| INAFM1 | 2.177534645 | -3.893785283 | 0.994427256 | -3.915605953 | 9.02E-05 | 0.002571541 |
| RAD54B | 21.87680553 | -3.305282269 | 0.845529528 | -3.909126957 | 9.26E-05 | 0.002634817 |
| DTYMK | 32.72431821 | -2.065168414 | 0.529176277 | -3.902609591 | 9.52E-05 | 0.002699987 |
| JHY | 3.833586709 | 5.381600481 | 1.379484259 | 3.901168459 | 9.57E-05 | 0.002709289 |
| POLD3 | 48.97270303 | -2.104434148 | 0.541200011 | -3.888459176 | 0.000100883 | 0.002847975 |
| FASN | 4.625891869 | -3.329225936 | 0.858394549 | -3.878433223 | 0.000105131 | 0.0029605 |
| C17orf58 | 4.284471516 | -5.538173894 | 1.429774005 | -3.873461033 | 0.000107301 | 0.003014049 |
| GOLGA2P5 | 2.637574026 | -3.330990763 | 0.860909486 | -3.869153284 | 0.000109214 | 0.003060165 |
| HPSE | 15.6113087 | -3.383901599 | 0.875498882 | -3.865112415 | 0.000111038 | 0.003095873 |
| ICA1L | 2.180856051 | -2.994198301 | 0.77536461 | -3.861664901 | 0.000112617 | 0.003124427 |
| ZNF726 | 1.977290675 | -4.330677056 | 1.123941972 | -3.853114451 | 0.000116625 | 0.003227671 |
| CGREF1 | 2.59991447 | -4.813803674 | 1.251174912 | -3.847426628 | 0.000119365 | 0.003271974 |
| HPGD | 54.78400736 | -2.431861416 | 0.632427875 | -3.845278663 | 0.000120416 | 0.003292137 |
| SLC48A1 | 5.026614703 | -2.969413725 | 0.773465679 | -3.839102118 | 0.000123485 | 0.003359748 |
| ZNF845 | 2.288592436 | -2.847423681 | 0.743293956 | -3.830817749 | 0.000127718 | 0.003466546 |
| SLC25A28 | 1.978215332 | -4.214653312 | 1.101586522 | -3.825984819 | 0.00013025 | 0.003526778 |
| CENPE | 56.7528746 | -2.337409949 | 0.612242774 | -3.81778283 | 0.000134656 | 0.003628635 |
| RMND5B | 1.539524727 | 4.213417539 | 1.104374113 | 3.815208533 | 0.000136068 | 0.003649214 |
| IDNK | 6.751877599 | -3.28455312 | 0.861159435 | -3.814105713 | 0.000136677 | 0.003651773 |
| DHX33 | 2.978071823 | -3.714761885 | 0.978635172 | -3.795859778 | 0.000147133 | 0.003908731 |
| STAM2 | 14.13886806 | -2.406868122 | 0.634191693 | -3.795174469 | 0.00014754 | 0.003910322 |
| ZNF826P | 2.333565195 | -4.492827729 | 1.185085786 | -3.79114135 | 0.000149957 | 0.003955763 |
| WDR12 | 12.39588339 | -3.117999022 | 0.822727545 | -3.789831813 | 0.000150749 | 0.003967384 |
| TP73 | 14.75764463 | -2.985859012 | 0.788883022 | -3.784919852 | 0.000153758 | 0.004037136 |
| SLC39A10 | 10.66719511 | 2.789247631 | 0.737136329 | 3.783896575 | 0.000154392 | 0.004044352 |
| RRM2B | 7.000649016 | -3.022189032 | 0.799198508 | -3.781524868 | 0.000155871 | 0.004073612 |
| PCYOX1 | 7.33494002 | -2.686262316 | 0.711186568 | -3.777155583 | 0.00015863 | 0.004136121 |
| SFR1 | 5.661122985 | -3.716937671 | 0.988370713 | -3.760671599 | 0.000169458 | 0.004387983 |
| SHC4 | 2.310555172 | -4.682471268 | 1.247431829 | -3.753689107 | 0.000174251 | 0.00450175 |
| GSEC | 2.885311699 | -4.88675666 | 1.30367814 | -3.748437983 | 0.000177939 | 0.004586518 |
| HEBP1 | 4.656916557 | -3.941352668 | 1.052331243 | -3.745353657 | 0.00018014 | 0.004632637 |
| PHF1 | 24.18923506 | 2.253248291 | 0.602011434 | 3.742866271 | 0.000181933 | 0.004657486 |
| GNG4 | 6.393639018 | -3.904151787 | 1.043582719 | -3.741104291 | 0.000183213 | 0.004679629 |
| FBXL18 | 1.500208829 | -3.038722664 | 0.814517438 | -3.730703017 | 0.000190946 | 0.00485512 |
| FDXACB1 | 4.397294977 | -3.988107312 | 1.069261455 | -3.729777496 | 0.000191649 | 0.004862013 |
| RNF138P1 | 2.485193211 | -2.189604004 | 0.589721704 | -3.712944577 | 0.000204862 | 0.005185535 |
| POLE2 | 24.28533742 | -2.54694464 | 0.686189938 | -3.711719598 | 0.000205856 | 0.005199017 |
| KIAA0355 | 4.464665334 | -2.785902177 | 0.750882194 | -3.710172113 | 0.000207118 | 0.005219198 |
| KIF11 | 79.37117953 | -2.337752878 | 0.630650646 | -3.706890483 | 0.00020982 | 0.005263715 |
| WDCP | 3.416502131 | -3.530566734 | 0.953053803 | -3.704477882 | 0.000211827 | 0.005293473 |
| HSDL2 | 18.67927889 | -3.153143805 | 0.851203657 | -3.704335362 | 0.000211946 | 0.005293473 |
| CBR3 | 3.368120875 | -4.974136271 | 1.346448243 | -3.694264741 | 0.000220524 | 0.005495535 |
| C20orf194 | 7.691131016 | 3.233372198 | 0.875766142 | 3.692049786 | 0.000222454 | 0.005531392 |
| DHRS7B | 3.678997404 | -4.081772375 | 1.10615558 | -3.690052692 | 0.000224208 | 0.005562719 |
| LMO7 | 6.005954242 | -3.640164416 | 0.98755882 | -3.68602289 | 0.000227786 | 0.005626711 |
| NDC1 | 30.16660369 | -2.830710441 | 0.769158198 | -3.680270779 | 0.000232986 | 0.00574258 |
| FANCG | 44.31925122 | -2.310137095 | 0.628954933 | -3.672977146 | 0.000239741 | 0.005896161 |
| MAGEF1 | 4.24476072 | -4.200246343 | 1.146382989 | -3.663911958 | 0.000248392 | 0.006056038 |
| KIR3DL1 | 3.049336577 | -4.106607701 | 1.121206223 | -3.662669381 | 0.000249601 | 0.006060503 |
| ZNF816 | 1.094330135 | 3.31571709 | 0.905361372 | 3.662313405 | 0.000249948 | 0.006060503 |
| RNF146 | 8.528640124 | -2.696754465 | 0.737334245 | -3.657438243 | 0.000254749 | 0.0061577 |
| SPTSSA | 14.29148919 | -3.590414359 | 0.98203105 | -3.656110832 | 0.000256071 | 0.006176403 |
| DIAPH2 | 11.81370727 | -2.974025671 | 0.814078713 | -3.653240926 | 0.000258951 | 0.006229303 |
| ARHGAP33 | 3.994812438 | -3.311635084 | 0.906595346 | -3.652826034 | 0.00025937 | 0.006229303 |
| CRK | 6.320005459 | -3.022533689 | 0.828919684 | -3.646352896 | 0.000265989 | 0.006374672 |
| DHX58 | 7.892701276 | -3.141344595 | 0.861985318 | -3.644313343 | 0.000268107 | 0.00641179 |
| CEP131 | 4.85902372 | -4.101403687 | 1.127429067 | -3.637837456 | 0.000274937 | 0.006561205 |
| PAQR4 | 29.29012043 | -2.209766751 | 0.607632327 | -3.636683983 | 0.00027617 | 0.006576709 |
| UBXN6 | 2.406515966 | -2.796303789 | 0.769696478 | -3.632995432 | 0.00028015 | 0.006657404 |
| ZNF813 | 0.942144122 | -2.862231481 | 0.788100754 | -3.631809088 | 0.000281441 | 0.00667401 |
| TOM1L1 | 2.118663847 | -2.334918264 | 0.643753147 | -3.627039767 | 0.000286689 | 0.006763973 |
| CD80 | 7.216054472 | -3.132179612 | 0.863606383 | -3.62686019 | 0.000286889 | 0.006763973 |
| LINC00888 | 3.890552067 | -4.16949591 | 1.152110147 | -3.619008062 | 0.000295734 | 0.00695439 |
| KIF1BP | 21.08090542 | -3.063789004 | 0.848164717 | -3.612257079 | 0.000303543 | 0.007112669 |
| SLC1A4 | 36.73949844 | -2.222835054 | 0.61538591 | -3.612099363 | 0.000303728 | 0.007112669 |
| APOOL | 7.967821204 | -2.95584853 | 0.82061353 | -3.601998288 | 0.00031578 | 0.007349072 |
| CENPA | 3.149145138 | -5.111210481 | 1.419483392 | -3.600753985 | 0.000317296 | 0.007363976 |
| GCLC | 2.827634362 | 3.811724005 | 1.058694681 | 3.600399694 | 0.000317728 | 0.007363976 |
| PTCHD3P1 | 2.369785833 | -3.637867698 | 1.011897321 | -3.59509569 | 0.000324272 | 0.007484844 |
| B4GALT7 | 4.876092672 | -3.51476351 | 0.97786724 | -3.594315635 | 0.000325245 | 0.00749195 |
| STRIP2 | 2.198847709 | -4.18095298 | 1.163956078 | -3.592019543 | 0.000328125 | 0.007542863 |
| CEP57L1 | 11.33846233 | -3.364685929 | 0.937082648 | -3.590596769 | 0.000329922 | 0.007565157 |
| HIST1H2AJ | 0.81987004 | -2.852222593 | 0.794449158 | -3.590188958 | 0.000330438 | 0.007565157 |
| RHD | 2.765540518 | 3.143848109 | 0.876782199 | 3.585665986 | 0.000336219 | 0.007644461 |
| ST7-AS2 | 1.890080954 | -3.771489931 | 1.052019767 | -3.584999113 | 0.000337079 | 0.007644461 |
| TMEM201 | 8.161906797 | -4.34580059 | 1.212446657 | -3.584323126 | 0.000337953 | 0.007644461 |
| SHPK | 1.478702893 | -3.734810318 | 1.042702896 | -3.581854746 | 0.000341163 | 0.007701133 |
| TRMT6 | 4.731637878 | -2.663554081 | 0.745451778 | -3.573073616 | 0.000352816 | 0.007948228 |
| CIDEB | 7.450610151 | -2.863896597 | 0.805109264 | -3.557152705 | 0.000374896 | 0.008345516 |
| BFSP2 | 1.479614548 | -3.453782769 | 0.971342238 | -3.555680619 | 0.000377002 | 0.008375836 |
| HIST1H1B | 9.694074998 | -2.993710254 | 0.842376843 | -3.553884796 | 0.000379585 | 0.008416635 |
| CEP72 | 1.898195018 | -4.31975886 | 1.215746842 | -3.553173004 | 0.000380614 | 0.008422862 |
| XYLB | 2.375941431 | -4.446268493 | 1.252926511 | -3.548706533 | 0.000387128 | 0.008516822 |
| BRCA2 | 48.8904471 | -2.354743069 | 0.663718805 | -3.547802246 | 0.00038846 | 0.008529456 |
| PLCD1 | 1.207800846 | 3.615681584 | 1.01931694 | 3.547161283 | 0.000389406 | 0.0085336 |
| MSANTD3 | 2.632988195 | -3.770117317 | 1.067264151 | -3.532506281 | 0.00041164 | 0.008971582 |
| CENPH | 22.49863431 | -2.56660607 | 0.72658666 | -3.532415623 | 0.000411782 | 0.008971582 |
| AGAP3 | 2.702677102 | -4.414341447 | 1.250542019 | -3.52994252 | 0.00041565 | 0.009038381 |
| MVK | 3.258506669 | -4.544050072 | 1.29317295 | -3.513876524 | 0.000441618 | 0.009547765 |
| CLN6 | 14.18107011 | -2.81396534 | 0.809837798 | -3.474727098 | 0.000511373 | 0.010847663 |
| PDP1 | 9.356681134 | -2.361694366 | 0.681165361 | -3.467138088 | 0.000526032 | 0.011114058 |
| QSER1 | 2.576777832 | -3.67737273 | 1.060770708 | -3.466698979 | 0.000526892 | 0.011114058 |
| RASD1 | 3.191313684 | -4.943315694 | 1.426232827 | -3.465994891 | 0.000528273 | 0.011122375 |
| CHAF1B | 25.21253063 | -2.731560237 | 0.790616994 | -3.454972835 | 0.000550348 | 0.011543991 |
| RBFA | 4.649067244 | -3.11569947 | 0.902586918 | -3.451966129 | 0.000556518 | 0.011651699 |
| RFC5 | 48.74748534 | -2.089896768 | 0.605518755 | -3.451415419 | 0.000557655 | 0.011653841 |
| PABPC1L | 4.123792191 | -3.350525336 | 0.972286016 | -3.446028514 | 0.00056889 | 0.011844696 |
| NR1D2 | 5.627682806 | 2.880836715 | 0.837587879 | 3.439444133 | 0.00058291 | 0.012072548 |
| CEP112 | 4.547125606 | -4.630341659 | 1.350813168 | -3.427817976 | 0.000608453 | 0.012569286 |
| ZNF200 | 11.36020555 | -2.802033823 | 0.817576938 | -3.427241759 | 0.000609746 | 0.012569286 |
| PDIK1L | 5.226459078 | -3.234724561 | 0.944970704 | -3.423095073 | 0.000619124 | 0.01272594 |
| MIR25 | 1.452262432 | -3.77019457 | 1.103470365 | -3.416670434 | 0.00063392 | 0.013006323 |
| RRP1 | 12.12875037 | -2.712140088 | 0.795206492 | -3.410611099 | 0.000648175 | 0.013274618 |
| LINC00665 | 2.056006914 | -3.871488309 | 1.137105572 | -3.40468678 | 0.0006624 | 0.013541328 |
| ETFRF1 | 7.330037057 | -2.390818992 | 0.702858399 | -3.401565658 | 0.00067001 | 0.013647371 |
| BAG2 | 4.082256605 | -3.580919948 | 1.054941567 | -3.394424925 | 0.000687729 | 0.013932696 |
| CTSL | 1.225658031 | -3.523288556 | 1.038661634 | -3.392142773 | 0.000693483 | 0.014024043 |
| CCNF | 24.67304563 | -2.53571853 | 0.747918823 | -3.390365976 | 0.000697994 | 0.014056818 |
| RCC2 | 23.09510423 | -2.07762179 | 0.612829202 | -3.390213428 | 0.000698382 | 0.014056818 |
| ZCCHC3 | 2.01747968 | -3.783290849 | 1.117360777 | -3.385916998 | 0.000709408 | 0.014193208 |
| DNA2 | 13.80647575 | -3.032118074 | 0.896154782 | -3.383475863 | 0.000715745 | 0.014294595 |
| ST7-OT3 | 2.097021871 | -4.061046699 | 1.201144889 | -3.380979878 | 0.000722278 | 0.014374104 |
| FANCB | 3.329775365 | -2.974595292 | 0.883422497 | -3.367126492 | 0.000759558 | 0.015089358 |
| ANKRD20A5P | 2.258306939 | 4.723521496 | 1.407301409 | 3.356439116 | 0.000789531 | 0.015629662 |
| RAD18 | 33.59390392 | -2.055328373 | 0.613475613 | -3.350301673 | 0.000807236 | 0.015868596 |
| NEK6 | 1.892535593 | -3.210097166 | 0.958799665 | -3.34803743 | 0.00081386 | 0.015970943 |
| ZNF253 | 6.53588563 | -2.593290247 | 0.775422824 | -3.344356354 | 0.000824737 | 0.016156244 |
| MOB3C | 16.27111382 | 2.595250478 | 0.777942142 | 3.336045625 | 0.000849792 | 0.01650355 |
| MLLT11 | 5.039185717 | -3.53546398 | 1.06011122 | -3.334993456 | 0.000853014 | 0.016537608 |
| PM20D2 | 21.59825759 | -2.709166221 | 0.814299362 | -3.326990473 | 0.000877894 | 0.016935611 |
| PKD2 | 5.879721516 | -3.061236908 | 0.92120348 | -3.323084392 | 0.00089028 | 0.017112818 |
| PRC1 | 55.83654583 | -2.111358326 | 0.635850759 | -3.320524977 | 0.000898483 | 0.01721176 |
| GDAP1 | 19.06594396 | -2.932079577 | 0.884861531 | -3.313602722 | 0.000921023 | 0.017583728 |
| POLA1 | 46.96715282 | -2.04855363 | 0.620521884 | -3.301339862 | 0.000962242 | 0.018277739 |
| ILVBL | 27.13884823 | -2.395277565 | 0.72801977 | -3.290127085 | 0.001001421 | 0.018831404 |
| PHF7 | 1.948016452 | -4.355422356 | 1.324184199 | -3.289136328 | 0.001004953 | 0.018866324 |
| PMCH | 3.56589412 | -3.725158667 | 1.134151273 | -3.28453422 | 0.001021511 | 0.019145256 |
| PURB | 3.576653383 | -3.487896609 | 1.063556459 | -3.279465401 | 0.00104004 | 0.019460143 |
| TM6SF1 | 4.413022269 | -4.017482293 | 1.225862837 | -3.277269015 | 0.001048165 | 0.019579644 |
| ERN2 | 1.581675134 | -3.348567811 | 1.023791592 | -3.270751428 | 0.001072621 | 0.019937307 |
| EXOC6B | 2.159607307 | -3.87578362 | 1.18516346 | -3.270252375 | 0.001074516 | 0.019939613 |
| TXNDC16 | 5.238005064 | -3.16939563 | 0.969852615 | -3.267914712 | 0.00108343 | 0.020055917 |
| LINC00612 | 5.479322937 | 2.719714371 | 0.83367548 | 3.262317816 | 0.001105052 | 0.020353172 |
| PLA2G12A | 3.294884003 | -3.726624564 | 1.142394648 | -3.262116617 | 0.001105836 | 0.020353172 |
| BRCA1 | 39.79408144 | -2.072624853 | 0.63583264 | -3.259701884 | 0.001115294 | 0.020424584 |
| BMP2K | 1.40275962 | -3.810540754 | 1.169636675 | -3.257884122 | 0.001122462 | 0.02046162 |
| FLJ37453 | 3.746060661 | -2.086188171 | 0.640358866 | -3.257842253 | 0.001122628 | 0.02046162 |
| BIK | 2.325909507 | -4.594437605 | 1.410863228 | -3.256472713 | 0.001128058 | 0.02052737 |
| LRWD1 | 4.56920257 | -3.19999936 | 0.983067723 | -3.255115884 | 0.001133461 | 0.020592428 |
| NUFIP1 | 3.499952824 | -2.552666707 | 0.785238028 | -3.250819008 | 0.001150731 | 0.020872517 |
| GATAD1 | 4.724161363 | -2.733444684 | 0.841496294 | -3.248314582 | 0.001160908 | 0.021023268 |
| ZNF680 | 5.078743318 | -2.389020361 | 0.736177025 | -3.245171038 | 0.001173801 | 0.021204099 |
| SPIN4 | 19.28662574 | -3.199739065 | 0.987810542 | -3.239223443 | 0.001198556 | 0.021566356 |
| FOXJ2 | 5.814438273 | -2.432040413 | 0.751366189 | -3.236824399 | 0.001208678 | 0.021679211 |
| PIGA | 2.577973769 | -3.731976338 | 1.157763507 | -3.223435802 | 0.001266627 | 0.022610594 |
| ZNF155 | 0.790148219 | -2.744502248 | 0.853230855 | -3.216599859 | 0.001297194 | 0.02301038 |
| COPRS | 6.560775298 | -3.193550427 | 0.993124994 | -3.215658097 | 0.001301458 | 0.023049719 |
| ACSM3 | 1.889164044 | -4.353504762 | 1.356160682 | -3.210168839 | 0.00132657 | 0.023420827 |
| SLC4A10 | 0.799840969 | 2.991611241 | 0.932291771 | 3.208878737 | 0.001332537 | 0.02348935 |
| ZKSCAN4 | 1.90839953 | -3.706895391 | 1.158382341 | -3.200062069 | 0.00137398 | 0.024144322 |
| BBS9 | 16.48822928 | 2.454044586 | 0.767003282 | 3.199522926 | 0.001376552 | 0.024151847 |
| SUV39H2 | 31.44393424 | -2.482739942 | 0.776084314 | -3.199059557 | 0.001378767 | 0.024153079 |
| EME1 | 2.582340767 | -2.835586444 | 0.887413628 | -3.195337953 | 0.001396672 | 0.024428746 |
| ZNF100 | 17.15116771 | -2.359611562 | 0.739254434 | -3.19188016 | 0.0014135 | 0.024675422 |
| MRPS31P5 | 2.457898624 | -3.13013152 | 0.981969962 | -3.187604143 | 0.001434568 | 0.024898263 |
| ATG4A | 11.79586766 | -2.658919766 | 0.834646111 | -3.185685203 | 0.001444116 | 0.025025426 |
| KLHL25 | 1.991637281 | -3.313482889 | 1.040650334 | -3.184050184 | 0.001452298 | 0.025081226 |
| BDH1 | 5.050656583 | -2.730261322 | 0.857546734 | -3.183804698 | 0.00145353 | 0.025081226 |
| B3GNT9 | 1.983838483 | -3.126269372 | 0.98300714 | -3.180311969 | 0.001471166 | 0.025299559 |
| ULBP2 | 3.282505332 | -4.385564578 | 1.379453618 | -3.179204085 | 0.001476801 | 0.025335998 |
| PGBD2 | 1.45707565 | -3.776495276 | 1.188502103 | -3.177525111 | 0.001485378 | 0.025427509 |
| TMEM101 | 11.95260504 | -2.577837939 | 0.81385392 | -3.167445503 | 0.001537845 | 0.026245893 |
| PRC1-AS1 | 19.44179913 | -2.218226899 | 0.70077836 | -3.165375851 | 0.001548827 | 0.026393331 |
| PRRG4 | 10.28982758 | -2.971390409 | 0.941779858 | -3.155079591 | 0.001604543 | 0.027260295 |
| DPY19L2P2 | 1.921293355 | -4.417401972 | 1.400363094 | -3.154469001 | 0.001607905 | 0.027276261 |
| FADS2 | 2.687518253 | -3.090137067 | 0.980541143 | -3.151460894 | 0.001624559 | 0.027517345 |
| PGM3 | 4.451848091 | -2.564923674 | 0.816698849 | -3.140599103 | 0.001686027 | 0.028358468 |
| CXorf56 | 7.300439764 | -2.663303475 | 0.848060538 | -3.140463865 | 0.001686805 | 0.028358468 |
| MPV17L2 | 8.81732282 | -2.653106608 | 0.84577228 | -3.136904189 | 0.001707419 | 0.028662249 |
| RFX2 | 1.576221932 | -4.108578443 | 1.309974511 | -3.136380447 | 0.001710472 | 0.028670762 |
| AMZ1 | 2.293079372 | -3.916929724 | 1.250878778 | -3.131342374 | 0.001740092 | 0.028994664 |
| METTL18 | 9.069349259 | -2.667195156 | 0.853979381 | -3.123254749 | 0.001788629 | 0.029728641 |
| PCTP | 2.053870195 | -4.415697189 | 1.416510791 | -3.117305718 | 0.001825122 | 0.030165582 |
| BCDIN3D | 1.809550909 | -4.270736524 | 1.370102774 | -3.117092094 | 0.001826445 | 0.030165582 |
| FAM173B | 5.192844422 | -2.645621199 | 0.84886361 | -3.116662284 | 0.00182911 | 0.030165582 |
| SNX8 | 9.955935384 | -2.923227705 | 0.93842571 | -3.115033692 | 0.001839239 | 0.030288287 |
| WEE1 | 20.81953267 | -2.032892802 | 0.652725902 | -3.114466266 | 0.00184278 | 0.030302302 |
| RPL13P5 | 2.259839166 | -3.797521105 | 1.220504428 | -3.111435747 | 0.0018618 | 0.030570428 |
| AHCTF1P1 | 2.460094144 | -2.286679811 | 0.735939769 | -3.107156194 | 0.001888966 | 0.030926321 |
| EXTL3 | 2.251158187 | -3.883004657 | 1.25129716 | -3.103183465 | 0.001914509 | 0.031292838 |
| GPAM | 1.77397518 | -4.24209842 | 1.367178331 | -3.102812796 | 0.001916909 | 0.031292838 |
| ROGDI | 5.85609884 | -2.960421364 | 0.955471295 | -3.098388596 | 0.001945761 | 0.031626339 |
| DIXDC1 | 1.66088898 | -4.082827342 | 1.318570837 | -3.096403491 | 0.001958836 | 0.031765071 |
| SAYSD1 | 20.74781725 | -2.095057165 | 0.677404098 | -3.092773088 | 0.001982957 | 0.032024098 |
| RPAP1 | 4.674697329 | -2.521590163 | 0.815338048 | -3.092692865 | 0.001983493 | 0.032024098 |
| TTC39B | 18.33808189 | 2.132509451 | 0.690036288 | 3.090430876 | 0.001998663 | 0.032139107 |
| PLAC8 | 0.756504176 | 2.012609319 | 0.651259755 | 3.0903327 | 0.001999324 | 0.032139107 |
| AFMID | 1.846102025 | -2.631046207 | 0.851786374 | -3.088856887 | 0.002009282 | 0.032240104 |
| CENPP | 26.28566642 | -2.205823044 | 0.71538142 | -3.083422329 | 0.002046345 | 0.032741523 |
| ERCC6L | 3.076232576 | -5.056999169 | 1.64334869 | -3.077252686 | 0.002089181 | 0.033191166 |
| STX2 | 6.728000239 | -2.632266142 | 0.858028037 | -3.067809008 | 0.002156344 | 0.03397071 |
| LOC100506844 | 2.890733706 | -2.869166206 | 0.935888782 | -3.065712788 | 0.002171518 | 0.034161981 |
| SAPCD2 | 2.02411778 | -4.313046209 | 1.407987499 | -3.063270243 | 0.002189323 | 0.03434614 |
| UAP1L1 | 5.067763293 | -3.00546022 | 0.981798051 | -3.061179657 | 0.002204668 | 0.034538771 |
| CALML4 | 2.910675337 | -2.863654593 | 0.936268433 | -3.058582872 | 0.002223866 | 0.034791142 |
| IFT88 | 6.175413051 | -2.448016994 | 0.801364841 | -3.054809582 | 0.002252035 | 0.035134234 |
| ZNF398 | 3.555462509 | -3.015039526 | 0.989152187 | -3.048104797 | 0.002302896 | 0.035779063 |
| MORC4 | 2.173038595 | -3.704079882 | 1.216416777 | -3.045074642 | 0.002326226 | 0.035992591 |
| DCAF15 | 2.664874164 | -2.593178187 | 0.85214902 | -3.043104111 | 0.002341513 | 0.036179428 |
| MED20 | 14.84673903 | -2.562893737 | 0.842569503 | -3.041759439 | 0.002351998 | 0.036291648 |
| AP1S3 | 1.413559984 | -3.697150883 | 1.216563505 | -3.039011828 | 0.002373555 | 0.036574183 |
| GLYCTK | 9.649467142 | -2.093433121 | 0.691270526 | -3.028384754 | 0.002458648 | 0.037782012 |
| FAM185A | 1.539443399 | -3.662023189 | 1.20941383 | -3.02793229 | 0.002462333 | 0.037787076 |
| CEP89 | 11.53218475 | -2.365979284 | 0.783436862 | -3.019999951 | 0.002527747 | 0.038633033 |
| C12orf43 | 3.677315351 | -3.071405771 | 1.020219274 | -3.01053494 | 0.002607879 | 0.03974987 |
| PHEX | 3.46563857 | -4.208484166 | 1.40116481 | -3.003561134 | 0.002668399 | 0.040453365 |
| FAM92A | 1.702527669 | -3.954888388 | 1.317470433 | -3.001880185 | 0.002683178 | 0.040622732 |
| PAPD7 | 6.020208114 | -2.924802555 | 0.974685151 | -3.000766505 | 0.00269301 | 0.040662282 |
| TADA1 | 10.55806143 | -2.268993696 | 0.756724863 | -2.99843947 | 0.002713661 | 0.040864535 |
| ETAA1 | 16.91822915 | -2.199748948 | 0.735560831 | -2.9905738 | 0.002784538 | 0.04170883 |
| HIST1H2AM | 0.806281229 | -2.465544228 | 0.824793486 | -2.989286739 | 0.002796296 | 0.041829317 |
| XRCC3 | 6.627236028 | -2.504930854 | 0.838155656 | -2.988622503 | 0.002802381 | 0.041864752 |
| PACS2 | 2.034813022 | -2.207938919 | 0.739640238 | -2.98515252 | 0.00283437 | 0.042230607 |
| ZNF252P | 1.097739336 | 3.456758532 | 1.159487834 | 2.981280553 | 0.002870457 | 0.042655444 |
| PRICKLE3 | 1.637953146 | -3.662460512 | 1.228780674 | -2.980564872 | 0.002877173 | 0.042698914 |
| AZIN2 | 4.561797213 | 3.774074333 | 1.26670794 | 2.979435286 | 0.002887802 | 0.042754133 |
| ZMYND19 | 2.306695933 | -4.525725684 | 1.519024674 | -2.979362852 | 0.002888485 | 0.042754133 |
| USP45 | 3.188605139 | -2.30286956 | 0.773863265 | -2.975809377 | 0.002922164 | 0.043073193 |
| C1orf74 | 1.251036738 | -3.495930768 | 1.176624018 | -2.971153669 | 0.002966833 | 0.043400527 |
| BAIAP3 | 1.36367293 | -2.227770776 | 0.750188321 | -2.969615379 | 0.002981728 | 0.043505646 |
| GALNT3 | 2.418317882 | 2.98368958 | 1.004924374 | 2.969068776 | 0.002987037 | 0.043526505 |
| ATP2A1 | 0.705679792 | -2.231631189 | 0.752100467 | -2.967198248 | 0.003005271 | 0.043735625 |
| DHODH | 5.956332236 | -3.366153957 | 1.13675252 | -2.961202107 | 0.003064408 | 0.044424053 |
| DCAF4 | 2.481084276 | -3.185439968 | 1.077680237 | -2.955830364 | 0.003118285 | 0.045095463 |
| BTN2A3P | 1.802574187 | -2.880084608 | 0.974423419 | -2.955680819 | 0.003119798 | 0.045095463 |
| CRAMP1 | 5.028978018 | -2.681979787 | 0.907487372 | -2.955390752 | 0.003122733 | 0.045095463 |
| KBTBD6 | 4.020586042 | -2.630898987 | 0.89291897 | -2.946402839 | 0.003214934 | 0.046249064 |
| NIPBL-AS1 | 5.238574686 | -2.62493346 | 0.891515155 | -2.944350912 | 0.003236328 | 0.046497451 |
| IRF5 | 13.57550428 | -2.231689724 | 0.759129265 | -2.939801989 | 0.003284221 | 0.047125427 |
| COX18 | 7.19393786 | -2.720946047 | 0.925730948 | -2.939240664 | 0.003290175 | 0.047150801 |
| PDGFD | 1.309324137 | -3.241350888 | 1.103805696 | -2.936523066 | 0.003319141 | 0.047505474 |
| TSR3 | 6.024885774 | -2.451657071 | 0.836144584 | -2.932097054 | 0.003366815 | 0.04806566 |
| ADCK2 | 4.927633649 | -2.668696559 | 0.910793911 | -2.930077294 | 0.003388777 | 0.048317954 |
| LIPE | 1.068234858 | -2.546445525 | 0.869663833 | -2.928080286 | 0.00341062 | 0.048567914 |
| ENO3 | 2.016370949 | -3.002832403 | 1.026121191 | -2.92639157 | 0.00342919 | 0.048709204 |
| C19orf54 | 2.46670164 | -3.28255807 | 1.122897493 | -2.923292723 | 0.003463507 | 0.04907289 |
| SLC26A2 | 8.885441917 | -2.104317505 | 0.721174155 | -2.917904766 | 0.00352392 | 0.049866118 |
| LAIR1 | 20.24913384 | -4.387773271 | 0.640423695 | -6.851359978 | 7.32E-12 | 7.87E-08 |
| SGK1 | 17.6616951 | 5.263485847 | 0.814678594 | 6.460812749 | 1.04E-10 | 3.64E-07 |
| LDLRAP1 | 11.53569493 | -3.699703044 | 0.57620465 | -6.420814278 | 1.36E-10 | 3.64E-07 |
| JOSD1 | 9.893998071 | 4.39174631 | 0.693818282 | 6.329822118 | 2.45E-10 | 5.28E-07 |
| BMI1 | 5.51657982 | 5.209697894 | 0.84057559 | 6.197774423 | 5.73E-10 | 1.03E-06 |
| ZNF468 | 10.71525533 | 3.633537616 | 0.594996704 | 6.106819741 | 1.02E-09 | 1.56E-06 |
| CD200 | 7.10694426 | -5.604711104 | 0.973947424 | -5.754634147 | 8.68E-09 | 9.23E-06 |
| GUSBP4 | 3.169796531 | -4.136509931 | 0.720591546 | -5.740436388 | 9.44E-09 | 9.23E-06 |
| PNPO | 3.734419321 | -4.521152197 | 0.799359311 | -5.655969892 | 1.55E-08 | 1.31E-05 |
| DNAJB1 | 687.5412958 | 2.067738825 | 0.366682704 | 5.63904107 | 1.71E-08 | 1.31E-05 |
| BICD2 | 4.674422779 | -5.578176008 | 0.990834635 | -5.629774952 | 1.80E-08 | 1.31E-05 |
| FEM1C | 5.668698256 | 4.74917265 | 0.843985871 | 5.627076013 | 1.83E-08 | 1.31E-05 |
| HSPA1B | 106.4129237 | 2.302207103 | 0.418257682 | 5.504279301 | 3.71E-08 | 2.49E-05 |
| FAM118B | 12.90451804 | 2.22226896 | 0.411243264 | 5.403782028 | 6.53E-08 | 4.13E-05 |
| ORC5 | 25.01791656 | -3.358442817 | 0.623147732 | -5.389480926 | 7.07E-08 | 4.22E-05 |
| PRDM10 | 4.302877967 | -4.194369696 | 0.782209726 | -5.362206016 | 8.22E-08 | 4.42E-05 |
| PRMT9 | 16.91010297 | 3.892791934 | 0.728002616 | 5.347222454 | 8.93E-08 | 4.57E-05 |
| BFSP2 | 3.528349966 | -4.744529964 | 0.912426314 | -5.199904791 | 1.99E-07 | 9.75E-05 |
| SLX4 | 4.13720399 | 4.765729184 | 0.933437614 | 5.105567969 | 3.30E-07 | 0.000152062 |
| SLC25A42 | 5.94759291 | -4.072453602 | 0.812263741 | -5.013708475 | 5.34E-07 | 0.000220874 |
| PPP1R3B | 2.410141259 | 4.850430554 | 0.971560554 | 4.992411986 | 5.96E-07 | 0.000231109 |
| TUBGCP5 | 6.974715362 | -3.75927242 | 0.754233621 | -4.984228114 | 6.22E-07 | 0.000231109 |
| IL2RA | 1.159523308 | 2.558081251 | 0.513774084 | 4.979000169 | 6.39E-07 | 0.000231109 |
| AMY2B | 5.584162881 | -2.38476185 | 0.479910029 | -4.969185274 | 6.72E-07 | 0.000233283 |
| KLF3 | 1.684682477 | -3.741077565 | 0.754352185 | -4.959324889 | 7.07E-07 | 0.00023777 |
| REEP3 | 17.38582732 | -2.956462522 | 0.600702155 | -4.921677903 | 8.58E-07 | 0.000279673 |
| RRM2 | 1.694398568 | 3.057367446 | 0.634250122 | 4.820444398 | 1.43E-06 | 0.000440193 |
| STAM2 | 12.70429367 | -3.213529422 | 0.670212352 | -4.794792893 | 1.63E-06 | 0.000473389 |
| FAM200B | 9.171294821 | -3.153581291 | 0.659130766 | -4.784454702 | 1.71E-06 | 0.0004853 |
| ZNF420 | 7.302213785 | -3.983117347 | 0.837187683 | -4.757735249 | 1.96E-06 | 0.000539943 |
| LONP1 | 5.920065313 | -3.210516767 | 0.679460359 | -4.725097979 | 2.30E-06 | 0.000618482 |
| SOCS3 | 13.5104303 | 3.629606502 | 0.784302375 | 4.627815262 | 3.70E-06 | 0.000924374 |
| CRK | 8.247424312 | -3.451253603 | 0.746615459 | -4.62253167 | 3.79E-06 | 0.00092669 |
| NFIL3 | 5.765502457 | 5.157480771 | 1.131300181 | 4.558896797 | 5.14E-06 | 0.001229125 |
| GGT7 | 4.799560205 | -3.436336717 | 0.759852544 | -4.522373115 | 6.12E-06 | 0.00142985 |
| ETFRF1 | 11.77827295 | -3.108756903 | 0.689973819 | -4.505615745 | 6.62E-06 | 0.001514554 |
| GSAP | 3.655706028 | -3.744271268 | 0.832419745 | -4.498056767 | 6.86E-06 | 0.001536705 |
| NTPCR | 11.64584664 | 3.514047712 | 0.787319998 | 4.463303002 | 8.07E-06 | 0.001702101 |
| PLA2G12A | 3.831841061 | -3.988028305 | 0.895038816 | -4.455704305 | 8.36E-06 | 0.001729611 |
| ACTG2 | 6.637089241 | -4.010006191 | 0.902511129 | -4.443165365 | 8.86E-06 | 0.001798991 |
| ZNF671 | 2.007537528 | -4.112571362 | 0.940120129 | -4.374516866 | 1.22E-05 | 0.002380043 |
| TMCO6 | 3.810958344 | -2.620048827 | 0.600476777 | -4.36328086 | 1.28E-05 | 0.00246094 |
| ZNF845 | 2.245369323 | -2.82060484 | 0.651148484 | -4.33173832 | 1.48E-05 | 0.002791592 |
| MRPS31P5 | 3.578806348 | -3.738302814 | 0.872382559 | -4.285164549 | 1.83E-05 | 0.003273481 |
| PDK2 | 5.474261282 | -3.874304568 | 0.915815785 | -4.230440915 | 2.33E-05 | 0.00407398 |
| AMT | 6.059978778 | -4.735502802 | 1.120629723 | -4.22575156 | 2.38E-05 | 0.00407398 |
| A2M-AS1 | 2.340776622 | -3.293429518 | 0.779454305 | -4.225301596 | 2.39E-05 | 0.00407398 |
| LAMP3 | 1.291861455 | 3.400013889 | 0.80632673 | 4.216670191 | 2.48E-05 | 0.004110887 |
| PUS3 | 2.561839866 | -4.60885032 | 1.108034049 | -4.15948438 | 3.19E-05 | 0.005007985 |
| ATP11A | 3.338034677 | -3.887912313 | 0.935078631 | -4.157845322 | 3.21E-05 | 0.005007985 |
| IL10 | 3.877084117 | 4.726722194 | 1.137778835 | 4.15434182 | 3.26E-05 | 0.005012683 |
| DSN1 | 7.790957907 | 3.624520037 | 0.876105298 | 4.137082658 | 3.52E-05 | 0.005328756 |
| TBXAS1 | 2.95113063 | -4.029831351 | 0.976183092 | -4.128151149 | 3.66E-05 | 0.005388192 |
| FOXJ2 | 9.206830675 | -3.173466323 | 0.777064342 | -4.083917062 | 4.43E-05 | 0.006436571 |
| MOGS | 5.153656035 | -3.777155301 | 0.927684066 | -4.071596615 | 4.67E-05 | 0.006608145 |
| SLC26A2 | 15.86612207 | -3.017941721 | 0.744016421 | -4.056283754 | 4.99E-05 | 0.006964815 |
| CDKN1A | 23.41630283 | 2.92098299 | 0.721157788 | 4.050407603 | 5.11E-05 | 0.007050488 |
| BCDIN3D | 3.129558439 | -5.054618745 | 1.249750445 | -4.044502458 | 5.24E-05 | 0.007139037 |
| GGT1 | 4.587482616 | -3.340191581 | 0.82681143 | -4.039846884 | 5.35E-05 | 0.007191207 |
| FCGR3A | 10.690874 | -2.704245706 | 0.670090361 | -4.035643347 | 5.45E-05 | 0.007230805 |
| CSTF2T | 17.455422 | -3.051154165 | 0.758661619 | -4.02175896 | 5.78E-05 | 0.007577098 |
| DIXDC1 | 2.393633599 | -4.605072216 | 1.147653832 | -4.012596907 | 6.01E-05 | 0.007684354 |
| TCF19 | 6.10887219 | 3.277026982 | 0.817459185 | 4.008795843 | 6.10E-05 | 0.007684354 |
| LZTS2 | 3.780064889 | -4.227025965 | 1.054601211 | -4.008174769 | 6.12E-05 | 0.007684354 |
| FBXL18 | 2.773693639 | -4.012161592 | 1.003970692 | -3.996293542 | 6.43E-05 | 0.007954735 |
| EEFSEC | 12.8883873 | 2.91743512 | 0.737310239 | 3.95686234 | 7.59E-05 | 0.00928202 |
| PLAGL1 | 19.91423812 | -2.692566943 | 0.684306071 | -3.934740688 | 8.33E-05 | 0.010065509 |
| FGR | 7.773163661 | -3.181842906 | 0.809241885 | -3.931881144 | 8.43E-05 | 0.010072843 |
| AARS2 | 4.682240257 | -3.289096407 | 0.84476172 | -3.893519711 | 9.88E-05 | 0.011677963 |
| TOM1L1 | 2.561995323 | -2.657318744 | 0.686738849 | -3.869474909 | 0.00010907 | 0.012560782 |
| SRD5A1 | 4.354581241 | -3.900687646 | 1.008474477 | -3.86790914 | 0.000109773 | 0.012560782 |
| ZNF559 | 12.11955693 | -2.697204416 | 0.698483321 | -3.861515854 | 0.000112686 | 0.012685161 |
| CUL7 | 1.937769981 | -3.62022912 | 0.937920467 | -3.859846595 | 0.000113458 | 0.012685161 |
| PLEKHB1 | 1.892404207 | -2.896354862 | 0.750772831 | -3.857831214 | 0.000114398 | 0.012685161 |
| C10orf143 | 2.546619851 | 4.543458234 | 1.179549425 | 3.851859139 | 0.000117224 | 0.012865982 |
| C11orf80 | 7.567759508 | -2.379181492 | 0.619966702 | -3.837595606 | 0.000124245 | 0.013498766 |
| TUBE1 | 3.959342114 | 3.129609142 | 0.81664573 | 3.832272706 | 0.000126965 | 0.013656343 |
| ZNF142 | 6.716261518 | -3.296236562 | 0.862704854 | -3.820816059 | 0.000133011 | 0.014026122 |
| AHCY | 1.354639823 | 3.79712445 | 0.994731825 | 3.81723431 | 0.000134956 | 0.014026322 |
| COQ3 | 1.449960724 | -3.692210202 | 0.967554931 | -3.816021276 | 0.000135621 | 0.014026322 |
| TMEM177 | 1.745156256 | -3.897210176 | 1.023249389 | -3.808661133 | 0.000139721 | 0.014128277 |
| INTS12 | 10.05616385 | -3.026211401 | 0.794852316 | -3.80726248 | 0.000140514 | 0.014128277 |
| DTWD1 | 5.225855283 | -3.10720143 | 0.816137511 | -3.807203307 | 0.000140547 | 0.014128277 |
| ZNF224 | 12.30514491 | -2.481223868 | 0.652603724 | -3.802037558 | 0.000143511 | 0.014292629 |
| TPM1 | 1.311768289 | -3.381724814 | 0.891384277 | -3.793790063 | 0.000148365 | 0.014640508 |
| CEP76 | 1.17107803 | 3.43594628 | 0.908577311 | 3.781677397 | 0.000155775 | 0.015231978 |
| SAYSD1 | 6.807757392 | -3.11106373 | 0.824079532 | -3.775198399 | 0.00015988 | 0.015492551 |
| ZNF780A | 10.66099363 | -2.338523767 | 0.619894698 | -3.772453247 | 0.00016165 | 0.015524199 |
| B3GNT9 | 2.765346542 | -3.648537791 | 0.967907915 | -3.769509199 | 0.000163569 | 0.015569441 |
| METTL4 | 9.462499772 | -2.976653175 | 0.790810642 | -3.764053005 | 0.000167181 | 0.015773714 |
| NAPEPLD | 13.24166957 | -2.895521824 | 0.77780266 | -3.722694681 | 0.000197108 | 0.01843558 |
| SLC25A19 | 1.711036321 | 3.653541103 | 0.983720671 | 3.714002573 | 0.000204007 | 0.018754669 |
| GINS2 | 2.659549247 | 3.457968923 | 0.934185496 | 3.70158704 | 0.000214255 | 0.0195299 |
| C1R | 2.144842701 | -4.424139908 | 1.205294232 | -3.670589131 | 0.000241992 | 0.02182615 |
| ACOT4 | 3.01050466 | -3.037734256 | 0.82872627 | -3.665545991 | 0.000246811 | 0.021939702 |
| ZNF320 | 4.406480013 | -3.155273482 | 0.868705977 | -3.632153532 | 0.000281066 | 0.024779867 |
| MMAA | 2.102374117 | -3.87264962 | 1.067953788 | -3.626233331 | 0.000287586 | 0.024945724 |
| ENOX2 | 7.148903059 | 2.669019292 | 0.736955468 | 3.621683272 | 0.000292692 | 0.02518559 |
| RETSAT | 10.96352781 | -2.995823027 | 0.832374378 | -3.599129319 | 0.000319284 | 0.027255738 |
| C3AR1 | 9.65256079 | -3.001879835 | 0.835751254 | -3.59183408 | 0.000328359 | 0.027592408 |
| LRRC42 | 1.818608623 | 3.826784572 | 1.066645064 | 3.587683195 | 0.000333629 | 0.027696081 |
| PPIEL | 8.955811178 | -3.169090381 | 0.883539015 | -3.586814308 | 0.000334743 | 0.027696081 |
| RCBTB1 | 8.023677236 | -2.874212087 | 0.802893012 | -3.579819532 | 0.000343832 | 0.028230935 |
| GALNT4 | 2.74164004 | -3.290610227 | 0.920425116 | -3.575098256 | 0.000350096 | 0.028527551 |
| NUF2 | 1.814584624 | -4.08060907 | 1.143787237 | -3.567629485 | 0.000360225 | 0.028859356 |
| HACE1 | 4.175059037 | -3.464259959 | 0.976813503 | -3.546490654 | 0.000390399 | 0.030428458 |
| NR4A3 | 13.43790434 | 3.086598213 | 0.871400283 | 3.542112934 | 0.000396935 | 0.03052692 |
| ITGB3BP | 6.314529428 | 2.713767027 | 0.767155708 | 3.537439662 | 0.000404026 | 0.030603584 |
| ATF3 | 7.627136997 | 3.32127642 | 0.941022414 | 3.529433911 | 0.00041645 | 0.031324007 |
| SMARCD1 | 5.987508198 | -2.329867777 | 0.660673527 | -3.526503909 | 0.000421085 | 0.031452706 |
| BYSL | 1.613566525 | 2.94480143 | 0.836211317 | 3.52159959 | 0.000428952 | 0.031819327 |
| BCL6 | 3.266124878 | 3.224026881 | 0.920839488 | 3.501182262 | 0.000463199 | 0.033752703 |
| ZBTB21 | 7.412091878 | 2.71425749 | 0.775705672 | 3.499081661 | 0.000466864 | 0.033752703 |
| RNF146 | 6.963834918 | -2.502230229 | 0.718447145 | -3.482831335 | 0.000496141 | 0.035340999 |
| ROGDI | 7.135437471 | -3.293604686 | 0.948404914 | -3.472783234 | 0.000515091 | 0.036449479 |
| CFL2 | 3.426689402 | -3.600176688 | 1.037852731 | -3.468870466 | 0.000522651 | 0.036742734 |
| POLA2 | 10.78541976 | 2.840795059 | 0.820167937 | 3.463674853 | 0.00053285 | 0.036852784 |
| KLHDC10 | 3.473091189 | -3.862142138 | 1.115124546 | -3.463417742 | 0.00053336 | 0.036852784 |
| DYNC2H1 | 5.224347246 | -3.056043854 | 0.882591926 | -3.46257853 | 0.000535026 | 0.036852784 |
| ANKRD20A5P | 2.140095688 | 4.596120389 | 1.32888285 | 3.458634739 | 0.000542921 | 0.036852784 |
| ST3GAL2 | 6.442031486 | -2.96907888 | 0.858646995 | -3.457857418 | 0.000544489 | 0.036852784 |
| KRBOX4 | 8.792359152 | -2.641437834 | 0.764917385 | -3.453232839 | 0.000553911 | 0.037032171 |
| SBF2 | 1.522311965 | -3.88452168 | 1.124957867 | -3.453037483 | 0.000554312 | 0.037032171 |
| IPO7 | 28.38719582 | 2.477832156 | 0.71995098 | 3.441667869 | 0.00057814 | 0.038252164 |
| ZBTB6 | 1.1077577 | 3.525551074 | 1.02483874 | 3.440103244 | 0.000581492 | 0.038252164 |
| TMEM251 | 3.732282601 | -2.907090086 | 0.845258817 | -3.43928987 | 0.000583242 | 0.038252164 |
| ZNF302 | 32.25926197 | -2.001205232 | 0.583033081 | -3.432404259 | 0.000598255 | 0.038998979 |
| PLAC8 | 6.933904311 | -2.399090278 | 0.700386377 | -3.425381129 | 0.000613938 | 0.039542706 |
| AQP3 | 7.021084309 | 2.020225229 | 0.589782001 | 3.425376198 | 0.000613949 | 0.039542706 |
| TCEAL1 | 1.480785527 | -3.720524724 | 1.087853287 | -3.420061113 | 0.000626071 | 0.040083431 |
| SHQ1 | 10.18447028 | -2.735070951 | 0.801965296 | -3.410460485 | 0.000648533 | 0.041033052 |
| SPIN4 | 5.059818083 | -3.930878504 | 1.158134319 | -3.394147327 | 0.000688426 | 0.042555834 |
| SLC2A6 | 5.338279418 | -3.166484383 | 0.936376983 | -3.381634149 | 0.00072056 | 0.044287701 |
| GPATCH1 | 16.52062973 | -2.548870575 | 0.754448043 | -3.3784574 | 0.000728937 | 0.044548006 |
| PAQR8 | 4.595501091 | -3.299530898 | 0.980739424 | -3.364329829 | 0.000767298 | 0.046365482 |
| ZNF174 | 5.931324587 | -3.363800951 | 1.001147278 | -3.359946158 | 0.000779577 | 0.046844281 |
| HSDL1 | 3.191862911 | -2.711991556 | 0.808497193 | -3.354361129 | 0.000795485 | 0.047138921 |
| CROCCP3 | 2.439612592 | -2.479660144 | 0.739556228 | -3.352902795 | 0.000799688 | 0.047138921 |
| SLC25A4 | 8.414381089 | 2.834606229 | 0.845621008 | 3.352100058 | 0.00080201 | 0.047138921 |
| ZNF615 | 1.546714241 | -3.023829417 | 0.902566968 | -3.35025491 | 0.000807372 | 0.047196172 |
| ZNF287 | 3.035863112 | -4.019433041 | 1.200439758 | -3.348300499 | 0.000813088 | 0.047273374 |
| RIC8B | 5.857946876 | -2.828018315 | 0.845123753 | -3.346277163 | 0.000819045 | 0.047363689 |
| FOXO3 | 3.957226449 | -2.9373062 | 0.878767585 | -3.342529072 | 0.000830187 | 0.047751267 |
| CDPF1 | 2.227026433 | -3.320933515 | 0.995153293 | -3.337107496 | 0.000846552 | 0.048433581 |
| TUBB2A | 2.324572529 | 4.585323994 | 1.377513419 | 3.328696425 | 0.000872535 | 0.049655986 |
| ZWINT | 2.519201391 | 2.692164526 | 0.809234017 | 3.326805929 | 0.000878475 | 0.049716595 |
| GGH | 1.859090381 | 4.428671068 | 1.332107288 | 3.324560347 | 0.000885581 | 0.049716595 |
| DUSP3 | 5.16872245 | 3.61725847 | 1.088468621 | 3.323254709 | 0.000889736 | 0.049716595 |
| ASPM | 0.952597551 | 2.952423626 | 0.88861021 | 3.322518236 | 0.000892088 | 0.049716595 |
